# Chemical and structural studies provide a mechanistic basis for recognition of the MYC G-quadruplex

**DOI:** 10.1038/s41467-018-06315-w

**Published:** 2018-10-12

**Authors:** David R. Calabrese, Xiang Chen, Elena C. Leon, Snehal M. Gaikwad, Zaw Phyo, William M. Hewitt, Stephanie Alden, Thomas A. Hilimire, Fahu He, Aleksandra M. Michalowski, John K. Simmons, Lindsey B. Saunders, Shuling Zhang, Daniel Connors, Kylie J. Walters, Beverly A. Mock, John S. Schneekloth

**Affiliations:** 1 Chemical Biology Laboratory, National Cancer Institute, Frederick, MD 21702 USA; 2 Structural Biophysics Laboratory, National Cancer Institute, Frederick, MD 21702 USA; 3 Laboratory of Cancer Biology and Genetics, National Cancer Institute, Bethesda, MD 20892 USA

## Abstract

G-quadruplexes (G4s) are noncanonical DNA structures that frequently occur in the promoter regions of oncogenes, such as *MYC*, and regulate gene expression. Although G4s are attractive therapeutic targets, ligands capable of discriminating between different G4 structures are rare. Here, we describe DC-34, a small molecule that potently downregulates *MYC* transcription in cancer cells by a G4-dependent mechanism. Inhibition by DC-34 is significantly greater for *MYC* than other G4-driven genes. We use chemical, biophysical, biological, and structural studies to demonstrate a molecular rationale for the recognition of the *MYC* G4. We solve the structure of the *MYC* G4 in complex with DC-34 by NMR spectroscopy and illustrate specific contacts responsible for affinity and selectivity. Modification of DC-34 reveals features required for G4 affinity, biological activity, and validates the derived NMR structure. This work advances the design of quadruplex-interacting small molecules to control gene expression in therapeutic areas such as cancer.

## Abstract (web summary)

Targeting noncoding nucleic acids with small molecules represents an important and significant challenge in chemical biology and drug discovery. Here the authors characterize DC-34, a small molecule that exhibits selective binding to specific G4 structures, and provide a structural basis for its selectivity

## Introduction

In addition to the double helical structure of DNA, the genome is known to fold into a variety of noncanonical three-dimensional structures that offer unique opportunities for small molecule binding^1,2^. Molecules that recognize specific DNA folds that are scarce in the genome could be used to alter gene expression or regulatory pathways controlled by these elements, thus offering potential as therapeutics. To develop chemical probes that bind to folded DNA in the genomic context, small molecules with high affinity and selectivity are needed^3,4^. One barrier to generating such reagents is the paucity of atomic level structural information available for nucleic acid-small molecule complexes, as the resulting mechanistic detail could guide ligand design efforts. Furthermore, many molecules reported to bind to nucleic acids have multiple mechanisms of action, fall far outside “drug-like” chemical space, and/or are characterized by high molecular weights, multiple cationic charges, or intercalating scaffolds^5,6^. While it is routine to characterize protein-binding small molecules using X-ray crystallography or NMR spectroscopy, there are comparatively few structures of “drug-like” small molecules in complex with nucleic acids. Understanding the chemical and structural basis for nucleic acid-small molecule interactions will greatly improve our ability to rationally design selective, high affinity small molecules and further explore nucleic acid-binding compounds as mechanistically novel therapeutics.

Developing small molecules that bind to and alter the function of regulatory nucleic acid sequences is particularly attractive when they govern the expression of so-called “undruggable” proteins, such as MYC^7,8^. The *MYC* gene encodes the transcription factor MYC (also known as c-Myc), which is responsible for affecting the expression of a large number of genes in the human genome^9–13^ and associated with proliferation, differentiation, apoptosis, and oncogenesis. Importantly, *MYC* is upregulated in 70% of all cancers^10^ and linked to ~100,000 deaths per year^14^. However, it has proven difficult to develop efficacious small molecule inhibitors of the MYC protein due to a lack of small molecule binding pockets and a short protein half-life of 20–30 min^7,8^. An attractive alternative route is the prevention of *MYC* transcription via small molecule-mediated stabilization of the G-quadruplex (G4) present within the *MYC* promoter region^15–17^. G4s are non-B DNA structures that occur in G-rich sequences and are characterized by stacks of Hoogsteen-bonded guanine tetrads stabilized by central potassium ions and flanked by loop regions^1,18^. G4s have been identified in genome-wide structural probing studies using a G4-specific antibody^19^, as well as by a chemical probing approach employing ss-DNA seq^20,21^, which together, have identified about 10,000 G4-forming sequences in cells. About 90% of *MYC* expression is regulated by a G4-forming 27 nucleotide sequence found in the CT element (sometimes referred to as the nuclease hypersensitive element III or NHEIII region) of the *MYC* gene (Pu27)^13,14,22,23^. Small molecules that bind and stabilize the *MYC* G4 have been shown to decrease *MYC* expression and present a potential method for targeting cancers where MYC contributes to the oncogenic phenotype^3,4^. However, many of these ligands that silence *MYC* expression in cells are not selective, and therefore their activity cannot always be attributed to a MYC-dependent mechanism of action^24^.

As with many nucleic acids, there are few structures of small molecule ligands bound to G4s. As a method to provide insights for rational design for small molecule ligands, Yang and co-workers solved an NMR structure of the *MYC* G4 (Pu22 G14T/G23T)^25^ and later in complex with a ligand^26^. Others have also reported small molecule ligands for G4s, and they are a target of considerable interest^3,4,16,27^. In one example, CX-5461 was found to target BRCA1/2-deficient tumors and is reported to stabilize G4 structures and dsDNA, and is being investigated in phase I clinical trials^28^. Another example, Quarfloxin (CX-3552, Cylene Pharmaceuticals, Tetragene), is reported to act through the inhibition of rRNA biogenesis by disrupting interaction between nucleolin and ribosomal G4 DNA and advanced to phase II trials^29^. Despite considerable efforts, a G4-modulating drug is not yet available, and molecules that discriminate between different G4 structures have proved challenging to develop^29^.

In this study, we report the discovery of a drug-like compound with dramatic effects on MYC expression in multiple myeloma cells, demonstrate that it acts by a G4-dependent mechanism of action, and solve a complete structure by NMR of the compound in complex with the *MYC* G4. We synthesize a focused library of analogs^30^, which are evaluated for their affinity, and their ability to silence *MYC* and limit cell growth in a MYC-driven multiple myeloma cell line. The most potent analog (DC-34) inhibits *MYC* at the transcriptional level only when a G4 is present in the promoter. Importantly, DC-34 does not transcriptionally downregulate several other G4-dependent genes to the same extent. To establish a structural basis for this selectivity, we synthesized an isotopically labeled DC-34 for use in NMR studies. This probe, along with an unlabeled version, is used to solve the NMR structure of DC-34 in complex with the G4. DC-34 adopts a three-dimensional conformation that enables specific contacts with the G4 that govern selectivity and biological activity. Insights gained from this structure and the corresponding chemical derivatives provide a basis for the recognition of the *MYC* G4 and have implications for the development of selective nucleic acid-binding compounds with biological activity.

## Results

### Structure of DC-34 influences binding and cellular activity

**Fig. 1:**
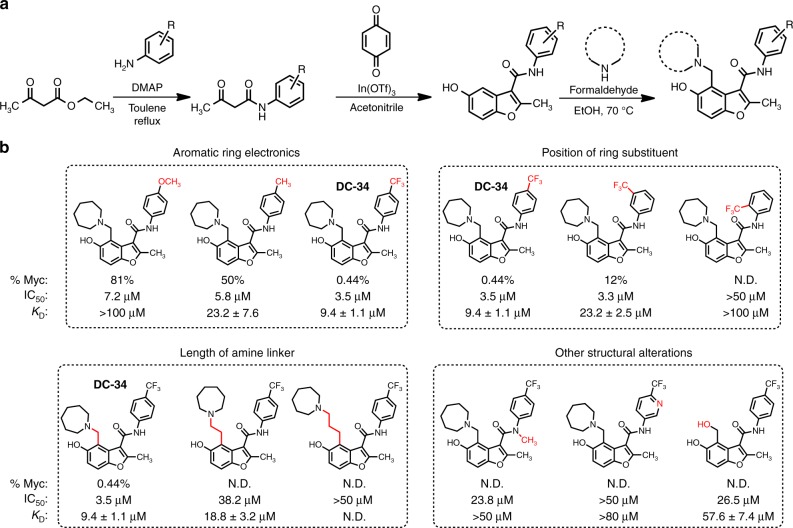
Structure–activity relationship of DC-34. **a** Synthesis scheme of MYC G4-binding scaffold. **b** Table of selected analogs, with the parent compound on the left. %Myc indicates to the percent of MYC protein expressed at a 10 µM dose in L363 MM cells, IC_50_ is the half maximum concentration for cytotoxicity in L363 MM cells at 48 h, and *K*_D_ determined by a fluorescence intensity assay. N. D. indicates that value was not determined

As part of an effort to understand the factors that govern molecular recognition and selectivity for G4 structures we designed an efficient, flexible, and scalable synthetic route that enabled the generation of a focused library of 25 compounds (Fig. 1a, b, and Supplementary Table 1)^31,32^. Most compounds were evaluated for affinity and effects on cell viability, and selected compounds also for effects on MYC protein levels in L363 cells (a MYC-driven multiple myeloma cell line^33^). Affinities were generated by measuring *K*_D_ values determined by the compound-induced change in fluorescence of an Alexa Fluor® 647 tag conjugated to the 5′ end of Pu27. Selected compounds are illustrated in Fig. 1b (Supplementary Table 1).

From this effort, clear trends were established to generate a structure–activity relationship and determine requirements for binding. Substitution of an electron withdrawing trifluoromethyl group in place of the methyl group on the aryl amide yielded increased affinity, decreased cell viability, and decreased MYC expression (DC-34, Fig. 1b). With DC-34, the most potent compound, MYC protein expression was reduced to 0.4% at a 10 μM dose compared to 50% for the methyl-substituted compound and an increase in potency was observed in the cell viability assay. Conversely, replacement of the methyl group with an electron donating methoxy substituent or substituted pyridine resulted in worse activity than the methyl-bearing compound for all assays, likely indicating a weaker interaction for the aromatic ring with the electron rich guanine tetrads. A fluoro substituent in place of the methyl group yielded a compound with minimal activity and weaker binding affinity (Supplementary Table 1).

The position of the trifluoromethyl group on the benzene ring was also evaluated next. The para-trifluoromethyl group emerged as the most potent, while the meta-analogs and ortho-analogs had weaker activity. Substitution of the aromatic ring in the ortho-position is likely to force the amide substituent out of plane with the arene, suggesting that these substituents are required to be co-planar for binding. Similarly, *N*-methylation of the amide also resulted in loss of activity and binding affinity by likely influencing the orientation of the amide bond, further highlighting a role for this group in the interaction with the *MYC* G4^34^.

Since the *para*-trifluoromethylbenzene emerged as the most effective substituent, we then investigated the amine ring of the 4-position on the benzofuran. Alteration of the azepane ring to other aliphatic amines did not result in dramatic increases in potency, while bicyclic heterocycles were uniformly inferior (Supplementary Table 1). Increasing the number of carbons between the benzofuran core and amine decreased activity, indicating that the benzylic amine substituent exhibited the best performance in all assays. The relative position of the cationic amine is thus clearly important for binding, though the nature of the aliphatic ring is somewhat less consequential. Variation of the methyl group on the 2-position of the benzofuran core with branched aliphatic groups resulted in considerably weaker affinity (Supplementary Table 1). Taken together, DC-34 displayed the best combination of affinity, ability to silence MYC, and limit cell growth. Therefore, this compound was the focus of further study.

### Biophysical analysis of DC-34 reflects *MYC* G4 preference

**Fig. 2:**
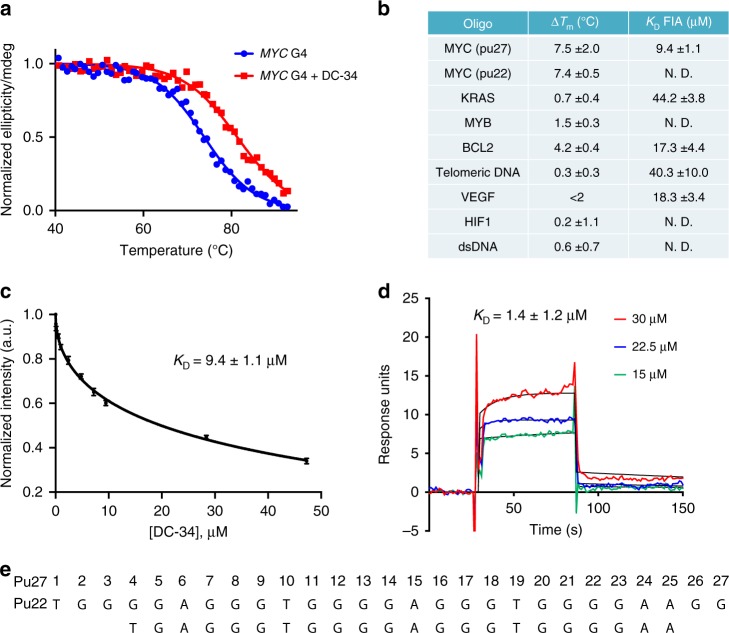
Biophysical analysis of the DC-34/*MYC* G4 interaction. **a** CD thermal melt of the *MYC* G4 Pu22 (10 µM) in the absence (blue) and presence (red) of DC-34 (40 µM). **b** Table of changes in thermal melting temperatures in the presence of four equivalents of DC-34 (measured by circular dichroism) and *K*_D_ values of DC-34 against a panel of G4-forming sequences and dsDNA. **c** Fluorescence intensity curves of 5′ Alexa Fluor labeled Pu22 MYC upon titration of DC-34. *K*_D_ = 9.4 ± 1.1 µM. **d** Sensorgrams corresponding to 15 to 30 µM injections of DC-34 over a 5′ biotin labeled MYC sensor chip. *K*_D_ = 1.4 ± 1.2 µM. **e** Sequence of *MYC* Pu 27 and Pu22

To evaluate the interaction between DC-34 and the *MYC* G4 in greater detail, we performed in-depth biophysical analyses. To test whether DC-34 confers stability to the *MYC* G4, we performed a circular dichroism (CD)-based thermal melt assay, in which molar ellipticity was measured as a function of increasing temperature^3^. In the presence of DC-34, the melting temperature of Pu27 and Pu22 increased by 7.5 ± 2.0 and 7.4 ± 0.5 °C, respectively (Fig. 2a for Pu22 and Supplementary Fig. 1 for Pu27). Increasing the length of loops 1 and 2 of Pu22 resulted in lower Δ*T*_m_ values with DC-34, while changing the length of the third loop had no effect (Supplementary Table 2). An A25T mutant G4 had a similar decrease in stability as removing both 5′ and 3′ tails, highlighting the importance of this residue for DC-34 binding (Supplementary Table 2). DC-34 exhibited a smaller Δ*T*_m_ of 4.2 ± 0.4 °C for the *BCL-2* G4^35^, and minimal change was observed with the G4 oligos from *VEGF*^36^, *KRAS*^37^, *MYB*^38^, and *HIF1α*^39^ genes (Fig. 2b). Similarly, no change in *T*_m_ was observed for two different antiparallel G4 structures including a G4 derived from telomeric DNA^40^ or dsDNA (Supplementary Table 2).

Surface plasmon resonance (SPR) experiments were used to further quantitate the binding of DC-34 to the *MYC* G4. DC-34 had a *K*_D_ of 1.4 ± 1.2 μM with a 5′-biotin labeled Pu27 (Fig. 2d). This was in reasonable agreement with the *K*_D_ of 9.4 ± 1.1 μM from the fluorescence intensity assay (FIA) with a 5′-Alexa Fluor ® labeled Pu27 (Fig. 2c). By FIA, DC-34 displayed a tighter binding affinity for *MYC* than several other G4 oligos, including DNA and RNA G4 oligos from the *VEGF*^41^, *KRAS*^37^, *BCL2*^35^, telomeric DNA^40^, and *NRAS*^42^ genes (Fig. 2b and Supplementary Fig. 2). Thus, unlike other G4-binding compounds such as CX-4561, DC-34 does not bind to B-DNA and has weaker binding to a variety of other quadruplex sequences^3^.

### DC-34 requires the G4 to downregulate *MYC* in cancer cells

**Fig. 3:**
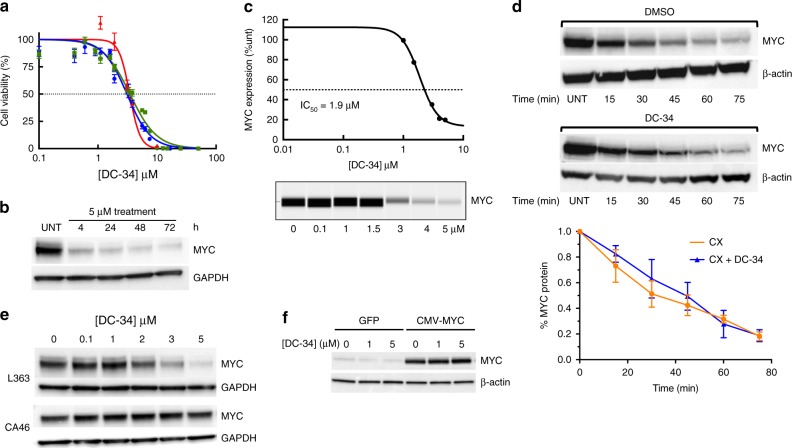
DC-34 silencs MYC expression in cancer cells. **a** Inhibition of L363 MM cell proliferation at 24 (IC_50_ = 3.4 µM) (red), 48 (IC_50_ = 3.4 µM) (green) and 72 h (IC_50_ = 3.1 µM) (blue). **b** Inhibition of MYC protein translation with 5 μM of DC-34 is sustained over time in L363 cells. **c** The half maximal inhibitory concentration (IC_50_) of DC-34 with respect to MYC protein inhibition as determined by Peggy protein expression. **d** A Western blot of MYC protein during a representative cycloheximide-chase degradation experiment with L363 multiple myeloma cells. Cells were treated with 10 µg/mL of cycloheximide (CX) in the absence or presence of DC-34 (5 µM) and MYC protein expression was assessed at indicated time points. β-actin was used as loading control. **e** MYC protein levels are inhibited as a function of the dose of DC-34 in L363 cells; only the highest dose of DC-34 affected MYC in the more resistant CA46 Burkitt’s lymphoma cells. **f** Western blot analysis of 293T cells transiently transfected with either GFP or CMV-MYC plasmid (the CMV promoter lacks a MYC G4) (right) and dosed with different concentrations of DC-34. All western blots were exposed for <1 min

We next performed time course experiments of cells treated with DC-34 to assess tumor cell viability and MYC protein levels (Fig. 3a–c). The decrease in cell viability (Fig. 3a) and MYC protein levels (Fig. 3b, c) in L363 multiple myeloma (MM) cells and other MM cells (Supplementary Table 3) was sustained over the entirety of the 72-h time course. The IC_50_ value for DC-34 in L363 MM cells was 3.4 μM at 24 h (Fig. 3a) while the IC_50_ value for MYC protein levels was 1.9 µM at 24 h (Fig. 3c). The effects of DC-34 in decreasing MYC expression were not due to changes in protein stability, as demonstrated by cycloheximide pulse-chase experiments; the stability of MYC was not altered in the presence of DC-34 up to a time of 75 min (Fig. 3d). To measure specificity, we tested DC-34 activity against the CA46 cell line, which lacks the MYC G4 promoter sequence due to a translocation placing *MYC* expression under the control of the IgH promoter^43^. In MYC G4(−) CA46 cells, DC-34 dosed up to 5 µM did not decrease MYC protein levels (Fig. 3e). Additionally, when human 293T cells were transfected with a plasmid expressing MYC from a CMV promoter (which lacks the G4), DC-34 had no effect on MYC protein levels (Fig. 3f) and little effect on cell viability (IC_50_ = 34 µM) (Supplementary Table 3). Although 293T cells express low levels of MYC protein, treatment with DC-34 did show a modest decrease (Fig. 3f) which was not seen in the transiently expressed MYC.

**Fig. 4:**
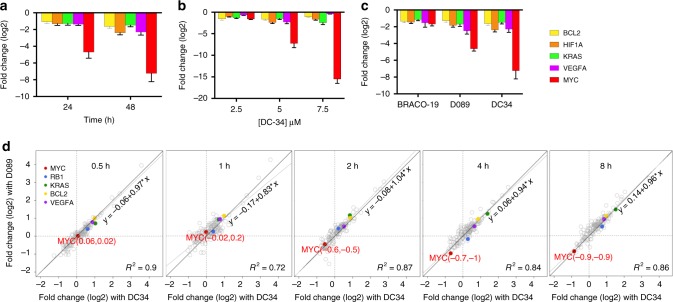
Effects of DC-34 on gene expression. **a** qPCR analysis of the indicated G4 containing genes following 5 μM treatment in L363 cells. **b** qPCR analysis of G4-associated genes at 48 h as a function of [DC-34]. **c** qPCR analysis of G4-associated genes after 48 h treatment with Braco-19, a pan-G4 binder, D089, a MYC G4 analog, and DC-34 at the indicated doses in L363 cells. Data in **a**–**c** are the average log_2_ values for ΔΔ*C*_t_ of three replicates in L363 cells. **d** Effects of D089 (15 µM) and DC-34 (5 µM) on expression of genes (Nanostring) at the indicated time points. G4 controlled genes including MYC (red) are highlighted in color. Fitted linear regression lines (solid) for gene expression and identity lines (dashed) for drugs are indicated

To confirm that preferential binding (Fig. 2b) results in a functional effect, we evaluated the impact of DC-34 on the expression of a panel of G4-driven oncogenes, including *BCL-2*^35,44^, *KRAS*^37^, *HIF-1α*^39^, *VEGFA*^36^, as well as *MYC* by quantitative PCR (qPCR). Treatment with DC-34 resulted in a dramatic decrease in *MYC* RNA in a time and dose dependent manner, and had weaker effects on the other genes (Fig. 4a–c). In comparison to BRACO-19^45^ (another pan-G4-binding molecule), and another *MYC* G4-stabilizing analog (D089), DC-34 had superior ability to silence *MYC* expression (Fig. 4c). Furthermore, protein levels of Rb1^41^ and Bcl2 remained unaffected up to 5 μM, a level above the IC_50_ value for L363 cells; both proteins showed modest decreases over a 48 h period with 10 µM DC-34 (Supplementary Fig. 3).

In order to more broadly analyze the changes in gene expression following drug treatment, we analyzed Nanostring (Cancer Panel) data from a time course (0.5 h, 1 h, 2 h, 4 h, 8 h) of treatment with either D089 (another *MYC* G4-stabilizing compound^30^) or DC-34. The gene expression responses were highly correlated, with *R*^2^ values ranging from 0.72 to 0.90 (Fig. 4d). A set of cancer-associated genes are highlighted in Fig. 4d (gray circles), illustrating that of the known G4-driven genes in this panel (colored circles), only MYC (red circles) decreases over the 8 h period.

We investigated cell cycle following DC-34 treatment in L363 cells by flow cytometry. We completed a time series of cell cycle analyses which demonstrated more cells accumulating in G0–G1 by 24–48 h following treatment with 5 µM DC-34 (Supplementary Fig. 4A, B). After 48 h of treatment with 5 µM DC-34, 63.54% of cells were arrested in G0/G1 vs. 49.37% of untreated cells. Consistent with growth arrest at G0–G1, we also noted p16 induction, a surrogate marker for senescence, following DC-34 treatment, especially at high concentrations (Supplementary Fig. 4C). Together, these data confirm that by binding preferentially to the *MYC* G4, DC-34 decreases both cell viability and *MYC* expression in L363 cells.

### DC-34 binds independently to the *MYC* G4 3′ and 5′ ends

**Fig. 5:**
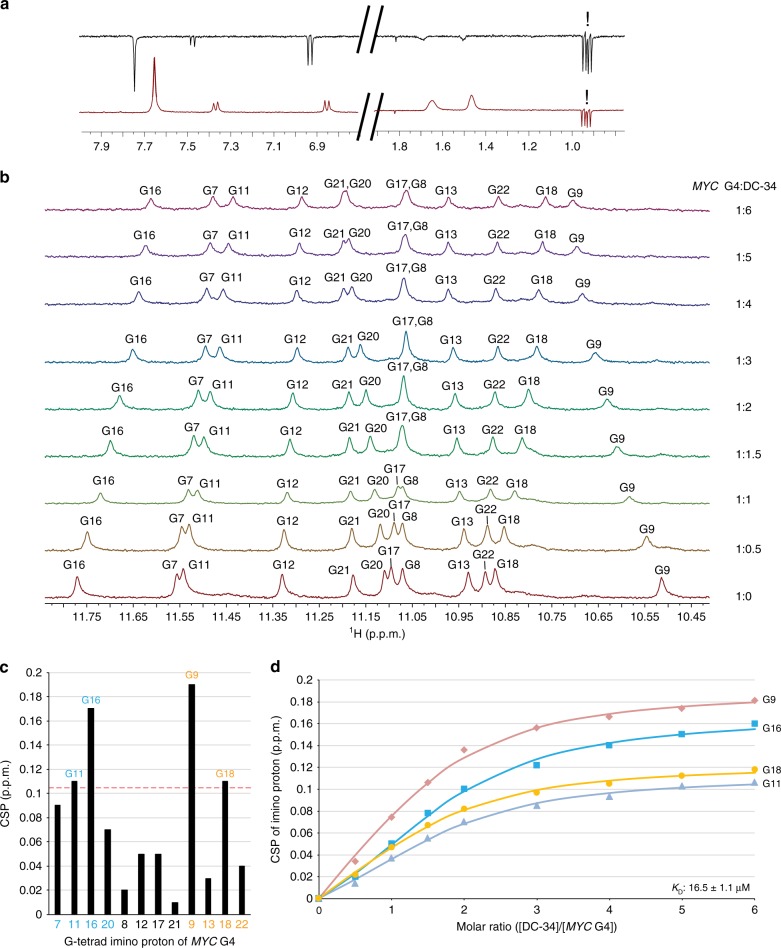
Shifting of imino protons during a titration experiment indicates DC-34 binding to each end of *MYC* G4. **a** WaterLOGSY NMR spectra of DC-34 and N-methyl-l-valine (non-binding control, peaks indicated with !) in the absence (top) and presence (bottom) of MYC G4. **b** Expanded 1D ^1^H NMR spectra illustrating the imino region during titration of DC-34 into *MYC* G4. Molar ratios of *MYC* G4:DC-34 are as indicated at 1:0, 1:0.5, 1:1, 1:1.5, 1:2, 1:3, 1:4, 1:5, and 1:6. The G-tetrad imino protons are labeled in each spectrum. **c** Plot of chemical shift perturbation (CSP) comparing *MYC* G4 G-tetrad imino protons alone and with sixfold molar excess DC-34. The red dashed line indicates one standard deviation above the average value. **d** CSP values for G9 (red), G11 (purple), G16 (blue), and G18 (orange) H1 plotted against molar ratio of DC-34 to *MYC* G4. An overall *K*_D_ value of 16.5 ± 1.1 µM was obtained by fitting the data to a non-cooperative binding mode with the software package Bindfit v0.5

We used NMR experiments to probe the structural origin of the interaction between DC-34 and the *MYC* Pu22 G14T/G23T mutant^25,26^. We performed WaterLOGSY^46,47^ experiments on DC-34 in the presence and absence of the *MYC* G4 with *N*-methyl-l-valine used as an internal, non-binding control molecule (Fig. 5a). In the absence of DNA, all peaks phased negatively in the WaterLOGSY experiment, confirming that DC-34 does not aggregate in buffer. Upon the addition of DNA, only the peaks for DC-34 became positively phased and chemical shift perturbations were observed, confirming a direct interaction between DC-34 and the G4.

We titrated DC-34 into a sample of the Pu22 G14T/G23T G4 to demonstrate chemical shift perturbation of the imino protons. Fast exchange chemical shift perturbations were observed, allowing all 12 guanine imino protons from the *MYC* G4 tetrad planes^25^ to be tracked upon addition of DC-34. The observation of a new set of 12 well-resolved imino proton peaks (Fig. 5b) indicate the formation of a well-defined DC-34/*MYC* G4 complex. Perturbations saturated above six molar equivalents of DC-34, indicating that the effect is governed by affinity as well as stoichiometry. The largest perturbations were observed for G9 and G18 (3′ face) and G11 and G16 (5′ face) (Fig. 5b). Minimal chemical shift perturbations were observed for the imino protons from G8 and G21, in the central guanine tetrad. Plots of the chemical shift changes observed for G9, G18, G11, and G16 imino protons at varying molar ratio of DC-34 to *MYC* G4 yielded an overall *K*_D_ value of 16.5 ± 1.1 μM, by fitting the data to a non-cooperative binding mode with the software package Bindfit^48^ (Fig. 5d). The Pu22 G14T/G23T sequence used in NMR studies differs slightly from the wild-type Pu27 used in affinity measurements by fluorescence (Fig. 2c), a potential source of the small difference in observed affinities. Altogether, these findings suggest that DC-34 likely stacks independently on the 3′ and 5′ faces of the *MYC* G4, with similar affinity for each site.

### Distinct binding of DC-34 at the 5′ and 3′ ends of *MYC* G4

**Fig. 6:**
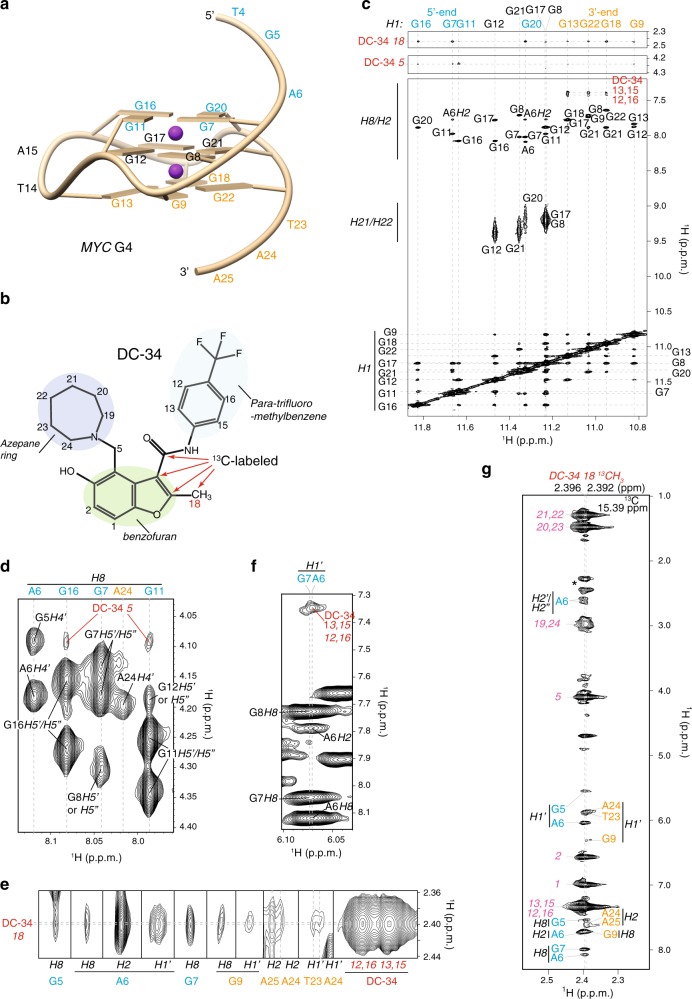
Intermolecular NOE interactions indicate distinct binding modes for DC-34 at the 5′ and 3′ end of *MYC* G4. **a** Representative G-quadruplex structure for the *MYC* G4 with nucleic acid number indicated. The lowest energy structure displayed in Fig. 7 was used to make this figure. **b** Annotation of DC-34 indicating numbering scheme and ^13^C-labeling. **c**–**f** Expanded regions of a 2D NOESY spectrum acquired on *MYC* G4 with twofold molar excess unlabeled DC-34 at pH 6.4 in buffer A (25 mM Tris-*d*_11_ and 50 mM KCl) with 90% H_2_O/10% DMSO-*d*_6_. H1–H1, H21/H22–H1, and H8/H2–H1 NOE interactions within *MYC* G4 are labeled (**c**, bottom panel), as are intermolecular NOE interactions between *MYC* G4 H1 and DC-34 methyl (**c**, top panel) or methanediyl (**c**, middle panel) groups. **g** Selected region from a ^1^H, ^13^C half-filtered NOESY experiment acquired with *MYC* G4 and twofold molar excess DC-34 with selective ^13^C-labeling as indicated by red arrows in **b**. Intermolecular NOE interactions between the DC-34 methyl group and *MYC* G4 protons are displayed and labeled as are intramolecular NOE interactions involving ^12^C bound DC-34 protons. A breakthrough intramolecular signal from the DC-34 methyl group is indicated by a black asterisk. Labeling of NOEs in **c**–**g** is color coded with DC-34 in red (intermolecular) or pink (intramolecular) and *MYC* G4 in black, light blue (for the 5′ G-tetrad and flanking residues), or orange (for the 3′ G-tetrad and flanking residues)

We assigned 92% of DC-34-bound *MYC* G4 protons; most of the unassigned protons are guanine and adenine NH_2_ groups that are not present in the spectra. For clarity, we provide a representative G-quadruplex structure for *MYC* G4 with nucleic acid numbers indicated (Fig. 6a) and the DC-34 chemical structure with our numbering scheme and ^13^C-labeling included (Fig. 6b). The base imino protons (H1) of the *MYC* G-tetrads were assigned by tracking in the 1D titration spectra (Fig. 5b), validated by inter-residue NOE interactions (Fig. 6c, bottom panel), and used to assign the G-tetrad base aromatic protons (H8) (Fig. 6c, bottom panel). The ribose protons (H1′, H2′, H2”, H3′, H4′, H5′, and H5”) were assigned by NOE interactions (Fig. 6d and Supplementary Fig. 5A) and TOCSY spectra (Supplementary Fig. 5B). Our NMR data indicated a single G-quadruplex conformation for DC-34-bound *MYC* G4, with each proton exhibiting a single chemical shift value and NOEs characteristic of the three G-tetrad stacked structure (Fig. 6c).

The regions flanking the G-quadruplex were similarly assigned by NOE interactions with neighboring residues (Fig. 6c, bottom panel, and Fig. 6d) and intra-residue NOEs (Fig. 6d). Involvement of these bases in DC-34 binding was suggested by comparing their chemical shift values in the free and complexed state, as demonstrated for G22, T4, and T23 (Supplementary Fig. 5C). Loop residues T10, T14, A15, and T19 shifted only slightly following DC-34 addition (Supplementary Fig. 5D, demonstrated for A15), suggesting that they are not at the binding surface.

The protons of DC-34 in the complexed state were readily assigned and intermolecular NOEs confirmed direct interaction with flanking residues and 5′ and 3′ G-tetrads (Fig. 6c–e). Intermolecular NOEs were not observed to the central G-tetrad or loop residues. G7, G11, G16, and G20 H1 of the 5′ G-tetrad interact with DC-34 methyl and methanediyl groups (Fig. 6c, top and middle panels respectively); G7 H8 interacts with the DC-34 methyl group (Fig. 6e); and G11 and G16 H8 form NOEs with the DC-34 methanediyl group (Fig. 6d). The G7 and A6 ribose H1′ protons are overlapped but show NOE interactions with the DC-34 *para*-trifluoromethylbenzene protons (Fig. 6f). The DC-34 methyl group also shows NOEs to A6 H2, H8, and H1′, as well as G5 H8 (Fig. 6e). These interactions define a distinct orientation for DC-34 at the 5′ end.

At the 3′ end, intermolecular NOE interactions are detected between G9, G13, G18, and G22 H1 and DC-34 methyl, methanediyl, and/or *para*-trifluoromethylbenzene protons (Fig. 6c). G9 H8 and H1′, A24 and A25 H2, and T23 and A24 H1′ interact with the DC-34 methyl group (Fig. 6e). In contrast to the 5′ end, NOEs were observed from DC-34 trifluoromethylbenzene to G13, G18, and G22 H1 (Fig. 6c, bottom panel) as well as G18 and G22 H8 (Supplementary Fig. 5E). However, only G11 H8 of the 5′ end G-tetrad base region exhibited NOEs to the DC-34 para-trifluoromethylbenzene group (Supplementary Fig. 5E), although NOEs were also observed to the H1′ of overlapped A6 and G7 (Fig. 6f). Collectively, these NOEs place the DC-34 para-trifluoromethylbenzene group closer to the flanking and first residue of the 5′ G-tetrad, and more centered over the G-tetrad base region at the 3′ end.

To confirm the intermolecular NOE interactions, we synthesized tetra-^13^C-labeled DC-34, including a ^13^C-labeled methyl group (Fig. 6b). This probe was synthesized using a route similar to Fig. 1a but with ^13^C-labeled reagents. Twofold molar excess of tetra-^13^C-labeled DC-34 was then mixed with unlabeled Pu22 DNA. A ^1^H, ^13^C half-filtered NOESY experiment was acquired to record interactions between the DC-34 methyl group and *MYC* G4 (Fig. 6g). Intermolecular NOE interactions between the DC-34 methyl group and *MYC* G4 5′ face residues (G5, A6, G7) or 3′ face residues (G9, T23, A24, A25) were observed (Fig. 6g), as well as intramolecular NOE interactions involving ^12^C bound DC-34 protons. The observed intermolecular NOE interactions recorded using the ^13^C-labeled sample were consistent with those observed with unlabeled DC-34/*MYC* G4 complex (Fig. 6c–f). No NOE interaction was observed between DC-34 and residues of the central G-tetrad or 1:2:1 loop region. Thus, the NOESY experiments demonstrate the presence of two DC-34 molecules bound to the *MYC* G4, one at the 5′ end and the other at the 3′ end. Unique NOE interactions at each of these ends further demonstrate distinct binding modes.

### Structure of the DC-34/*MYC* G4 complex

**Fig. 7:**
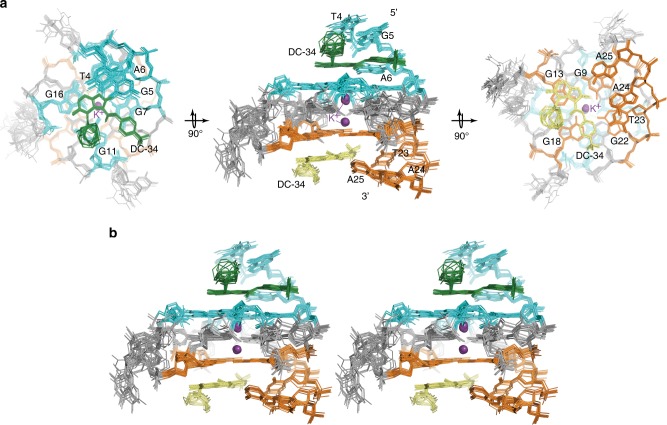
Structure of the DC-34*/MYC* G4 complex indicates an additional stacked layer at each end and rearrangement of the flanking residues. **a** The 15 lowest energy structures of the DC-34*/MYC* G4 complex are displayed with a top (left panel), side (middle panel), and bottom (right panel) view relative to the cylindrical axis of the DNA. Residues of *MYC* G4 from the 5′ G-tetrad and flanking residues are highlighted in light blue, whereas those from the 3′ end are indicated in orange. The two DC-34 molecules are colored green and yellow, and the two potassium ions displayed as purple spheres. **b** Stereo view of the 15 lowest energy structures of the DC-34*/MYC* G4 complex

We solved the structure of DC-34 complexed with the *MYC* G4 using NOE-derived distance constraints, hydrogen bonds, electrostatic bonds, and dihedral angle constraints, as described in Experimental Procedures and summarized in Table 1. The 15 lowest energy structures converged with an overall root-mean-square-deviation (r.m.s.d.) for all atoms including hydrogen of 0.89 ± 0.26 Å (Table 1) and higher convergence was found for the three G-tetrad structures alone (r.m.s.d. = 0.55 ± 0.12 Å), or the adjacent 5′- (T4-G5-A6) and 3′- (T23-A24-A25) flanking regions (Table 1). The superposition for the three G-tetrads is displayed for three different orientations (Fig. 7a, b) to highlight the *MYC* G4 parallel-stranded G-quadruplex with two bound potassium ions and an additional stacked plane contributed by DC-34 over each external G-tetrad of the *MYC* G4. The DC-34 benzofuran and para-trifluoromethylbenzene rings form pi–pi stacking interactions with the G-tetrads, from which the azepane ring is directed away (Fig. 7a, middle panel). The overall fold for the G-tetrad region is similar to the unbound state^25^, although differences exist in the flanking regions to accommodate DC-34.

**Table 1:** Structural and NMR statistics for the DC-34/*MYC* G4 complex

	DC-34/*MYC* G4
*NMR distance and dihedral constraints*	
Distance constraints	
Intramolecular NOEs	907
Intra-residue	610
Inter-residue	297
Sequential (|*i* – *j*| = 1)	253
Medium range (|*i* – *j*| ≤ 4)	22
Long range (|*i* – *j*| ≥ 5)	22
Hydrogen bonds	27
Intermolecular NOEs	45
Dihedral angle restraints	
*χ* (O4′-C1′-N9-C4)	12
G-tetrad planarity (5 atoms sub-groups)	24
Coordination bond (O6-K^+^)	16
*Structure statistics*	
Violations (mean and s.d.)	
Distance constraints (Å)	0
Dihedral angle constraints (°)	0
Max. dihedral angle violation (°)	0
Max. distance constraint violation (Å)	0
Deviations from idealized geometry	
Bond lengths (Å)	0.005 ± 0.000
Bond angles (°)	0.705 ± 0.005
Impropers (°)	0.461 ± 0.031
Average pairwise r.m.s.d (Å)^a^	
All atoms	0.89 ± 0.26
G-tetrads	0.55 ± 0.12
5′-end (T4-G5-A6)	0.29 ± 0.10
3′-end (T23-A24-A25)	0.29 ± 0.08

^a^Pairwise r.m.s.d. was calculated among 15 refined structures

**Fig. 8:**
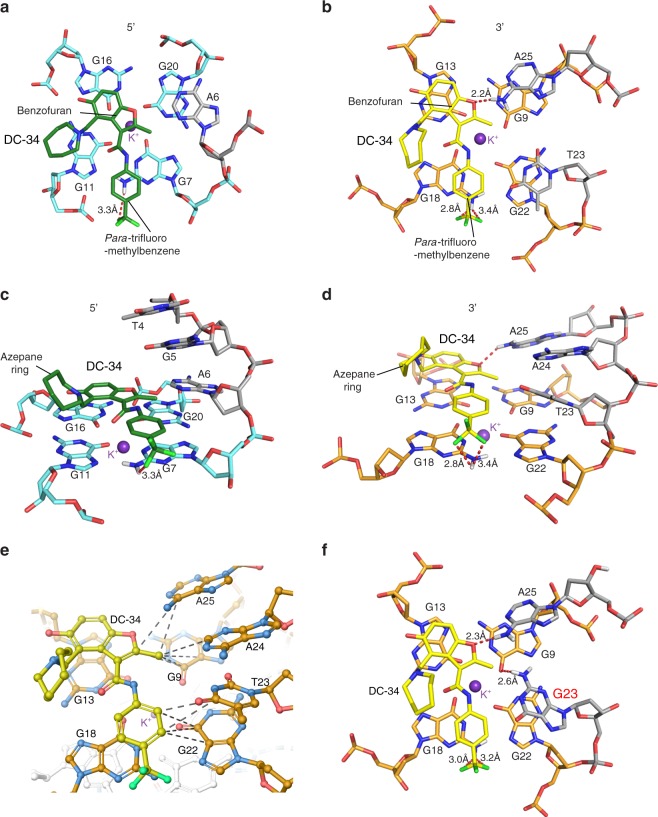
Specific contacts formed at each end of the *MYC* G4 provide a rationale for DC-34 as the preferred compound from the benzofuran-containing molecules screened. **a**–**d** Expanded views of the DC-34*/MYC* G4 complex to display interactions at the 5′ (**a**, **c**) and 3′ (**b**, **d**, **e**) ends of the *MYC* G4. The color scheme follows Fig. 7 but with flanking residues gray and oxygen, nitrogen and fluorine in red, navy, and light green. Hydrogen bonds between *MYC* G4 and DC-34 are indicated by dotted red lines. In **e** hydrophobic contacts to flanking residues are indicated by gray-dashed lines. **f** Expanded view of an energy minimized model structure of DC-34*/MYC* G4 with wild-type guanine substituted for T23

When unbound, A6 stacks over G11 with T4 and G5 directed away from the 5′ G-tetrad^25^ (Supplementary Fig. 6A). In the *MYC* G4 complex, A6 is displaced to form pi–pi stacking interactions with G20 while the DC-34 benzofuran and para-trifluoromethylbenzene rings stacks over G16 and G7, which are diagonal in the G-tetrad (Fig. 8a). This configuration enables the DC-34 benzofuran ring to interact with A6. DC-34 at the 3′ end stacks over G13 and G18, which are G-tetrad neighbors, while T23 packs against G22 (Fig. 8b); this configuration requires displacement of A25, which in the free state, forms pi–pi stacking interactions with G13^25^ (Supplementary Fig. 6B). At each end, distances and angles between DC-34 fluorines and NH_2_ groups from the guanines involved in G-tetrads are within the range of bonding interactions^49–52^. At the 5′ end, G7 is 3.3 Å from a fluorine on DC-34 with an angle of 140° (Fig. 8a, c). At the 3′ end, DC-34 is a bifurcated donor with G18, at distances of 2.8 Å and 3.0 Å and angles of 101° and 98°, respectively (Fig. 8b, d). Additionally, on the 3′ face, the benzylic amine carbon of DC-34 is 3.5 Å away from G13, indicating a cation–pi interaction^52,53^. The 5′ flanking residues T4 and G5 stack against each other (Fig. 8c), placing T4 far from DC-34. By contrast, A25 does not stack over A24 (Fig. 7a, middle panel) and instead adopts a configuration that enables a hydrogen bond between its NH_2_ group and the DC-34 benzofuran oxygen (Fig. 8b). A25, together with A24, also forms hydrophobic interactions with the benzofuran group of DC-34 (Fig. 8e), as supported by NOEs involving the methyl group (Fig. 6e–g).

## Discussion

Here, we demonstrate a molecular basis for specific recognition of the *MYC* G4 structure with a drug-like small molecule. Insights are provided by chemical approaches, e.g., alteration of the chemical structure of the ligand, as well as biochemical and biophysical experiments. Analysis of a focused library of analogs has revealed a direct structure–activity relationship. Through this work we identified trifluoromethyl-substituted compound DC-34 as the analog with the best affinity and ability to decrease *MYC* expression. The *K*_D_ measurements from SPR demonstrated a roughly sevenfold tighter binding interaction than FIA. This apparent difference in affinity may be due to the use of different labels on the oligonucleotide or the different biophysical techniques used to measure affinity. Furthermore, FIA experiments use a two-site binding model while SPR experiments only measure the higher affinity binding site. However, consistent with the low micromolar affinity, exposure of cells to low micromolar concentrations of DC-34 caused decreases in MYC protein levels, indicating that it is likely saturated at working concentrations. This effect was confirmed to occur at the transcriptional level. In 293T cells transfected with a plasmid expressing *MYC* from the CMV promoter (which lacks the *MYC* G4), DC-34 had no effect on MYC levels. In CA46 cells, which harbor a chromosomal translocation resulting in one *MYC* allele being driven by the IgH promoter (lacking the *MYC* G4)^43^, DC-34 had minimal effects on MYC protein levels at IC_50_ doses required to limit myeloma growth. In biophysical experiments, DC-34 preferentially stabilized the *MYC* G4 over six other known G4s and had no effect on the *T*_m_ of dsDNA. At doses of 5 µM and 7.5 µM, DC-34 exhibited a pronounced ability to decrease *MYC* mRNA, while having minor effects on other G4-driven genes.

To aid in solving the structure defined at the atomic level, we generated a ^13^C-labeled DC-34 sample for use in a ^13^C half-filtered NOESY experiment. Intermolecular NOEs observed with both this experiment and conventional NOESY experiments acquired with unlabeled DC-34 yielded a structure with distinct binding of DC-34 to each end of the *MYC* G4 with pi–pi stacking interactions between the benzofuran and methylbenzene rings of DC-34 and the terminal G-tetrads, as has been observed for other molecules^22^. Hydrogen bonding no doubt contributes to the specificity observed in the biophysical and biological assays. The oxygen in the benzofuran core forms a hydrogen bond to NH_2_ group of A25 and fluorines at the para position of the benzene ring forms hydrogen bonds with G7 and G18 NH_2_ groups; these interactions are not observed with other reported ligands that generally stack between flanking residues on the tail^26,54^. Changes to either of these functional groups decreased the affinity and activity of the corresponding analog. The carbon of the benzylic amine forms a cation–pi interaction with G13 on the 3′ face. Increasing the number of carbons between the benzofuran core and the amine ring alters the location of the positive charge relative to the tetrads, decreasing activity. Additionally, in order to accommodate ligand binding, both the 5′ and 3′ tails move away from the tetrads to generate hydrophobic-binding pockets. To stabilize the *MYC* G4 1:2:1 isomer we used a previously reported Pu22 DNA sequence with G14 and G23 substituted with thymine^24,25^. Whereas T14 showed no interaction with DC-34, T23 of the 3′ flanking region interacts with the trifluoromethylbenzene group. We therefore substituted T23 with the wild-type guanine in the *MYC* G4/DC-34 structure and performed energy minimization. The bulkier G23 purine base was readily accommodated in the DC-34 binding pocket with the NH_2_ group positioned to form a potential hydrogen bond with O_6_ from G9 (Fig. 8f); this possibility may further explain the stronger affinity measured for the Pu22 wild-type sequence. In addition, the wild-type *MYC* G4 is known to exist in multiple equilibrating conformations. However, we see comparable affinity for both wild-type (Pu22, Pu27) and mutant (Pu22 G14T/G23T) quadruplexes, suggesting that the major conformation in solution is recognized by DC-34.

Several G4/ligand structures are available^3,4^ and reveal a common stacking of ligand on one end of the G4 to form a 1:1 complex; as exemplified by the telomeric G4/MM41 and G4/L2H2-6M(2)OTD complexes^55,56^ (Supplementary Fig. 7A and 7B) and *MYC* Pu24 G4 complexes with TMPyP4 and Phen DC_3_^57,58^ (Supplementary Fig. 7C and 7D). By contrast, DC-34 molecules bound to the *MYC* G4 5′ and 3′ external tetrads forming a 2:1 complex, similar to a previously characterized *MYC* G4 ligated quindoline derivative complex^26,59^ (Supplementary Fig. 7E). All G4/ligand complexes were stabilized by pi–pi interactions. TMPyP4 and Phen DC_3_ overlap extensively with all four guanines of the 5′ G-tetrad (Supplementary Fig. 7C and 7D)^57,58^ whereas DC-34 and the quindoline derivative stack over only two guanines at each terminal G-tetrad plane with flanking segments reconfigured to cap the ligand^26^ (Fig. 8 and Supplementary Fig. 7E). At the 3′ end, these two ligands stack over G13 and G18 and form a hydrogen bond to a 3′ flanking residue; namely, DC-34 benzofuran oxygen and quindoline derivative N1 form hydrogen bonds to A25 NH_2_ (Fig. 8b) and T23 O4 (Supplementary Fig. 7E), respectively. A greater difference is observed at the 5′ end where DC-34 stacks over G7 and G16 while the quindoline derivate overlaps with G11 and G16 (Fig. 8a and Supplementary Fig. 7E). In addition to the pi–pi interactions, positively charged atoms present in the various other G4-binding ligands were observed to contribute electrostatic interactions with negatively charged G4 phosphates^55–57^. In the quindoline derivative, the protonated aminoalkylamino side chain is within 3 Å of the G16 phosphate group (red circle in Supplementary Fig. 7E) whereas the DC-34 azepane ring is too short for such an interaction. By contrast, bonding interactions are formed between the DC-34 fluorines and G7 or G18, and a cation–pi interaction forms between the DC-34 benzylic amine carbon and G13 (Fig. 8).

Although G4-binding ligands stack on a common tetrad structure, variations in groove dimensions, the inter-G-tract loops, and flanking segments allow for specificity^4,26,55,60^. In the *MYC* G4, each end has flanking regions that contribute favorably to DC-34 binding as mentioned above, with mutations in these regions reducing affinity (Supplementary Table 2). Moreover, neither DC-34-binding site in the *MYC* G4 is obscured by residues from the loop regions, whereas other G4s that we examined have features that make the DC-34 binding sites less accessible. We generated a model of a *KRAS* G4/DC-34 structure using the *KRAS* G4 structure (PDB 5I2V)^61^ with DC-34 placed in the analogous region compared to its *MYC*-binding sites. At the 5′ end, the *KRAS* G4 contains a four-base loop (A14-A15-T16-A17) while the *MYC* G4 has a one base loop (T19) (Supplementary Fig. 8A and 8B). This loop narrows the cavity in the *KRAS G4*, inducing steric clashes with DC-34 (Supplementary Fig. 8B). The *KRAS* 3′ end is less crowded; however, the additional loop nucleotide T8 inserted between G7 and G9 of stacked G-tetrads causes the G9 phosphate group to be ~4 Å from the DC-34 hydroxyl group (circled in Supplementary Fig. 8C). The steric clashes and unfavorable electrostatic interactions observed between DC-34 and *KRAS* G4 in the model supports the lack of binding and effect on gene expression measured experimentally. Similarly, in the *BCL2* G4 (PDB 2F8U), two lateral loops are located at both the 5′ and 3′ faces, a well-defined three nucleotide loop at the 3′-end and a seven-nucleotide loop that caps the 5′ G-tetrad to form potential reversed Watson–Crick hydrogen bonds (Supplementary Fig. 9)^62^. These lateral loops are likely to be more restricted than those of *MYC* G4, which we observed to be reconfigured upon DC-34 binding. The *MYC* G4 contains short loops (1-nucleotide or 2-nucleotide) and flexible flanking regions, which collectively make it more accessible for DC-34 binding.

A variety of compounds have been reported to bind to the *MYC* G4; however, these compounds generally do not have drug-like properties and are not selective for the *MYC* G4 over other G4 structures^24,27^ or other protein targets^63^, despite often exhibiting good anticancer effects. Our work demonstrates that drug-like compounds can discriminate between different quadruplexes by making discrete interactions or altering the conformation of the tail, loop, and tetrad portions of the quadruplex. These conformational changes and bonding interactions provide a plausible basis for G4 recognition and selectivity in controlling gene expression in cells. Here, we provide a structural basis for one such recognition event by solving an NMR structure of DC-34 bound to the *MYC* G4. Additionally, we demonstrate through chemical and functional studies that affinity for the target correlates directly with cellular activity and ability to regulate gene expression. Thus, further structure-based investigation of G4-binding compounds is likely to be fruitful in developing high affinity and selective probes of gene expression, as well as potential therapeutics.

## Methods

### Thermal melt assays

The thermal stability of the *MYC* oligonucleotide (TGA GGG TGG GGA GGG TGG GGA A) or other appropriate oligonucleotide with and without compound was determined using an Aviv Biomedical Model 420 Circular Dichroism (CD) Spectrometer equipped with a ThermoCube temperature regulator. To anneal the oligonucleotide, the sample was heated to 95 °C for three minutes and allowed to cool to RT over 1–2 h. The oligonucleotide was then diluted to 10 µM in 10 mM Tris buffer (pH 6.3, containing 30 mM KCl) and four equiv of compound were added to yield 40 µM compound in 1% DMSO. Spectra were recorded from 224 to 312 nm at 25 °C with a step size of 2 nm, followed by heating from 25 to 97 °C at 1 °C/min in a 0.1 cm quartz cuvette. To calculate the *T*_m_ of each sample, ellipticity was plotted as a function of temperature and fit in GraphPad Prism 7 software using a nonlinear sigmoidal dose-response model with a variable slope. Each condition was performed in triplicate, with Δ*T*_m_ values calculated using *T*_m(+compound)_ – *T*_m(apo)_ and then averaged to yield the final value.

### Fluorescence intensity titration

Alexa Fluor 647-labeled *MYC* (pu27): (TGG GGA GGG TGG GGA GGG TGG GGA AGG) or other appropriate oligonucleotide was heated at 95 °C for three minutes, allowed to cool to RT, and diluted to 50 nM in 25 mM Tris buffer (pH 6.4, containing 50 mM KCl). Compound was added as a solution both in buffer containing 2–3% DMSO, and the sample was allowed to equilibrate for 10 min. Fluorescence intensity spectra were recorded at RT using a Photon Technology International, Inc. QuantaMaster 600^TM^ Spectrofluorometer equipped with Felix GX 4.2.2 software. Fluorescence intensity was recorded at an excitation wavelength of 645 nm, with the resulting emission spectrum recorded from 650 to 800 nm, and the fluorescence intensity at the emission maximum was used in all calculations. *K*_D_ values were fit using a 2:1 binding model.

### Surface plasmon resonance

SPR was conducted using a Biacore 3000 (Biacore, Inc) instrument. Streptavidin (Rockland) was immobilized to 20000 RU in both flow cells using EDC/NHS coupling to a CM5 chip (GE). The surface was then blocked with ethanolamine. Next, 1 μM *MYC* (pu27) 5′-biotin-TEG BiotinTEG TGG GGA GGG TGG GGA GGG TGG GGA AGG (obtained as an HPLC-purified sample from Integrated DNA Technologies, Inc.) was refolded in 10 mM TRIS, 30 mM KCl, 3% DMSO, pH 6.4 by heating at 98 °C in a heater block for 2 min then cooled to room temperature, and immobilized on one flow cell of the SPR chip to a density of 450 RU. Compounds were injected at a flow rate of 30 mL/min in 10 mM TRIS, 30 mM KCl, 3% DMSO, 0.01% Tween-20, pH 6.4 for 1 min. Each injection was repeated twice for consistency. Each trace was fit individually to a Langmuir model (1:1 binding, reporting on the highest affinity binding site) for DC-34.

### Cell culture methods

Human multiple myeloma cell lines L363, KMS12PE, JIM1, AMO1, KMM1, KMS27, ARD, OPM1, KHM11, KARPAS417, and H929 were cultured in Advanced RPMI 1640 (6% heat-inactivated fetal bovine serum (FBS): Gibco by Life Technologies, 2 mM l-glutamine: Gibco by Life Technologies, 100 U/mL Penicillin and 100 μg/mL Streptomycin: Gibco by Life Technologies, 100 µg/mL Normocin: InvivoGen) and incubated at 37 °C with 5% CO_2_. Human Burkitt′s lymphoma cell line CA46 was cultured in RPMI 1640 (10% heat-inactivated fetal bovine serum (FBS): Gibco by Life Technologies, 2 mM l-glutamine: Gibco by Life Technologies, 100 U/mL Penicillin and 100 μg/mL Streptomycin: Gibco by Life Technologies, 100 µg/mL Normocin: InvivoGen) and incubated at 37 °C with 5% CO_2_. Media was changed every 2 days. For cells plated and harvested for protein or RNA, pellets were washed twice with cold PBS. After aspirating off the PBS, pellets were flash frozen in dry ice and transferred to a −80 °C freezer for short term storage. Cell lines were obtained from Michael Kuehl (NCI) and tested by CNV fingerprinting to verify their authenticity^64^.

### Cell viability (mts) experiments

Cell viability experiments were performed using CellTiter 96 AQueous One Solution Cell Proliferation Assay System (Promega). Cells were plated in quadruplicate on clear, flat-bottomed 96-well tissue cultured treated plates (Corning Costar) and incubated for each designated time in a 37 °C incubator with 5% CO_2_. Concentrated drug stocks were diluted down in Eppendorf tubes to each specific dose point before being added to the plate. After incubation, MTS reagent was added directly to the wells and incubated again at 37 °C with 5% CO_2_ for 90 min. The absorbance of the MTS formazan was immediately read at 500 nm on an Omega 640 spectrophotometer. A blank measurement was taken from the absorbance of the wells with media only and subtracted accordingly. Percentage cell viability was normalized to the absorbance of untreated wells after blanking and averaged from the four quadruplicate wells.

### Protein assays and western blotting

Cell pellets were first lysed in RIPA buffer (RIPA, sodium orthovanadate, PMSF, protease inhibitor, and phosphatase inhibitors A and B), vortexed to homogenize, and sonicated in an ice water bath for 5 min on high, with intervals of 30 s on, 1 min off. Homogenized pellets were then incubated on ice for 90 min before being spun down and transferred to new Eppendorf tubes. Protein was next quantitated by a standard BCA protocol. 15 µg of protein was loaded into each well of 4–12% Bis-Tris Gels (Novex), electrophoresed at 135 V for 90 min to achieve optimal band separation, and transferred with the iBlot2 system (Life Technologies). Equal protein loading and transfer was confirmed by Ponceau staining (Thermo Scientific) after the initial protein transfer. Western blots were blocked in 5% dry milk in 1XTBST (10X TBS, DI H_2_O, Tween 20) for 1 h, washed three times in 1XTBST for 10 min each, and incubated on a rocker in a 4 °C cold room overnight with primary monoclonal antibodies in 5% BSA at the designated dilution by the manufacturer. Blots were washed three times with 1XTBST before incubation with the appropriate species and dilution of polyclonal secondary antibodies in 5% dry milk on a rocker at room temperature for 1 h. Blots were again washed three times with 1XTBST before imaging with SuperSignal West Dura Chemiluminescent Substrate (Thermo Scientific) on a GBOX F3 Imager (Syngene). Primary antibodies were purchased and used as follows: c-myc (abcam ab32072, rabbit, 1:10,000), Rb1 (Cell Signaling 9309, mouse, 1:2000), Bcl2 (Cell Signaling 2870 S, rabbit, 1:1000), GAPDH (abcam ab128915, rabbit, 1:25,000), and Vinculin (Cell Signaling 13901 P, rabbit, 1:1000). Secondary antibodies were purchased and used as follows: HRP-linked Anti-rabbit IgG (Cell Signaling 7074, 1:2500), and HRP-linked Anti-mouse IgG (Cell Signaling 7076, 1:2500). For a higher throughput quantitation of MYC protein from treated cells, a size-based automated capillary immunoassay system (Peggy Sue Simple Western, ProteinSimple, Santa Clara, CA) was used by the Center for Cancer Research Collaborative Protein Technology Resource Group and operated according to manufacturer’s protocols. Uncropped western blots are shown in Supplementary Figs. 82–97.

For more precise quantification of the MYC protein in determining the half maximal inhibitory concentration (IC_50_) due to drug treatment, the size-based automated capillary immunoassay system (Simple Western, ProteinSimple, Santa Clara, CA) was performed by the Center for Cancer Research Collaborative Protein Technology Resource group according to the manufacturer’s protocol. A more comprehensive description of the experimental procedures have been described previously^65^.

### Quantitative PCR

RNA was first isolated from cells using manufacturer’s protocols from the Qiagen RNeasy Mini Kit with the addition of QIAshredder columns. RNA concentration was evaluated using a NanoDrop ND-1000 Spectrophotometer. cDNA was next reverse transcribed using a master mix of MultiScribeTM reverse transcriptase (1 U/µL, Applied Biosystems), 1X RT buffer (Applied Biosystems), dNTP mix (2 mM, Applied Biosystems), MgCl_2_ (5.5 mM, Applied Biosystems), RNAse inhibitor (0.4 U/µL, Applied Biosystems), and 1X RT random primers (Applied Biosystems). Master mix was added to 1 µg of RNA and thermal cycled at 25 °C for 10 min, 48 °C for 60 min, 95 °C for 5 min, and finally held at 4 °C until use. cDNA was diluted 1:10 with ultrapure, RNAse free water before use in qPCR. Samples for qPCR were prepared with 1:4 between diluted cDNA and primer master mix (1X SYBR Green PCR Mix: Applied Biosystems, 0.2 µM forward primer, 0.2 µM reverse primer, ultrapure H_2_O). qPCR was performed on an Applied Biosystems 7500 Fast Real-Time PCR System per manufacturer’s protocol. All primers were validated to ensure the absence of primer-dimers, the presence of a single peak dissociation curve, and an acceptable standard curve from serial dilutions of the same cDNA.

### Cycloheximide-chase degradation assay

L363 multiple myeloma cells were grown at 37 °C in Gibco RPMI Advanced Media (6% FBS) and plated at ~1.0 × 10^6^ cells per ml density. Cycloheximide was added to a final concentration of 10 µg/mL and DC-34 was added to a final concentration of 5 µM. An equivalent amount of DMSO was added for control samples. Cell samples were obtained every 15 min at indicated time points by centrifugation and washed once with PBS prior to flash freezing. Whole-cell protein extracts were prepared using the RIPA lysis buffer (ThermoFisher #89900) for 1 h with intermittent vortexing. Proteins were separated by SDS-PAGE and analyzed by immunoblotting with c-myc (ab32072) and β-actin (cell signaling #4967) primary antibodies. A secondary goat anti-rabbit antibody (IgG (H + L)-HRP, Invitrogen G21234) was used for detection by the Chemidoc^TM^ Touch Imaging System. Three replicates of the assay were performed and signal intensity was normalized to β-actin and averaged, with error bars representing standard deviation.

### Water ligand observed gradient spectroscopy (waterLOGSY)

A reference 1D-1H and 1D WaterLOGSY spectrum of 100 µM *N*-methyl-l-valine (Chem-Impex-International) and 100 µM compound was collected, followed by a separate sample containing 5 µM *MYC*, 100 µM *N*-methyl-l-valine, and 100 µM compound. *MYC* oligonucleotide (TGA GGG TGG GGA GGG TGG GGA A) was buffer exchanged into 10 mM Tris-*d*_11_ buffer (pH 6.4, containing 50 mM KCl) using centrifugal filtration (3 kDa MWCO, EMD Millipore). A sample of DC-34 and *N*-methyl-l-valine, each at 100 µM, was prepared in 25 mM Tris-*d*_11_ buffer (pH 6.4, containing 50 mM KCl and 10% DMSO-*d*_6_), and 1D reference proton and WaterLOGSY spectra without oligonucleotide were recorded. These spectra were recorded at 20 °C on a Bruker AVANCE III 500 MHz spectrometer equipped with TCI cryogenically cooled probe. The “zgesgp” excitation sculpting water suppression pulse sequence from Bruker was used for data acquisition with 128 scans. All data were processed and visualized with MestReNova software (Version 8.1.2–11880).

### NMR spectroscopic experiments of *MYC* G4/DC-34

NMR samples of *MYC* G4, DC-34, or the *MYC* G4/DC-34 mixtures (at equimolar or twofold molar excess DC-34) were at pH 6.4 in buffer A (25 mM Tris-*d*_11_ and 50 mM KCl) with 90% H_2_O/10% D_2_O, 90% H_2_O/10% DMSO-*d*_6_, or 90% D_2_O/10% DMSO-*d*_6_ as indicated. DC-34 was first dissolved in DMSO-*d*_6_ to a 10 mM stock concentration, and a shortened *MYC* oligonucleotide (TGA GGG TGG GTA GGG TGG GTA A) was buffer exchanged (3 kDa MWCO spin column) into buffer A. With the exception of the WaterLOGSY experiments, all NMR data were acquired at 25 °C on Bruker Avance 700, 800, or 850 MHz spectrometers equipped with cryogenically cooled probes. 1D ^1^H and 2D homonuclear ^1^H–^1^H experiments including NOESY (80, 150, 300, and 400 ms mixing time), TOCSY (80 ms mixing time) and COSY were collected for *MYC* G4 with DC-34 at twofold molar excess and using relaxation delays of 2 s. A 2D NOESY (300 ms mixing time) experiment was also collected for *MYC* G4 with equimolar DC-34. Intermolecular NOE interactions between *MYC* G4 and the DC-34 methyl group were confirmed by using a ^13^C-half-filtered NOESY spectrum (300 ms mixing time) recorded on unlabeled *MYC* G4 mixed with twofold molar excess DC-34 with selective ^13^C-labeling (Fig. 6g). 1D ^1^H and ^13^C spectra, 2D homonuclear ^1^H–^1^H (NOESY, TOCSY, COSY), and heteronuclear ^1^H–^13^C (HSQC, HMBC) spectra were collected for DC-34. For the 1D ^1^H titration experiment, DC-34 was first diluted in buffer A to 1 mM with a final DMSO-*d*_6_ concentration of 10%, and titrated into 0.1 mM *MYC* G4 in buffer A with varying molar ratios of DC-34 (*MYC* G4:DC-34 at 1:0, 1:0.5, 1:1, 1:1.5, 1:2, 1:3, 1:4, 1:5, 1:6). NMR spectra were processed and visualized by MestReNova for 1D experiments (mestrelab.com) and NMRPipe^66^ and XEASY^67^ for 2D and 3D experiments.

### *K*_D_ fitting of the NMR titration data

Chemical shift perturbation (CSP) of *MYC* G4 imino protons was calculated by using Eq. ( 1), in which Δ*δ*_H_ represents the change in proton value (in parts per million).

1 $${\mathrm{CSP}} = \Delta \delta _{\mathrm H}$$

A *K*_D_ value was determined by using Bindfit v0.5 software (http://supramolecular.org). Raw data, including *MYC* G4 and DC-34 concentrations and CSP values of *MYC* G4 G9, G11, G16, and G18 imino protons, were inputted into the software and a non-cooperative binding mode was used to fit the data.

### Structure calculations

NOE-derived distance restraints were calculated from ^1^H–^1^H NOESY spectra recorded with varying mixing times (80, 150, and 300 ms). In the final set of structure calculations, only data from the 300 ms mixing time was used, with validation by the data collected with 80 and 150 ms mixing times and using the ^13^C-half-filtered NOESY spectrum. The peak volumes were classified as strong, medium, weak, or very weak with upper limits of 3, 4, 5, and 6 Å, respectively. The NOE crosspeak between the thymine base methyl and H6 protons was used for referencing, with an upper limit distance of 3.0 Å. Hydrogen bond restraints were set to 2.7–3.1 Å for acceptor–donor N7-N2 or O6-N1 pairs and 1.9–2.1 Å for acceptor–donor N7-H21 or O6-H1 pairs. Two K^+^ ions were incorporated within the G-tetrads by 16 electrostatic bond restraints between G-tetrad O6 atoms and the K^+^ ions. The anti configuration of the G-tetrads was experimentally determined based on intensity of intra-residue H8-H1’ crosspeaks, and dihedral angle restraints for these glycosidic torsion angles (*χ*) set to −158 ± 50° during structure calculations. In addition, planarity restraints were introduced for G-tetrads G7–G11–G16–G20, G8–G12–G17–G21, and G9–G13–G18–G22 by defining guanine N3, C6, N7, and N9 atoms to be within in a plane. Altogether, 907 intramolecular and 45 intermolecular NOE-derived distance restraints, 27 hydrogen bond restraints, 16 coordination bond restraints, 12 dihedral angles restraints, and 24 planarity restraints (Table 1) were combined to calculate the structure of the *MYC* G4/DC-34 complex by using simulated annealing in XPLOR-NIH 2.45 with the RNA-ff1 force field^68^. During the calculations, thymine methyl protons were replaced by pseudo-atoms.

The overall structure calculation was performed through two sequential stages, as described in the Xplor-NIH distribution package^68^, whereby the structure of *MYC* G4 was established followed by that of the *MYC* G4/DC-34 complex. For the first stage, 20 linear starting structures of *MYC* G4 with two K^+^ ions were subjected to simulated annealing with all restraints mentioned above and in Table 1 except for the intermolecular NOE-derived distance restraints. The lowest energy *MYC* G4 structure with best geometry was then used as the starting structure for the second stage with two DC-34 molecules added. The topology and parameter files of DC-34 were generated by the GlycoBioChem PRODRG2 Server^69^. A second iteration of simulated annealing was performed with all restraints included (Table 1) to generate 100 structures. For each stage of the structure calculations, simulated annealing was performed at high temperature (3000 K), followed by cooling with 1 K decrements to 25 K, and the resulting structures subjected to 500 steps of Powell minimization.

The 15 lowest energy structures without distance or dihedral angle violations greater than 0.5 Å or 5°, respectively were selected for presentation and statistical analyses. Visualization was performed with PyMOL (PyMOL Molecular Graphics System, http://www.pymol.org), UCSF Chimera^70^ and Shrödinger Maestro (www.schrodinger.com).

### Model generation

The lowest energy *MYC* G4/DC-34 structure was modified by replacing T23 with the wild-type G23 in UCSF Chimera v1.11.2 to generate a starting model structure for *MYC* G4 T23G/DC-34. This structure was subsequently energy minimized by Shrödinger Maestro. Only G22-A25 and the nearby DC-34 molecule were free to move during minimization. To generate a model *KRAS* G4/DC-34 structure, the G-tetrad region of the lowest energy *MYC* G4/DC-34 structure was superimposed onto the corresponding region of an available *KRAS* G4 structure (PDB 5I2V) in PyMOL.

### RNA isolation for nanostring nCounter® gene expression

RNA was isolated from cells using Trizol reagent (Sigma) and further purified using a Qiagen RNeasy Mini Kit. Isolated RNA was eluted in a 30 μL volume and its purity assessed using a NanoDrop ND-1000 spectrophotometer (OD 260/280 nm 1.7–2.5).

### NanoString nCounter® gene expression quantification

RNA (100 ng) was analyzed by NanoString nCounter XT CodeSet Gene Expression Assays which delivers direct, multiplexed measurements of gene expression through digital readouts of the abundance of mRNA transcripts. The nCounter XT CodeSet Gene Expression Assay system uses gene-specific probe pairs that hybridize directly to the mRNA sample in solution eliminating any enzymatic reactions that might introduce bias in the results. A Reporter Probe carries the fluorescent signal; a Capture Probe allows the complex to be immobilized for data collection. The nCounter XT assay simultaneously measures the expression levels of 730 target genes plus 40 endogenous control (house-keeping) genes in a single hybridization reaction using an nCounter CodeSet. Each assay run includes a reference sample consisting of in vitro transcribed RNAs of six targets that are used for normalization purposes. The raw expression data were normalized using nSolver Analysis software version 3.0. Platform specific variability was accounted for with the geometric mean of the four positive controls (ERCC_00117.1, ERCC_00112.1, ERCC_00002.1, and ERCC_00002.1), followed by assay-specific normalization with the geometric mean of six house-keeping genes (AGK, EDC3, FCF1, MRPS5, PRPF38A, and USP39) chosen by the geNorm algorithm. Genes with low expression in all samples (normalized expression <50) or with small treatment effect at all time points (expression change compared to the untreated sample less than 1.5-fold) were removed, leaving 141 genes for the subsequent analysis. Before the analysis the normalized data were log base 2 transformed.

### Synthetic procedures and compound characterization

Chemical synthesis and compound characterization are show in Supplementary Methods and Supplementary Figs. 12-81.

## Electronic supplementary material

Supplementary Information

## Data Availability

The structural coordinates and chemical shift data for the *MYC* G4 /DC-34 complex have been deposited into the Protein Data Bank (PDB) and Biological Magnetic Resonance Data Bank (BMRB) with respective accession codes 5W77 and 27144, respectively. The data that support the findings of this study are available from the corresponding authors upon request.

## References

[1] Burge S, Parkinson GN, Hazel P, Todd AK, Neidle S (2006). Quadruplex DNA: sequence, topology and structure. Nucleic Acids Res..

[2] Bacolla A, Wells RD (2004). Non-B DNA conformations, genomic rearrangements, and human disease. J. Biol. Chem..

[3] Neidle S (2016). Quadruplex nucleic acids as novel therapeutic targets. J. Med. Chem..

[4] Neidle Stephen (2017). Quadruplex nucleic acids as targets for anticancer therapeutics. Nature Reviews Chemistry.

[5] Thomas JR, Hergenrother PJ (2008). Targeting RNA with small molecules. Chem. Rev..

[6] Ali A, Bhattacharya S (2014). DNA binders in clinical trials and chemotherapy. Bioorgan. Med. Chem..

[7] Gregory MA, Hann SR (2000). c-Myc proteolysis by the ubiquitin-proteasome pathway: Stabilization of c-Myc in Burkitt’s lymphoma cells. Mol. Cell Biol..

[8] Whitfield JR, Beaulieu ME, Soucek L (2017). Strategies to inhibit Myc and their clinical applicability. Front. Cell Dev. Biol..

[9] Bretones G, Delgado MD, Leon J (2015). Myc and cell cycle control. Biochim Biophys Acta.

[10] Dang CV (2012). MYC on the path to cancer. Cell.

[11] Lin CY (2012). Transcriptional amplification in tumor cells with elevated c-Myc. Cell.

[12] Nie ZQ (2012). c-Myc is a universal amplifier of expressed genes in lymphocytes and embryonic stem cells. Cell.

[13] Cui JJ, Waltman P, Le VH, Lewis EA (2013). The effect of molecular crowding on the stability of human c-MYC promoter sequence I-motif at neutral pH. Molecules.

[14] Zhang L (2010). The impact of C-MYC gene expression on gastric cancer cell. Mol. Cell Biochem..

[15] Dang CV, Reddy EP, Shokat KM, Soucek L (2017). Drugging the ‘undruggable’ cancer targets. Nat. Rev. Cancer.

[16] Rhodes D, Lipps HJ (2015). G-quadruplexes and their regulatory roles in biology. Nucleic Acids Res..

[17] Nasiri HR (2014). Targeting a c-MYC G-quadruplex DNA with a fragment library. Chem. Commun. (Camb.)..

[18] Ohnmacht SA, Neidle S (2014). Small-molecule quadruplex-targeted drug discovery. Bioorg. Med. Chem. Lett..

[19] Hansel-Hertsch R (2016). G-quadruplex structures mark human regulatory chromatin. Nat. Genet..

[20] Kwok CK, Marsico G, Sahakyan AB, Chambers VS, Balasubramanian S (2016). rG4-seq reveals widespread formation of G-quadruplex structures in the human transcriptome. Nat. Methods.

[21] Chambers VS (2015). High-throughput sequencing of DNA G-quadruplex structures in the human genome. Nat. Biotechnol..

[22] Deng N, Wickstrom L, Cieplak P, Lin C, Yang D (2017). Resolving the ligand-binding specificity in c-MYC G-quadruplex DNA: absolute binding free energy calculations and SPR experiment. J. Phys. Chem. B.

[23] Tomonaga T, Levens D (1996). Activating transcription from single stranded DNA. Proc. Natl Acad. Sci. USA.

[24] Boddupally PV (2012). Anticancer activity and cellular repression of c-MYC by the G-quadruplex-stabilizing 11-piperazinylquindoline is not dependent on direct targeting of the G-quadruplex in the c-MYC promoter. J. Med. Chem..

[25] Ambrus A, Chen D, Dai JX, Jones RA, Yang DZ (2005). Solution structure of the biologically relevant g-quadruplex element in the human c-MYC promoter. implications for g-quadruplex stabilization. Biochemistry.

[26] Dai JX, Carver M, Hurley LH, Yang DZ (2011). Solution structure of a 2:1 quindoline-c-MYC G-quadruplex: insights into G-quadruplex-interactive small molecule drug design. J. Am. Chem. Soc..

[27] Balasubramanian S, Hurley LH, Neidle S (2011). Targeting G-quadruplexes in gene promoters: a novel anticancer strategy?. Nat. Rev. Drug Discov..

[28] Xu H (2017). CX-5461 is a DNA G-quadruplex stabilizer with selective lethality in BRCA1/2 deficient tumours. Nat. Commun..

[29] Bidzinska J, Cimino-Reale G, Zaffaroni N, Folini M (2013). G-quadruplex structures in the human genome as novel therapeutic targets. Molecules.

[30] Felsenstein KM (2016). Small molecule microarrays enable the identification of a selective, quadruplex-binding inhibitor of MYC expression. ACS Chem. Biol..

[31] Xia L, Lee YR (2014). Regioselective synthesis of novel and diverse naphtho[1,2-b]furan-3-carboxamides and benzofuran-3-carboxamides by cascade formal [3+2] cycloaddition. RSC Adv..

[32] Salome C (2014). Benzofuran derivatives as anticancer inhibitors of mTOR signaling. Eur. J. Med. Chem..

[33] Simmons JK (2017). Cooperative targets of combined mTOR/HDAC inhibition promote MYC degradation. Mol. Cancer Ther..

[34] Chatterjee J, Mierke DF, Kessler H (2008). Conformational preference and potential templates of N-methylated cyclic pentaalanine peptides. Chem. Eur. J..

[35] Agrawal P, Lin C, Mathad RI, Carver M, Yang DZ (2014). The major G-quadruplex formed in the human BCL-2 proximal promoter adopts a parallel structure with a 13-nt loop in K+solution. J. Am. Chem. Soc..

[36] Agrawal P, Hatzakis E, Guo KX, Carver M, Yang DZ (2013). Solution structure of the major G-quadruplex formed in the human VEGF promoter in K+: insights into loop interactions of the parallel G-quadruplexes. Nucleic Acids Res..

[37] Brito H (2015). Targeting KRAS oncogene in colon cancer cells with 7-carboxylate indolo[3,2-b]quinoline tri-alkylamine derivatives. PLoS ONE.

[38] Palumbo SL (2008). A novel G-quadruplex-forming GGA repeat region in the c-myb promoter is a critical regulator of promoter activity. Nucleic Acids Res..

[39] De Armond R, Wood S, Sun D, Hurley LH, Ebbinghaus SW (2005). Evidence for the presence of a guanine quadruplex forming region within a polypurine tract of the hypoxia inducible factor 1alpha promoter. Biochemistry.

[40] Moye AL (2015). Telomeric G-quadruplexes are a substrate and site of localization for human telomerase. Nat. Commun..

[41] Yoshida W, Saito T, Yokoyama T, Ferri S, Ikebukuro K (2013). Aptamer selection based on G4-forming promoter region. PLoS ONE.

[42] Kumari S, Bugaut A, Huppert JL, Balasubramanian S (2007). An RNA G-quadruplex in the 5 ‘ UTR of the NRAS proto-oncogene modulates translation. Nat. Chem. Biol..

[43] Brown RV, Danford FL, Gokhale V, Hurley LH, Brooks TA (2011). Demonstration that drug-targeted down-regulation of MYC in non-hodgkins lymphoma is directly mediated through the promoter G-quadruplex. J. Biol. Chem..

[44] Onel B (2016). A new G-quadruplex with hairpin loop immediately upstream of the human BCL2 P1 promoter modulates transcription. J. Am. Chem. Soc..

[45] Burger AM (2005). The G-quadruplex-interactive molecule BRACO-19 inhibits tumor growth, consistent with telomere targeting and interference with telomerase function. Cancer Res..

[46] Huang R, Bonnichon A, Claridge TDW, Leung IKH (2017). Protein-ligand binding affinity determination by the waterLOGSY method: an optimised approach considering ligand rebinding. Sci. Rep..

[47] Szczepina MG, Bleile DW, Mullegger J, Lewis AR, Pinto BM (2011). WaterLOGSY NMR experiments in conjunction with molecular-dynamics simulations identify immobilized water molecules that bridge peptide mimic MDWNMHAA to anticarbohydrate antibody SYA/J6. Chemistry.

[48] Thordarson P (2011). Determining association constants from titration experiments in supramolecular chemistry. Chem. Soc. Rev..

[49] Kim D (2005). (2R)-4-Oxo-4-[3-(trifluoromethyl)-5,6-dihydro[1,2,4]triazolo[4,3-alpha]pyrazin-7(8H)-yl]-1-(2,4,5-trifluorophenyl)butan-2-amine: a potent, orally active dipeptidyl peptidase IV inhibitor for the treatment of type 2 diabetes. J. Med. Chem..

[50] Weisberg E (2005). Characterization of AMN107, a selective inhibitor of native and mutant Bcr-Abl. Cancer Cell..

[51] Fraley ME (2006). Kinesin spindle protein (KSP) inhibitors. Part 2: The design, synthesis, and characterization of 2,4-diaryl-2,5-dihydropyrrole inhibitors of the mitotic kinesin KSP. Bioorg. Med. Chem. Lett..

[52] Bissantz C, Kuhn B, Stahl M (2010). A medicinal chemist’s guide to molecular interactions. J. Med. Chem..

[53] Gallivan JP, Dougherty DA (1999). Cation-pi interactions in structural biology. Proc. Natl Acad. Sci. USA.

[54] Mathad RI, Hatzakis E, Dai JX, Yang DZ (2011). c-MYC promoter G-quadruplex formed at the 5 ‘-end of NHE III1 element: insights into biological relevance and parallel-stranded G-quadruplex stability. Nucleic Acids Res..

[55] Micco M (2013). Structure-based design and evaluation of naphthalene diimide G-quadruplex ligands as telomere targeting agents in pancreatic cancer cells. J. Med. Chem..

[56] Chung WJ (2013). Solution structure of an intramolecular (3+1) human telomeric G-quadruplex bound to a telomestatin derivative. J. Am. Chem. Soc..

[57] Phan AT, Kuryavyi V, Gaw HY, Patel DJ (2005). Small-molecule interaction with a five-guanine-tract G-quadruplex structure from the human MYC promoter. Nat. Chem. Biol..

[58] Chung WJ, Heddi B, Hamon F, Teulade-Fichou MP, Phan AT (2014). Solution structure of a G-quadruplex bound to the bisquinolinium compound Phen-DC3. Angew. Chem. Int Ed..

[59] Ou TM (2007). Stabilization of G-quadruplex DNA and down-regulation of oncogene c-myc by quindoline derivatives. J. Med. Chem..

[60] Wirmer-Bartoschek J (2017). Solution NMR structure of a Ligand/hybrid-2-G-quadruplex complex reveals rearrangements that affect ligand binding. Angew. Chem. Int Ed..

[61] Kerkour A (2017). High-resolution three-dimensional NMR structure of the KRAS proto-oncogene promoter reveals key features of a G-quadruplex involved in transcriptional regulation. J. Biol. Chem..

[62] Dai J, Chen D, Jones RA, Hurley LH, Yang D (2006). NMR solution structure of the major G-quadruplex structure formed in the human BCL2 promoter region. Nucleic Acids Res..

[63] Tawani A, Mishra SK, Kumar A (2017). Structural insight for the recognition of G-quadruplex structure at human c-myc promoter sequence by flavonoid Quercetin. Sci. Rep..

[64] Keats JJ, Chesi M, Kuehl WM, Bergsagel PL (2007). A simple and reliable method to verify the authenticity and purity of human myeloma cell lines. Blood.

[65] Chen JQ (2013). Absolute quantitation of endogenous proteins with precision and accuracy using a capillary Western system. Anal. Biochem..

[66] Delaglio F (1995). Nmrpipe - a multidimensional spectral processing system based on unix pipes. J. Biomol. NMR.

[67] Bartels C, Xia TH, Billeter M, Guntert P, Wuthrich K (1995). The program Xeasy for computer-supported NMR spectral-analysis of biological macromolecules. J. Biomol. NMR.

[68] Bermejo GA, Clore GM, Schwieters CD (2016). Improving NMR Structures of RNA. Structure.

[69] Schuttelkopf AW, van Aalten DMF (2004). PRODRG: a tool for high-throughput crystallography of protein-ligand complexes. Acta Crystallogr. D..

[70] Pettersen EF (2004). UCSF chimera - a visualization system for exploratory research and analysis. J. Comput. Chem..

