# Supplementary material for: Chemical and structural studies provide a mechanistic basis for recognition of the MYC G-quadruplex
Source: Nat Commun. 2018 Oct 12;9:4229. doi: 10.1038/s41467-018-06315-w (PMC6185959; doi:10.1038/s41467-018-06315-w)
Supplement: Supplementary file 1 — Supplementary Information [file 41467_2018_6315_MOESM1_ESM.pdf]

Chemical and Structural Studies Provide a Mechanistic Basis for Recognition of the MYC G-Quadruplex

David R. Calabrese[1, ⊥], Xiang Chen[2, ⊥], Elena Leon[3, ⊥], Snehal Gaikwad[3], Zaw Phyo[3], William M. Hewitt[1], Stephanie Alden[1], Thomas A. Hilimire[1], Fahu He[2], Aleksandra M. Michalowski[3], John K. Simmons[3], Lindsey B. Saunders[1], Shuling Zhang[3], Daniel Connors[3], Kylie J. Walters[2, *], Beverly A. Mock[3, *], and John S. Schneekloth, Jr.[1, *]

Supplementary Information

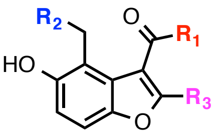

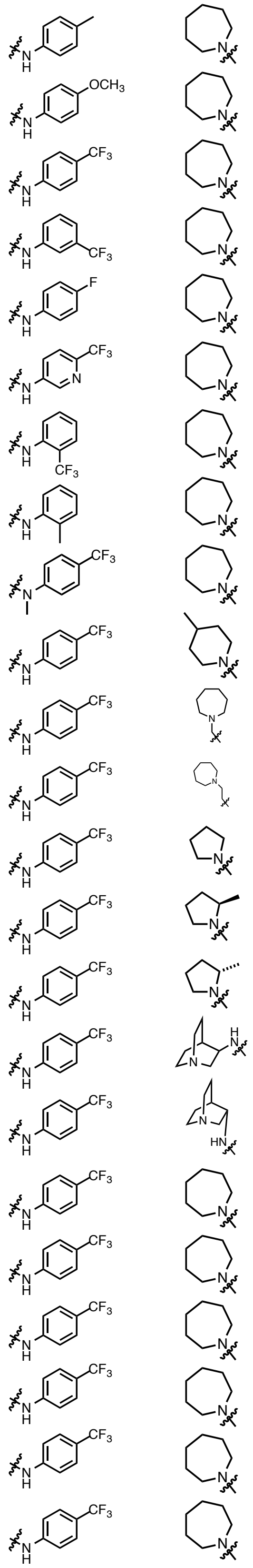

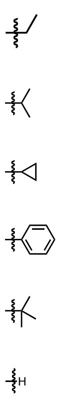

R_1	R_2	R_3	Protein %	IC50	K_D FI (µM)
		CH_3	50%	7.6 uM	23.2 ± 7.6
		CH_3	81%	7.2 uM	>100
		CH_3	0.4%	3.5 uM	9.1 ± 1.1
		CH_3	12%	3.3 uM	23.2 ± 2.5
		CH_3	N.D.	>50 uM	N.D.
		CH_3	N.D.	>50 uM	>80
		CH_3	N.D.	>50 uM	>100
		CH_3	73%	>10 uM	53.4 ± 10.9
		CH_3	N.D.	26.5 uM	57.6 ± 7.4
		CH_3	N.D.	4.8 uM	6.1 ± 2.3
		CH_3	N.D.	31.2 uM	18.8 ± 3.2
		CH_3	N.D.	>50 uM	N.D.
		CH_3	32%	1.6 uM	15.5 ± 8.5
		CH_3	N.D.	6.8 uM	15.1 ± 9.3
		CH_3	N.D.	4.5 uM	17.8 ± 11.5
		CH_3	N.D.	>50 uM	36.1 ± 10.9
		CH_3	N.D.	>50 uM	30.1 ± 13.5 µM
			N.D.	4.9 uM	> 35
			N.D.	6.4 uM	24.2
			N.D.	4.1 uM	20.1
			N.D.	4.6 uM	> 17.5
			N.D.	3.9 uM	8.9
		H	N.D.	9.3 uM	> 27.5

Supplementary Table 1: Analogs of DC-34. Compounds were evaluated as percent of

MYC protein expressed at a 10 µM dose (relative to a DMSO control), viability at 48 hours in L363 cells, and K_D (determined by fluorescence intensity assay).

Oligo name	Sequence	△ Tm	Structure
Pu27	TGG GGA GGG TGG GGA GGG TGG GGA AGG	7.5 ± 2.0	Parallel
Pu22	TGA GGG TGG GGA GGG TGG GGA A	7.6 ± 0.5	Parallel
NMR (121)	TGA GGG TGG GTA GGG TGG GTA A	6.2 ± 0.4	Parallel
131	TGA GGG TGG GTA AGG GTG GGT AA	4.6 ± 0.3	Parallel
122	TGA GGG TGG GTA GGG TAG GGT AA	7.0 ± 0.3	Parallel
221	TGA GGG TAG GGT AGG GTG GGT AA	4.0 ± 1.0	Parallel
333	TGA GGG TTA GGG TAA GGG TTA GGG TAA	0.0 ± 1.0	Anti-parallel
GAT Tail	TGA GGG TGG GGA GGG TGG GGA T	4.5 ± 0.8	Parallel
no tail	GGG TGG GGA GGG TGG G	4.3 ± 1.7	Parallel
BCl2	AGG GGC GGG CGC GGG AGG AAG GGG GCG GGA	4.2 ± 0.4	Parallel
HIF1a	GGG AGG GAG AGG GGG CGG G	0.2 ± 1.1	Parallel
KRAS	AGG GCG GTG TGG GAA GAG GGA AGA GGG GGA GGC AG	0.7 ± 0.4	Parallel
MYB	GGA GGA GGA GGT CAC GGA GGA GGA GGA GAA GGA GGA GGA GGA	1.5 ± 0.3	Parallel
Telomeric DNA	TTA GGG TTA GGG TTA GGG TTA GGG TTA	0.3 ± 0.3	Anti-parallel
VEGF	CGG GGC GGG CCG GGG GCG GGG T	<2	Parallel
dsDNA	CAA TCG GAT CGA ATT CGA TCC GAT TG	0.6 ± 0.7	B-DNA

Supplementary Table 2: ΔT_m and structural topology of G4 forming sequences. Assays were performed with DNA oligo (10 µM) in the presence of DC-34 (40 µM).

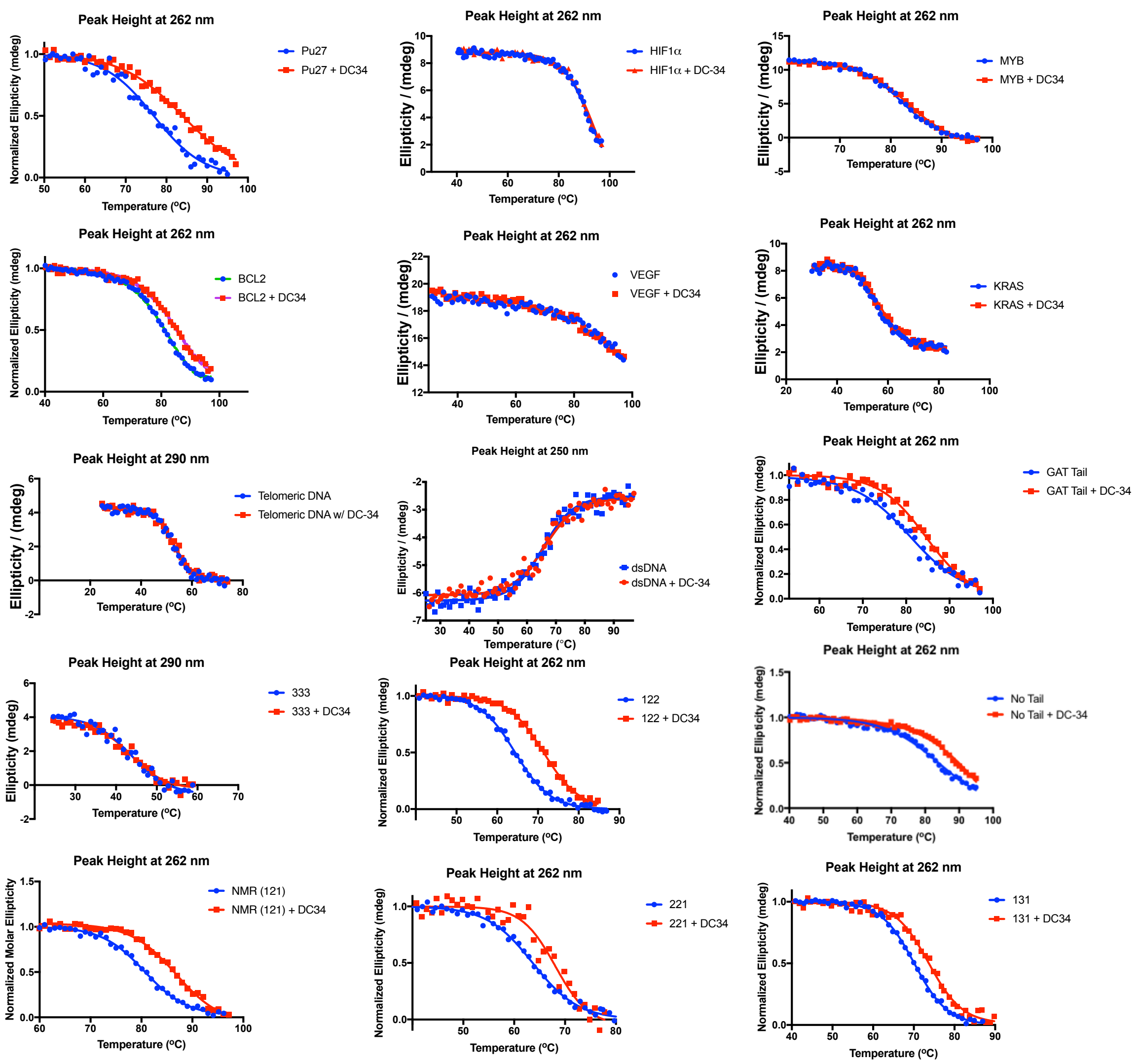

Supplementary Figure 1: CD melting temperature curves of the G4s with and without DC-34. Assays were performed with DNA oligo (10 µM) in the absence (blue) and presence (red) of compounds (40 µM) in triplicate; representative curves are shown.

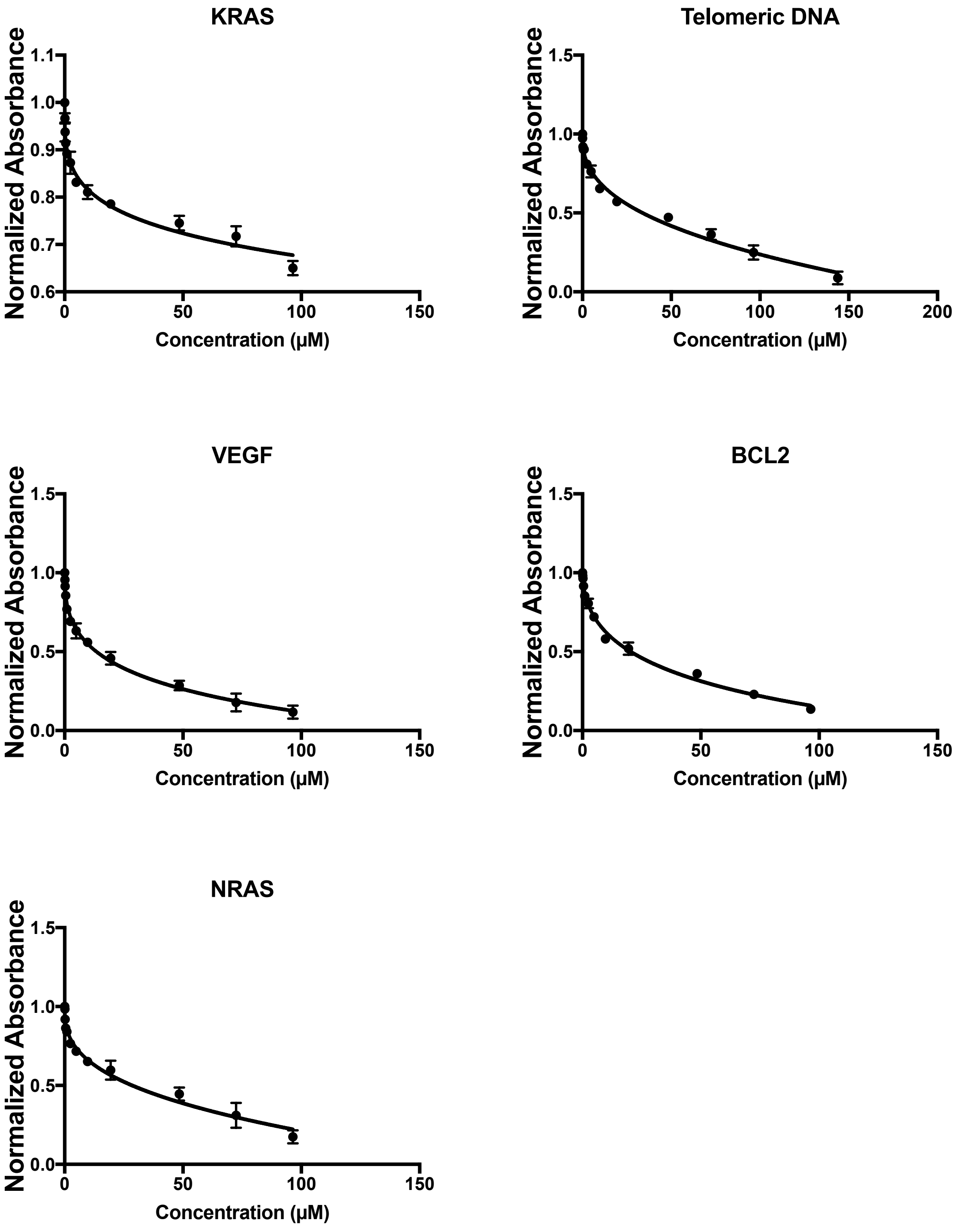

Supplementary Figure 2: Fluorescence intensity titration of 5’ Alexa Fluor labeled DNA and RNA oligos upon titration of DC-34. Assays were performed in triplicate.

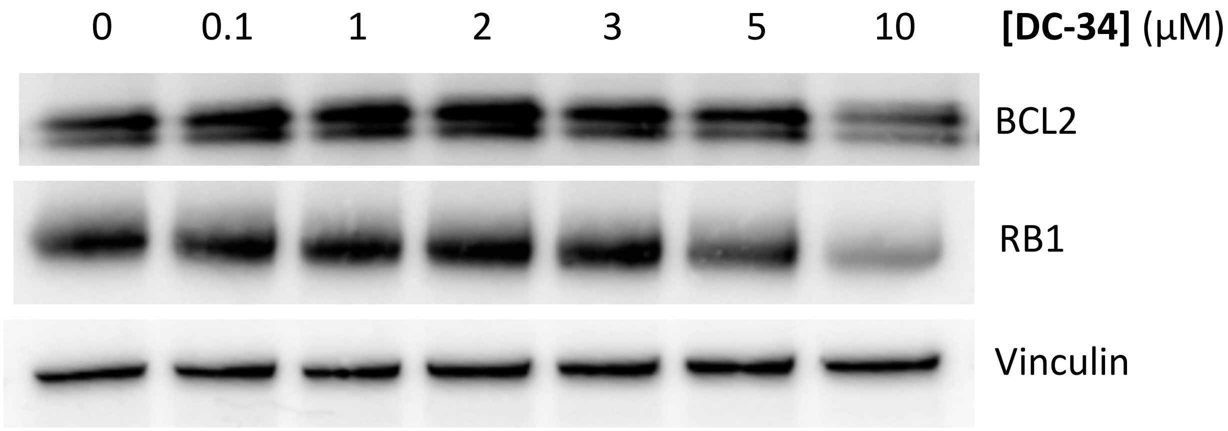

Supplementary Figure 3: Rb1 and Bcl2 protein levels expressed in L363 cells at increasing doses of DC-34 (48 hrs post-treatment).

Cell Line	IC_{50} (μM)
L363	3.44
KMS12PE	5.33
JIM1	6.35
AMO1	5.92
KMM1	4.13
KMS27	5.06
ARD	5.75
OPM1	6.48
Karpas417	4.83
H929	4.89
CA46 (Burkitt's lymphoma)	9.20
CMV-MYC Transfected 293T cells	34.00
Human Fibroblast 1634	19.55

Supplementary Table 3: The half maximal inhibitory concentration (IC_{50}) (cytotoxicity) of DC-34 against a panel of multiple myeloma cell lines, and the human fibroblast 1634 cell line. Two cell lines, CA46 Burkitt's lymphoma cell line and CMV-MYC transfected 293T cells, where MYC levels are under the control of IGH and CMV promoters, respectively, were more resistant to DC-34 cytotoxicity.

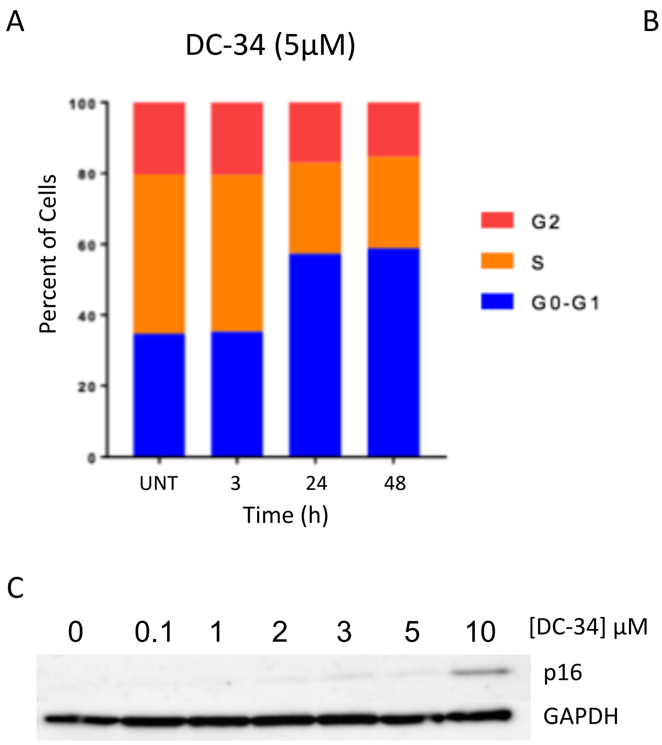

Supplementary Figure 4: Cell cycle analyses of untreated and DC-34 treated L363 cells (gated on live cells): A) Time course of percent of cells in G0, G1 and G2 after 5µM DC-34, B) Percent of cells in G0, G1 and G2 after 48 hours in response to increasing concentrations of DC-34. C) Western blot of increasing p16 protein in response to increasing concentrations of DC-34.

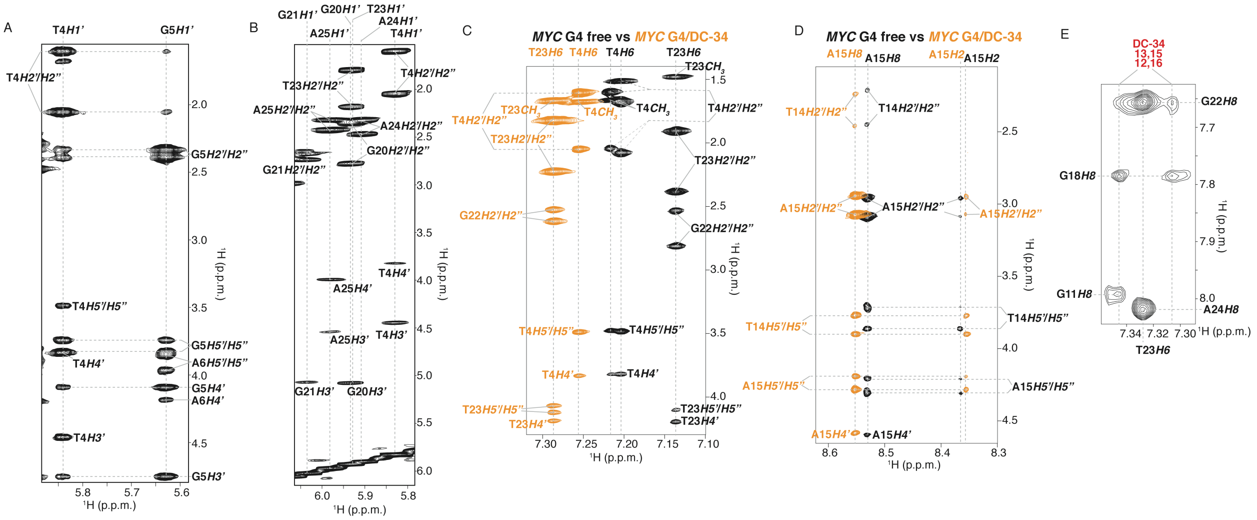

Supplementary Figure 5. NOESY spectra indicate chemical shift changes caused by DC-34 binding and intermolecular NOEs. (A,B) Example regions of 2D homonuclear NOESY (A) and TOCSY (B) spectra that were used to assign the ribose protons of *MYC* G4 with DC-34 bound. (C, D) Overlaid regions of 2D NOESY experiments acquired on free (black) and DC-34-bound (orange) *MYC* G4 highlighting chemical shift changes in the (C) G-tetrad (G22) and flanking (T4 and T23) region or (D) loop (T14 and A15) region. (E) A 2D NOESY experiment indicates intermolecular NOEs between the DC-34 trifluoromethylbenzene group and G18, G22, and G11 H8.

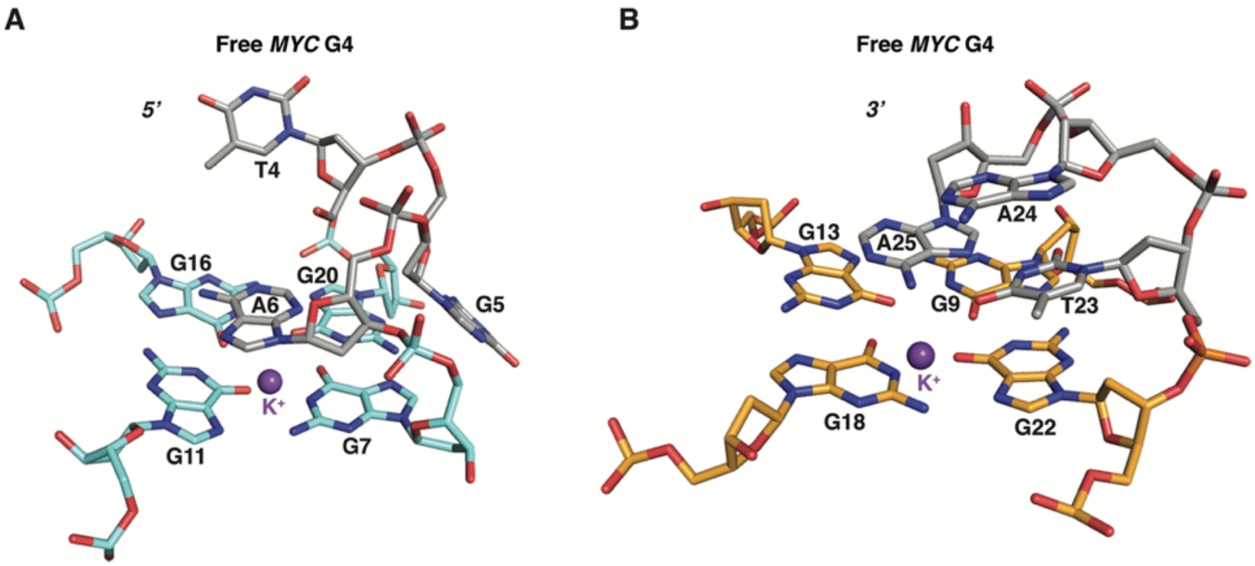

Supplementary Figure 6: Expanded views of the free *MYC* G4 structure at the 5' (A) or 3' (B) end. The color scheme follows Figure 8.

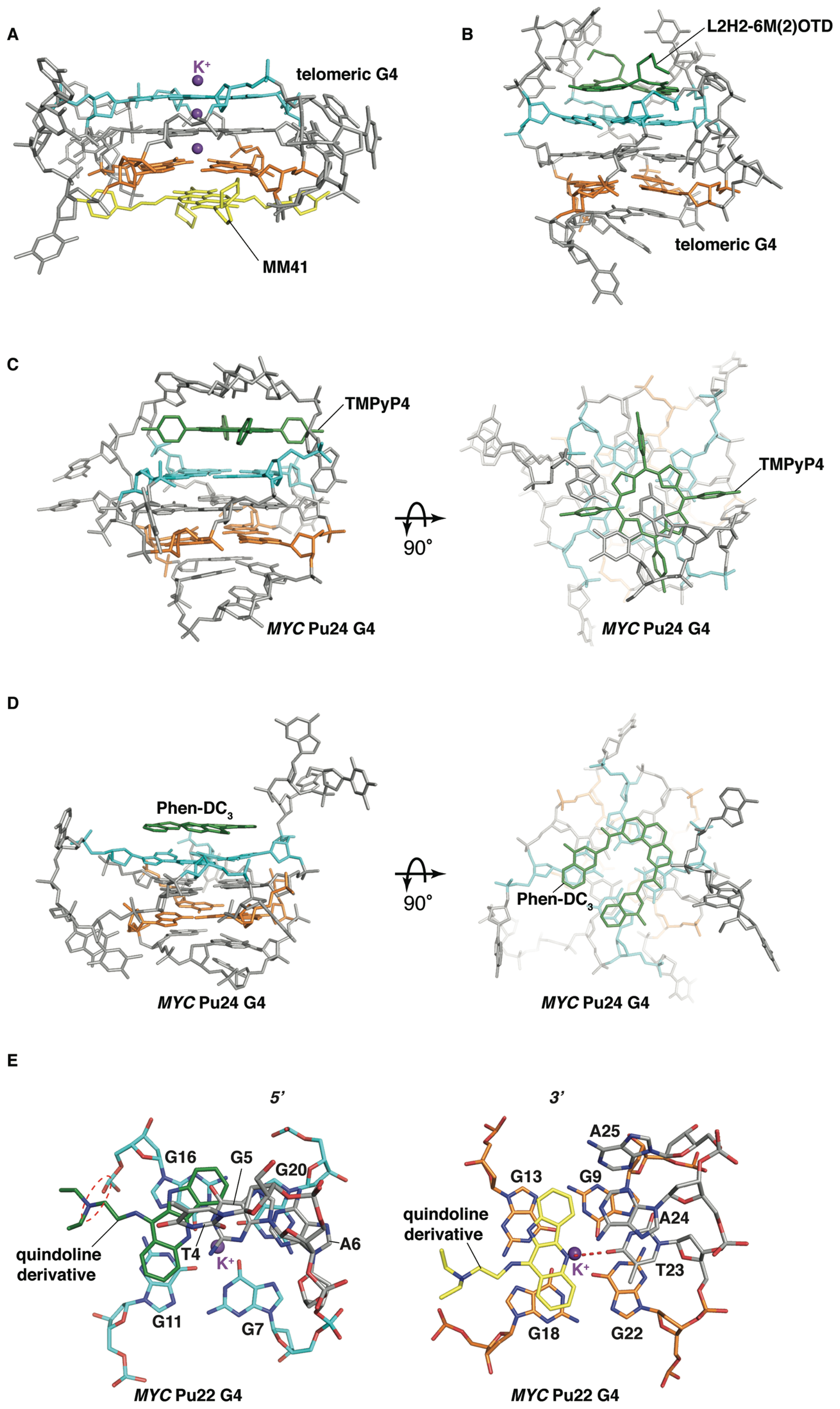
A
K+
telomeric G4
MM41
B
L2H2-6M(2)OTD
telomeric G4
C
TMPyP4
MYC Pu24 G4
90°
TMPyP4
MYC Pu24 G4
D
Phen-DC3
MYC Pu24 G4
90°
Phen-DC3
MYC Pu24 G4
E
5'
G16
G5
G20
quindoline derivative
T4
K+
A6
G11
G7
MYC Pu22 G4
3'
A25
G13
G9
quindoline derivative
A24
K+
T23
G18
G22
MYC Pu22 G4

Supplementary Figure 7. Comparison of different G4/ligand complexes. (A-D) Structures of telomeric G4/MM41 (**A**, PDB 3UYH), telomeric G4/L2H2-6M(2)OTD (**B**, PDB 2MB3), *MYC* Pu24 G4/TMPyP4 (**C**, PDB 2A5R), and *MYC* Pu24 G4/Phen-DC_3 (**D**, PDB 2MGN) are displayed with the color scheme from Figure 8. In **C** and **D**, the structures are displayed with a side (left panel) and top (right panel) view relative to the cylindrical axis of the DNA. (**E**) Expanded views of the *MYC* Pu22 G4/quindoline derivative complex to display interactions at the 5' (left) and 3' (right) ends of the *MYC* G4. A hydrogen bond between T23 O_4 of *MYC* G4 and N1 of quindoline derivative is indicated by a dashed red line; a dashed circle highlights a potential electrostatic interaction involving the G16 phosphate group of *MYC* G4 and the aminoalkylamino side chain of quindoline derivative. Potassium ions are displayed as purple spheres in (**A**) and (**E**), but missing from the PDB coordinate files for (B-D).

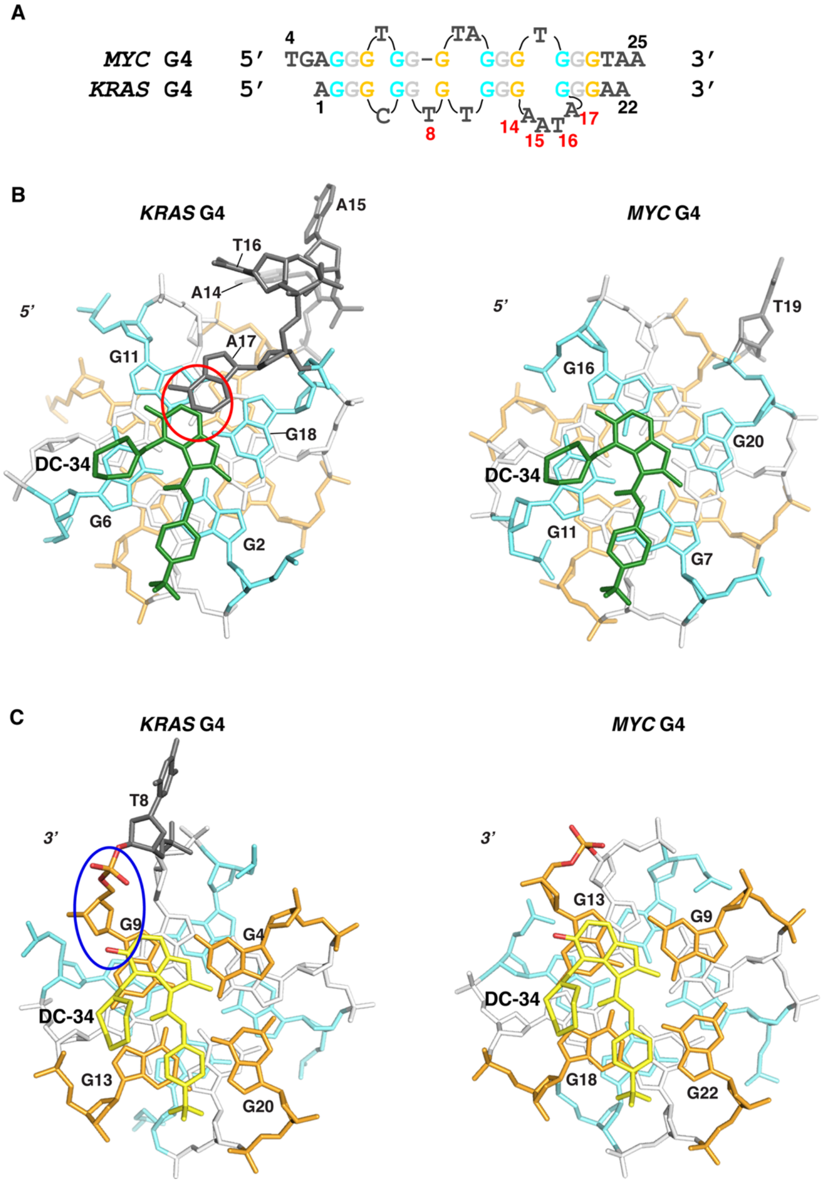

Supplementary Figure 8: Model structure of the *KRAS* G4 with DC-34. (A) Sequence alignment of the *MYC* and *KRAS* G4 region (for sequence used in PDB 5I2V). Loop residues that impact DC-34 binding in the model *KRAS*/DC-34 structure are labeled in red. (B, C) Expanded views of the *KRAS* G4/DC-34 model structure at the 5' (B) or 3' (C) end. Regions with steric clashes or electric repulsions between DC-34 and the *KRAS* G4 are highlighted by red or blue circles respectively. Comparative views of *MYC* G4/DC-34 are provided to the left. Residues from the 5', middle, or 3' G-tetrad are highlighted in blue, white, or orange, respectively. Residues in loops are highlighted in dark gray. The two DC-34 molecules are colored green and yellow and for simplicity, the two potassium ions are not shown.

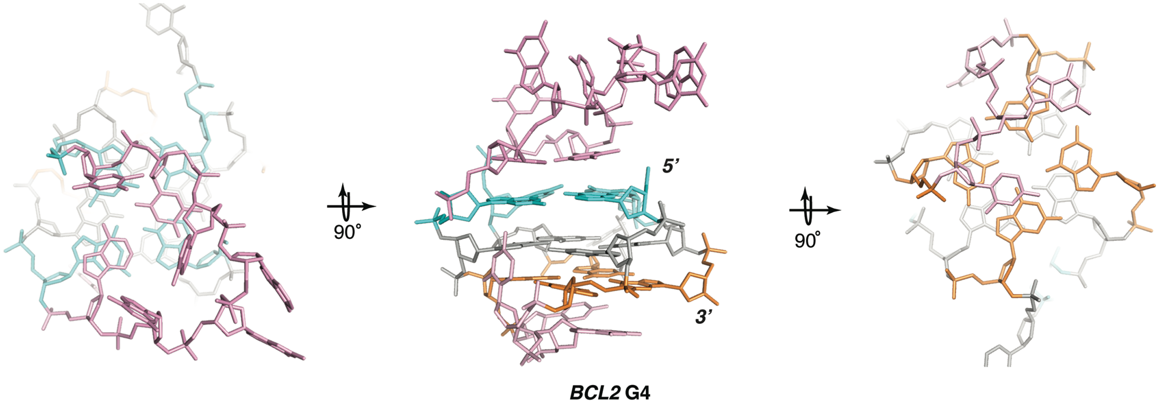

Supplementary Figure 9: The *BCL2* G4 structure is displayed with a top (left panel), side (middle panel) and bottom (right panel) view relative to the cylindrical axis of the DNA. The color scheme follows Figure 7 with the two lateral loops at 5' or 3' face in pink.

Oligos used

Fluorescence Titration

Alexafluor647-MYC (pu27): 5′ -Alexafluor647 TGG GGA GGG TGG GGA GGG TGG GGA AGG

Alexafluor647-NRAS: 5′ -Alexafluor647 UGU GGG AGG GGC GGG UCU GGG

Alexafluor647-KRAS: 5′ -Alexafluor647 AGG GCG GTG TGG GAA GAG GGA AGA GGG GGA GGC AG

Alexafluor647-BCL2: 5′ -Alexafluor647 AGG GGC GGG CGC GGG AGG AAG GGG GCG GGA

Alexafluor647-Telomeric DNA: 5′ -Alexafluor647 TTA GGG TTA GGG TTA GGG TTA GGG TTA

Alexafluor647-VEGF: 5′ -Alexafluor647 CGG GGC GGG CCG GGG GCG GGG T

NMR

NMR-MYC: 5′ - TGA GGG TGG GTA GGG TGG GTA A

SPR

Biotin MYC (pu27): 5' – BiotinTEG TGG GGA GGG TGG GGA GGG TGG GGA AGG

Thermal Melt

BCl2: 5' - AGG GGC GGG CGC GGG AGG AAG GGG GCG GGA

MYC (pu22): 5′ - TGA GGG TGG GGA GGG TGG GGA A

MYC 121 (NMR): 5' - TGA GGG TGG GTA GGG TGG GTA A

221: 5' - TGA GGG TAG GGT AGG GTG GGT AA

131: 5' - TGA GGG TGG GTA AGG GTG GGT AA

122: 5' - TGA GGG TGG GTA GGG TAG GGT AA

333: 5' - TGA GGG TTA GGG TAA GGG TTA GGG TAA

GAT Tail: 5' - TGA GGG TGG GGA GGG TGG GGA T

No Tails: 5' - GGG TGG GGA GGG TGG G

MYC (pu27): 5′ - TGG GGA GGG TGG GGA GGG TGG GGA AGG

MYB: 5'- GGA GGA GGA GGT CAC GGA GGA GGA GGA GAA GGA GGA GGA GGA

VEGF: 5' - CGG GGC GGG CCG GGG GCG GGG T

KRAS: 5' - AGG GCG GTG TGG GAA GAG GGA AGA GGG GGA GGC AG

HIF1a: 5' - GGG AGG GAG AGG GGG CGG G

Telomeric DNA: 5' – TTA GGG TTA GGG TTA GGG TTA GGG TTA

dsDNA: 5' - CAA TCG GAT CGA ATT CGA TCC GAT TG

QPCR

MYC (*forward*): TGAGGAGACACCGCCCAC

MYC (reverse): CAACATCGATTTCTTCCTCATCTTC

BCL2 (*forward*): GGTGGGGTCATGTGTGTGG

BCL2 (reverse): CGGTTCAGGTACTCAGTCATCC

VEGFA (*forward*): GGAGGAGGGCAGAATCATCA

VEGFA (reverse): CTTGGTGAGGTTTGATCCGC

KRAS (*forward*): GGACTGGGGAGGGCTTTCT

KRAS (reverse): GCCTGTTTTGTGTCTACTGTTCT

RB1 (*forward*): TCACATTCCTCGAAGCCCTT

RB1 (reverse): ACGGTCGCTGTTACATACCA

Synthetic Procedures and Compound Characterization

General Remarks: Flash column chromatography was performed using a Teledyne ISCO CombiFlash Rf automated chromatography system. HPLC purifications were performed on a Waters HPLC with a 2545 model pump and a Phenomenex Luna 10 micron C18 column (75 x 30 mm), using an acetonitrile/water gradient with 0.1% TFA. Proton (^{1}H NMR) and carbon (^{13}C NMR) magnetic resonance spectra where obtained in the specified deuterated solvent at 400 MHz or 500 MHz and 101 MHz or 126 MHz, respectively. The following abbreviations were utilized to describe peak patterns: br = broad, s = singlet, d = doublet, t = triplet, q = quartet, app = apparent, and m = multiplet. Unless otherwise noted, all chemicals were obtained from Sigma Aldrich and used without further purification. Toluene, acetonitrile, dimethylformamide, and dichloromethane were obtained from GlassContour Solvent Systems and were dried over alumina under an argon atmosphere.

HRMS. High resolution mass spectrometry data were acquired on an Agilent 6520 Accurate-Mass Q-TOF LC/MS System, (Agilent Technologies, Inc.) equipped with a dual electro-spray source, operated in the positive-ion mode. Separation was performed on a Zorbax 300SB-C18 Poroshell column (2.1 mm x 150 mm; particle size 5 mm). The analytes were eluted using a water/acetonitrile gradient with 0.1% formic acid. Data were acquired at high resolution (1,700 *m/z*), 4 GHz. To maintain mass accuracy during the run time, an internal mass calibration sample was infused continuously during the LC/MS runs. Data acquisition and analysis were performed using MassHunter Workstation Data Software, LCMS Data Acquisition (version B.06.01) and Qualitative Analysis (version B.07.00).

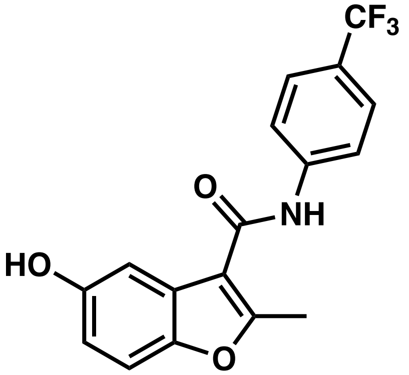

5-hydroxy-2-methyl-*N*-(4-(trifluoromethyl)phenyl)benzofuran-3-carboxamide. (1) To a solution of acetomitrile (10 mL), 3-oxo-*N*-(4-(trifluoromethyl)phenyl)butanamide (350 mg, 1.43 mmol, 1 eq), p-benzoquinone (154 mg, 1.43 mmol, 1 eq), and In(OTf)$_3$ (40 mg, 71 µmol, 5 mol %) was added. The solution was stirred at room temperature for 16 h. The solvent was removed under reduced pressure. The residue was purified by chromatography on silica gel to afford the desired product (236 mg, 49%). A small amount of HPLC purified product was used for analytical characterization. ^{1}H NMR (500 MHz, DMSO-d_6) δ 10.38 (s, 1H), 9.32 (s, 1H), 7.94 (d, *J* = 8.5 Hz, 2H), 7.73 (d, *J* = 8.5 Hz, 2H), 7.39 (d, *J* = 8.9 Hz, 1H), 7.05 (d, *J* = 2.5 Hz, 1H), 6.75 (dd, *J* = 8.8, 2.5 Hz, 1H), 2.62 (s, 3H). ^{13}C NMR (126 MHz, DMSO-d_6) δ 162.45, 158.75, 153.81, 146.99, 142.71, 126.66, 126.01 (q, *J* = 3.8 Hz), 124.41 (q, J = 270.9 Hz), 123.52 (q, *J* = 31.9 Hz), 119.80, 113.13, 112.85, 111.25, 105.15, 13.83. HRMS for $C_{17}H_{12}F_3NO_3$ $[M+H]^+$ Calc: 336.08420, found: 336.08470 (error = -1.22 ppm).

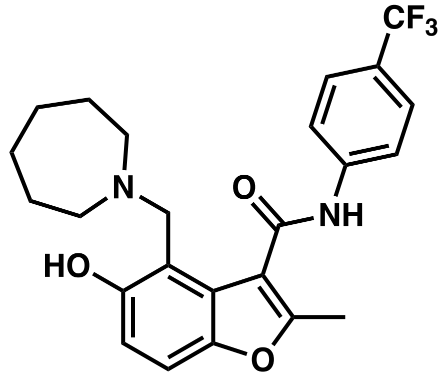

4-(azepan-1-ylmethyl)-5-hydroxy-2-methyl-*N*-(4-(trifluoromethyl)phenyl)benzofuran-3-carboxamide. (DC-34) A solution of 5-hydroxy-2-methyl-*N*-(4-(trifluoromethyl)phenyl)benzofuran-3-carboxamide (215 mg, 0.642 mmol,1 eq), 37% aq. formaldihyde (104 µL, 1.28 mmol, 2 eq), and azepane (144 µL, 1.28 mmol, 2 eq) in EtOH (3 mL) was heated to 70 °C for 6 h. After the reaction was cooled to room temperature, the solvent was removed under reduced pressure. The residue was purified by chromatography on silica gel to afford the desired product (66%, 188 mg). A small amount of HPLC purified product was used for analytical characterization as the trifluoroacetic acid salt. ^{1}H NMR (500 MHz, DMSO-d_6) δ 10.99 (s, 1H), 10.36 (s, 1H), 8.92 (s, 1H), 7.96 (d, *J* = 8.5 Hz, 2H), 7.80 (d, *J* = 8.6 Hz, 2H), 7.59 (d, *J* = 8.9 Hz, 1H), 7.00 (d, *J* = 8.9 Hz, 1H), 4.47 (d, *J* = 5.5 Hz, 2H), 3.35 – 3.27 (m, 2H), 3.25 – 3.13 (m, 2H), 2.62 (s, 3H), 1.83 (dt, *J* = 14.7, 7.1 Hz, 2H), 1.77 – 1.67 (m, 2H), 1.65 – 1.46 (m, 4H). ^{13}C NMR (126 MHz, DMSO-d_6) δ 164.08, 158.34, 157.72 (q, *J* = 30.9 Hz), 153.75, 147.16, 142.09, 126.64, 126.25 (q, *J* = 3.7 Hz), 124.35 (q, *J* = 32.1 Hz), 124.28 (q, *J* = 271.6 Hz), 120.06, 117.21 (q, *J* = 300.3 Hz), 113.86, 113.55, 112.96, 107.31, 53.73, 51.54, 26.65, 22.47, 14.10. HRMS for $C_{24}H_{25}F_3N_2O_3$ $[M+H]^+$ calc: 447.18900, found: 447.19002 (error = -2.11 ppm).

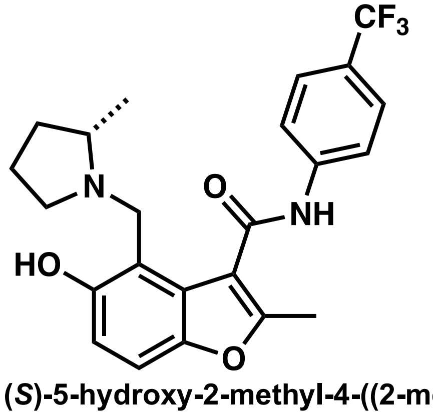

(*S*)-5-hydroxy-2-methyl-4-((2-methylpyrrolidin-1-yl)methyl)-*N*-(4-(trifluoromethyl)phenyl)benzofuran-3-carboxamide. (2) A solution of 5-hydroxy-2-methyl-*N*-(4-(trifluoromethyl)phenyl)benzofuran-3-carboxamide (46 mg, 0.14 mmol,1 eq), 37% aq. formaldihyde (22 µL, 0.28 mmol, 2 eq), and amine (26 µL, 0.28 mmol, 2 eq) in EtOH (2 mL) was heated to 70 °C for 6 h. After the reaction was cooled to room temperature, the solvent was removed under reduced pressure. The residue was purified by chromatography on silica gel to afford the desired product (77%, 46 mg). A small amount of HPLC purified product was used for analytical characterization as the trifluoroacetic acid salt. ^{1}H NMR (400 MHz, DMSO-d_6) δ 10.95 (s, 1H), 10.45 (s, 1H), 8.83 (s, 1H), 7.96 (d, *J* = 8.4 Hz, 2H), 7.78 (d, *J* = 8.5 Hz, 2H), 7.58 (d, *J* = 8.9 Hz, 1H),

7.02 (d, *J* = 8.9 Hz, 1H), 4.63 (dd, *J* = 13.2, 3.9 Hz, 1H), 4.43 (dd, *J* = 13.1, 7.3 Hz, 1H), 3.58 (dq, *J* = 8.7, 6.6 Hz, 1H), 3.26 (ddt, *J* = 25.6, 11.3, 6.7 Hz, 2H), 2.61 (s, 3H), 2.26 – 2.11 (m, 1H), 1.95 – 1.81 (m, 2H), 1.60 (dq, *J* = 12.8, 8.5 Hz, 1H), 1.30 (d, *J* = 6.5 Hz, 3H). ^{13}C NMR (101 MHz, DMSO-*d*$_6$) δ 163.77, 158.23, 158.10 (q, J = 33.8 Hz), 153.57, 147.16, 142.14, 126.54, 126.19 (q, *J* = 3.9 Hz), 124.30 (q, J = 271.4 Hz), 124.27 (q, *J* = 32.1 Hz), 120.05, 116.42 (q, *J* = 296.3 Hz), 113.84, 113.33, 112.85, 107.83, 64.13, 53.17, 48.39, 31.12, 21.27, 16.40, 14.00. HRMS for $C_{23}H_{23}F_3N_2O_3$ $[M+H]^+$ calc: 433.17335, found: 433.17419 (error = -1.65 ppm).

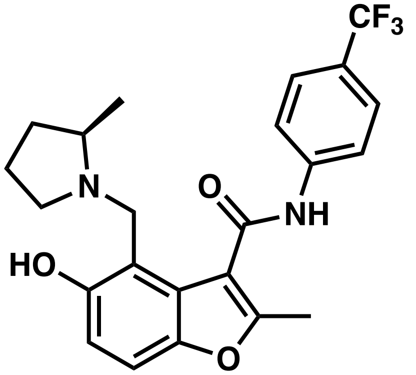

(*R*)-5-hydroxy-2-methyl-4-((2-methylpyrrolidin-1-yl)methyl)-*N*-(4-(trifluoromethyl)phenyl)benzofuran-3-carboxamide. (3) A solution of 5-hydroxy-2-methyl-*N*-(4-(trifluoromethyl)phenyl)benzofuran-3-carboxamide (24 mg, 72 µmol,1 eq), 37% aq. formaldihyde (12 µL, 143 µmol, 2 eq), and amine (14 µL, 143 µmol, 2 eq) in EtOH (1 mL) was heated to 70 °C for 6 h. After the reaction was cooled to room temperature, the solvent was removed under reduced pressure. The residue was purified by chromatography on silica gel to afford the desired product (49%, 15 mg). A small amount of HPLC purified product was used for analytical characterization as the trifluoroacetic acid salt. ^{1}H NMR (400 MHz, DMSO-*d*$_6$) δ 10.79 (s, 1H), 7.95 (d, *J* = 8.4 Hz, 2H), 7.73 (d, *J* = 8.6 Hz, 2H), 7.30 (d, *J* = 8.8 Hz, 1H), 6.72 (d, *J* = 8.7 Hz, 1H), 4.11 (d, *J* = 13.9 Hz, 1H), 3.69 (d, *J* = 13.9 Hz, 1H), 2.77 (dt, *J* = 9.6, 5.8 Hz, 1H), 2.48 (s, 3H), 2.39 (q, *J* = 6.9 Hz, 1H), 2.18 (q, *J* = 8.7 Hz, 1H), 1.93 – 1.80 (m, 1H), 1.61 – 1.49 (m, 2H), 1.34 – 1.21 (m, 1H), 0.97 (d, *J* = 6.1 Hz, 3H). ^{13}C NMR (126 MHz, DMSO-*d*$_6$) δ 163.77, 158.27, 157.95 (q, *J* = 32.9 Hz), 153.53, 147.17, 142.12, 126.55, 126.23 (q, *J* = 3.7 Hz), 124.30 (q, *J* = 271.4 Hz), 124.29 (q, *J* = 31.9 Hz), 120.06, 113.84, 113.37, 112.83, 107.85, 64.17, 53.18, 48.42, 31.14, 21.28, 16.43, 14.04. HRMS for $C_{23}H_{23}F_3N_2O_3$ $[M+H]^+$ calc: 433.17335, found: 433.17567 (error = -5.20 ppm).

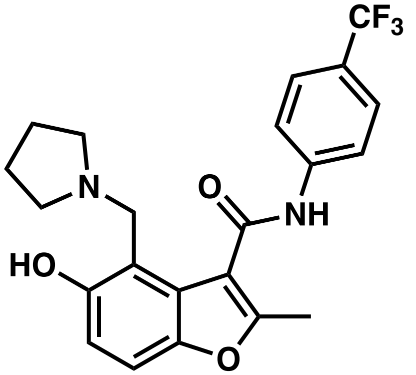

5-hydroxy-2-methyl-4-(pyrrolidin-1-ylmethyl)-*N*-(4-(trifluoromethyl)phenyl)benzofuran-3-carboxamide. (4) A solution of 5-hydroxy-2-methyl-*N*-(4-(trifluoromethyl)phenyl)benzofuran-3-carboxamide (32 mg, 88.2 µmol,1 eq), 37% aq. formaldihyde (µL, 0.176 mmol, 2 eq), and amine (µL, 0.176 mmol, 2 eq) in EtOH (2 mL) was heated to 70 °C for 6 h. After the reaction was cooled to room temperature, the solvent was removed under reduced pressure. The residue was purified by chromatography on silica gel to afford the desired product (81%, 30 mg). A small amount of HPLC purified product was used for analytical characterization as the trifluoroacetic acid salt. ^{1}H NMR (500 MHz, DMSO-d_6) δ 10.90 (s, 1H), 10.29 (s, 1H), 9.17 (d, *J* = 6.9 Hz, 1H), 7.97 (d, *J* = 8.5 Hz, 2H), 7.79 (d, *J* = 8.6 Hz, 2H), 7.57 (d, *J* = 8.9 Hz, 1H), 6.99 (d, *J* = 8.9 Hz, 1H), 4.47 (d, *J* = 5.5 Hz, 2H), 3.45 (m, 2H), 3.29 – 3.15 (m, 2H), 2.62 (s, 3H), 2.08 – 1.97 (m, 2H), 1.90 (dt, *J* = 8.8, 4.2 Hz, 2H). ^{13}C NMR (126 MHz, DMSO-d_6) δ 163.82, 158.20, 157.94 (q, *J* = 33.1 Hz), 153.34, 147.13, 142.21, 126.69, 126.25 (q, *J* = 4.1 Hz), 124.32, 124.25 (q, *J* = 31.7 Hz), 120.04, 116.66 (q, *J* = 296.4 Hz), 113.93, 113.32, 113.00, 108.06, 53.75, 49.52, 22.59, 14.12. HRMS for $C_{22}H_{21}F_3N_2O_3$ $[M+H]^+$ calc: 419.5770, found: 419.15814 (error = -0.76 ppm).

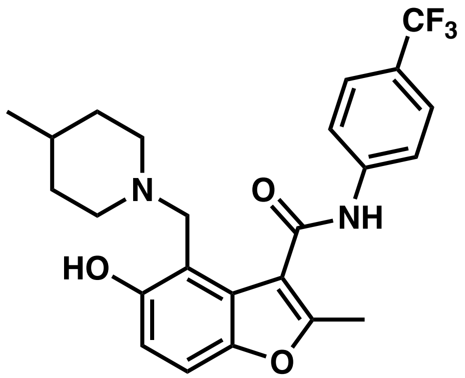

5-hydroxy-2-methyl-4-((4-methylpiperidin-1-yl)methyl)-*N*-(4-(trifluoromethyl)phenyl)benzofuran-3-carboxamide. (5) A solution of 5-hydroxy-2-methyl-*N*-(4-(trifluoromethyl)phenyl)benzofuran-3-carboxamide (60 mg, 0.179 mmol,1 eq), 37% aq. formaldihyde (29 µL, 0.358 mmol, 2 eq), and amine (42 µL, 0.358 mmol, 2 eq) in EtOH (2 mL) was heated to 70 °C for 6 h. After the reaction was cooled to room temperature, the solvent was removed under reduced pressure. The residue was purified by chromatography on silica gel to afford the desired product (79%, 63 mg). A small amount of HPLC purified product was used for analytical characterization as the trifluoroacetic acid salt. ^{1}H NMR (400 MHz, DMSO-d_6) δ 10.69 (s, 1H), 7.96 (d, *J* = 8.4 Hz, 2H), 7.72 (d, *J* = 8.5 Hz, 2H), 7.28 (d, *J* = 8.8 Hz, 1H), 6.74 (d, *J* = 8.8 Hz, 1H), 3.70 (s, 2H), 2.65 (d, *J* = 10.9 Hz, 2H), 2.48 (s, 4H), 1.83 (td, *J* = 11.8, 2.4 Hz, 2H), 1.46 – 1.32 (m, 2H), 1.24 (m, 1H), 0.88 (qd, *J* = 12.0, 3.8 Hz, 2H), 0.73 (d, *J* = 6.5 Hz, 3H). ^{13}C NMR (126 MHz, DMSO-d_6) δ 163.34, 155.16, 152.98, 147.13, 143.01, 126.07 (q, *J* = 3.8 Hz), 125.67, 124.43 (q, *J* = 271.4 Hz), 123.36 (q, *J* = 31.8 Hz), 119.08, 115.24, 114.27, 112.88, 109.69, 53.97, 52.55, 33.51, 30.01, 21.64, 13.32. HRMS for $C_{24}H_{25}F_3N_2O_3$ $[M+H]^+$ calc: 447.18900, found: 447.19005 (error = -2.05 ppm).

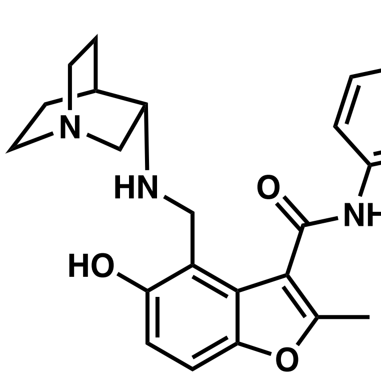

5-hydroxy-2-methyl-4-((((1*R*,3*S*,4*R*)-quinuclidin-3-yl)amino)methyl)-*N*-(4-(trifluoromethyl)phenyl)benzofuran-3-carboxamide. (6) A solution of 5-hydroxy-2-methyl-*N*-(4-(trifluoromethyl)phenyl)benzofuran-3-carboxamide (60 mg, 0.18 mmol,1 eq), 37% aq. formaldihyde (29 μL, 0.36 mmol, 2 eq), triethyl amine (75 μL, 0.54 mmol, 3 eq), and amine (71 μL, 0.36 mmol, 2 eq) in EtOH (2 mL) was heated to 70 °C for 6 h. After the reaction was cooled to room temperature, the solvent was removed under reduced pressure. The residue was purified by chromatography on silica gel to afford the desired product. (48%, 41 mg). A small amount of HPLC purified product was used for analytical characterization as the trifluoroacetic acid salt. ^{1}H NMR (500 MHz, DMSO-d_6) δ 10.91 (s, 1H), 10.43 (s, 1H), 9.13 (s, 1H), 8.85 (s, 1H), 7.97 (d, *J* = 8.5 Hz, 2H), 7.78 (d, *J* = 8.5 Hz, 2H), 7.56 (d, *J* = 8.9 Hz, 1H), 7.02 (d, *J* = 8.9 Hz, 1H), 4.48 – 4.28 (m, 2H), 3.75 (s, 1H), 3.72 – 3.63 (m, 1H), 3.28 (tt, *J* = 11.6, 5.9 Hz, 4H), 3.22 – 3.13 (m, 1H), 2.64 (s, 3H), 2.54 (q, *J* = 3.1 Hz, 1H), 2.18 (dtt, *J* = 10.0, 7.0, 3.2 Hz, 1H), 2.00 – 1.87 (m, 1H), 1.87 – 1.71 (m, 1H). ^{13}C NMR (126 MHz, DMSO-d_6) δ 163.97, 158.39 (q, *J* = 33.9 Hz), 158.34, 153.47, 147.15, 142.19, 126.33, 126.16 (q, *J* = 3.7 Hz), 124.32 (q, *J* = 271.4 Hz), 124.27 (q, *J* = 32.1 Hz), 120.17, 116.47 (q, *J* = 295.7 Hz), 113.87, 113.12, 112.91, 108.15, 52.13, 48.52, 45.48, 45.00, 41.13, 21.53, 21.33, 16.23, 14.20. HRMS for $C_{25}H_{27}F_3N_3O_3$ $[M+H]^+$ calc: 474.19990, found: 474.19919 (error = 0.76 ppm).

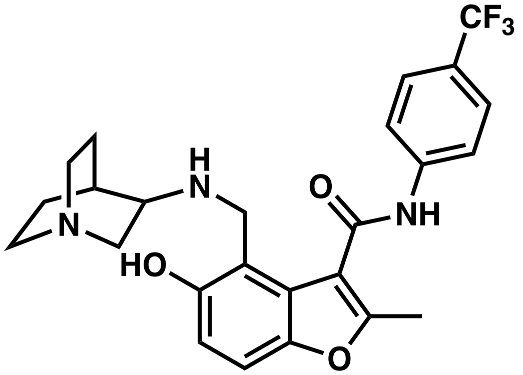

5-hydroxy-2-methyl-4-((((1*s*,4*s*)-quinuclidin-3-yl)amino)methyl)-*N*-(4-(trifluoromethyl)phenyl)benzofuran-3-carboxamide. (7) A solution of 5-hydroxy-2-methyl-*N*-(4-(trifluoromethyl)phenyl)benzofuran-3-carboxamide (60 mg, 0.18 mmol,1 eq), 37% aq. formaldihyde (29 μL, 0.36 mmol, 2 eq), triethyl amine (75 μL, 0.54 mmol, 3 eq), and amine (71 μL, 0.36 mmol, 2 eq) in EtOH (2 mL) was heated to 70 °C for 6 h. After the reaction was cooled to room temperature, the solvent was removed under reduced pressure. The residue was purified by chromatography on silica gel to afford the desired product (39%, 33 mg). A small amount of HPLC purified product was used for analytical characterization as the trifluoroacetic acid salt. ^{1}H NMR (500 MHz, DMSO-d_6) δ 10.92 (s,

1H), 10.58 – 10.42 (m, 2H), 9.14 (s, 1H), 8.86 (s, 1H), 7.97 (d, *J* = 8.5 Hz, 2H), 7.78 (d, *J* = 8.5 Hz, 2H), 7.56 (d, *J* = 8.8 Hz, 1H), 7.02 (d, *J* = 8.9 Hz, 1H), 4.41 (t, *J* = 9.8 Hz, 2H), 3.35 – 3.21 (m, 4H), 3.21 – 3.12 (m, 1H), 2.64 (s, 3H), 2.54 (q, *J* = 3.2 Hz, 1H), 2.18 (dtt, *J* = 10.0, 6.7, 3.1 Hz, 1H), 1.92 (dddd, *J* = 13.1, 9.6, 5.9, 3.4 Hz, 1H), 1.87 – 1.69 (m, 1H). ^{13}C NMR (126 MHz, DMSO-d_6) δ 163.97, 158.42 (q, *J* = 33.3 Hz), 158.32, 153.49, 147.15, 142.20, 126.33, 126.16 (q, *J* = 3.8 Hz), 124.33 (q, *J* = 271.1 Hz)jjj, 124.27 (q, *J* = 32.0 Hz), 120.17, 116.63 (q, *J* = 296.4 Hz), 113.88, 113.11, 112.92, 108.17, 52.13, 48.52, 45.47, 44.99, 41.13, 21.54, 21.34, 16.23, 14.20. HRMS for $C_{25}H_{27}F_3N_3O_3$ [M+H]$^+$ calc: 474.19990, found: 474.20065 (error = -1.28 ppm).

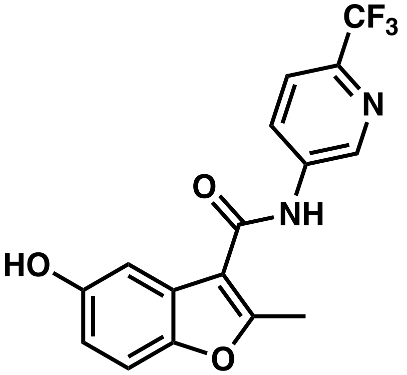

5-hydroxy-2-methyl-*N*-(6-(trifluoromethyl)pyridin-3-yl)benzofuran-3-carboxamide. (8) Ethyl acetoacetate (200 µL, 1.57 mmol, 1 eq), analine (0.254 g, 1.57 mmol, 1eq), and DMAP (48 mg, 0.393 mmol, 5 mol%) in a solution of toluene (3 mL) were heated to reflux and stirred for 14 hours. Then the reaction was cooled to room temperature. The solvent was removed under reduced pressure. The residue was purified by chromatography on silica gel to afford the desired off-white solid (150 mg). The solid was then added to p-benzoquinone (66 mg, 0.61 mmol, 1 eq) and In(OTf)$_3$ (17 mg, 30 µmol, 5 mol %) in a solution of acetonitrile (10 mL). The solution was stirred at room temperature for 16 h. The solvent was removed under reduced pressure. The residue was purified by chromatography on silica gel to afford the desired product (91 mg, 44%). A small amount of HPLC purified product was used for analytical characterization as the trifluoroacetic acid salt. ^{1}H NMR (500 MHz, DMSO-d_6) δ 10.59 (s, 1H), 9.36 (s, 1H), 9.04 (d, *J* = 2.4 Hz, 1H), 8.42 (dd, *J* = 8.6, 2.4 Hz, 1H), 7.93 (d, *J* = 8.6 Hz, 1H), 7.40 (d, *J* = 8.8 Hz, 1H), 7.09 (d, *J* = 2.5 Hz, 1H), 6.77 (dd, *J* = 8.8, 2.5 Hz, 1H), 2.64 (s, 3H). ^{13}C NMR (126 MHz, DMSO-d_6) δ 162.83, 159.45, 153.88, 147.03, 141.57, 140.67 (q, *J* = 34.1 Hz), 138.74, 127.48, 126.43, 121.82, 121.22 (q, *J* = 2.8 Hz), 112.96, 112.66, 111.33, 105.27, 13.91. HRMS for $C_{16}H_{11}F_3N_2O_3$ [M+H]$^+$ calc: 337.07945, found: 337.07982 (error = -0.84 ppm).

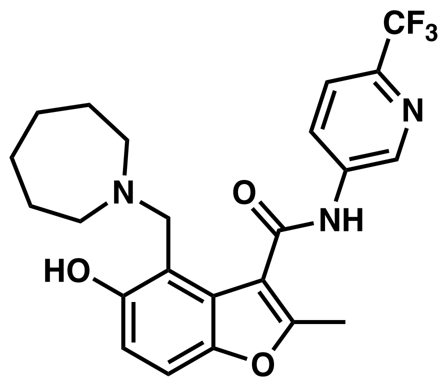

4-(azepan-1-ylmethyl)-5-hydroxy-2-methyl-*N*-(6-(trifluoromethyl)pyridin-3-yl)benzofuran-3-carboxamide. (9) A solution of 5-hydroxy-2-methyl-*N*-(6-(trifluoromethyl)pyridin-3-yl)benzofuran-3-carboxamide (40 mg, 0.12 mmol,1 eq), 37% aq. formaldihyde (19 μL, 0.24 mmol, 2 eq), and azepane (27 μL, 0.24 mmol, 2 eq) in EtOH (2 mL) was heated to 70 °C for 6 h. After the reaction was cooled to room temperature, the solvent was removed under reduced pressure. The residue was purified by chromatography on silica gel to afford the desired product (71%, 38 mg). A small amount of HPLC purified product was used for analytical characterization as the trifluoroacetic acid salt. ^{1}H NMR (500 MHz, DMSO-d_6) δ 11.18 (s, 1H), 10.37 (s, 1H), 9.11 (d, *J* = 2.4 Hz, 1H), 8.85 (s, 1H), 8.40 (dd, *J* = 8.6, 2.4 Hz, 1H), 8.00 (d, *J* = 8.6 Hz, 1H), 7.60 (d, *J* = 8.9 Hz, 1H), 7.01 (d, *J* = 8.9 Hz, 1H), 4.48 (d, *J* = 5.5 Hz, 2H), 3.38 – 3.27 (m, 2H), 3.20 (dt, *J* = 13.9, 7.5 Hz, 2H), 2.65 (s, 3H), 1.84 (dt, *J* = 14.6, 6.9 Hz, 2H), 1.72 (dd, *J* = 15.5, 9.1 Hz, 2H), 1.56 (ddt, *J* = 16.5, 13.6, 9.6 Hz, 4H). ^{13}C NMR (126 MHz, DMSO-d_6) δ 164.46, 158.83, 157.99 (q, *J* = 33.7 Hz), 153.83, 147.19, 141.65, 141.35 (q, *J* = 34.3 Hz), 138.26, 127.86, 126.56, 121.71, (q, *J* = 273.9 Hz), 121.40 (q, *J* = 3.0 Hz), 116.42 (q, *J* = 296.1 Hz), 113.59, 113.52, 113.06, 107.39, 53.76, 51.53, 26.66, 22.48, 14.20. HRMS for $C_{23}H_{24}F_3N_3O_3$ $[M+H]^+$ calc: 448.18425, found: 448.18518 (error = -1.79 ppm).

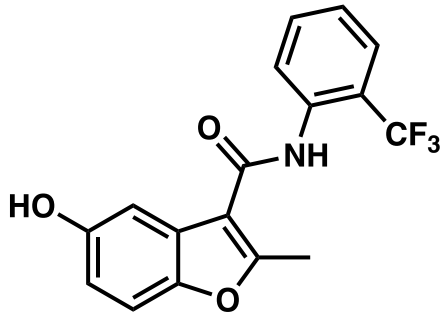

5-hydroxy-2-methyl-*N*-(2-(trifluoromethyl)phenyl)benzofuran-3-carboxamide. (10) The 3-oxo-*N*-(2-(trifluoromethyl)phenyl)butanamide (40 mg, 0.16 mmol, 1 eq), p-benzoquinone (18 mg, 0.16 mmol, 1 eq), and In(OTf)$_3$ (5 mg, 8.2 μmol, 5 mol %) was added to a solution of acetonitrile (7 mL). The solution was stirred at room temperature for 16 h. The solvent was removed under reduced pressure. The residue was purified by chromatography on silica gel to afford the desired product (26 mg, 48%). A small amount of HPLC purified product was used for analytical characterization. ^{1}H NMR (500 MHz, DMSO-d_6) δ 9.70 (s, 1H), 9.29 (s, 1H), 7.81 (dd, *J* = 7.9, 1.4 Hz, 1H), 7.75 (t, *J* = 7.7, 1H), 7.60 (d, *J* = 7.9 Hz, 1H), 7.54 (t, *J* = 7.7 Hz, 1H), 7.37 (d, *J* = 8.8 Hz, 1H), 7.14 (d, *J* = 2.5 Hz, 1H), 6.75 (dd, *J* = 8.8, 2.5 Hz, 1H), 6.53 (s, 1H), 2.65 (s, 3H). ^{13}C NMR (126 MHz, DMSO-d_6) δ 163.20, 158.66, 153.75, 146.99, 135.57, 133.13, 130.75, 127.23,

126.87, 126.45 (q, J = 5.2 Hz), 125.95 (q, J = 29.1 Hz), 123.73 (q, J = 273.4 Hz), 112.75, 112.44, 111.09, 105.30, 13.71. HRMS for $C_{17}H_{12}F_3NO_3$ $[M+H]^+$ Calc: 336.08420, found: 336.08459 (error = -0.97 ppm).

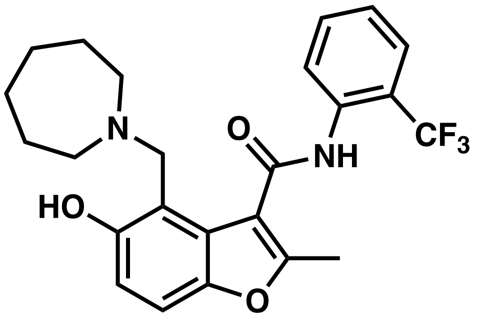

4-(azepan-1-ylmethyl)-5-hydroxy-2-methyl-*N*-(2-(trifluoromethyl)phenyl)benzofuran-3-carboxamide. (11) A solution of 5-hydroxy-2-methyl-*N*-(2-(trifluoromethyl)phenyl)benzofuran-3-carboxamide (37 mg, 0.110 mmol,1 eq), 37% aq. formaldihyde (18 µL, 0.221 mmol, 2 eq), and azepane (18 µL, 0.221 mmol, 2 eq) in EtOH (3 mL) was heated to 70 °C for 6 h. After the reaction was cooled to room temperature, the solvent was removed under reduced pressure. The residue was purified by chromatography on silica gel to afford the desired product (65%, 32 mg). A small amount of HPLC purified product was used for analytical characterization as the trifluoroacetic acid salt. ^{1}H NMR (500 MHz, DMSO-d_6) δ 10.55 (s, 1H), 10.29 (s, 1H), 7.87 (d, J = 7.8 Hz, 1H), 7.81 (t, J = 7.7 Hz, 1H), 7.74 (d, J = 7.8 Hz, 1H), 7.62 (t, J = 7.7 Hz, 1H), 7.58 (d, J = 8.9 Hz, 1H), 6.99 (d, J = 8.9 Hz, 1H), 4.48 (d, J = 5.6 Hz, 2H), 3.23 (dtd, J = 20.3, 6.9, 3.8 Hz, 3H), 2.72 (s, 3H), 1.89 – 1.76 (m, 2H), 1.74 – 1.62 (m, 2H), 1.54 (p, J = 3.0 Hz, 4H). ^{13}C NMR (126 MHz, DMSO-d_6) δ 165.54, 158.25, 157.85 (q, J = 32.2 Hz), 153.76, 147.18, 134.72, 133.52, 131.19, 128.22, 126.79, 126.66 (d, J = 5.2 Hz), 126.25 (q, J = 29.6 Hz), 123.62 (q, J = 273.1 Hz), 116.79 (q, J = 298.0 Hz), 113.59, 113.13, 113.05, 107.28, 53.45, 51.44, 26.46, 22.63, 13.82. HRMS for $C_{24}H_{25}F_3N_2O_3$ $[M+H]^+$ calc: 447.18900 found: 447.18980 (error = -1.58 ppm).

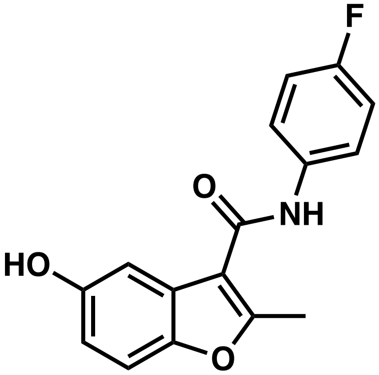

***N*-(4-fluorophenyl)-5-hydroxy-2-methylbenzofuran-3-carboxamide. (12)** The *N*-(4-fluorophenyl)-3-oxobutanamide (374 mg, 1.92 mmol, 1 eq), p-benzoquinone (207 mg, 1.92 mmol, 1 eq), and $In(OTf)_3$ (54 mg, 96 µmol, 5 mol %) was added to a solution of acetonitrile (12 mL). The solution was stirred at room temperature for 16 h. The solvent was removed under reduced pressure. The residue was purified by chromatography on silica gel to afford the desired product (268 mg, 49%). A small amount of HPLC purified product was used for analytical characterization. ^{1}H NMR (500 MHz, DMSO-d_6) δ 10.07 (s, 1H), 9.30 (s, 1H), 7.78 – 7.68 (m, 2H), 7.37 (d, J = 8.8 Hz, 1H), 7.25 – 7.16 (m, 2H),

7.05 (d, *J* = 2.4 Hz, 1H), 6.74 (dd, *J* = 8.8, 2.5 Hz, 1H), 2.60 (s, 3H). ^{13}C NMR (126 MHz, DMSO-d_6) δ 161.94, 158.22 (q, *J* = 240.1 Hz), 158.15, 153.72, 146.98, 135.43 (d, *J* = 2.6 Hz), 126.79, 121.82 (d, *J* = 7.9 Hz), 115.25 (d, *J* = 22.4 Hz), 113.30, 112.72, 111.17, 105.17, 13.77. HRMS for $C_{16}H_{12}FN_2O_3$ $[M+H]^+$ calc: 286.08740, found: 286.08778 (error = -1.04 ppm).

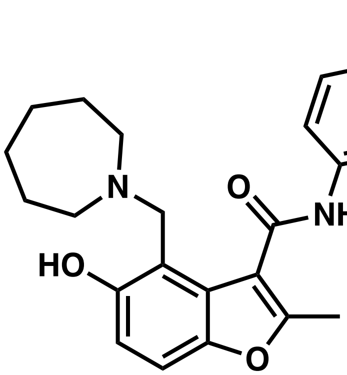

4-(azepan-1-ylmethyl)-*N*-(4-fluorophenyl)-5-hydroxy-2-methylbenzofuran-3-carboxamide. (13) A solution of *N*-(4-fluorophenyl)-5-hydroxy-2-methylbenzofuran-3-carboxamide (230 mg, 0.807 mmol,1 eq), 37% aq. formaldihyde (131 µL, 1.61 mmol, 2 eq), and azepane (182 µL, 1.61 mmol, 2 eq) in EtOH (5 mL) was heated to 70 °C for 6 h. After the reaction was cooled to room temperature, the solvent was removed under reduced pressure. The residue was purified by chromatography on silica gel to afford the desired product (54%, 173 mg). A small amount of HPLC purified product was used for analytical characterization as the trifluoroacetic acid salt. ^{1}H NMR (500 MHz, DMSO-d_6) δ 10.79 (s, 1H), 10.52 (s, 1H), 9.05 (s, 1H), 7.78 (dd, *J* = 8.9, 5.0 Hz, 2H), 7.57 (d, *J* = 8.9 Hz, 1H), 7.26 (t, *J* = 8.8 Hz, 2H), 7.02 (d, *J* = 8.9 Hz, 1H), 4.48 (d, *J* = 4.6 Hz, 2H), 3.33 – 3.25 (m, 2H), 3.25 – 3.15 (m, 2H), 2.61 (s, 3H), 1.80 (m, 2H), 1.77 – 1.69 (m, 3H), 1.55 (m, 4H). ^{13}C NMR (126 MHz, DMSO-d_6) δ 163.55, 159.69, 157.98 (q, *J* = 32.2), 157.81 (d, *J* = 9.2 Hz), 153.81, 147.15, 134.87 (d, *J* = 2.7 Hz), 126.69, 122.11 (d, *J* = 8.0 Hz), 117.26 (q, *J* = 300.2 Hz), 115.56 (d, *J* = 22.3 Hz), 113.98, 113.47, 112.95, 107.25, 53.66, 51.59, 26.60, 22.58, 14.02. HRMS for $C_{23}H_{25}FN_2O_3$ $[M+H]^+$ calc: 397.18492, found: 397.19275 (error = -0.94 ppm).

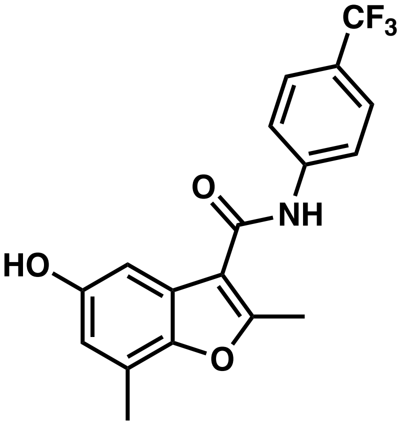

5-hydroxy-2,7-dimethyl-*N*-(4-(trifluoromethyl)phenyl)benzofuran-3-carboxamide. (14) 3-oxo-*N*-(4-(trifluoromethyl)phenyl)butanamide (230 mg, 0.939 mmol, 1 eq) was added with 2-methyl-p-benzoquinone (115 mg, 0.939 mmol, 1 eq) and $In(OTf)_3$ (26 mg, 47 µmol, 5 mol %) to a solution of acetonitrile (20 mL). The solution was stirred at room

temperature for 16 h. The solvent was removed under reduced pressure. The residue was purified by chromatography on silica gel to afford the desired product (73 mg, 27%). A small amount of HPLC purified product was used for analytical characterization. ^{1}H NMR (400 MHz, DMSO-d_6) δ 10.34 (s, 1H), 9.19 (s, 1H), 7.94 (d, *J* = 8.2 Hz, 2H), 7.72 (d, *J* = 8.5 Hz, 2H), 6.90 – 6.84 (m, 1H), 6.59 (dd, *J* = 2.4, 1.0 Hz, 1H), 2.62 (s, 3H), 2.39 (s, 3H). ^{13}C NMR (126 MHz, DMSO-d_6) δ 162.59, 158.39, 153.72, 146.10, 142.73, 126.06, 126.00 (q, *J* = 4.2 Hz), 124.42 (q, *J* = 271.6 Hz), 123.50 (q, *J* = 31.8 Hz), 120.99, 119.81, 113.79, 113.37, 102.65, 14.79, 13.89. HRMS for $C_{18}H_{14}F_3NO_3$ $[M+H]^+$ calc: 350.09985, found: 350.10021 (error = -0.98 ppm).

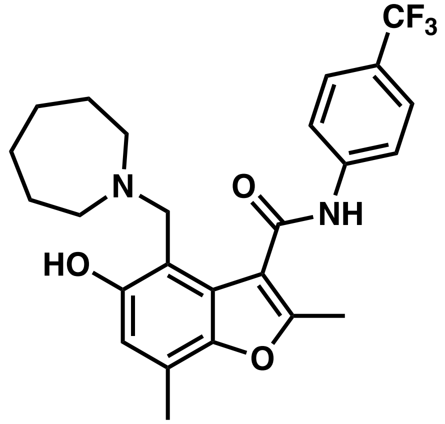

4-(azepan-1-ylmethyl)-5-hydroxy-2,7-dimethyl-*N*-(4-(trifluoromethyl)phenyl)benzofuran-3-carboxamide. (15) To a solution of 5-hydroxy-2,7-dimethyl-*N*-(4-(trifluoromethyl)phenyl)benzofuran-3-carboxamide (125 mg, 0.36 mmol,1 eq), 37% aq. formaldihyde (58 μL, 0.72 mmol, 2 eq), and azepane (80 μL, 0.72 mmol, 2 eq) in EtOH (3 mL) was heated to 70 °C for 6 h. After the reaction was cooled to room temperature, the solvent was removed under reduced pressure. The residue was purified by chromatography on silica gel to afford the desired product (61%, 101 mg). A small amount of HPLC purified product was used for analytical characterization as the trifluoroacetic acid salt. ^{1}H NMR (500 MHz, DMSO-d_6) δ 10.98 (s, 1H), 10.25 (s, 1H), 8.88 (s, 1H), 7.96 (d, *J* = 8.4 Hz, 2H), 7.79 (d, *J* = 8.6 Hz, 2H), 6.83 (s, 1H), 4.44 (d, *J* = 5.5 Hz, 2H), 3.34 – 3.24 (m, 2H), 3.17 (ddd, *J* = 14.3, 10.1, 4.4 Hz, 2H), 2.62 (s, 3H), 2.44 (s, 3H), 1.82 (dt, *J* = 14.8, 7.1 Hz, 2H), 1.71 (dd, *J* = 16.1, 8.8 Hz, 2H), 1.64 – 1.47 (m, 4H). ^{13}C NMR (126 MHz, DMSO-d_6) δ 164.22, 158.00 (q, *J* = 33.4 Hz), 157.89, 153.66, 146.23, 142.09, 126.25 (q, *J* = 3.9 Hz), 126.01, 124.37 (q, *J* = 32.1 Hz), 124.29 (q, J = 271.6 Hz), 123.61, 120.10, 116.50 (q, *J* = 296.3 Hz), 114.10, 113.76, 104.79, 53.57, 51.50, 26.63, 22.53, 14.87, 14.14. HRMS for $C_{25}H_{27}F_3N_2O_3$ $[M+H]^+$ calc: 461.20465, found: 461.20570 (error = -1.91 ppm).

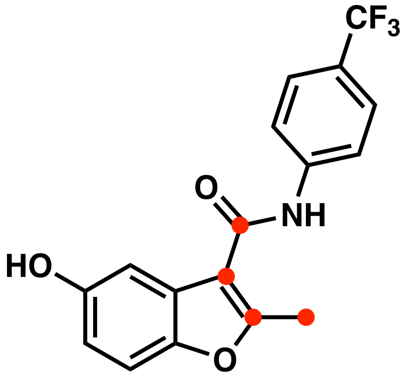

4-^{13}C-labeled 5-hydroxy-2-methyl-*N*-(4-(trifluoromethyl)phenyl)benzofuran-3-carboxamide. (16) Red dots in structure indicate a uniformly ^{13}C labeled carbon. 4-^{13}C-labeled ethyl acetoacetate (100 µL, 0.762 mmol, 1 eq), analine (96 µL, 0.762 mmol, 1eq), and DMAP (5 mg, 38 µmol, 5 mol%) was added to a solution of toluene (1.5 mL) were heated to reflux and stirred for 14 hours. Then the reaction was cooled to room temperature. The solvent was removed under reduced pressure. The orange solid was purified by triteration with cold toluene to afford the desired product, a white powder (91 mg mmol, 1eq). The white powder was then added to p-benzoquinone (39 mg, 0.365 mmol, 1 eq), and In(OTf)$_3$ (10 mg, 18 µmol, 5 mol %) in a solution of acetonitrile (5 mL) without further purification. The solution was stirred at room temperature for 16 h. The solvent was removed under reduced pressure. The residue was purified by chromatography on silica gel to afford the desired product (68 mg, 26% over two steps). A small amount of HPLC purified product was used for analytical characterization. ^{1}H NMR (500 MHz, DMSO-d_6) δ 10.38 (d, J = 2.6 Hz, 1H), 9.32 (s, 1H), 7.94 (d, J = 8.5 Hz, 2H), 7.73 (d, J = 8.5 Hz, 2H), 7.39 (d, J = 8.7 Hz, 1H), 7.05 (t, J = 2.4 Hz, 1H), 6.75 (dd, J = 8.8, 2.5 Hz, 1H), 2.61 (ddd, J = 129.4, 7.2, 3.1 Hz, 3H). ^{13}C NMR (126 MHz, DMSO-d_6) δ 162.44 (dd, J = 75.1, 7.1 Hz), 158.73 (ddd, J = 74.3, 52.0, 7.0 Hz), 153.80 (d, J = 4.5 Hz), 146.98, 142.71, 126.65 (d, J = 55.0 Hz), 126.01 (q, J = 3.9 Hz), 124.41 (q, J = 271.2 Hz), 123.51 (q, J = 32.1 Hz), 119.79 (d, J = 2.1 Hz), 113.09 (td, J = 74.6, 4.2 Hz), 112.84, 111.26 (d, J = 2.5 Hz), 105.15 (d, J = 4.2 Hz), 13.81 (dd, J = 52.1, 4.3 Hz). HRMS for $C_{17}H_{12}F_3NO_3$ $[M+H]^+$ Calc: 340.09817, found: 340.09802 (error = -0.44 ppm).

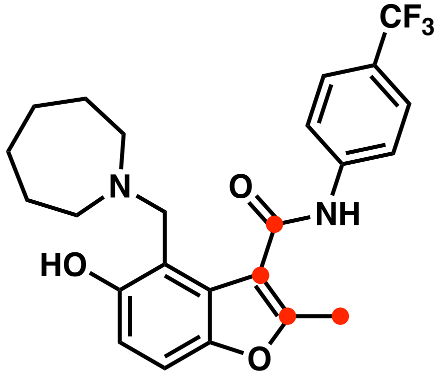

4-^{13}C-labeled 4-(azepan-1-ylmethyl)-5-hydroxy-2-methyl-*N*-(4-(trifluoromethyl)phenyl)benzofuran-3-carboxamide. (17) Red dots in structure indicate a uniformly ^{13}C labeled carbon. To a solution of 4-^{13}C-labeled 5-hydroxy-2-methyl-*N*-(4-(trifluoromethyl)phenyl)benzofuran-3-carboxamide (35 mg, 0.103 mmol,1

eq), 37% aq. formaldihyde (17 µL, 0.206 mmol, 2 eq), and azepane (23 µL, 0.206 mmol, 2 eq) in EtOH (2 mL) was heated to 70 °C for 16 h. After the reaction was cooled to room temperature, the solvent was removed under reduced pressure. The residue was purified by chromatography on silica gel to afford the desired product. (62%, 29 mg). ^{1}H NMR (500 MHz, DMSO-d_6) δ 11.01 (s, 1H), 10.76 (d, *J* = 2.5 Hz, 1H), 7.96 (d, *J* = 8.4 Hz, 2H), 7.73 (d, *J* = 8.6 Hz, 2H), 7.30 (d, *J* = 8.8 Hz, 1H), 6.74 (d, *J* = 8.8 Hz, 1H), 3.87 (s, 2H), , 2.52 – 2.51 (m, 4H), 2.48 (ddd, *J* = 129.5, 7.2, 3.4 Hz, 3H), 1.44 (s, 8H). ^{13}C NMR (126 MHz, DMSO-d_6) δ 163.36 (dd, *J* = 74.6, 6.7 Hz), 155.37 (ddd, *J* = 73.3, 52.9, 6.6 Hz), 153.80 (d, *J* = 4.3 Hz), 147.04, 142.77, 126.11 (q, *J* = 3.8 Hz), 125.40 (q, *J* = 55.5 Hz), 124.40 (*J* = 271.1 Hz), 123.50 (q, *J* = 32.0 Hz), 114.79 (td, *J* = 73.9, 4.9 Hz), 114.10 (d, *J* = 5.4 Hz), 113.03, 109.88 (t, *J* = 2.6 Hz), 54.25, 54.21, 26.99, 26.13, 13.28 (dd, *J* = 53.1, 5.0 Hz). HRMS for $C_{24}H_{25}F_3N_2O_3$ $[M+H]^+$ calc 451.20297, found: 451.20305 (error = 0.18 ppm).

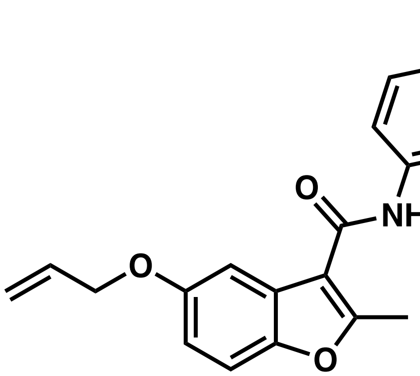

5-(allyloxy)-2-methyl-*N*-(4-(trifluoromethyl)phenyl)benzofuran-3-carboxamide. (18) To a solution of hydroxybenzoquinone (224 mg, 667 µmol, 1 equiv.) in DMF (3.3 mL, 0.2M) was added potassium carbonate (138 mg, 1.00 mmol, 1.5 equiv.) and allyl bromide (60.6 µL, 701 µmol, 1.05 equiv), and the resulting suspension was stirred overnight. The reaction mixture was then diluted in ethyl acetate (100 mL), washed with Brine (5 x 50 mL), dried over Na_2SO_4, concentrated *in vacuo*, and the residue was purified by flash chromatography (4 g column, 0% to 50% EtOAc/Hexanes) to afford the title compound (182 mg, 485 µmol, 73% yield) as a colorless solid. ^{1}H NMR (400 MHz, Chloroform-*d*) δ 7.75 (d, *J* = 8.3 Hz, 2H), 7.69 (s, 1H), 7.64 (d, *J* = 8.6 Hz, 2H), 7.38 (d, *J* = 9.0 Hz, 1H), 7.17 (d, *J* = 2.5 Hz, 1H), 6.93 (dd, *J* = 8.9, 2.5 Hz, 1H), 6.08 (ddt, *J* = 17.2, 10.6, 5.3 Hz, 1H), 5.44 (dq, *J* = 17.3, 1.6 Hz, 1H), 5.32 (dq, *J* = 10.5, 1.4 Hz, 1H), 4.58 (dt, *J* = 5.4, 1.5 Hz, 2H), 2.73 (s, 3H). ^{13}C NMR (101 MHz, $CDCl_3$) δ 162.5, 162.0, 155.9, 148.8, 141.0, 141.0, 133.4, 126.6 (q, *J* = 3.7 Hz), 126.4 (q, *J* = 31.5 Hz), 125.9, 124.2 (q, *J* = 271.6 Hz), 119.8, 118.0, 113.1, 112.2, 104.1, 70.0, 14.4. HRMS for $C_{20}H_{17}F_3NO_3$ $[M+H]^+$ calc. 376.11550, found 376.11759 (error = -4.68).

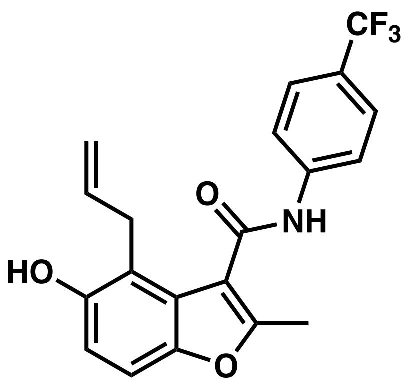

4-allyl-5-hydroxy-2-methyl-*N*-(4-(trifluoromethyl)phenyl)benzofuran-3-carboxamide. **(19)** To a flask containing the allyl ether (528 mg, 1.41 mmol) under argon was added N,N-dimethylaniline (14 mL, 0.1 M). The flask was attached to an argon balloon and heated in an oil bath to 185°C for 25h. The reaction mixture was cooled to room temperature, diluted with EtOAc (200 mL), washed with aq. 1 M HCl (4 x 100 mL), brine (100 mL) and dried (Na_2SO_4). The organic solution was then filtered, concentrated onto silica gel, and purified by automated flash chromatography (12 g column, Hexanes to 50% EtOAc/Hexanes) to afford the title compound (380 mg, 1.01 mmol, 72% yield) as a brown oil and used directly without further purification. A small amount of HPLC purified product was used for analytical characterization. ^{1}H NMR (500 MHz, DMSO-d_6) δ 10.80 (s, 1H), 9.16 (s, 1H), 7.94 (d, *J* = 8.5 Hz, 2H), 7.73 (d, *J* = 8.5 Hz, 2H), 7.25 (d, *J* = 8.8 Hz, 1H), 6.84 (d, *J* = 8.8 Hz, 1H), 5.78 (ddt, *J* = 16.6, 10.0, 6.3 Hz, 1H), 4.89 – 4.63 (m, 2H), 3.57 (d, *J* = 6.4 Hz, 2H), 2.48 (s, 3H). ^{13}C NMR (126 MHz, DMSO) δ 164.0, 155.6, 151.5, 147.7, 143.1, 137.2, 126.6 (q, *J* = 3.8 Hz), 126.2, 124.8 (q, *J* = 271.2 Hz), 124.1 (q, *J* = 32.0 Hz), 119.8, 117.4, 115.3, 115.1, 113.1, 109.2, 29.9, 13.8. HRMS for $C_{20}H_{17}F_3NO_3$ $[M+H]^+$ calc. 376.11550, found 376.11649 (error = -2.13).

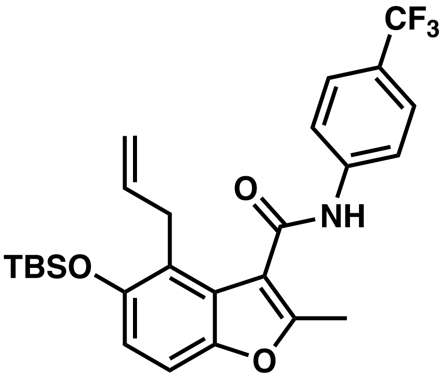

4-allyl-5-((*tert*-butyldimethylsilyl)oxy)-2-methyl-*N*-(4-(trifluoromethyl)phenyl)benzofuran-3-carboxamide. **(20)** To a solution of the hydroxybenzofuran (51 mg, 0.153 mmol, 1 equiv.) in DMF (1.5 mL, 0.1 M) was added imidazole (42 mg, 0.613 mmol, 4 equiv.) and TBSCl (46 mg, 0.307 mmol, 2 equiv.). The resulting solution was stirred at room temperature for 3.5 h, then diluted in EtOAc (30 mL), washed with brine (3 x 10 mL), dried (Na_2SO_4), and concentrated onto silica gel. Purification by flash chromatography (4 g column, Hexanes to CH_2Cl_2) afforded the title compound (25 mg, 0.0502 mmol, 33% yield). ^{1}H NMR (500 MHz, Chloroform-*d*) δ 7.82 (s, 1H), 7.73 (d, *J* = 8.4 Hz, 2H), 7.61 (d, *J* = 10.0 Hz, 1H), 7.22 (d, *J* = 6.7 Hz, 1H), 6.83 (d, *J* = 8.7 Hz, 0H), 6.17 – 5.91 (m, 1H), 5.04 (d, *J* = 9.5 Hz, 1H), 4.76 (d, *J* = 17.3 Hz, 1H), 3.63 (s, 2H), 2.57 (s, 3H), 0.99 (s, 9H), 0.22 (s, 6H). ^{13}C NMR (126 MHz, $CDCl_3$) δ 163.7, 158.3, 150.2, 149.0, 140.9, 138.2, 126.5 (q, *J* = 3.8 Hz), 126.3 (q, *J* = 32.7 Hz),

125.7, 124.2 (q, *J* = 271.6 Hz), 120.6, 119.6, 116.0, 115.3, 114.5, 109.5, 31.0, 25.9, 18.4, 13.7, -4.0. HRMS for $C_{26}H_{31}F_3NO_3Si$ [M+H]$^+$ calc. 490.20198, found 490.20257 (error = -0.89).

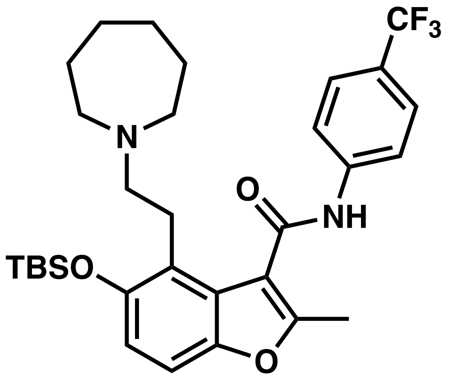

4-(2-(azepan-1-yl)ethyl)-5-((*tert*-butyldimethylsilyl)oxy)-2-methyl-*N*-(4-(trifluoromethyl)phenyl)benzofuran-3-carboxamide. **(21)** To a suspension of the allyl benzofuran (50 mg, 102 μmol, 1 equiv.) in *tert*-butanol (1.3 mL) was added THF (530 μL), H_2O (140 μL), *N*-methylmorpholine-*N*-oxide (14 mg, 122 μmol, 1.2 equiv.) and osmium tetroxide (2.5 wt% in *t*BuOH, 51.9 μL, 5.11 μmol, 5 mol%). The resulting solution was allowed to stir at room temperature for 12 h. To the reaction mixture was added sodium periodate (66 mg, 306 μmol, 3 equiv.) as a solution in H_2O (165 μL), and the solution was stirred for an additional 75 min, after which the reaction was quenched with 10% aqueous $NaHSO_3$ (10 mL) and sat. aqueous $NaHCO_3$ and extracted with ethyl acetate (3 x 10 mL). The combined organic layers were washed with brine (10 mL), dried over Na_2SO_4, and concentrated *in vacuo* to give 53 mg of a yellowish solid. This solid was dissolved in CH_2Cl_2 (1 mL) and methanol (1 mL) and hexamethyleneimine (23.0 μL, 204 μmol, 2 equiv,) was added and the reaction was stirred overnight. To the reaction solution was added sodium triacetoxyborohidride (43 mg, 204 μmol, 2 equiv.) and stirred for an additional hour, after which the mixture was concentrated *in vacuo* and partitioned between ethyl acetate (10 mL) and 2 M aqueous Na_2CO_3 (10 mL). The organic layer was dried over Na_2SO_4 and concentrated *in vacuo*, and the residue purified by flash chromatography (4 g column, 0% to 50% EtOAc/Hexanes) to afford the title compound (29 mg, 50.5 μmol, 49% over 2 steps) as a colorless solid. ^{1}H NMR (500 MHz, Chloroform-*d*) δ 9.68 (s, 1H), 7.76 (d, *J* = 9.3 Hz, 2H), 7.61 (d, *J* = 8.3 Hz, 2H), 7.16 (d, *J* = 8.6 Hz, 1H), 6.77 (d, *J* = 8.6 Hz, 1H), 3.19 (t, *J* = 7.2 Hz, 2H), 2.85 (t, *J* = 7.4 Hz, 2H), 2.58 (s, 6H), 1.50 – 1.34 (m, 6H), 1.02 (s, 9H), 0.24 (s, 6H). ^{13}C NMR (126 MHz, $CDCl_3$) δ 164.4, 158.0, 150.0, 149.0, 141.4, 126.7 (q, *J* = 32.7 Hz), 126.3 (q, *J* = 3.7 Hz), 126.1, 124.2 (q, *J* = 271.7 Hz), 121.2, 115.9, 114.8, 109.2, 57.3, 56.3, 26.9, 26.1, 18.5, 14.0, -3.8. HRMS for $C_{31}H_{42}F_3N_2O_3Si$ [M+H]$^+$ calc. 575.29113, found 575.29115 (error = 1.02).

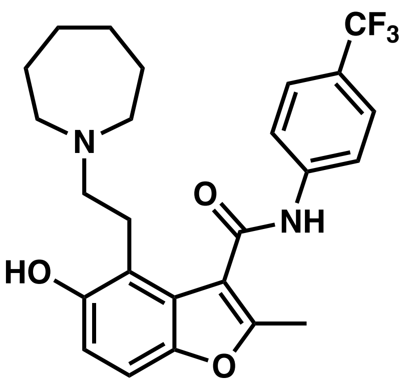

4-(2-(azepan-1-yl)ethyl)-5-hydroxy-2-methyl-*N*-(4-(trifluoromethyl)phenyl)benzofuran-3-carboxamide. **(22)** To a solution of the silyl ether (20 mg, 34.8 µmol, 1 equiv.) in THF was added acetic acid (4.0 µL, 69.6 µmol, 2 equiv.) and tetrabutylammonium fluoride (1 M in THF, 70 µL, 69.6 µmol, 2 equiv.) and the resulting solution was stirred for 1 h. The mixture was concentrated *in vacuo*, and the residue dissolved in methanol and purified by reverse-phase HPLC (25% to 95% CH_3CN/H_2O + 0.1% TFA, 10 min gradient). The resulting fractions were combined and lyophilized to afford the product (19.8 mg, 34.5 µmol, 99% yield) as a slightly brown solid. ^1H NMR (500 MHz, Methanol-d_4) δ 7.93 (d, J = 8.3 Hz, 2H), 7.71 (d, J = 8.5 Hz, 2H), 7.27 (d, J = 8.8 Hz, 1H), 6.85 (d, J = 8.8 Hz, 1H), 3.50 – 3.38 (m, 4H), 3.27 – 3.19 (m, 4H), 2.59 (s, 3H), 1.93 – 1.82 (m, 2H), 1.82 – 1.62 (m, 6H). ^{13}C NMR (126 MHz, MeOD) δ 166.6, 162.9 (d, J = 36.0 Hz), 158.2, 153.2, 149.7, 143.2, 127.5 – 127.2 (m), 125.6 (d, J = 270.8 Hz), 121.2, 118.2 (d, J = 290.8 Hz),115.2, 114.1, 113.6, 111.4, 57.8, 56.2, 27.3, 24.9, 23.5, 13.7. HRMS for $C_{25}H_{28}F_3N_2O_3$ $[M+H]^+$ calc. 461.20465, found 461.20509 (error = -0.60).

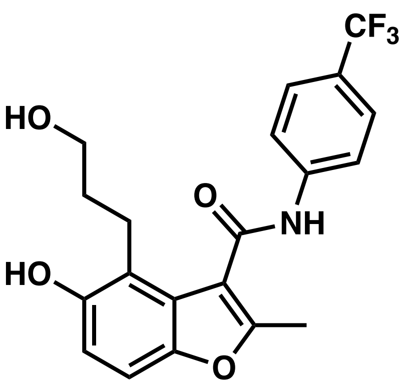

5-hydroxy-4-(3-hydroxypropyl)-2-methyl-*N*-(4-(trifluoromethyl)phenyl)benzofuran-3-carboxamide. **(23)** To an ice cooled solution of the allyl benzofuran (117 mg, 311 µmol, 1 equiv.) in THF (4.6 mL) under argon atmosphere was added $BH_3{\cdot}SMe_2$ (2M in THF, 1.55 mL, 10 equiv.). The solution was warmed to room temperature and stirred overnight, after which it was cooled to 0°C and hydrogen peroxide (30 wt% in H_2O, 1.4 mL, 40 equiv.) and NaOH (3M in H2O, 1.4 mL, 40 equiv) were added sequentially. The solution was stirred for 4 h at 0°C, then quenched with aqueous 5% citric acid (30 mL) and extracted with ethyl acetate (3 x 30 mL). The combined organic layers were washed with brine (30 mL), dried (Na_2SO_4), and concentrated *in vacuo* and the residue was purified by flash chromatography (4 g, 0% to 100% EtOAc/Hexanes) to afford the title compound (44 mg, 112 µmol, 36% yield). ^1H NMR (500 MHz, DMSO-d_6) δ 10.85 (s, 1H), 9.04 (s, 1H), 7.94 (d, J = 8.4 Hz, 2H), 7.73 (d, J = 8.4 Hz, 2H), 7.20 (d, J = 8.7 Hz, 1H), 6.80 (d, J = 8.7 Hz, 1H), 4.29 (t, J = 5.2 Hz,

1H), 3.21 (td, *J* = 6.9, 5.1 Hz, 2H), 2.79 – 2.68 (m, 2H), 2.46 (s, 3H), 1.69 – 1.51 (m, 3H). ^{13}C NMR (126 MHz, DMSO) δ 163.7, 154.6, 150.9, 147.2, 142.6, 126.1 (q, *J* = 3.7 Hz), 125.7, 124.4 (q, *J* = 271.4 Hz), 123.7 (q, *J* = 32.1 Hz), 119.7, 119.4, 114.9, 112.5, 108.2, 60.9, 32.8, 22.4, 13.2. HRMS for $C_{20}H_{19}F_3NO_4$ $[M+H]^+$ calc. 394.12607, found 394.12631 (error = -0.46).

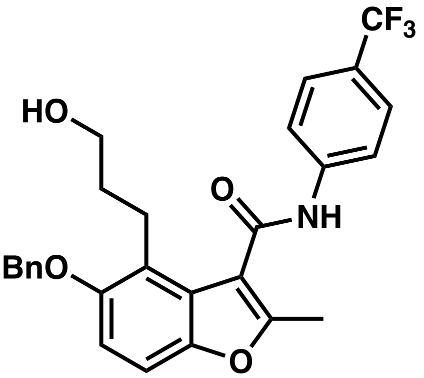

5-(benzyloxy)-4-(3-hydroxypropyl)-2-methyl-*N*-(4-(trifluoromethyl)phenyl)benzofuran-3-carboxamide. **(24)** To a solution of the hydroxybenzofuran (44 mg, 112 µmol, 1 equiv.) in DMF (1 mL) was added potassium carbonate (39 mg, 280 µmol, 2.5 equiv.) and benzyl bromide (14.6 µL, 123 µmol, 1.1 equiv.) and the reaction mixture was stirred for 3 days at room temperature. The reaction was diluted in ethyl acetate (30 mL), washed with brine (3 x 10 mL), dried (Na_2SO_4), and concentrated *in vacuo* and the residue was purified by flash chromatography (4 g column, 0% to 70% EtOAc/Hexanes) to afford the title compound (25 mg, 51.7 µmol, 46% yield) as a colorless solid. ^{1}H NMR (500 MHz, DMSO-d_6) δ 7.95 (d, *J* = 8.3 Hz, 2H), 7.74 (d, *J* = 8.5 Hz, 2H), 7.48 (d, *J* = 6.9 Hz, 2H), 7.43 – 7.34 (m, 3H), 7.31 (t, *J* = 7.3 Hz, 1H), 7.09 (d, *J* = 9.0 Hz, 1H), 5.14 (s, 2H), 3.22 (t, *J* = 6.9 Hz, 2H), 2.95 – 2.78 (m, 2H), 2.49 (s, 3H), 1.64 (dq, *J* = 9.9, 7.0 Hz, 2H). ^{13}C NMR (126 MHz, DMSO) δ 163.4, 155.4, 152.1, 148.2, 142.4, 137.6, 128.4, 127.6, 127.2, 126.16 (q, *J* = 3.8 Hz), 125.8, 124.37 (q, *J* = 271.3 Hz), 123.76 (q, *J* = 32.1 Hz), 122.9, 119.4, 114.8, 110.7, 108.4, 70.7, 60.9, 33.2, 22.6, 13.2. HRMS for $C_{27}H_{25}F_3NO_4$ $[M+H]^+$ calc. 484.17302, found 484.17385 (error = -1.33).

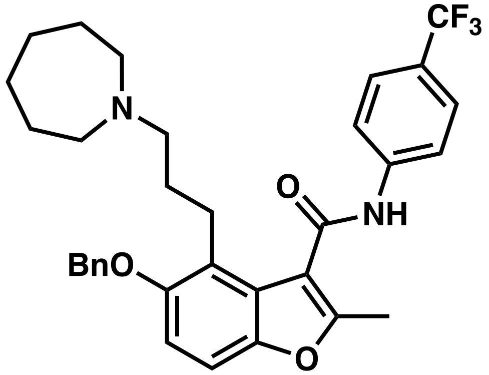

4-(3-(azepan-1-yl)propyl)-5-(benzyloxy)-2-methyl-*N*-(4-(trifluoromethyl)phenyl)benzofuran-3-carboxamide. **(25)** To a suspension of the hydroxypropylbenzofuran (20 mg, 41.4µmol, 1 equiv.) in CH_2Cl_2 (1 mL) was added Dess-Martin periodinane (36 mg, 85.2 µmol, 2.5 equiv.). The resulting mixture was stirred for 40 min, then diluted in ethyl acetate (20 mL), washed with a 1:1 mixture of 5% aqueous $Na_2S_2O_3$ and saturated aqueous $NaHCO_3$ (3 x 10 mL). The organic layer was washed further with brine (10 mL), dried over Na_2SO_4, and

concentrated *in vacuo* to obtain 22 mg of brown solid which was used without further purification. The obtained solid was redissolved in CH_2Cl_2 (1 mL) and methanol (1 mL) and to this solution was added hexamethyleneimine (9.32 µL, 82.7 µmol, 2 equiv.) and the solution was stirred overnight. Sodium triacetoxyborohydride was then added and the solution was stirred for an additional 2.5 h, after which it was partitioned between 2M aqueous Na_2CO_3 (20 mL) and ethyl acetate (10 mL). The aqueous layer was further extracted with ethyl acetate (2 x 10 mL) and the combined organic layers were washed with brine (10 mL), dried over Na_2SO_4, concentrated *in vacuo*, and the resulting residue was purified by flash chromatography (4 g column, Hexanes to EtOAc) to obtain the title compound (10.2 mg, 18.1 µmol, 44% yield) as a brown solid. ^{1}H NMR (400 MHz, Methanol-d_4) δ 7.92 (d, J = 8.4 Hz, 2H), 7.71 (d, J = 8.6 Hz, 2H), 7.52 – 7.45 (m, 2H), 7.43 – 7.30 (m, 4H), 7.13 (d, J = 9.0 Hz, 1H), 5.14 (s, 2H), 3.26 (ddd, J = 13.6, 7.5, 2.8 Hz, 2H), 3.08 – 2.88 (m, 6H), 2.57 (s, 3H), 2.10 – 1.96 (m, 2H), 1.82 – 1.51 (m, 8H). ^{13}C NMR (101 MHz, MeOD) δ 166.5, 158.0, 154.4, 150.5, 143.3, 138.9, 129.7, 129.1, 128.9, 127.4 (q, J = 3.5 Hz), 127.3 (q, J = 32.5 Hz), 125.6 (q, J = 270.7 Hz), 121.8, 121.2, 115.6, 111.7, 110.4, 72.7, 58.4, 55.8, 27.3, 25.6, 24.7, 24.5, 18.5, 13.5. HRMS for $C_{33}H_{36}F_3N_2O_3$ $[M+H]^+$ calc. 565.26725, found 565.26692 (error = 1.03).

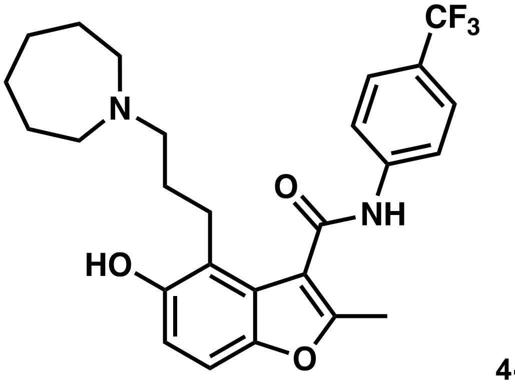

4-(3-(azepan-1-yl)propyl)-5-hydroxy-2-methyl-*N*-(4-(trifluoromethyl)phenyl)benzofuran-3-carboxamide. (26) To a solution of the benzyl ether (9.6 mg, 14.2 µmol) in methanol (1 mL) under nitrogen atmosphere was added palladium on carbon (10 wt%, 2 mg). The reaction was pressurized to 60 psi under hydrogen gas and stirred overnight. The reaction mixture was filtered through celite and purified directly by reverse-phase HPLC (10 min gradient, 10% to 75% CH_3CN/H_2O + 0.1% TFA) to obtain 3.9 mg of the title compound as a colorless solid. ^{1}H NMR (500 MHz, Methanol-d_4) δ 7.92 (d, J = 8.3 Hz, 2H), 7.71 (d, J = 8.4 Hz, 2H), 7.20 (d, J = 8.8 Hz, 1H), 6.82 (d, J = 8.8 Hz, 1H), 3.37 (ddd, J = 13.6, 7.7, 2.7 Hz, 2H), 3.15 – 3.03 (m, 4H), 2.91 (t, J = 7.6 Hz, 2H), 2.55 (s, 3H), 2.06 (qt, J = 8.1, 6.2 Hz, 2H), 1.92 – 1.57 (m, 8H). ^{13}C NMR (126 MHz, MeOD) δ 166.8, 157.3, 152.6, 149.8, 143.3, 127.4 (q, J = 3.7 Hz), 127.3 (q, J = 32.7 Hz), 127.2, 125.6 (q, J = 270.7 Hz), 121.2, 118.7, 115.6, 113.7, 110.4, 58.4, 55.9, 27.3, 25.4, 24.7, 24.3, 13.4. HRMS for C26H30F3N2O3 [M+H]+ calc. 475.22030, found 475.22080 (error = -0.44).

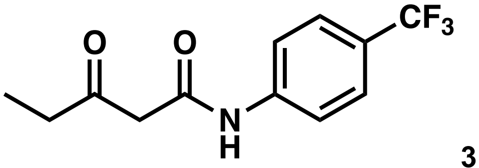

3-oxo-*N*-(4-(trifluoromethyl)phenyl)pentanamide. (27) A 20 mL scintillation vial was charged with a solution of 4-(trifluoromethyl)aniline (495 µL, 3.94 mmol, 1 equiv.) in toluene (0.75 mL, 5 M) and ethyl propionylacetate (561

μL, 3.94 mmol, 1 equiv.) and dimethylaminopyridine (24 mg, 197 μmol, 5 mol%) were added sequentially. The vial was capped tightly and the solution was heated to 110°C for 18h, after which the solution was allowed to cool to room temperature and sit for 24h to allow crystallization of the product. The solution was cooled further on ice and the crystalline product was collected by filtration, washed with cold toluene (3 x 2 mL), and dried under vacuum to obtain 245 mg (945 μmol, 24% yield) of colorless crystals. ^{1}H NMR (400 MHz, Chloroform-*d*) δ 9.51 (s, 1H), 7.68 (d, *J* = 8.4 Hz, 2H), 7.57 (d, *J* = 8.4 Hz, 2H), 3.59 (s, 2H), 2.62 (q, *J* = 7.2 Hz, 2H), 1.13 (t, *J* = 7.2 Hz, 3H). ^{19}F NMR (376 MHz, Chloroform-*d*) δ -62.16 . ^{13}C NMR (101 MHz, Chloroform-*d*) δ. ^{13}C NMR (101 MHz, $CDCl_3$) δ 208.4, 163.9, 140.7, 126.39 (q, *J* = 3.8 Hz), 126.37 (q, *J* = 32.8 Hz), 124.19 (q, *J* = 271.7 Hz), 119.8, 77.2, 48.4, 37.7, 7.5. HRMS for $C_{12}H_{13}F_3NO_2$ $[M+H]^+$ calc. 260.08929, found 260.08988 (error = -2.09).

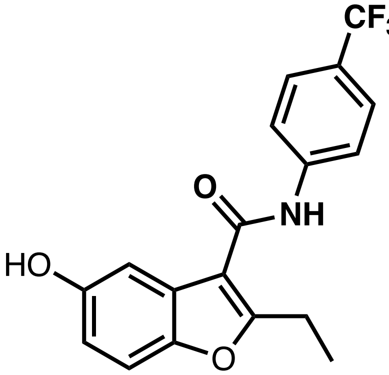

2-ethyl-5-hydroxy-N-(4-(trifluoromethyl)phenyl)benzofuran-3-carboxamide. **(28)** To a solution of the ß-ketoamide (200 mg, 772 μmol, 1 equiv.) in acetonitrile (3.9 mL, 0.2 M) was added p-benzoquinone (125 mg, 1.16 mmol, 1.5 equiv.) and indium (III) trifluoromethylsulfonate (22 mg, 38.6 μmol, 5 mol%). The solution was stirred for 18h, then concentrated directly onto silica gel and purified by flash chromatography (12 g column, 0% to 50% EtOAc/Hexane) to obtain 74 mg (212 μmol, 27% yield) of product. ^{1}H NMR (500 MHz, DMSO-d_6) δ 10.45 (s, 1H), 9.34 (s, 1H), 7.96 (d, *J* = 8.5 Hz, 2H), 7.73 (d, *J* = 8.5 Hz, 2H), 7.41 (d, *J* = 8.8 Hz, 1H), 7.06 (d, *J* = 2.5 Hz, 1H), 6.78 (dd, *J* = 8.8, 2.5 Hz, 1H), 3.01 (q, *J* = 7.5 Hz, 2H), 1.28 (t, *J* = 7.6 Hz, 3H). ^{13}C NMR (126 MHz, DMSO) δ 163.3, 162.5, 153.8, 147.0, 142.7, 126.5, 126.0 (q, *J* = 3.8 Hz), 124.4 (q, *J* = 271.3 Hz), 123.6 (q, *J* = 32.0 Hz), 119.8, 113.0, 112.5, 111.4, 105.2, 20.9, 12.2. HRMS for $C_{18}H_{15}F_3NO_3$ $[M+H]^+$ calc. 350.09985, found 350.10051 (error = -1.56).

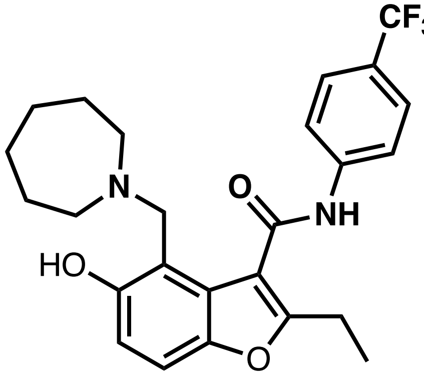

4-(azepan-1-ylmethyl)-2-ethyl-5-hydroxy-*N*-(4-(trifluoromethyl)phenyl)benzofuran-3-carboxamide. **(29)** To a solution of the benzofuran (22 mg, 63.0 μmol, 1 equiv.) in ethanol (630 μL, 0.1 M) in a 4 mL scintillation

vial was added hexamethyleneimine (21.3 µL, 189 µmol, 3 equiv.) and formaldehyde (37 wt% in H2O, 14.1 µL, 189 µmol, 3 equiv.). The scintillation vial was capped tightly and heated in an oil bath at 80°C for 18h, after which it was cooled to room temperature and purified directly by HPLC (25% to 95% CH_3CN/H_2O + 0.1% TFA, 10 min gradient). The obtained fractions were combined and lyophilized to afford the product (23 mg, 40.6 µmol, 64% yield) as a colorless solid in its trifluoroacetate salt form. ^{1}H NMR (500 MHz, DMSO-d_6) δ 11.07 (s, 1H), 10.47 (s, 1H), 8.92 (s, 1H), 7.96 (d, J = 8.4 Hz, 2H), 7.79 (d, J = 8.4 Hz, 2H), 7.60 (d, J = 8.9 Hz, 1H), 7.02 (d, J = 8.9 Hz, 1H), 4.44 (d, J = 5.2 Hz, 2H), 3.39 – 3.24 (m, 2H), 3.24 – 3.10 (m, 2H), 2.99 (q, J = 7.6 Hz, 2H), 1.89 – 1.64 (m, 4H), 1.64 – 1.40 (m, 4H), 1.28 (t, J = 7.6 Hz, 3H). ^{13}C NMR (126 MHz, DMSO) δ 164.1, 162.0, 157.9 (q, J = 31.1 Hz), 153.8, 147.1, 142.1, 126.7, 126.3 (q, J = 3.8 Hz), 124.4 (q, J = 32.0 Hz), 124.3 (q, J = 271.5 Hz), 120.0, 117.2 (q, J = 299.7 Hz), 113.6, 113.1, 113.0, 107.3, 53.8, 51.6, 26.7, 22.3, 21.1, 11.8. HRMS for $C_{25}H_{28}F_3N_2O_3$ $[M+H]^+$ calc. 461.20465, found 461.20572 (error = -1.99).

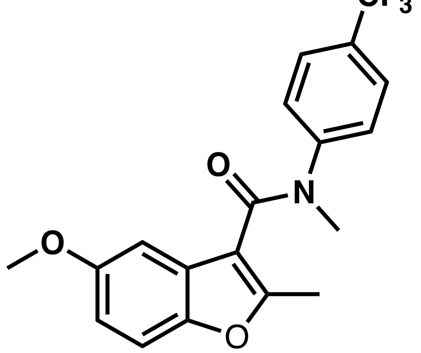

5-methoxy-N,2-dimethyl-N-(4-(trifluoromethyl)phenyl)benzofuran-3-carboxamide. **(30)** To a solution of the hydroxybenzofuran (67 mg, 200 µmol, 1 equiv.) in THF (2 mL, 0.1 M) at 0°C was added sodium hydride (60 wt%, 40 mg, 1.00 mmol, 5 equiv.). After stirring for 1h, methyl iodide (62.3 µL, 1.00 mmol, 5 equiv) was added and the solution was allowed to warm to room temperature and stir for 18h. The solution was cooled to 0°C and quenched by addition of a 5% aqeous citric acid solution (10 mL) and extracted with ethyl acetate (3 x 10 mL). The combined organic layers were washed with brine (10 mL), dried over Na_2SO_4, and purified by flash chromatography (4 g column, 0% to 30% EtOAc/Hexanes) to afford the product (29 mg, 79.8 µmol, 40% yield) as a colorless solid. ^{1}H NMR (500 MHz, Chloroform-d) δ 7.48 (d, J = 8.3 Hz, 2H), 7.25 – 7.19 (m, 3H), 6.86 (d, J = 2.5 Hz, 1H), 6.79 (dd, J = 8.9, 2.6 Hz, 1H), 3.78 (s, 3H), 3.57 (s, 3H), 2.23 (s, 3H). ^{13}C NMR (126 MHz, $CDCl_3$) δ 165.9, 156.9, 156.4, 148.6, 147.4, 128.1 (q, J = 32.9 Hz), 127.3, 126.3 (q, J = 3.8 Hz), 125.7, 123.8 (q, J = 272.0 Hz), 113.2, 111.5, 102.7, 56.1, 37.8, 14.0. HRMS for $C_{19}H_{17}F_3NO_3$ $[M+H]^+$ calc. 364.11550, found 364.11673 (error = -3.71).

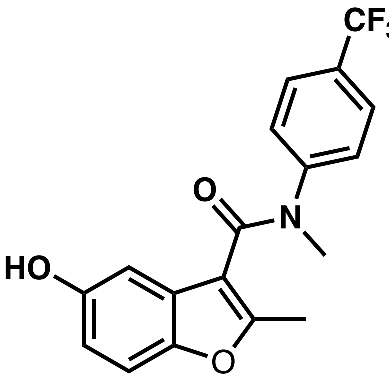

5-hydroxy-N,2-dimethyl-N-(4-(trifluoromethyl)phenyl)benzofuran-3-carboxamide. **(31)** To a solution of the methoxybenzofuran (23 mg, 63.3 µmol, 1 equiv.) in CH_2Cl_2 (317 µL) at 0°C was added boron tribromide (1 M in CH_2Cl_2, 317 µL, 317 µmol, 5 equiv.). After 30 min, the reaction was quenched by dropwise addition of methanol. The reaction was concentrated *in vacuo* and purified by flash chromatography (4 g column, 0% to 60% EtOAc/Hexanes) to afford the title compound (14 mg, 37.7 µmol, 60% yield) as a colorless solid. ^{1}H NMR (500 MHz, Methanol-d_4) δ 7.55 (d, J = 8.4 Hz, 2H), 7.40 (d, J = 8.3 Hz, 2H), 7.15 (d, J = 8.8 Hz, 1H), 6.73 (d, J = 2.5 Hz, 1H), 6.67 (dd, J = 8.9, 2.5 Hz, 1H), 3.55 (s, 3H), 2.26 (s, 3H). ^{13}C NMR (126 MHz, MeOD) δ 167.9, 158.0, 154.8, 149.3, 148.5, 129.4 (q, J = 32.6 Hz), 128.3, 127.6, 127.1 (q, J = 3.8 Hz), 125.3 (q, J = 270.9 Hz), 114.1, 113.8, 112.0, 105.7, 38.1, 13.6. HRMS for $C_{18}H_{15}F_3NO_3$ [M+H]$^+$ calc. 350.09985, found 350.10068 (error = -2.20).

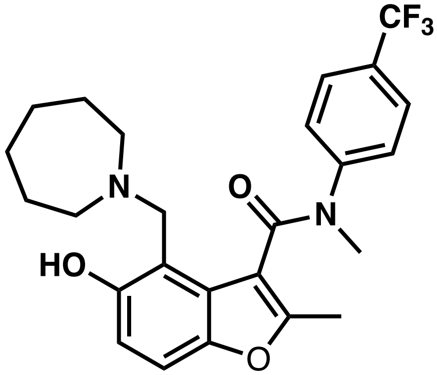

4-(azepan-1-ylmethyl)-5-hydroxy-N,2-dimethyl-N-(4-(trifluoromethyl)phenyl)benzofuran-3-carboxamide. **(32)** To a solution of the hydroxybenzofuran (12 mg, 35.4 µmol, 1 equiv.) in ethanol (354 µL, 0.1 M) in a 4 mL scintillation vial was added hexamethyleneimine (12.0 µL, 106 µmol, 3 equiv.) and formaldehyde (37 wt% in H2O, 7.92 µL, 106 µmol, 3 equiv.). The scintillation vial was capped tightly and heated in an oil bath at 80°C for 18h, after which it was cooled to room temperature, diluted in methanol, and purified directly by HPLC (35% to 95% CH_3CN/H_2O + 0.1% TFA, 10 min gradient). The obtained fractions were combined and lyophilized to afford the product (15 mg, 32.5 µmol, 92% yield) as a colorless solid in its trifluoroacetate salt form. ^{1}H NMR (500 MHz, Methanol-d_4) δ 7.63 (d, J = 8.2 Hz, 2H), 7.44 (d, J = 8.2 Hz, 2H), 7.40 (d, J = 8.8 Hz, 1H), 6.94 (d, J = 8.9 Hz, 1H), 4.76 (d, J = 13.7 Hz, 1H), 4.47 (d, J = 13.3 Hz, 1H), 3.73 – 3.54 (m, 4H), 3.49 – 3.38 (m, 1H), 2.21 – 1.92 (m, 6H), 1.91 – 1.64 (m, 5H). ^{13}C NMR (126 MHz, MeOD) δ 169.2, 162.5 (q, J = 34.9 Hz), 157.6, 155.3, 149.8, 148.2, 129.9 (q, J = 32.9 Hz), 128.5, 128.0, 127.3 (q, J = 3.7 Hz), 125.2 (q, J = 271.5 Hz), 118.0 (q, J = 293.7 Hz), 115.1, 114.2, 113.5, 107.9,

53.3, 38.5, 27.7, 27.5, 25.3, 14.0. HRMS for $C_{25}H_{28}F_3N_2O_3$ $[M+H]^+$ calc. 461.20465, found 461.20580 (error = -2.27).

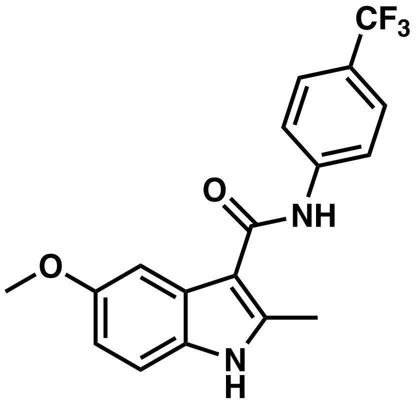

5-methoxy-2-methyl-*N*-(4-(trifluoromethyl)phenyl)-1*H*-indole-3-carboxamide. **(33)** To a solution of 5-methoxy-2-methyl-1H-indole-3-carboxylic acid (62 mg, 300 µmol, 1 equiv.) in DMF (1 mL, 0.3M) was added 1,1'-carbonyldiimizazole (49 mg, 300 µmol, 1 equiv.). The resulting solution was allowed to stir for 30 min at room temperature, after which 4-(trifluoromethyl)aniline (113 µL, 900 µmol, 3 equiv.) was added. The resulting solution was heated to 120°C and stirred overnight. The reaction mixture was diluted with ethyl acetate (30 mL), washed with brine (5 x 10 mL), dried (Na_2SO_4), and concentrated *in vacuo* and the residue purified by flash chromatography (4 g column, 0% to 40% EtOAc/Hexanes) to afford the product (68 mg, 195 µmol, 65% yield) as a slightly yellow solid. ^{1}H NMR (500 MHz, DMSO-d_6) δ 11.52 (s, 1H), 9.88 (s, 1H), 7.96 (d, *J* = 8.5 Hz, 2H), 7.68 (d, *J* = 8.5 Hz, 2H), 7.27 (d, *J* = 8.7 Hz, 1H), 7.24 (d, *J* = 2.5 Hz, 1H), 6.77 (dd, *J* = 8.7, 2.5 Hz, 1H), 3.77 (s, 3H), 2.61 (s, 3H). ^{13}C NMR (126 MHz, DMSO) δ 164.5, 154.2, 143.6, 140.7, 129.6, 126.9, 125.8 (q, *J* = 3.8 Hz), 124.6 (q, *J* = 271.1 Hz), 122.6 (q, *J* = 31.8 Hz), 119.4, 111.8, 110.9, 108.0, 102.0, 55.2, 13.4. HRMS for $C_{18}H_{16}F_3N_2O_2$ $[M+H]^+$ calc. 349.11584, found 349.11613 (error = -0.54).

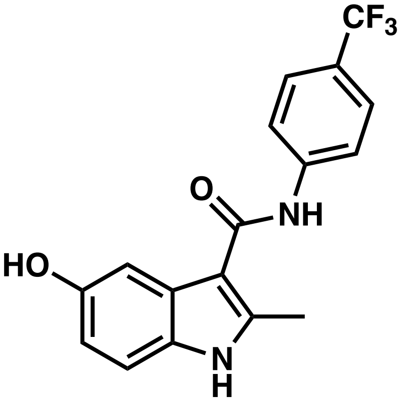

5-hydroxy-2-methyl-*N*-(4-(trifluoromethyl)phenyl)-1*H*-indole-3-carboxamide. **(34)** A suspension of methoxyindole (68 mg, 195 µmol, 1 equiv.) in CH_2Cl_2 (3 mL) under argon was cooled to -78°C. To this was added boron tribromide (1 M in CH_2Cl_2, 976 µL, 5 equiv.) and the solution was warmed to room temperature and stirred for 1 h. The solution then cooled to 0°C and quenched with the addition of methanol (2 mL) and stirred an additional 10 min. The reaction mixture was then diluted with CH_2Cl_2 (40 mL), washed with a 1:1 mixture of H_2O and Brine (3 x 20 mL). The combined organic layers were dried (Na_2SO_4), concentrated *in vacuo*, and the residue

was purified by flash chromatography (4 g column, 0% to 50% EtOAc/Hexanes) to afford the title compound (34 mg, 101 µmol, 52% yield) as a colorless residue. ^{1}H NMR (400 MHz, Methanol-d_4) δ 7.87 (d, *J* = 8.4 Hz, 2H), 7.63 (d, *J* = 8.5 Hz, 2H), 7.23 – 7.13 (m, 2H), 6.70 (dd, *J* = 8.6, 2.3 Hz, 1H), 2.64 (s, 3H). ^{13}C NMR (126 MHz, MeOD) δ 167.9, 153.1, 144.1, 142.9, 131.3, 128.6, 127.0 (q, *J* = 3.8 Hz), 126.0 (q, *J* = 32.5 Hz), 125.9 (q, *J* = 270.3 Hz), 121.0, 112.6, 112.4, 108.5, 105.0, 13.5. HRMS for $C_{17}H_{14}F_3N_2O_2$ $[M+H]^+$ calc. 335.10019, found 335.10197 (error = -4.61).

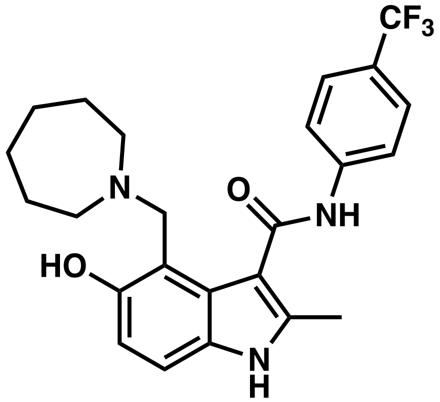

4-(azepan-1-ylmethyl)-5-hydroxy-2-methyl-*N*-(4-(trifluoromethyl)phenyl)-1*H*-indole-3-carboxamide. **(35)** To a solution of WH-227F-042 (24 mg, 73.0 µmol, 1 equiv.) in ethanol (730 µL, 0.1 M) in a 4 mL scintillation vial was added hexamethyleneimine (24.6 µL, 219 µmol, 3 equiv.) and formaldehyde (37 wt% in H2O, 16.3 µL, 219 µmol, 3 equiv.). The scintillation vial was capped tightly and heated in an oil bath at 80°C for 18h, after which it was cooled to room temperature, diluted in methanol to 1.4 mL, and purified directly by HPLC (25% to 95% CH_3CN/H_2O + 0.1% TFA, 10 min gradient). The obtained fractions were combined and lyophilized to afford the product (15 mg, 44.6 µmol, 61% yield) as a colorless solid in its trifluoroacetate salt form. ^{1}H NMR (500 MHz, DMSO-d_6) δ 11.78 (s, 1H), 10.59 (s, 1H), 9.84 (s, 1H), 9.25 (s, 1H), 7.95 (d, *J* = 8.5 Hz, 2H), 7.75 (d, *J* = 8.5 Hz, 2H), 7.35 (d, *J* = 8.6 Hz, 1H), 6.87 (d, *J* = 8.6 Hz, 1H), 4.49 (s, 2H), 3.33 – 3.27 (m, 2H), 3.27 – 3.13 (m, 2H), 2.56 (s, 3H), 1.94 – 1.81 (m, 2H), 1.81 – 1.68 (m, 2H), 1.68 – 1.49 (m, 4H). ^{13}C NMR (126 MHz, DMSO) δ 167.2, 157.8 (q, *J* = 30.6 Hz), 151.9, 142.7, 140.0, 129.7, 126.2, 126.1 (q, *J* = 4.0, 3.5 Hz), 124.4 (q, *J* = 271.2 Hz), 123.7 (q, *J* = 32.2 Hz), 119.7, 117.4 (q, *J* = 301.3 Hz), 114.1, 111.3, 108.8, 105.4, 53.3, 52.1, 26.4, 23.1, 13.6. HRMS for $C_{24}H_{27}F_3N_3O_2$ $[M+H]^+$ calc. 446.20499, found 446.20502 (error = -0.02).

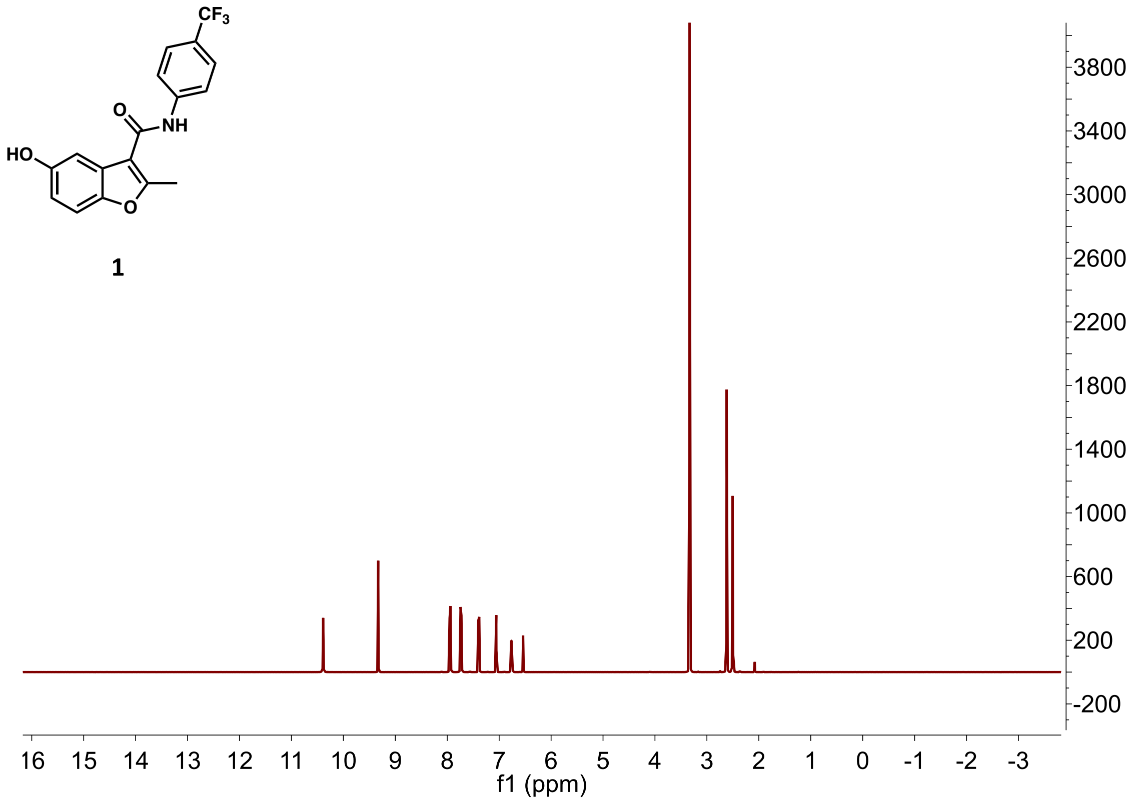

Supplementary Figure 10: ^{1}H NMR of **1**

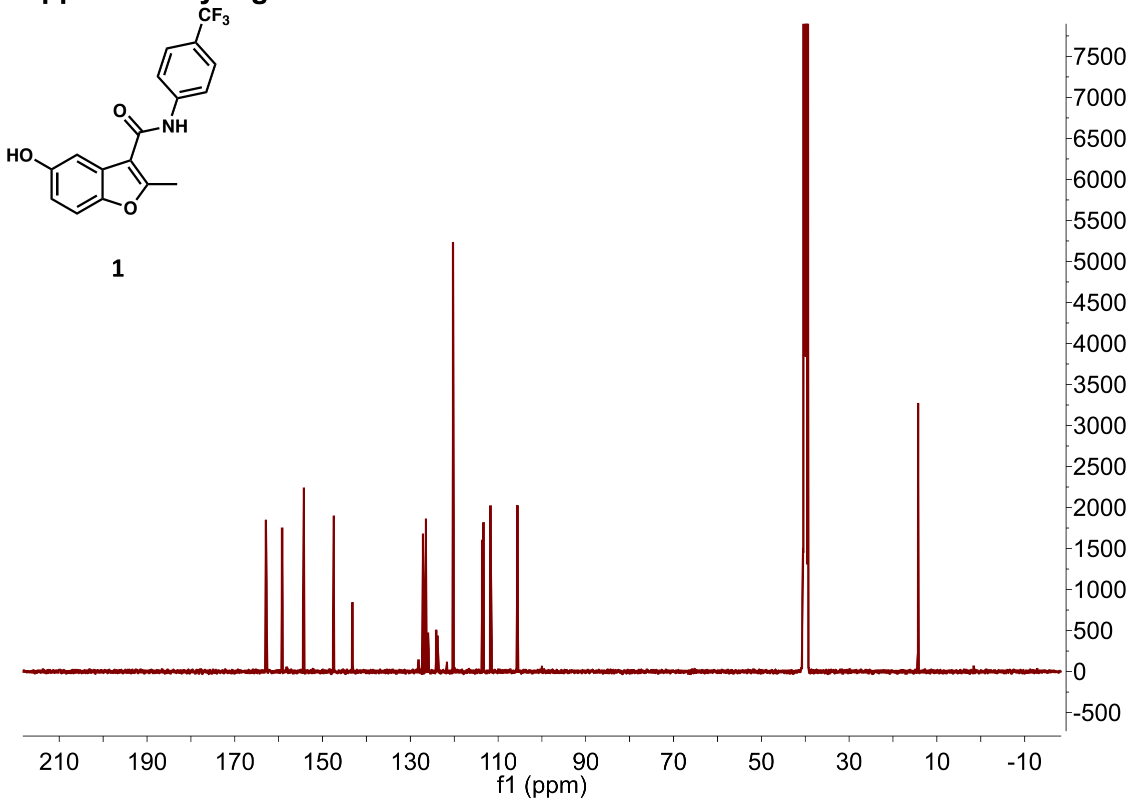

Supplementary Figure 11: ^{13}C NMR of **1**

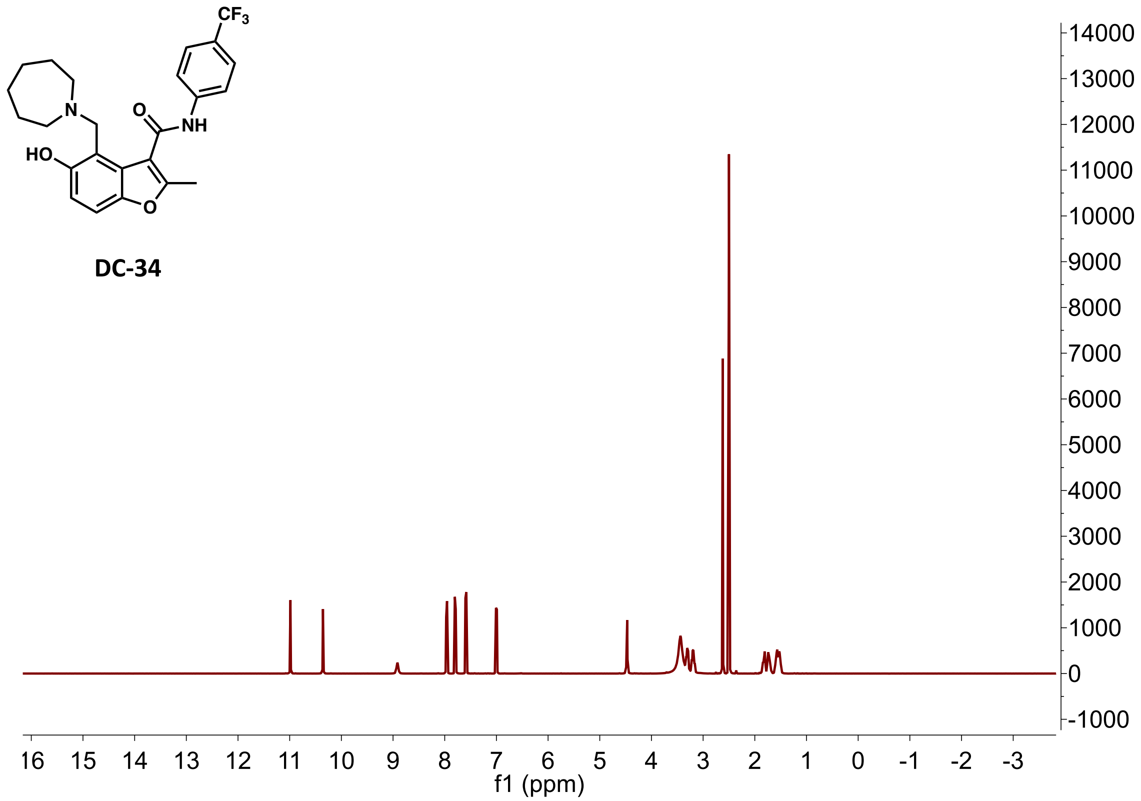

Supplementary Figure 12: ^{1}H NMR of **DC-34**

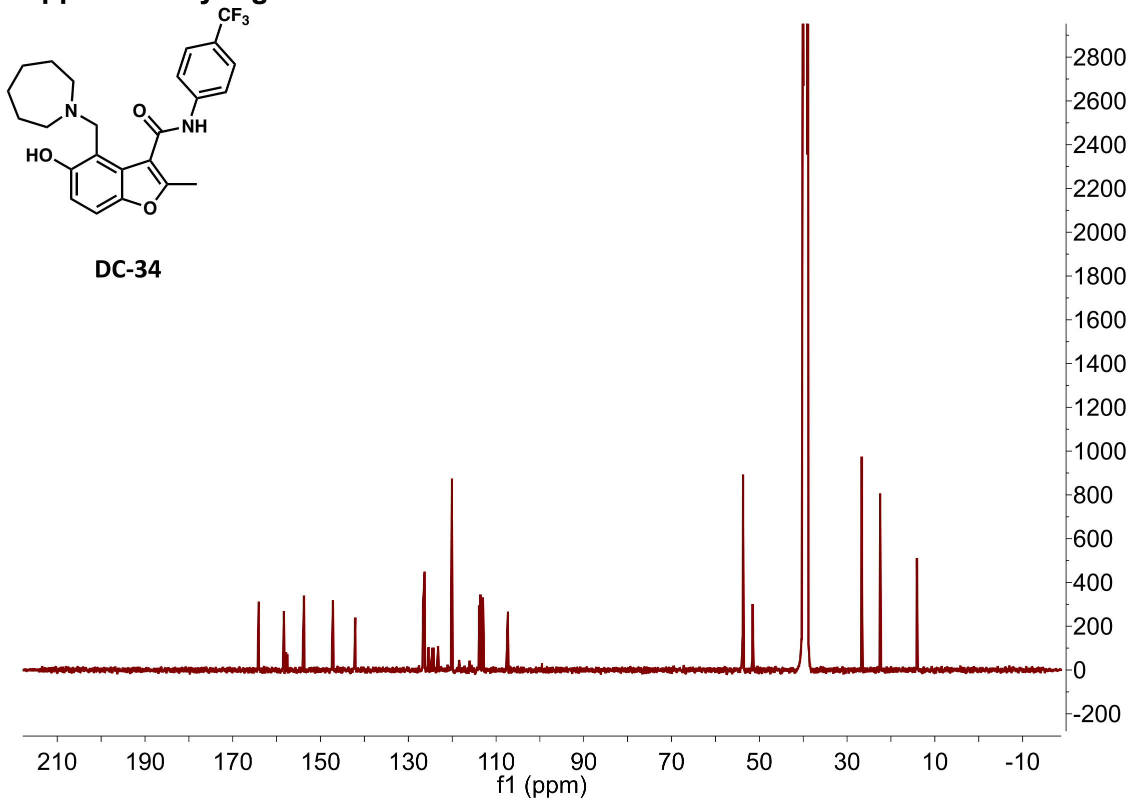

Supplementary Figure 13: ^{13}C NMR of **DC-34**

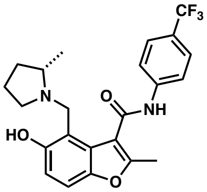

2

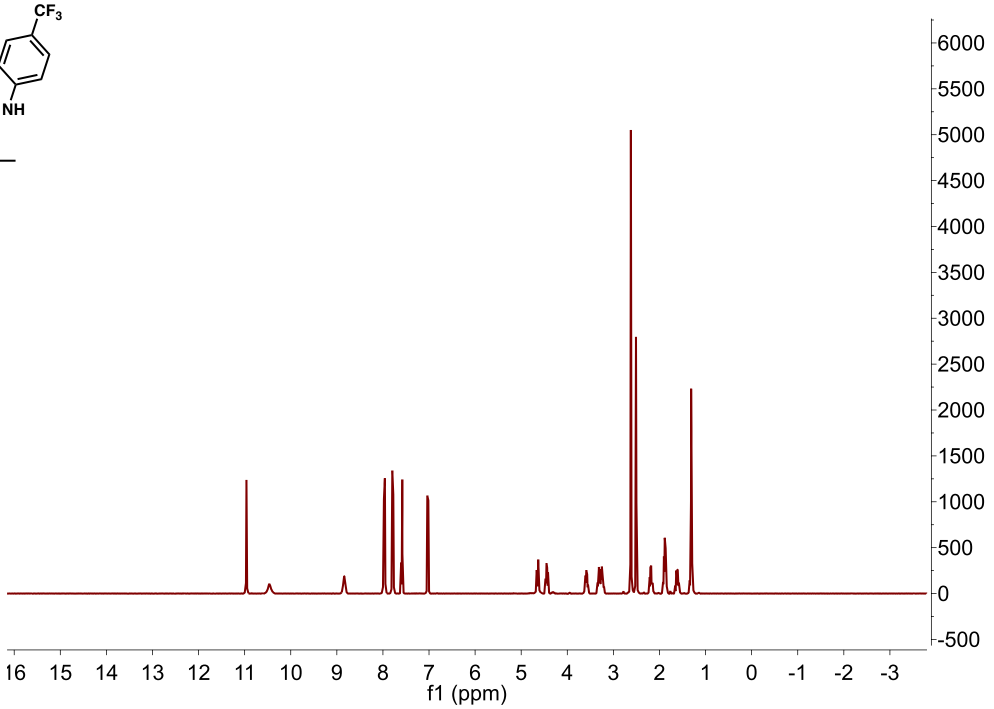

Supplementary Figure 14: ^{1}H NMR of **2**

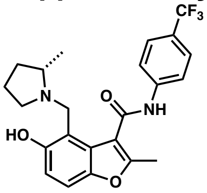

2

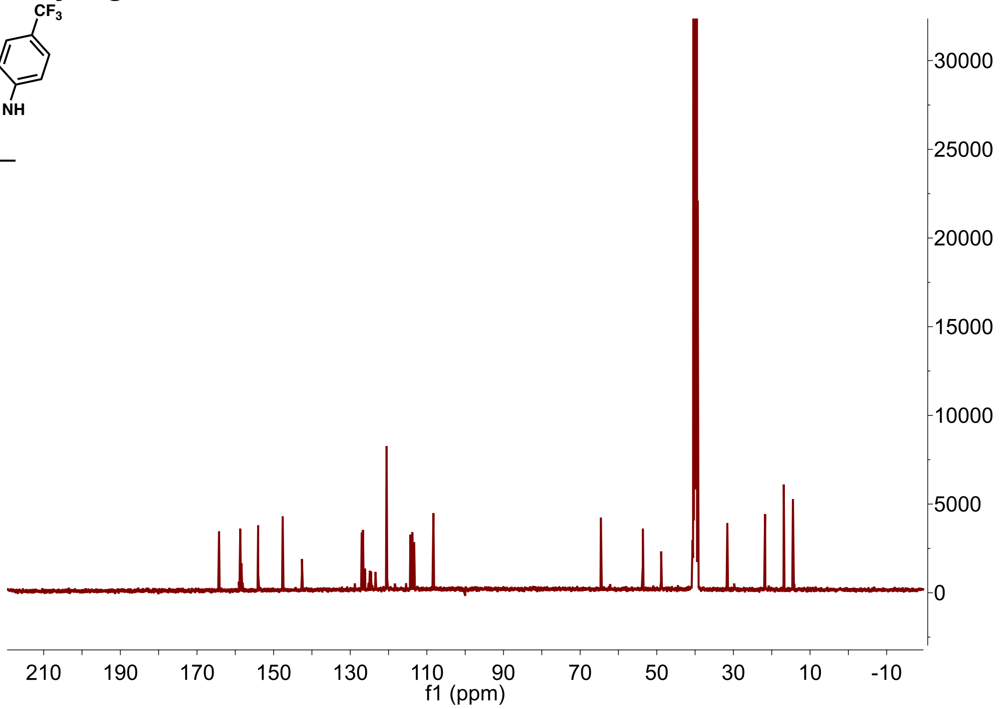

Supplementary Figure 15: ^{13}C NMR of **2**

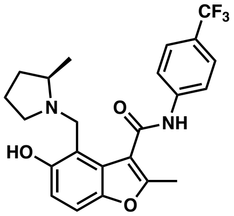

3

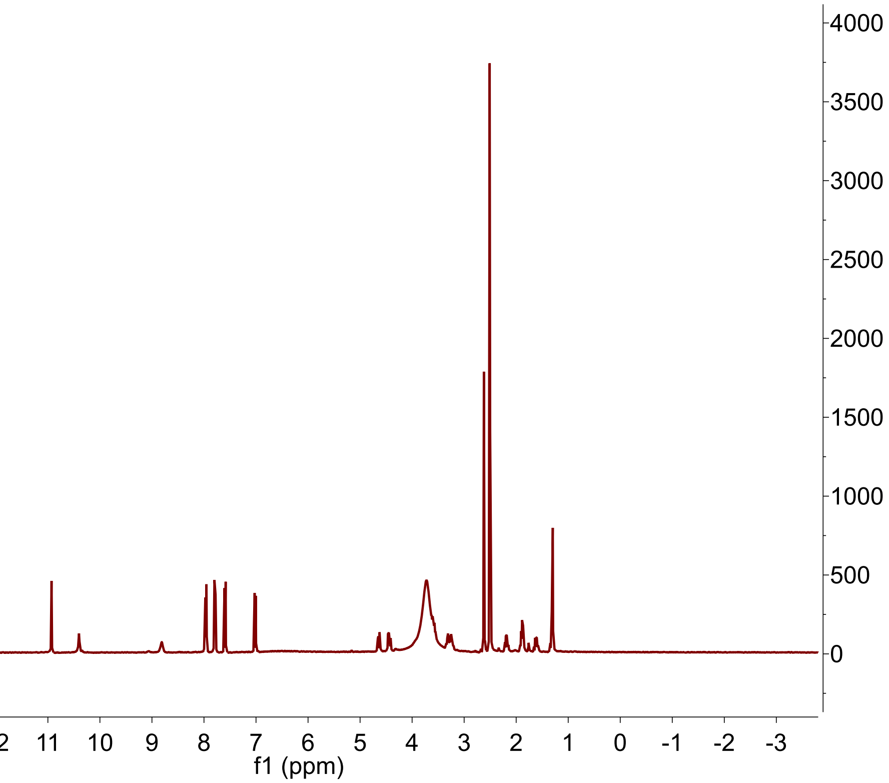

Supplementary Figure 16: ^{1}H NMR of **3**

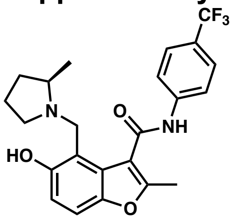

3

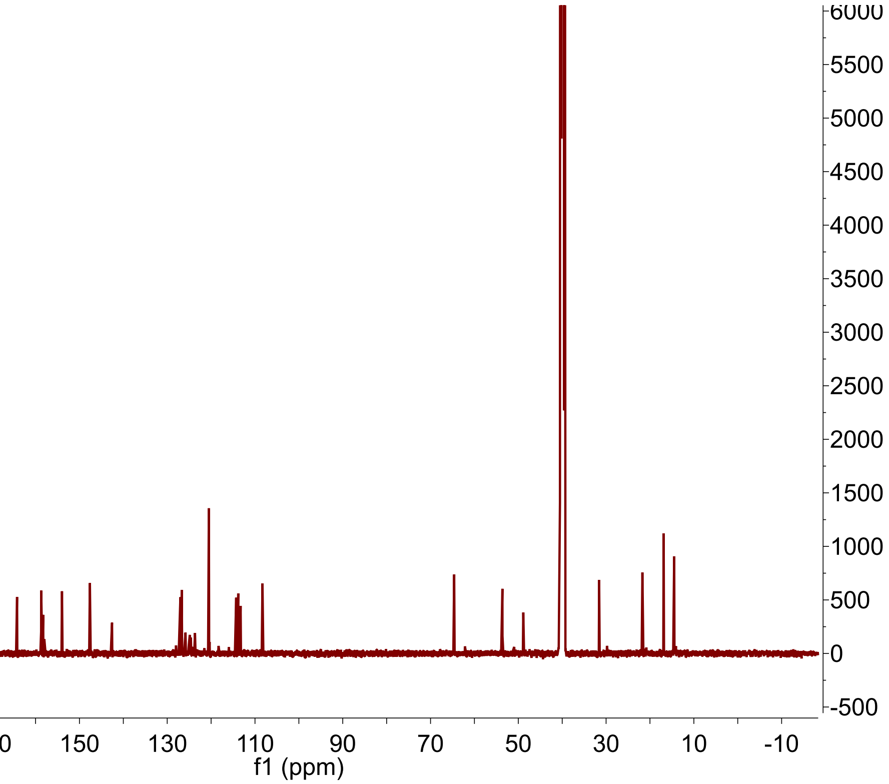

Supplementary Figure 17: ^{13}C NMR of **3**

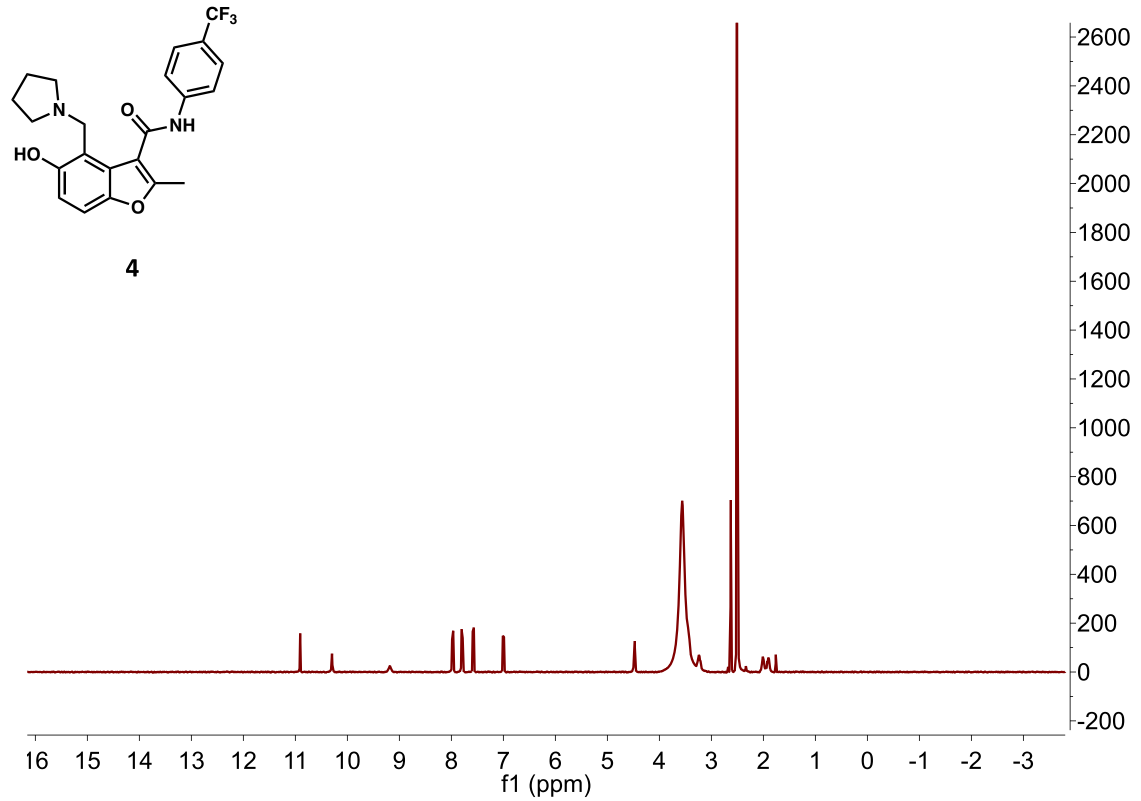

Supplementary Figure 18: ^{1}H NMR of **4**

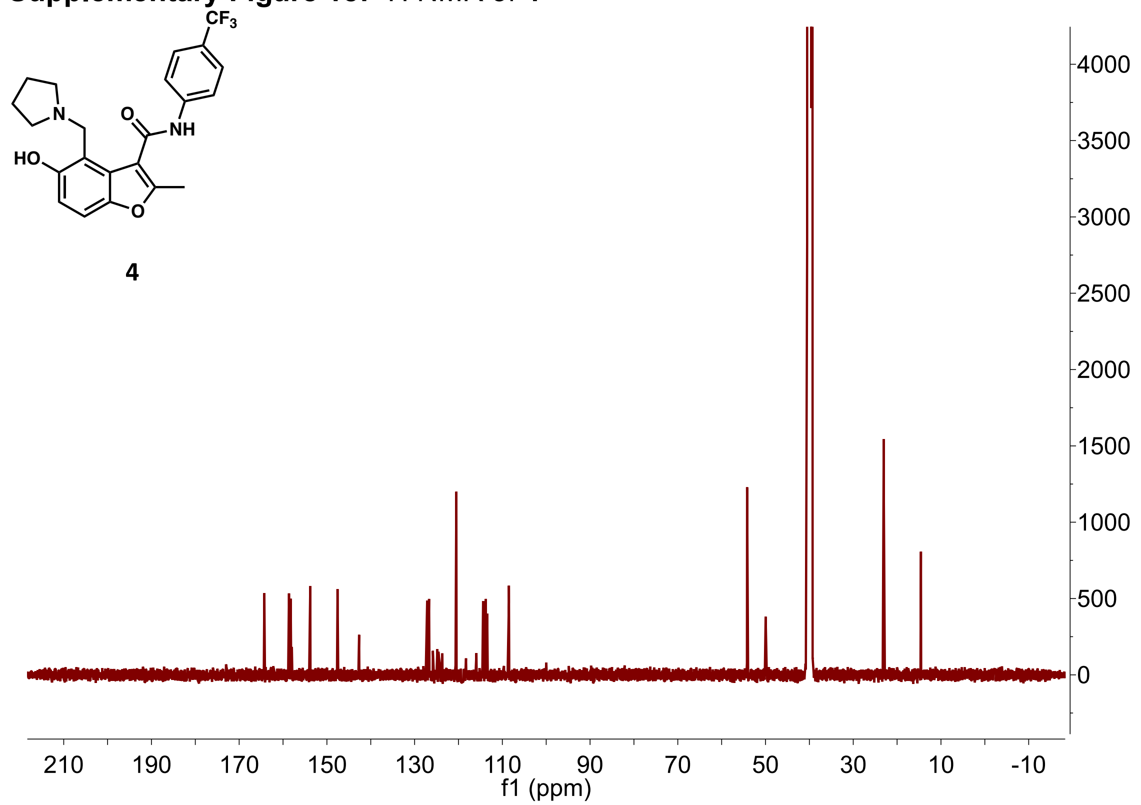

Supplementary Figure 19: ^{13}C NMR of **4**

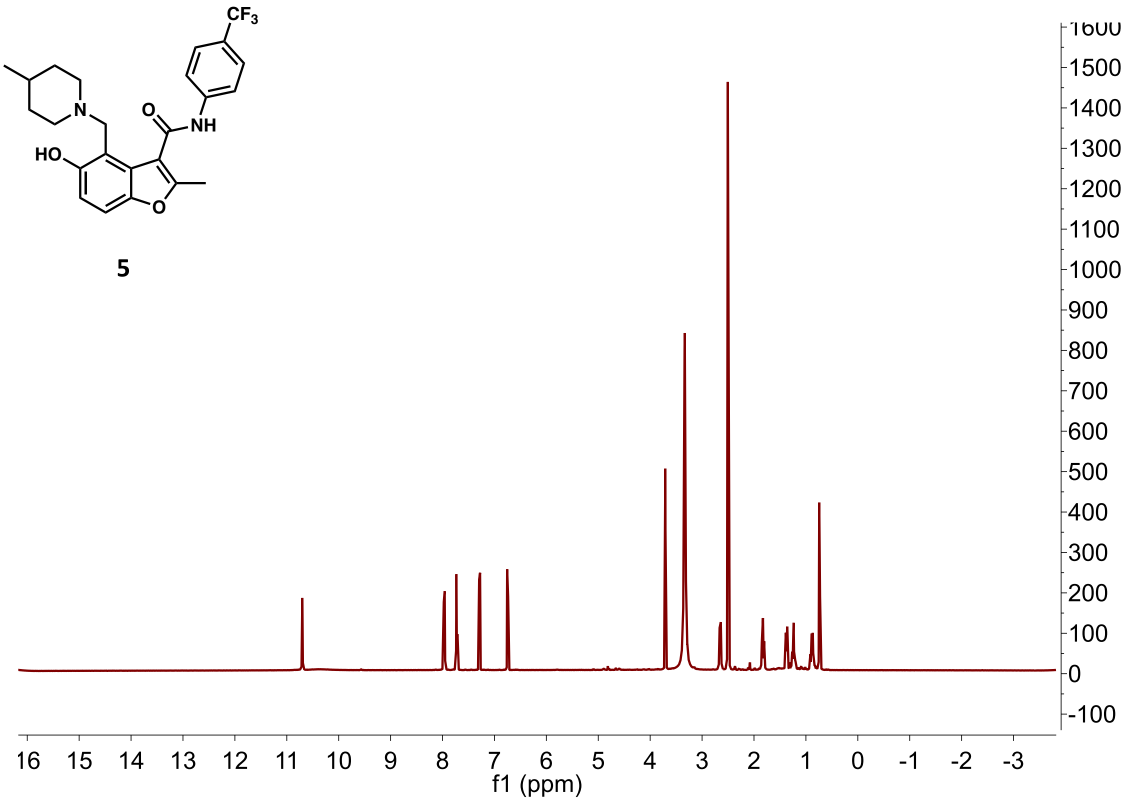

Supplementary Figure 20: ^{1}H NMR of **5**

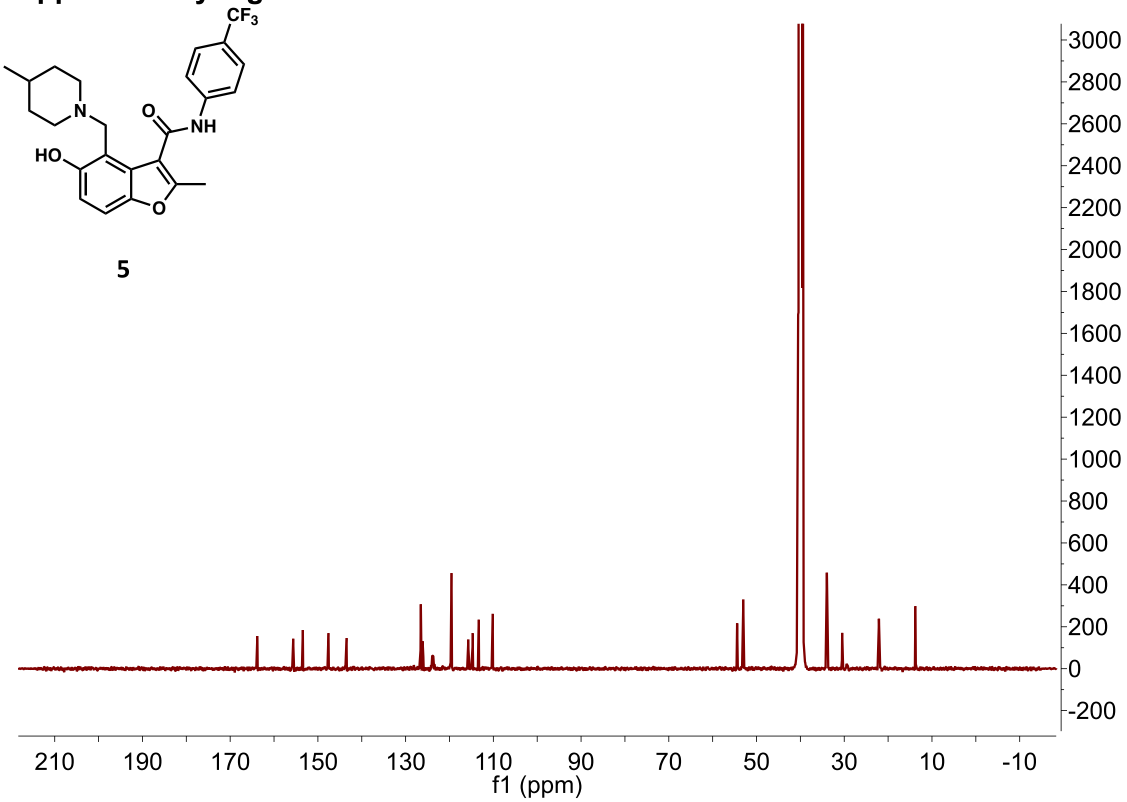

Supplementary Figure 21: ^{13}C NMR of **5**

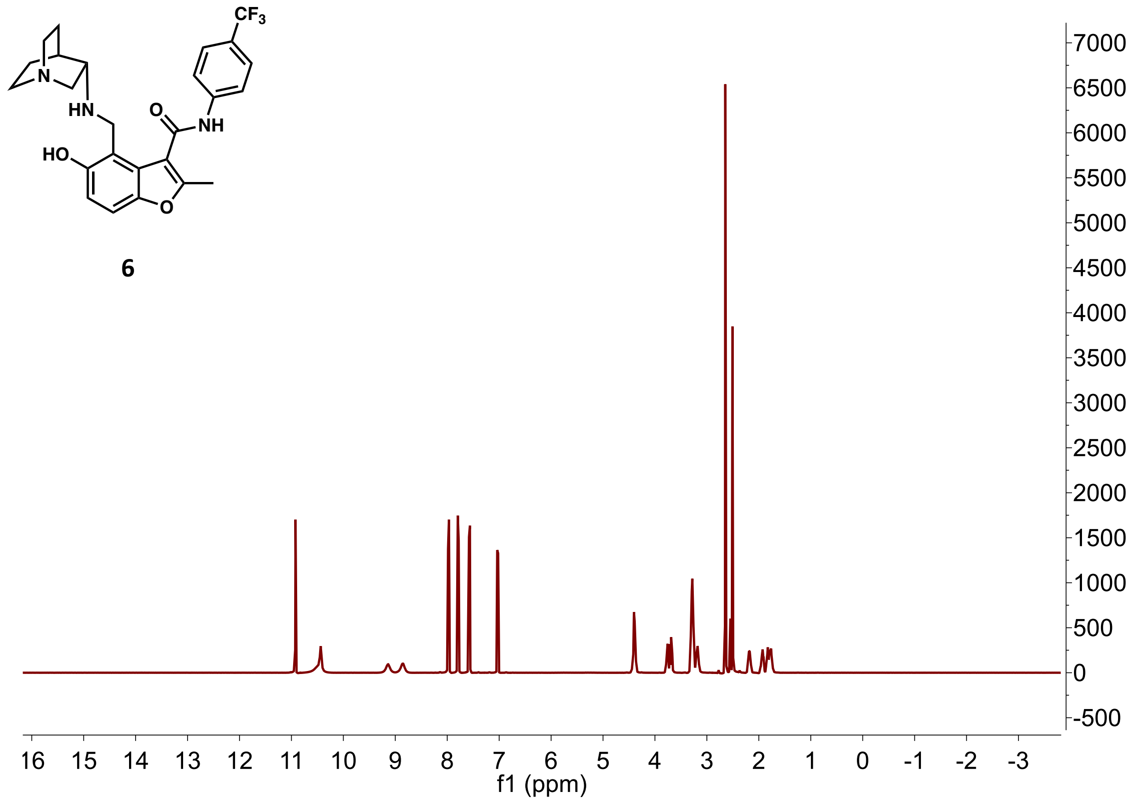

Supplementary Figure 22: ^{1}H NMR of **6**

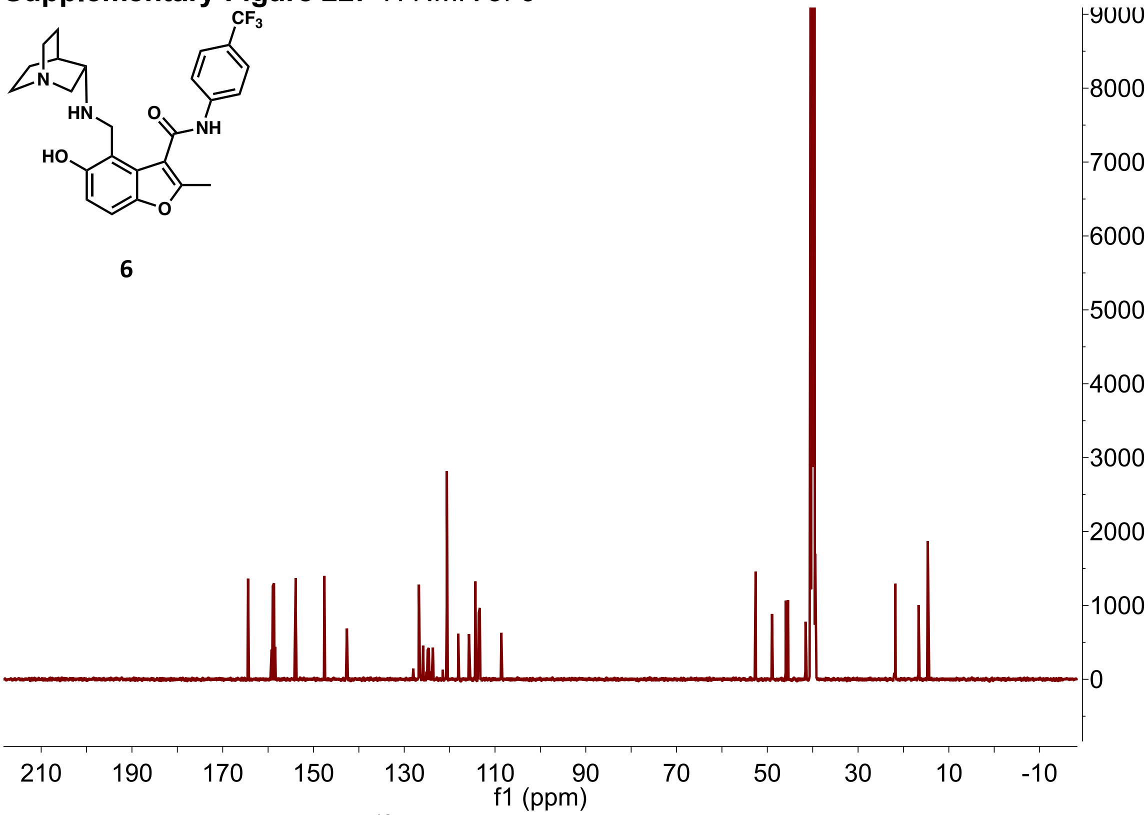

Supplementary Figure 23: ^{13}C NMR of **6**

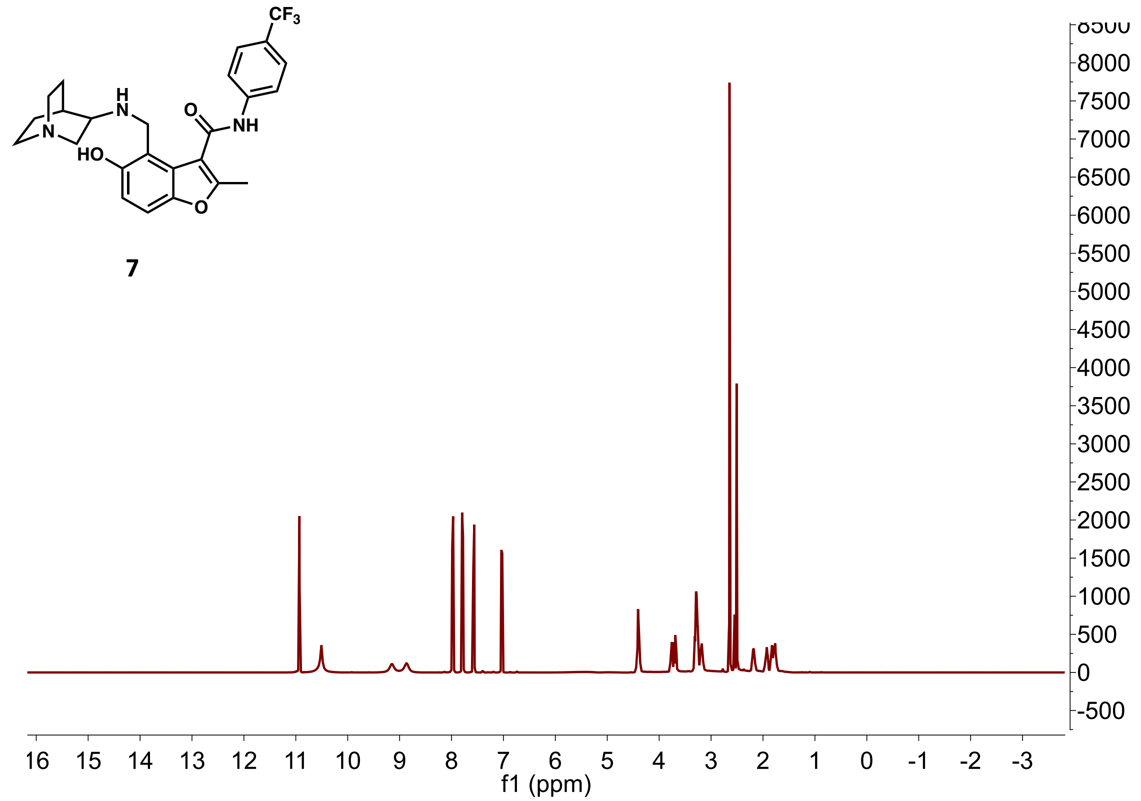

Supplementary Figure 24: ^{1}H NMR of **7**

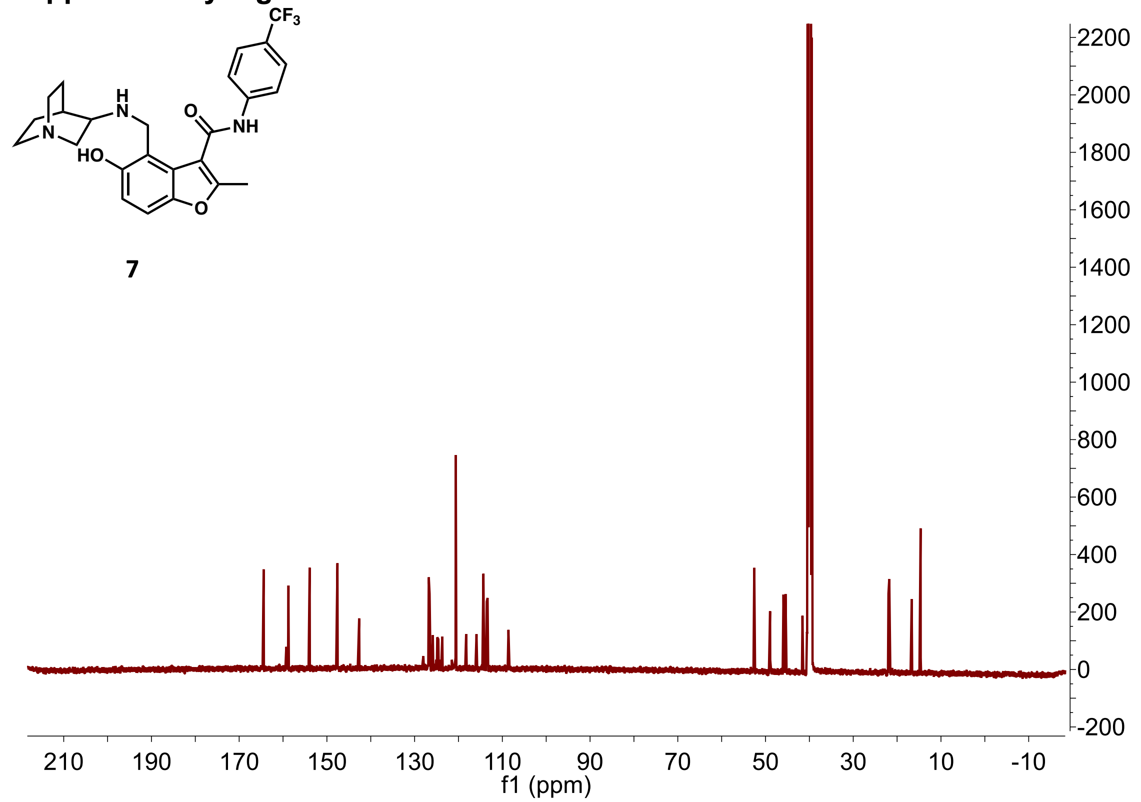

Supplementary Figure 25: ^{13}C NMR of **7**

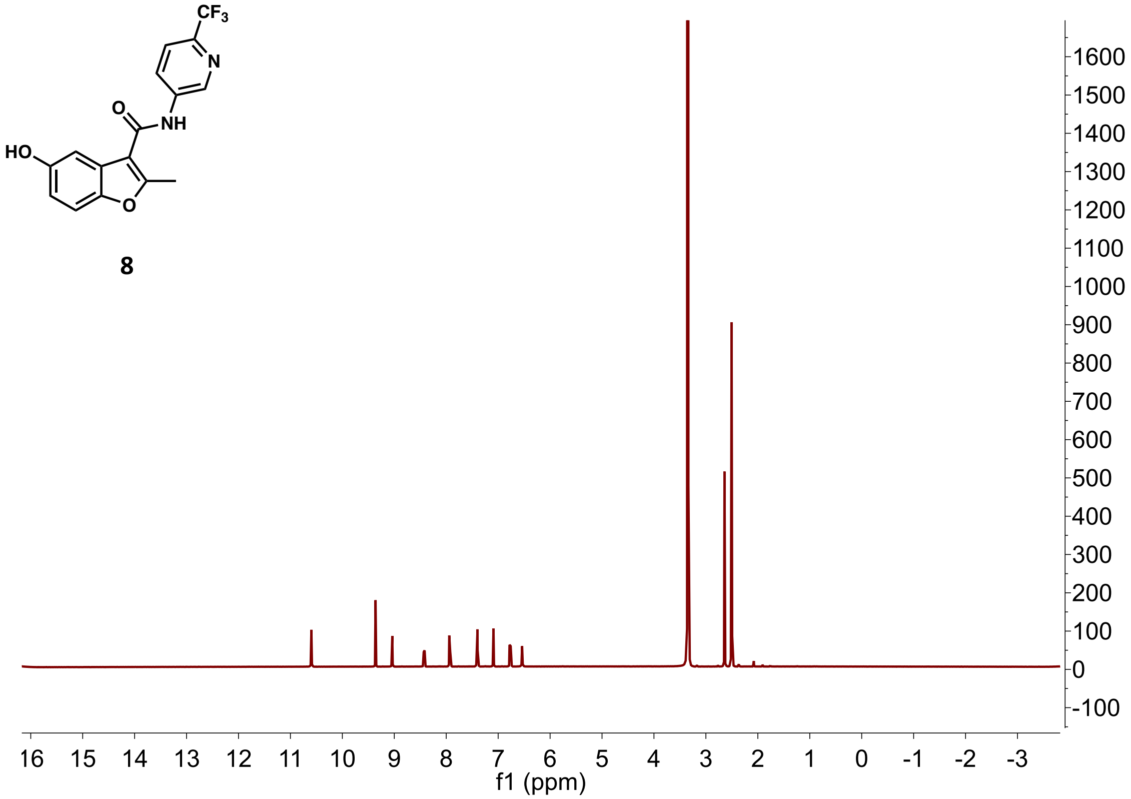

Supplementary Figure 26: ^{1}H NMR of **8**

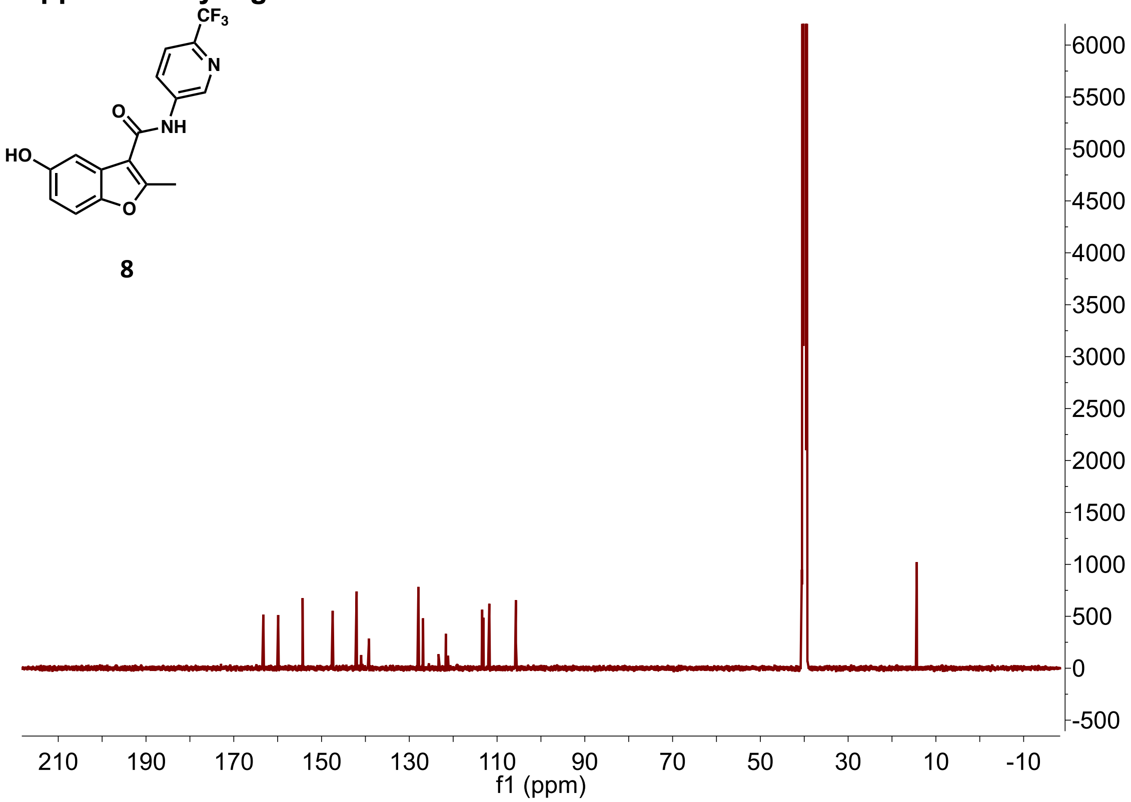

Supplementary Figure 27: ^{13}C NMR of **8**

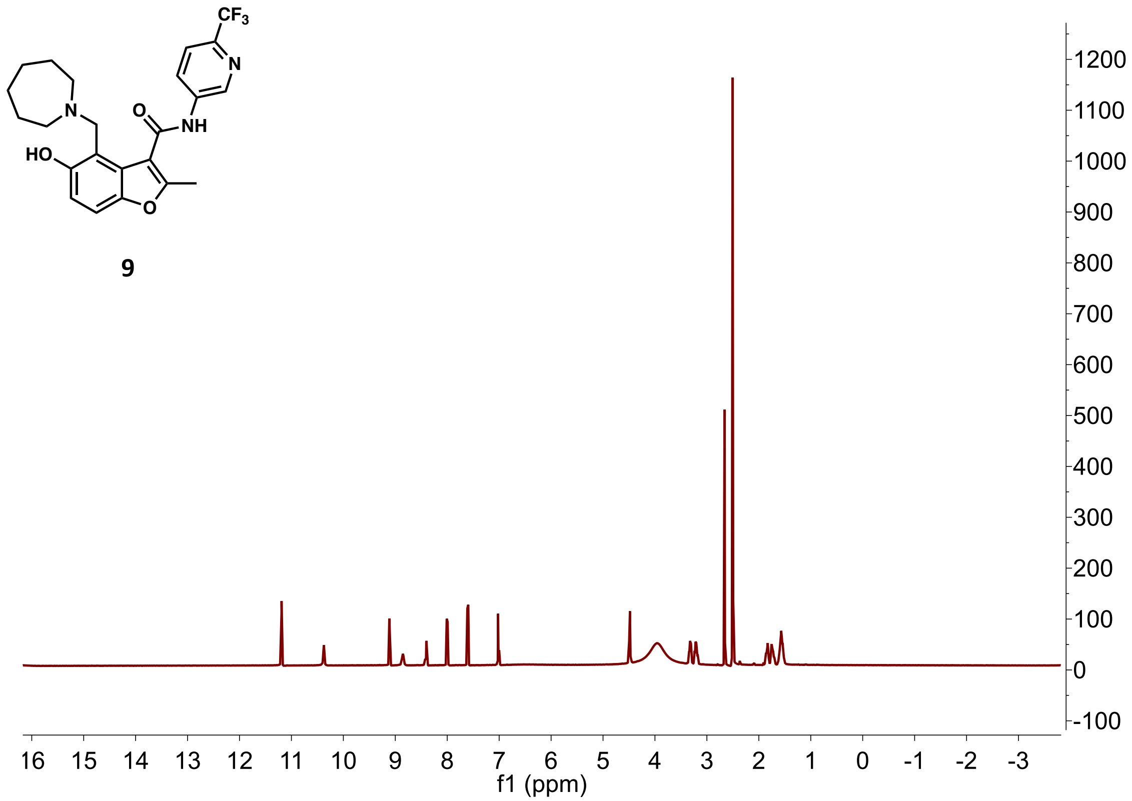

Supplementary Figure 28: ^{1}H NMR of **9**

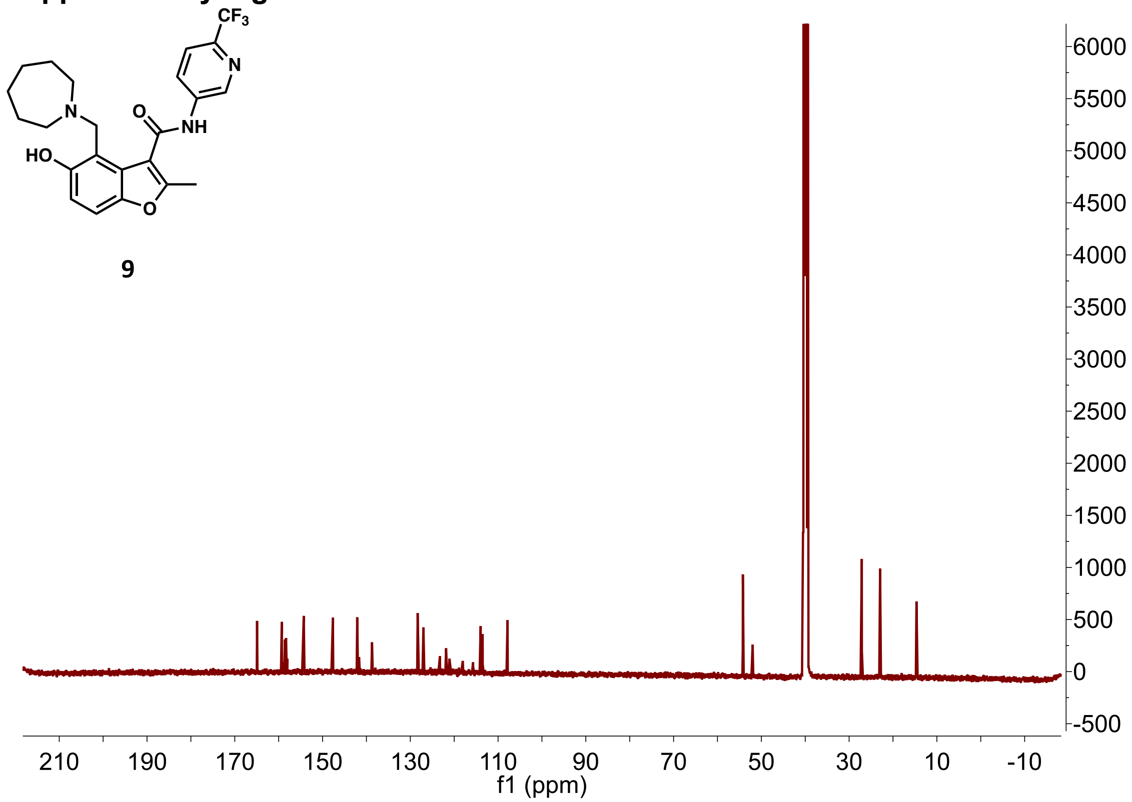

Supplementary Figure 29: ^{13}C NMR of **9**

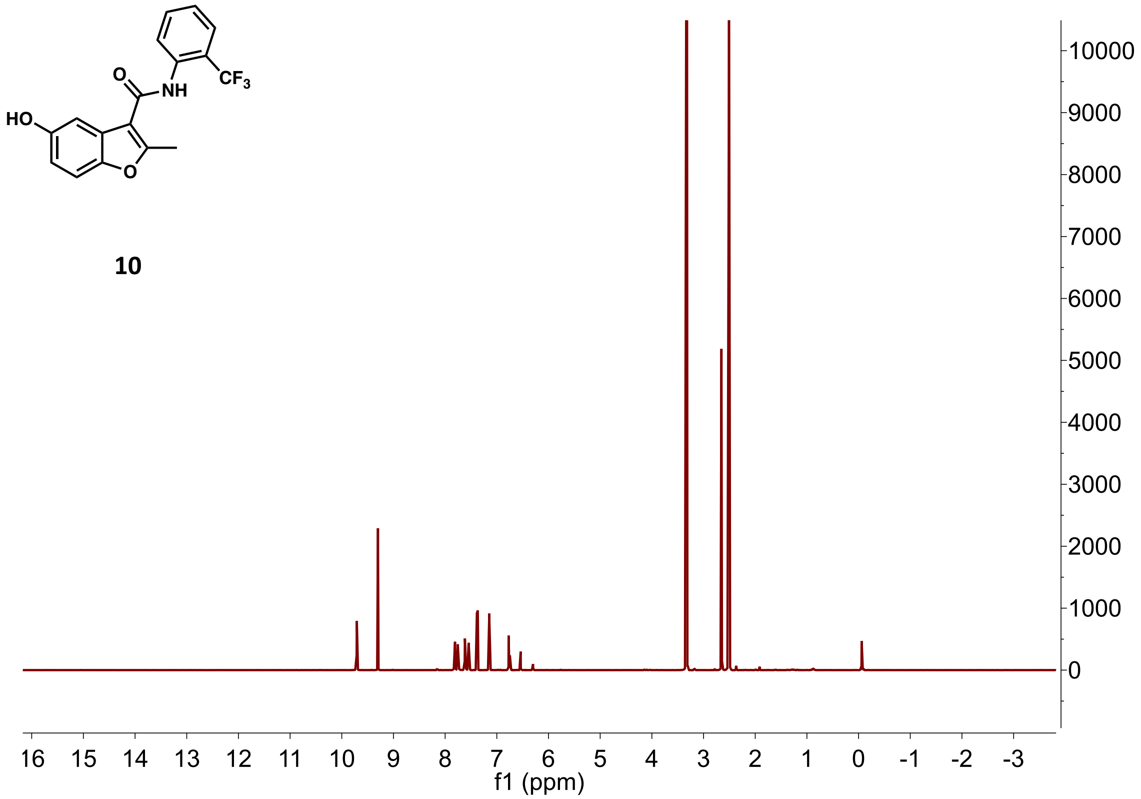

Supplementary Figure 30: ^{1}H NMR of **10**

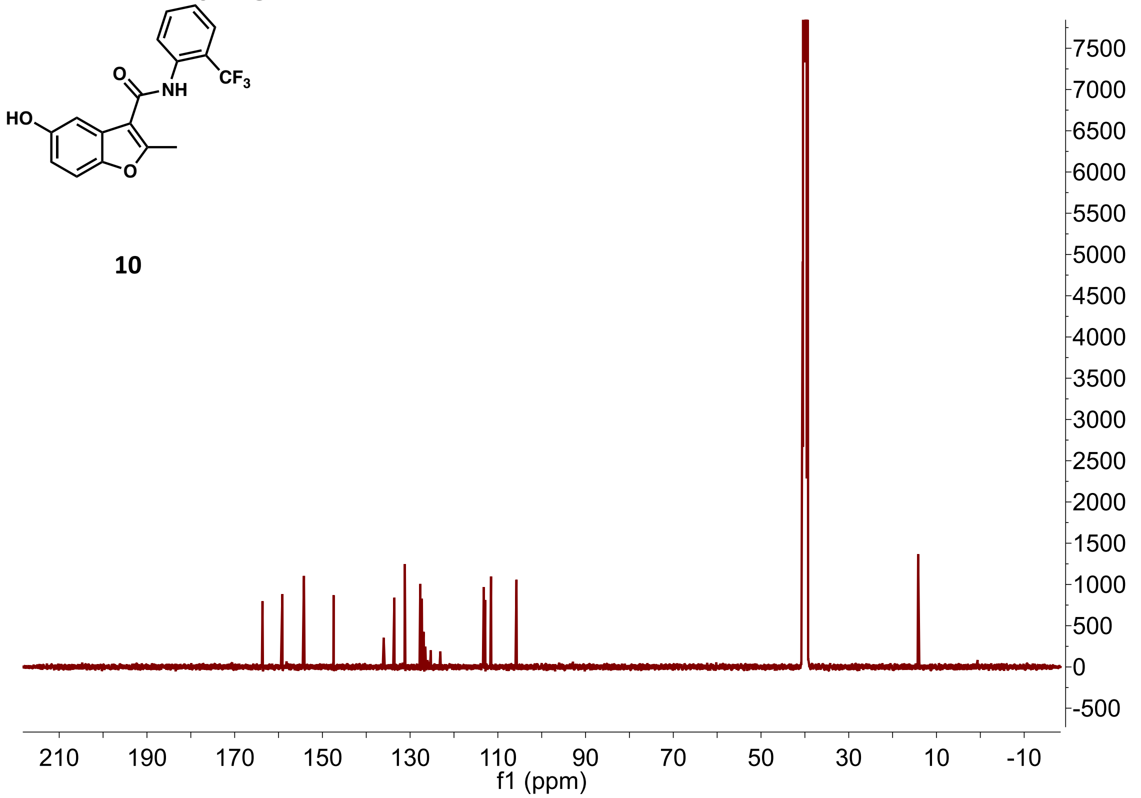

Supplementary Figure 31: ^{13}C NMR of **10**

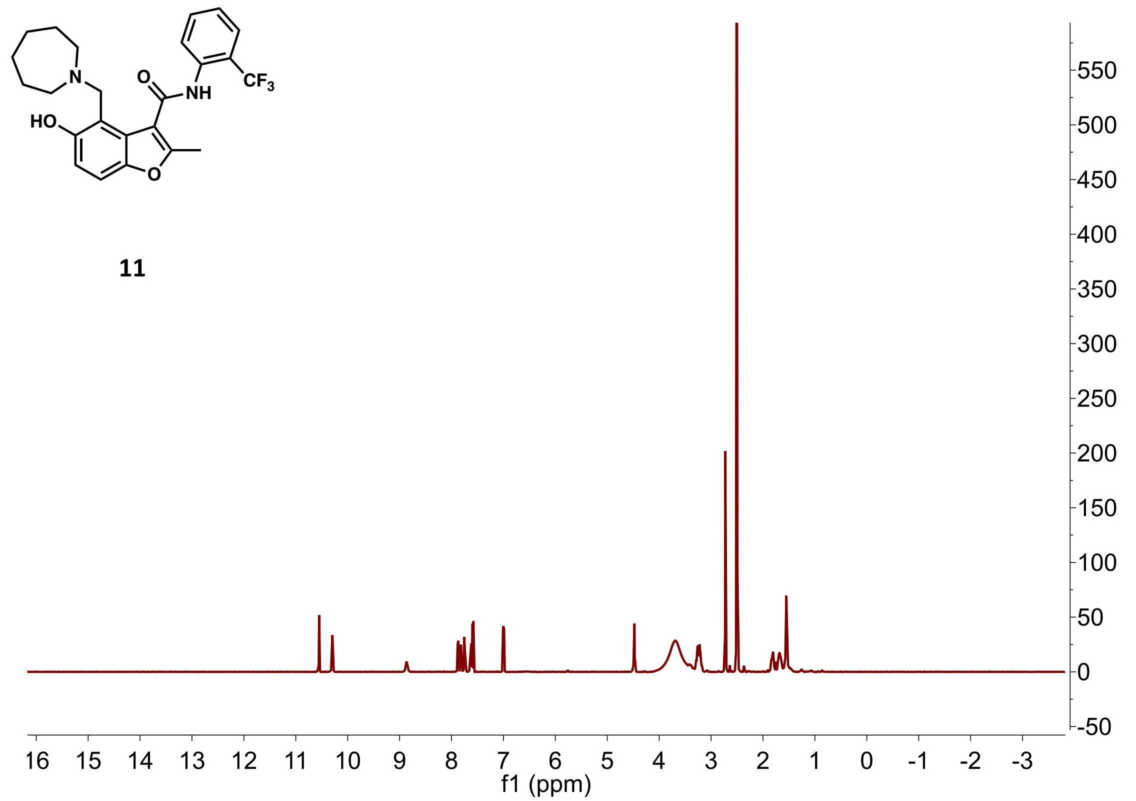

Supplementary Figure 32: ^{1}H NMR of **11**

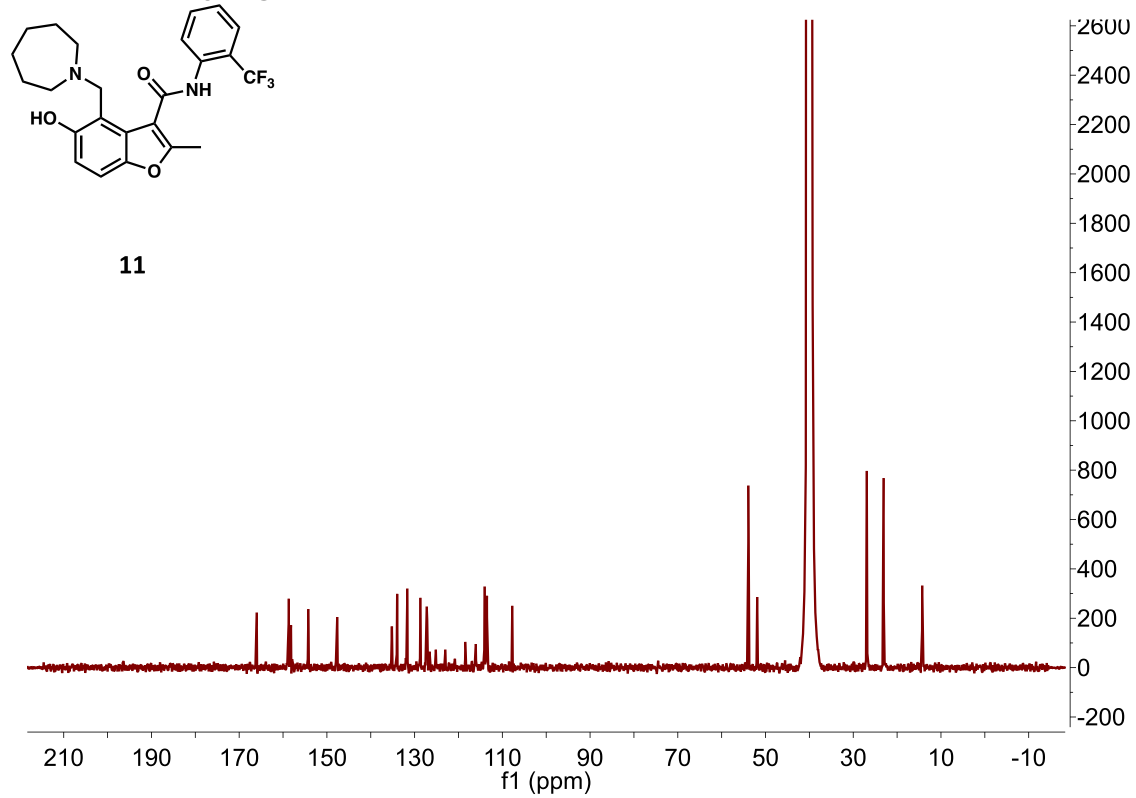

Supplementary Figure 33: ^{13}C NMR of **11**

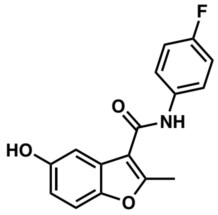

12

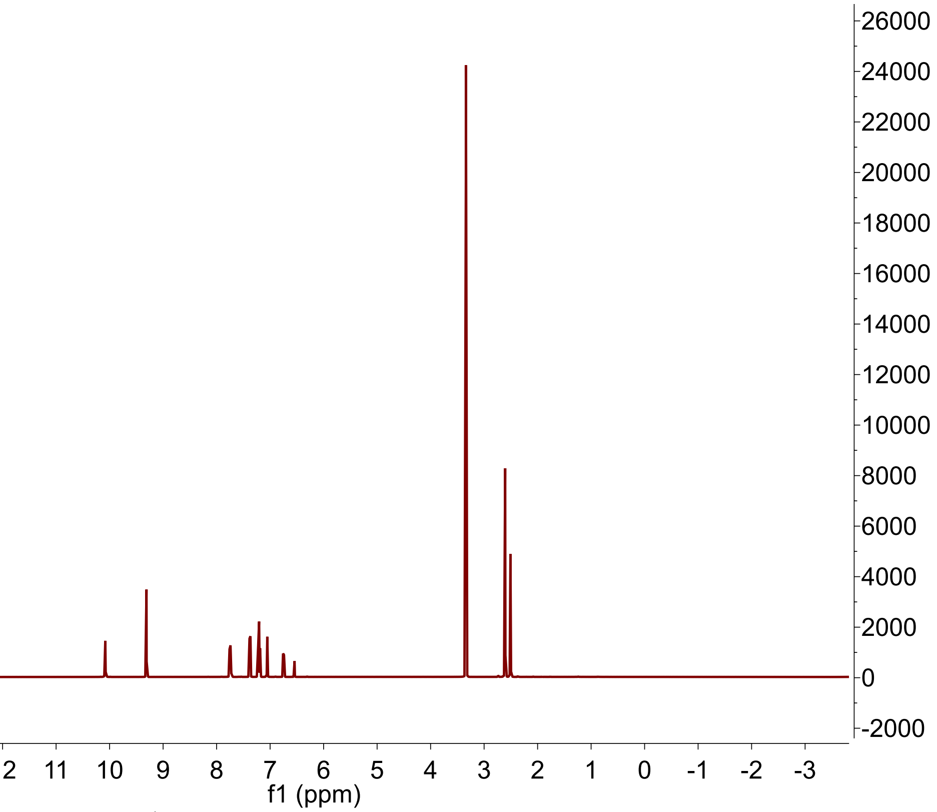

Supplementary Figure 34: ^{1}H NMR of **12**

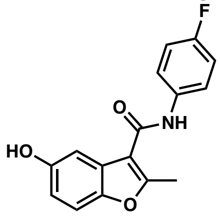

12

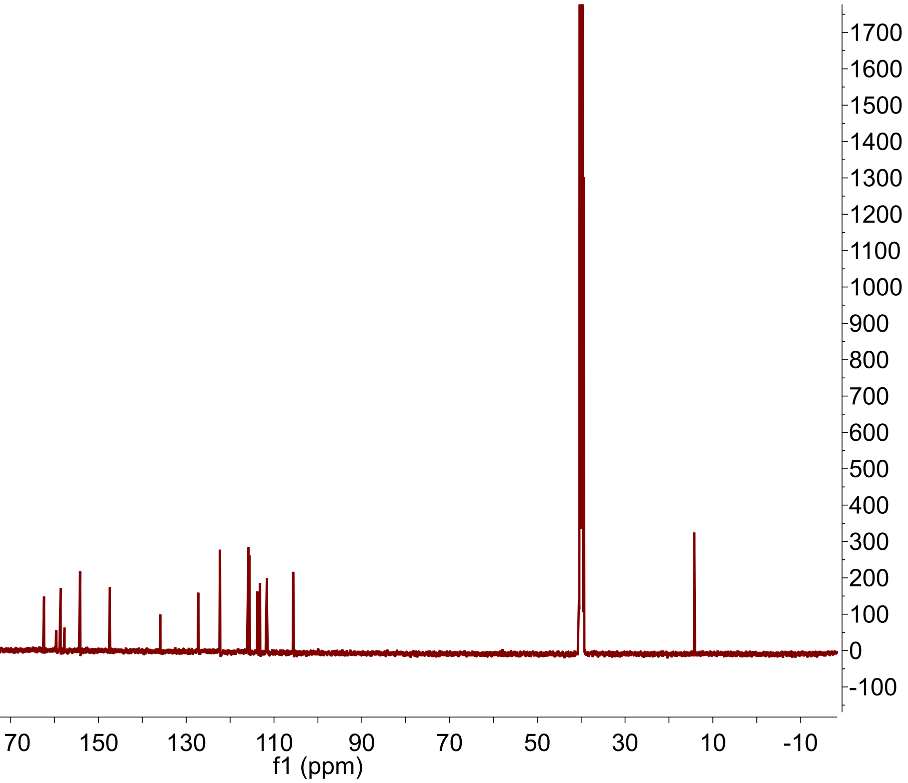

Supplementary Figure 35: ^{13}C NMR of **12**

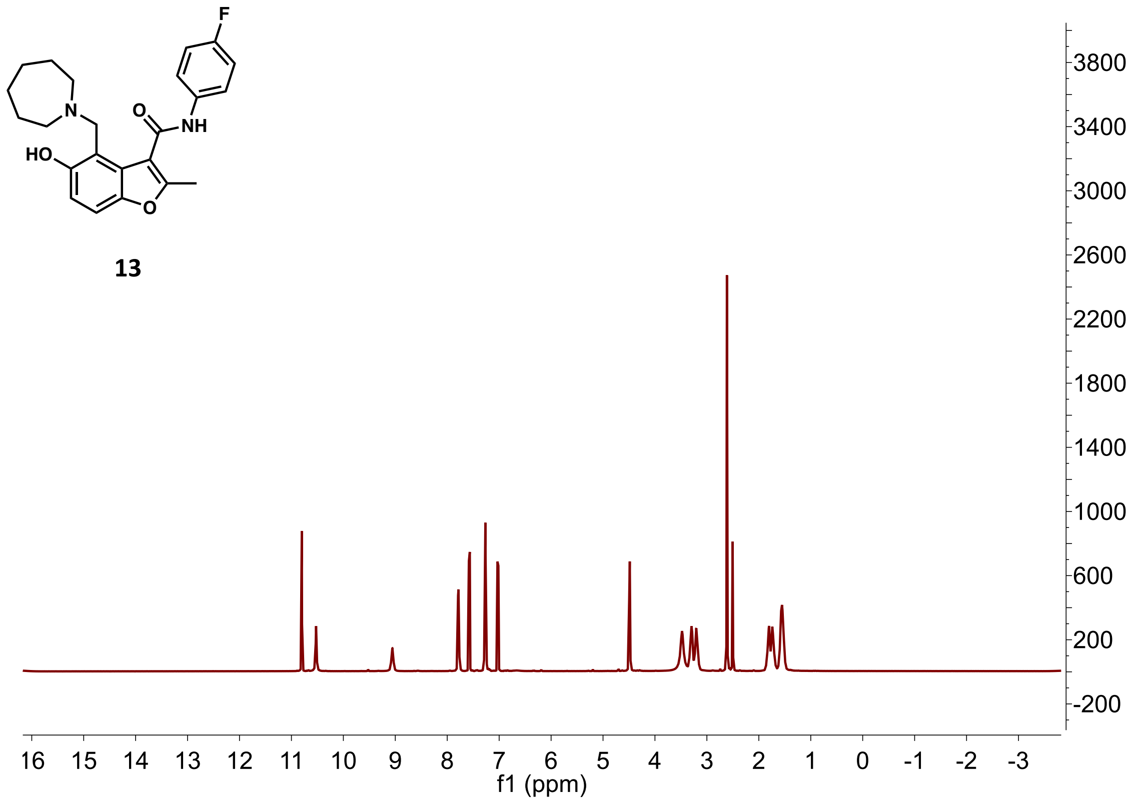

Supplementary Figure 36: ^{1}H NMR of **13**

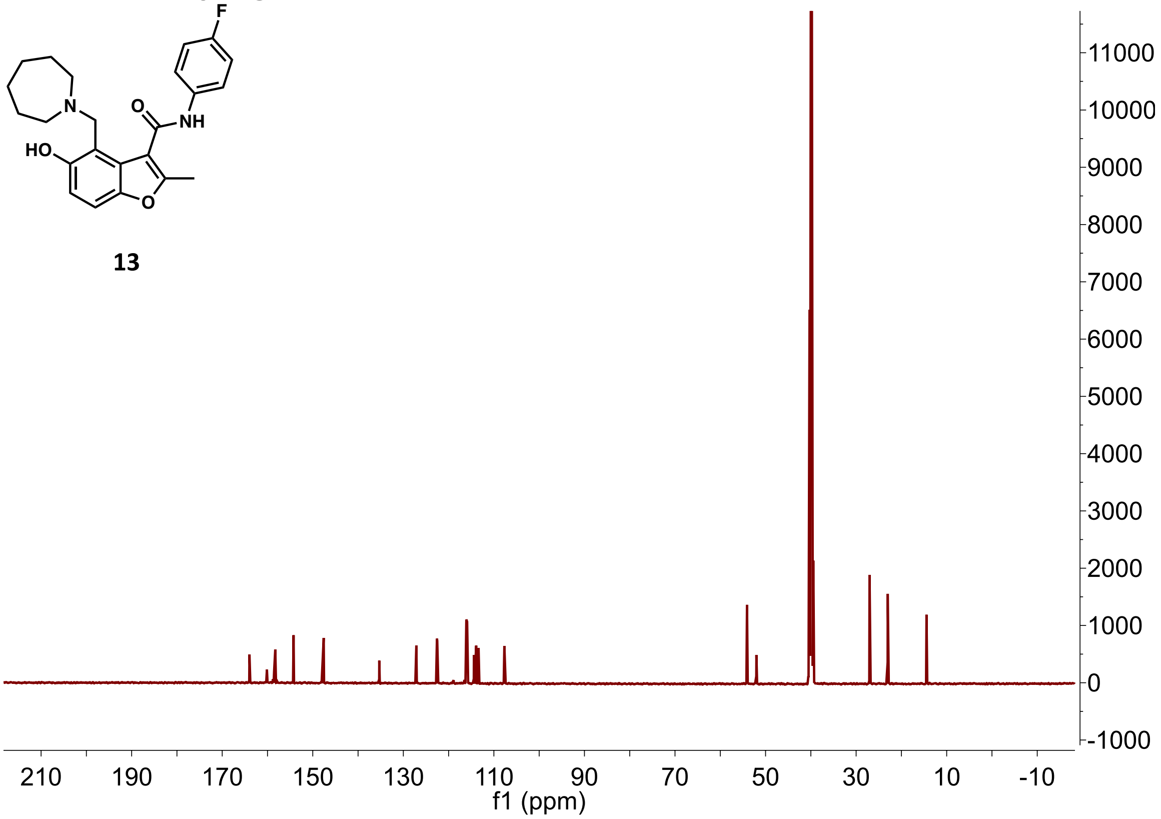

Supplementary Figure 37: ^{13}C NMR of **13**

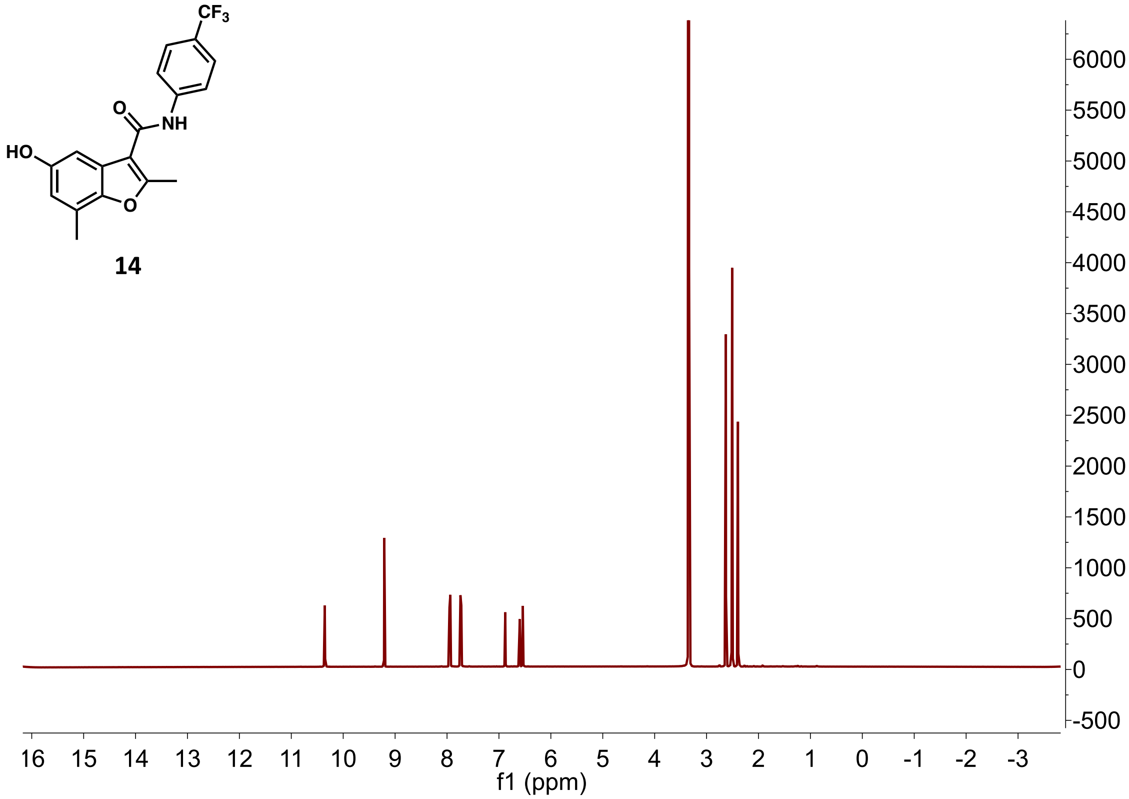

Supplementary Figure 38: ^{1}H NMR of **14**

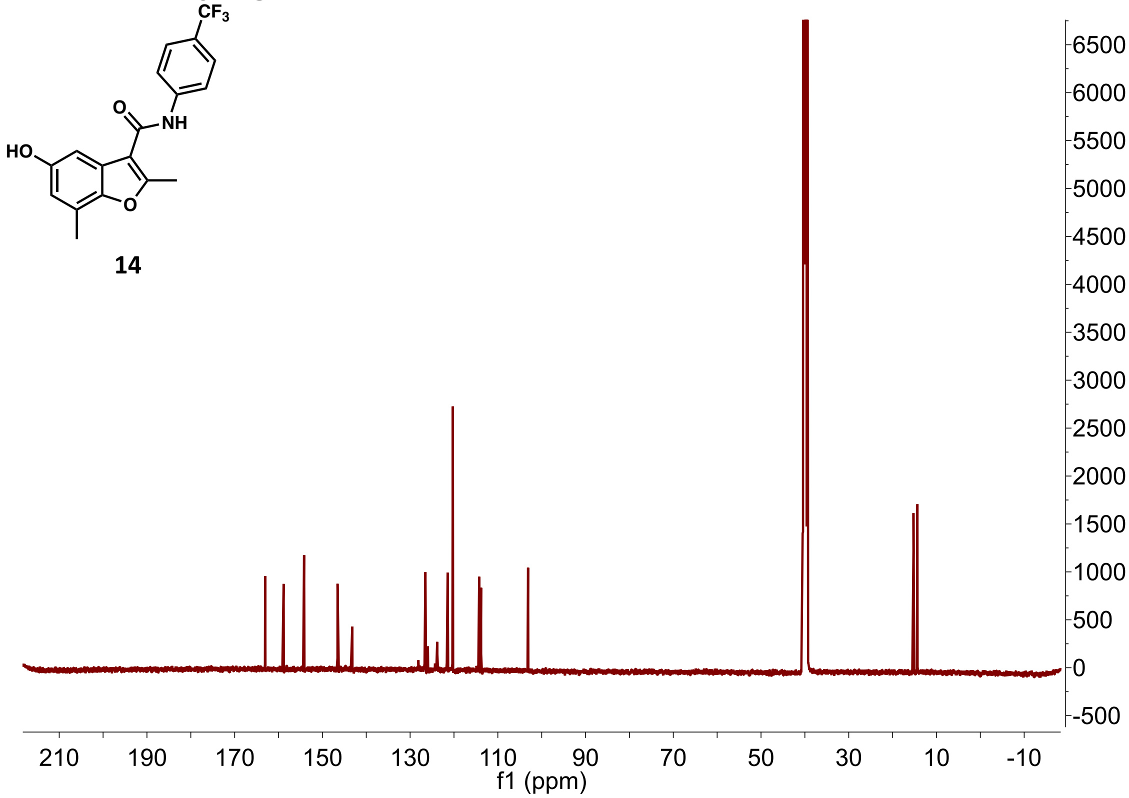

Supplementary Figure 39: ^{13}C NMR of **14**

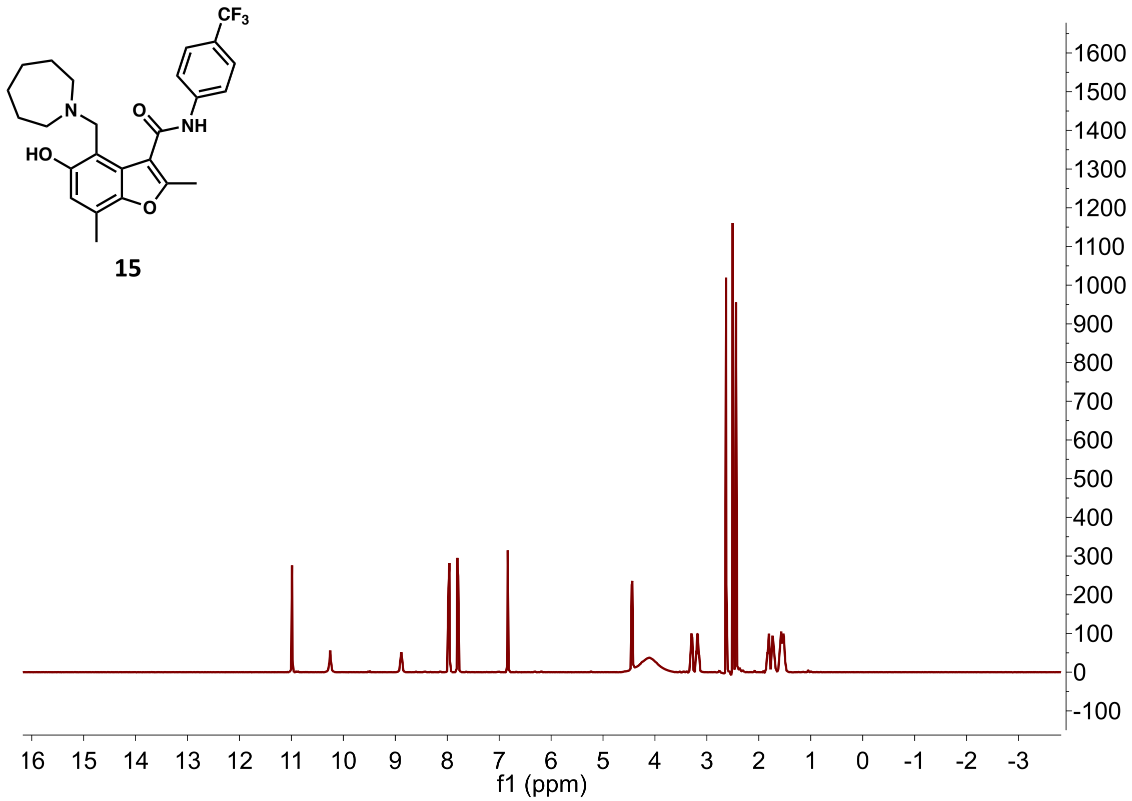

Supplementary Figure 40: ^{1}H NMR of **15**

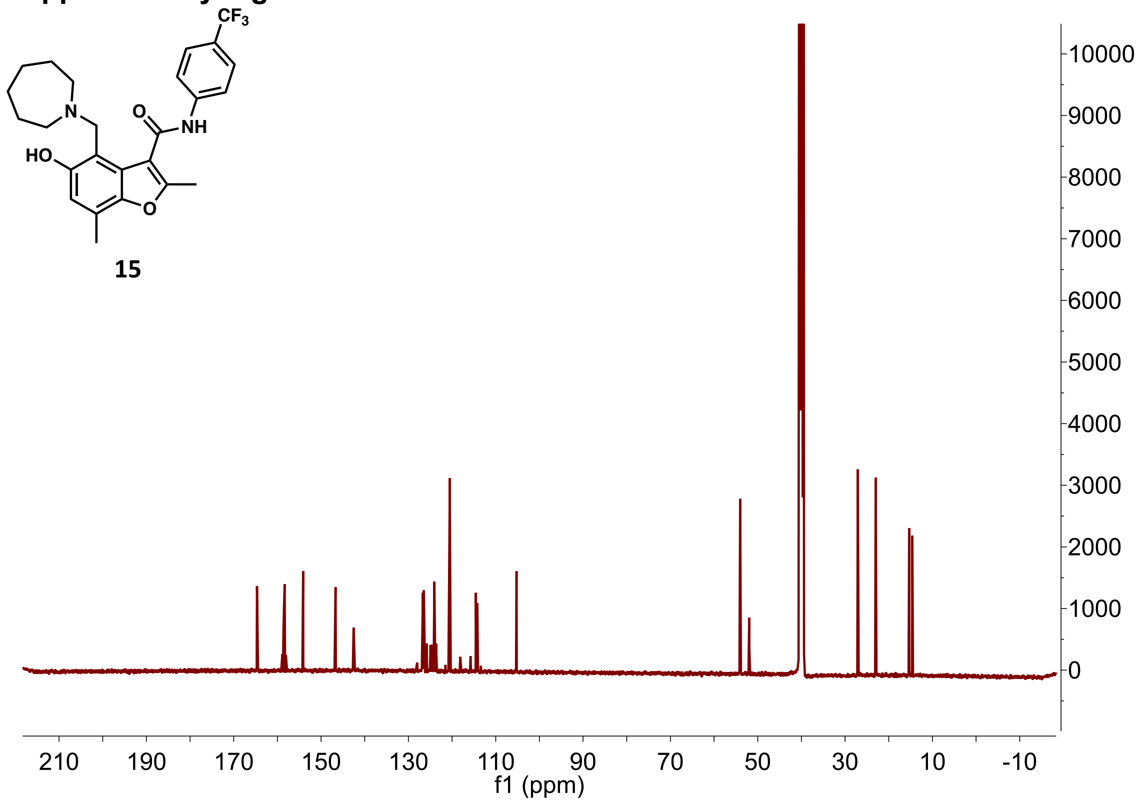

Supplementary Figure 41: ^{13}C NMR of **15**

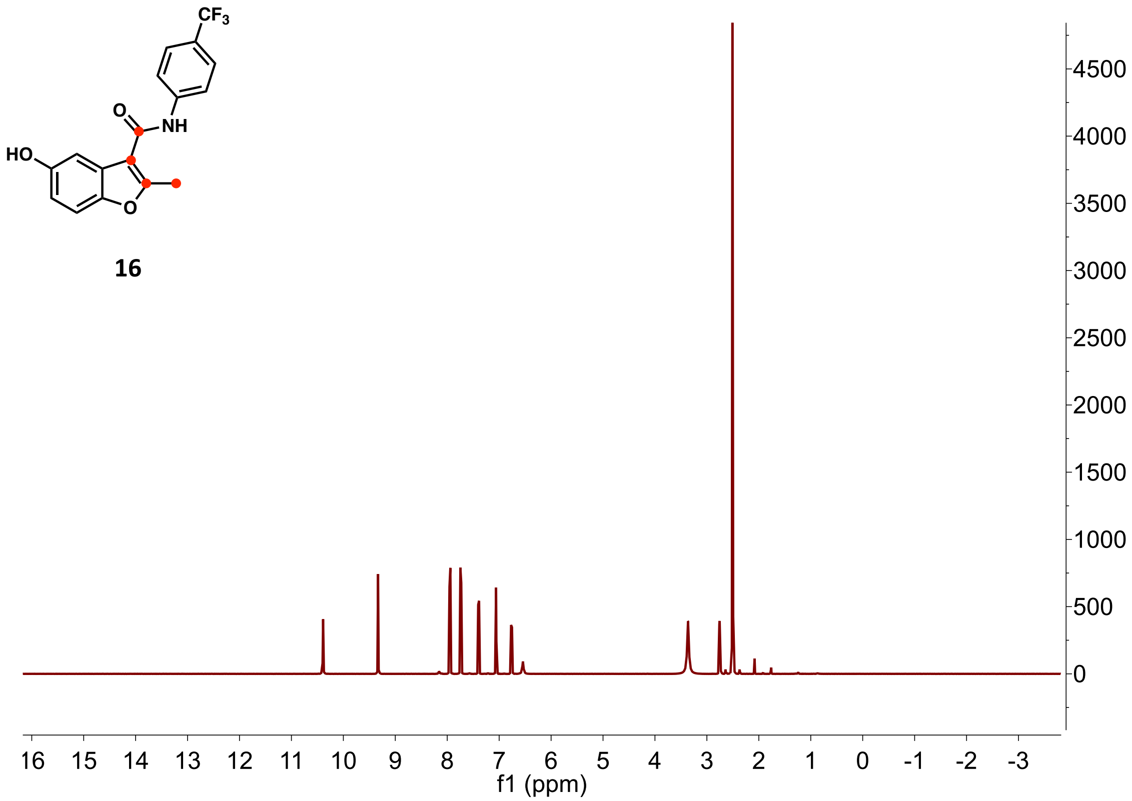

Supplementary Figure 42: ^{1}H NMR of **16**

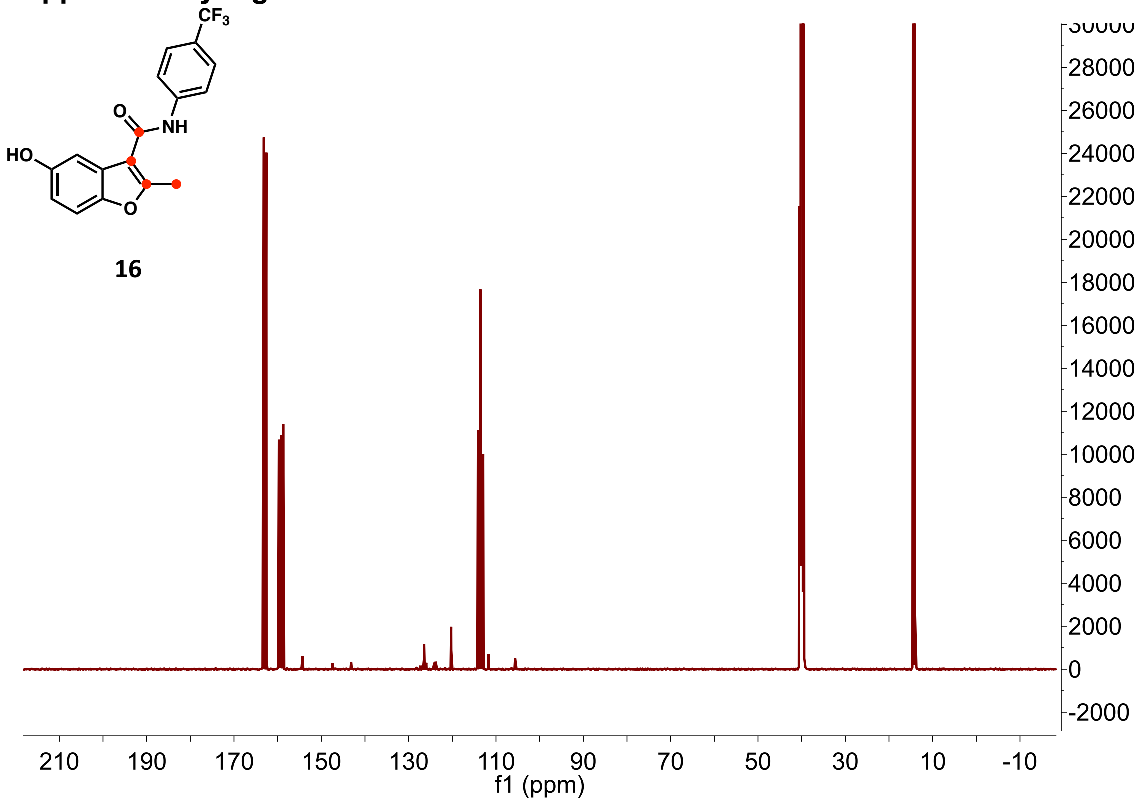

Supplementary Figure 43: ^{13}C NMR of **16**

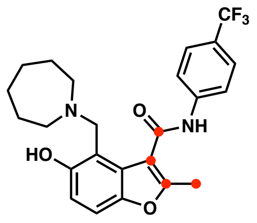

17

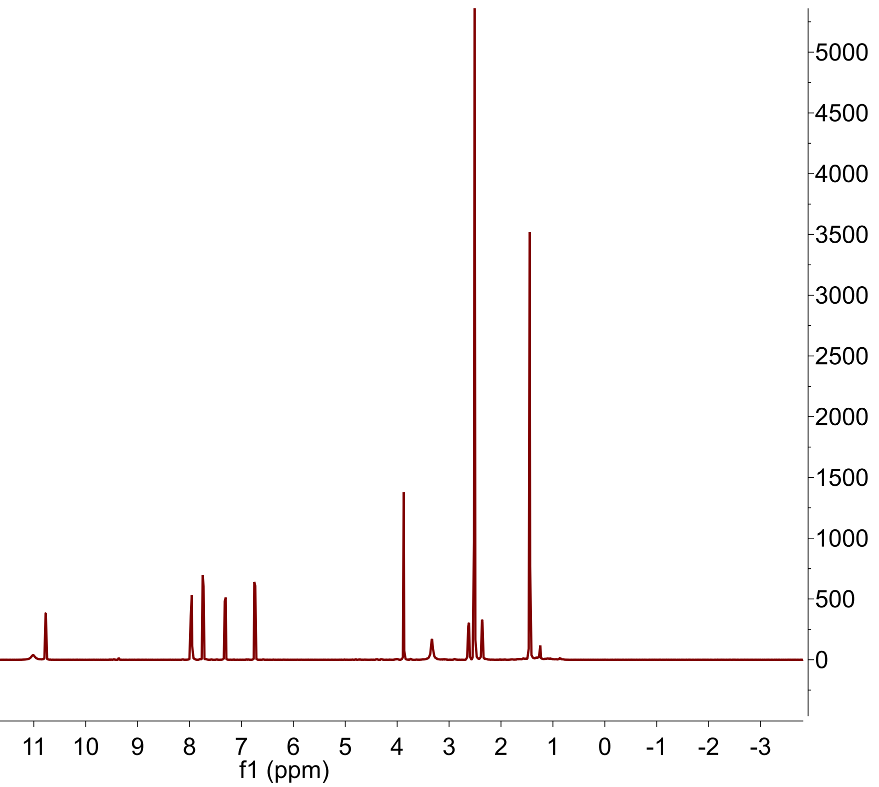

Supplementary Figure 44: ^{1}H NMR of **17**

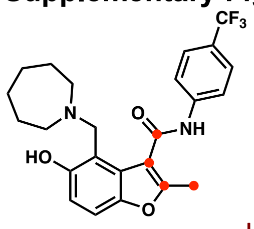

17

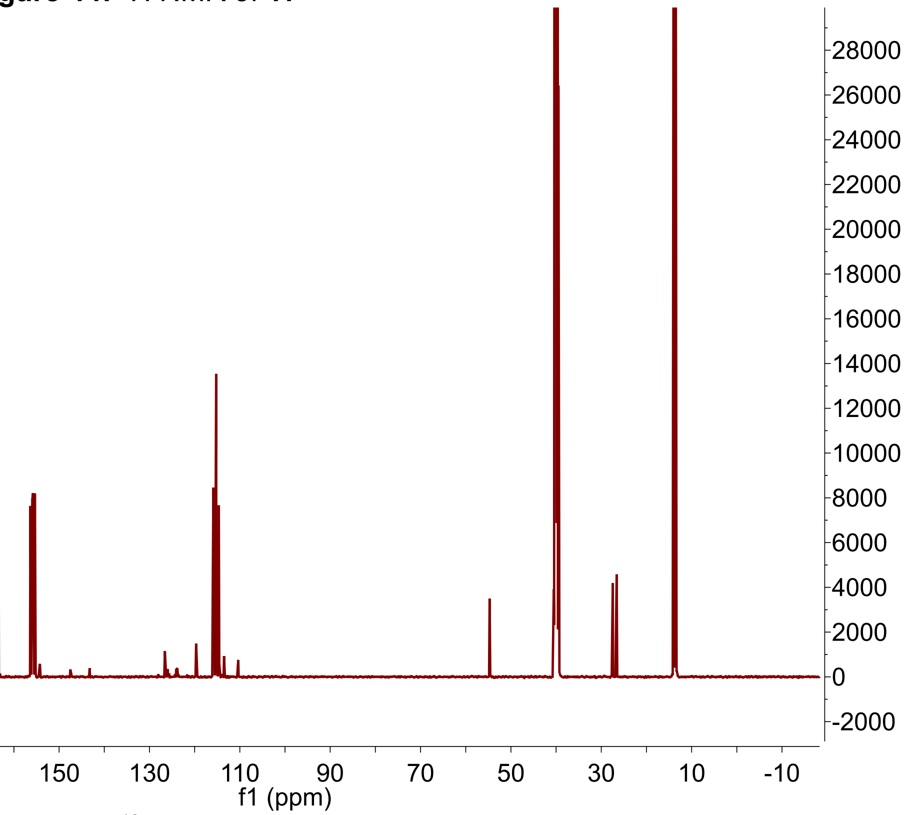

Supplementary Figure 45: ^{13}C NMR of **17**

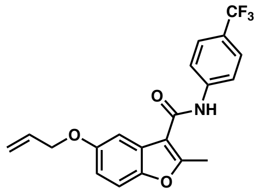

18

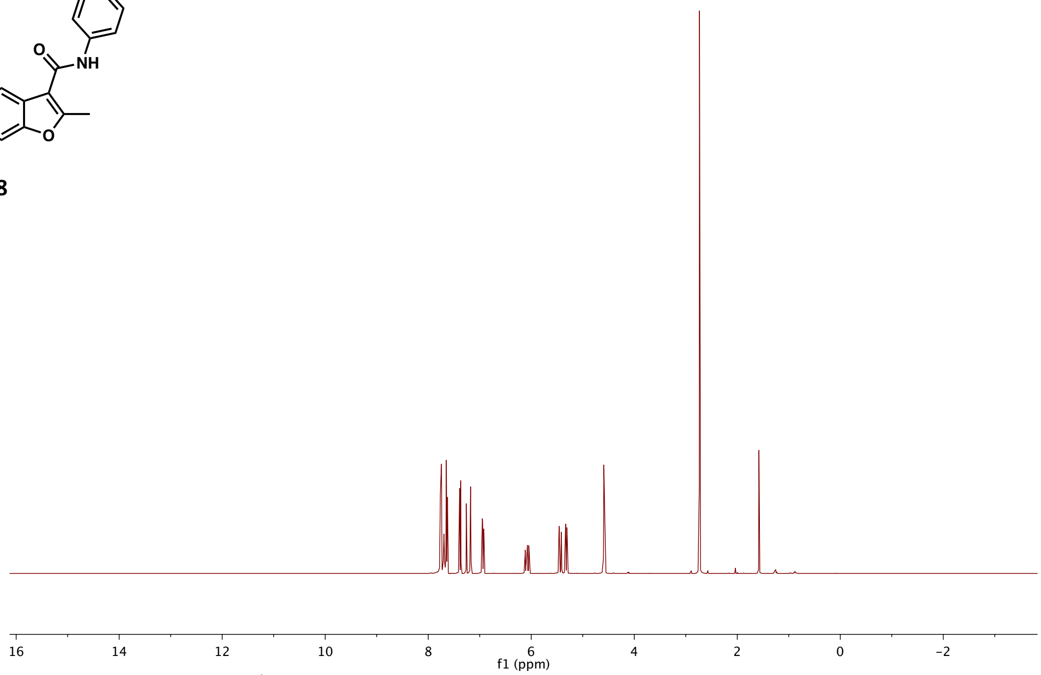

Supplementary Figure 46: ^{1}H NMR of **18**

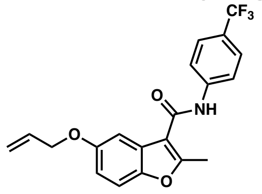

18

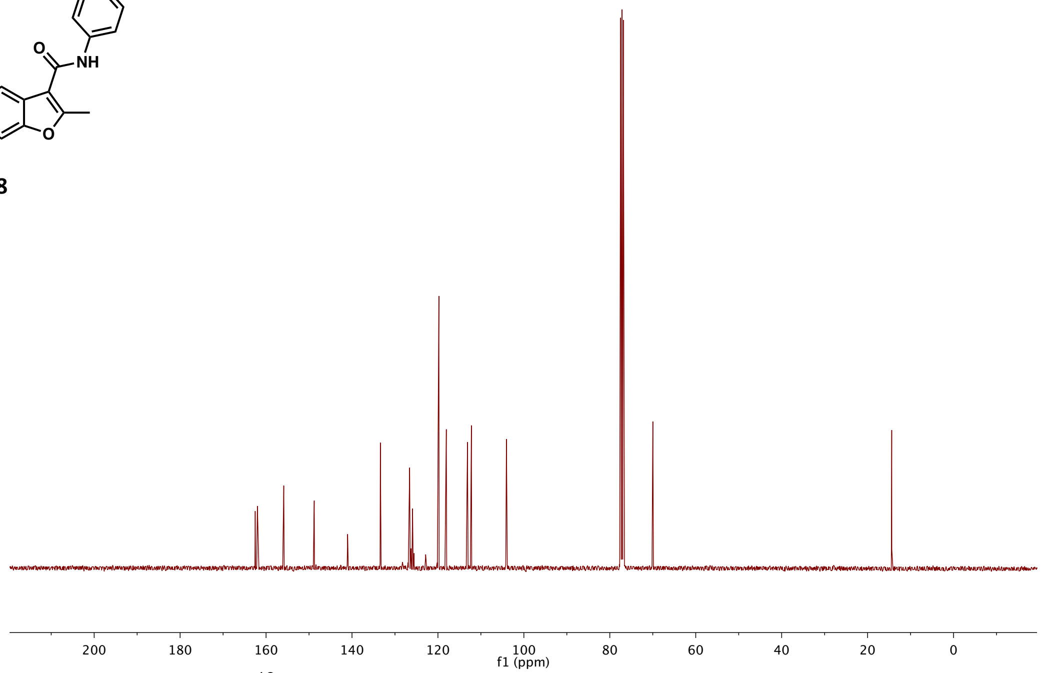

Supplementary Figure 47: ^{13}C NMR of **18**

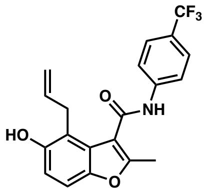

19

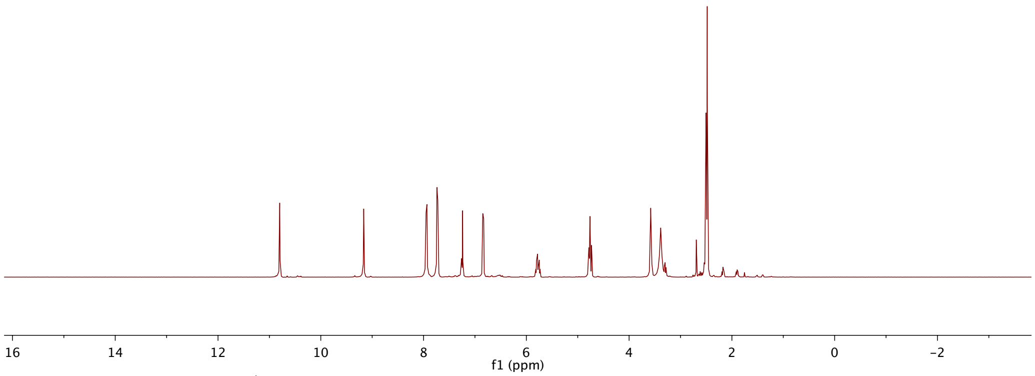

Supplementary Figure 48: ^{1}H NMR of **19**

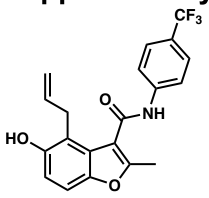

19

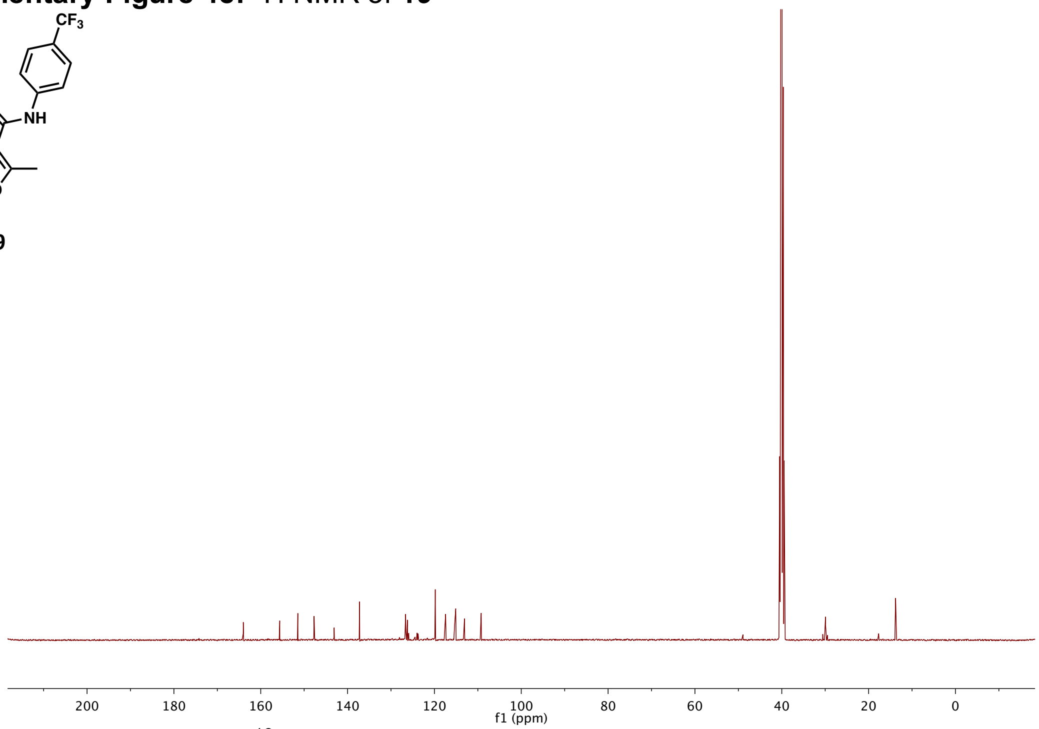

Supplementary Figure 49: ^{13}C NMR of **19**

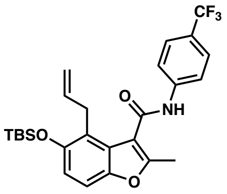

20

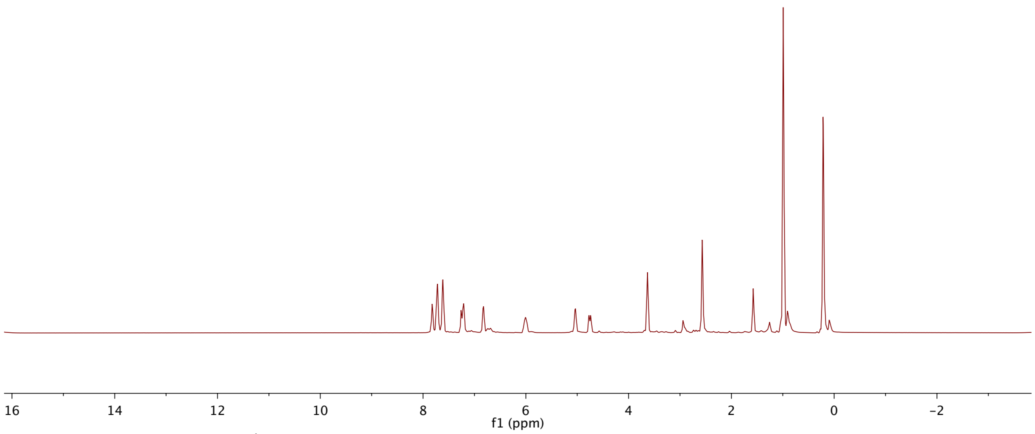

Supplementary Figure 50: ^{1}H NMR of **20**

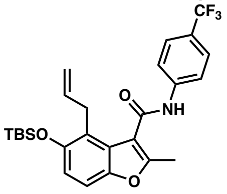

20

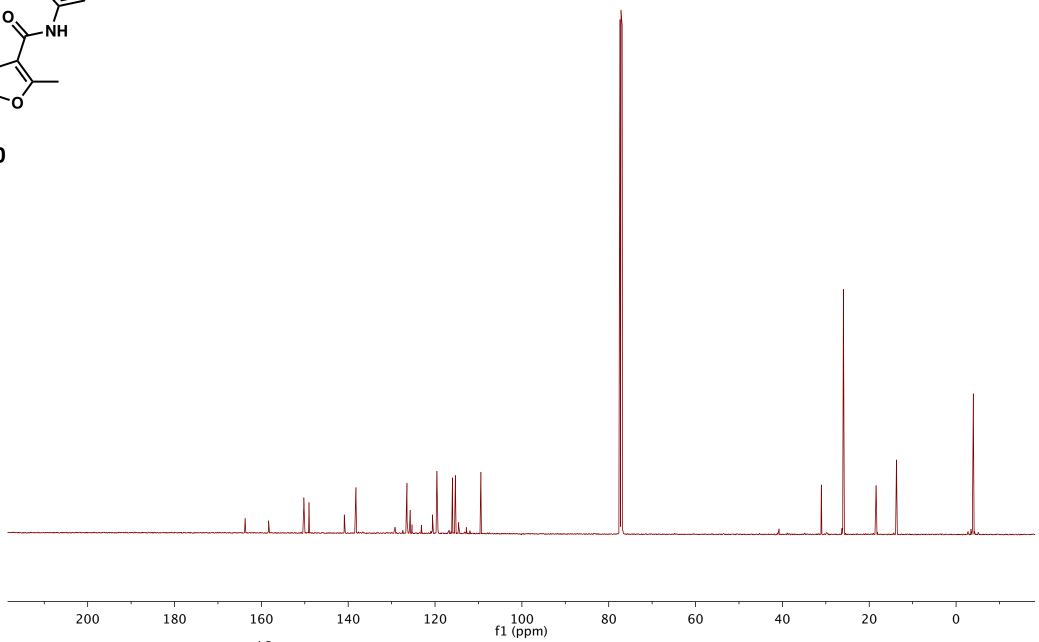

Supplementary Figure 51: ^{13}C NMR of **20**

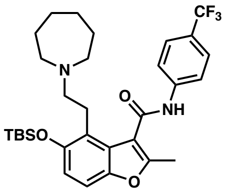

21

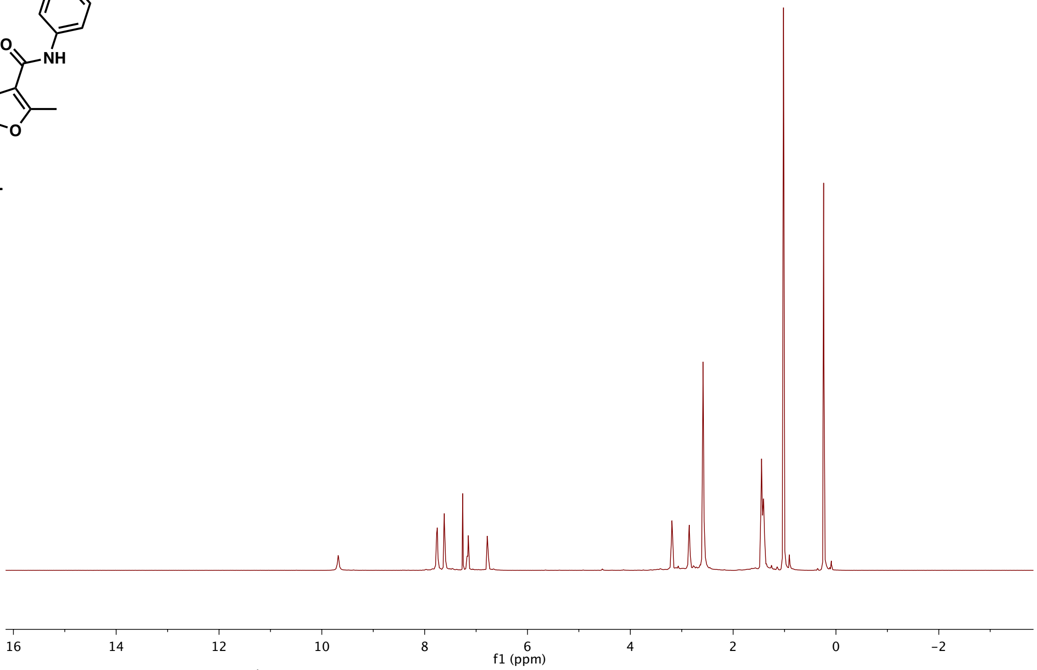

Supplementary Figure 52: ^{1}H NMR of **21**

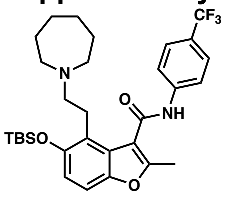

21

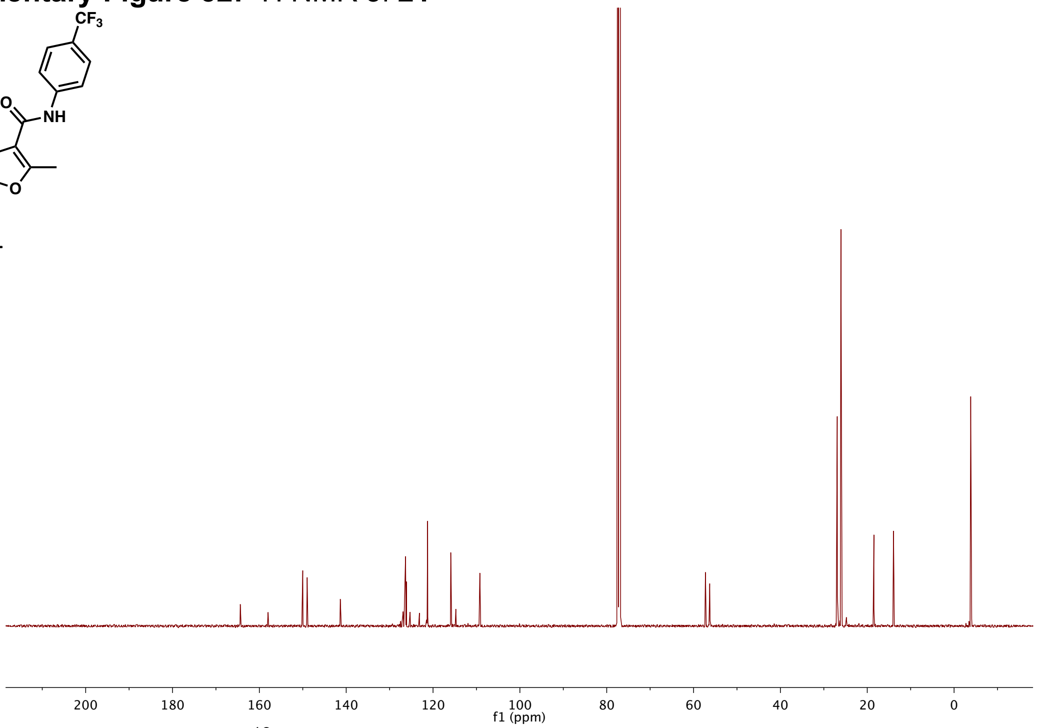

Supplementary Figure 53: ^{13}C NMR of **21**

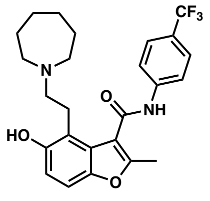

22

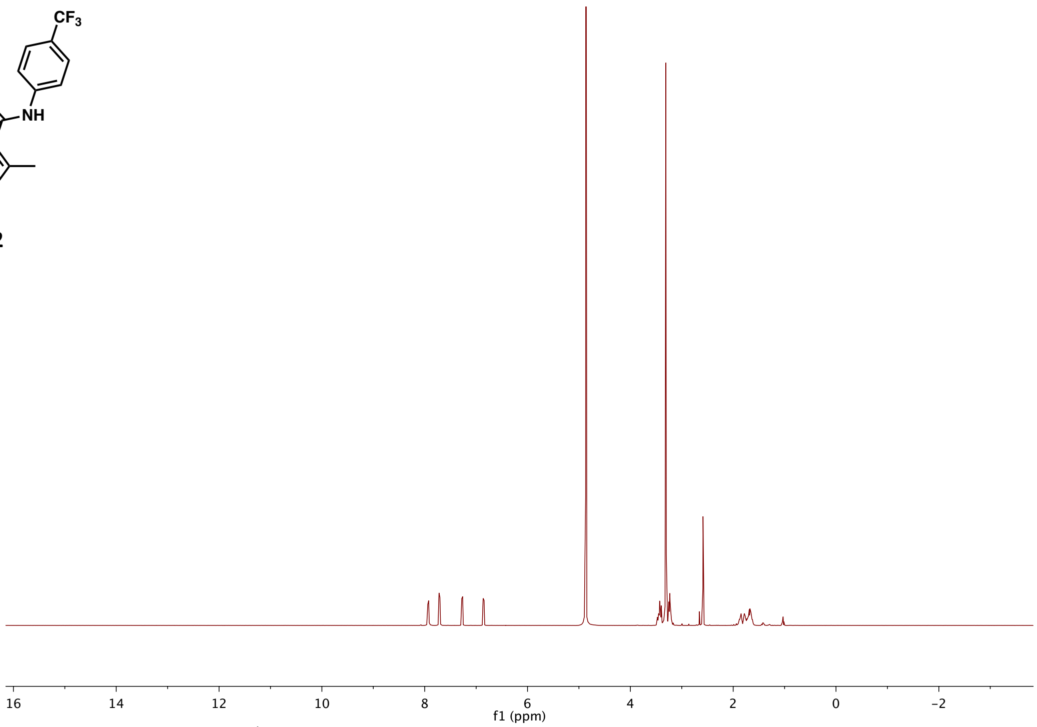

Supplementary Figure 54: ^{1}H NMR of **22**

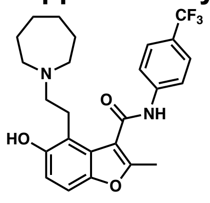

22

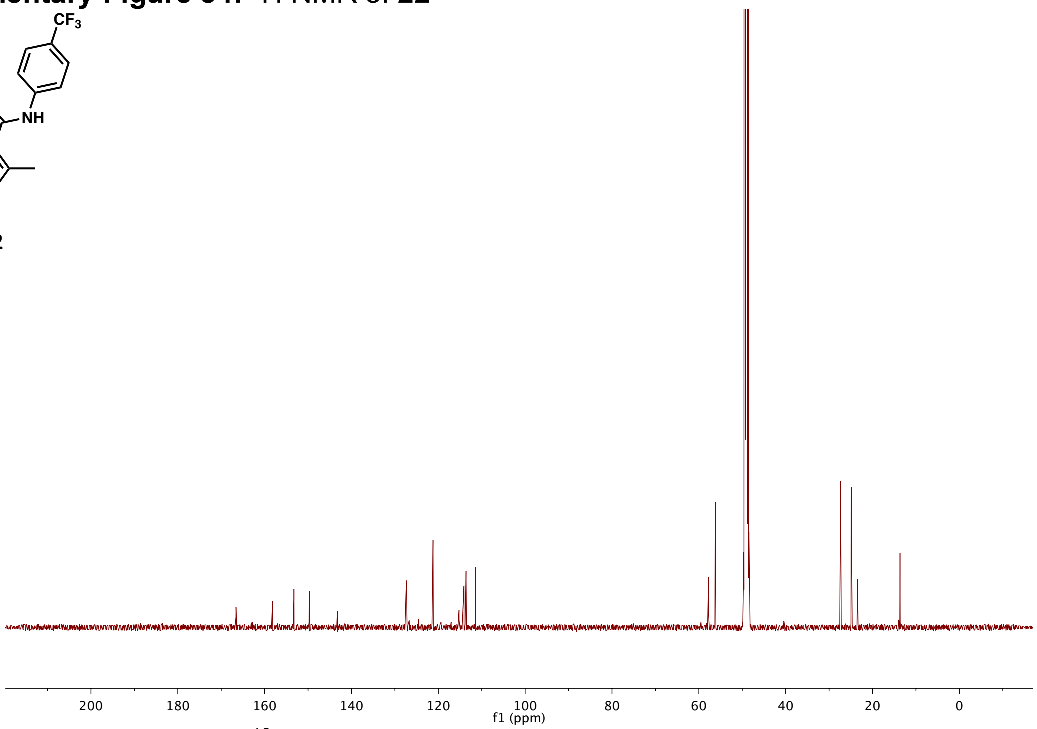

Supplementary Figure 55: ^{13}C NMR of **22**

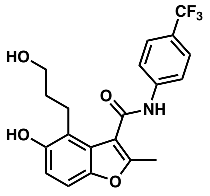

23

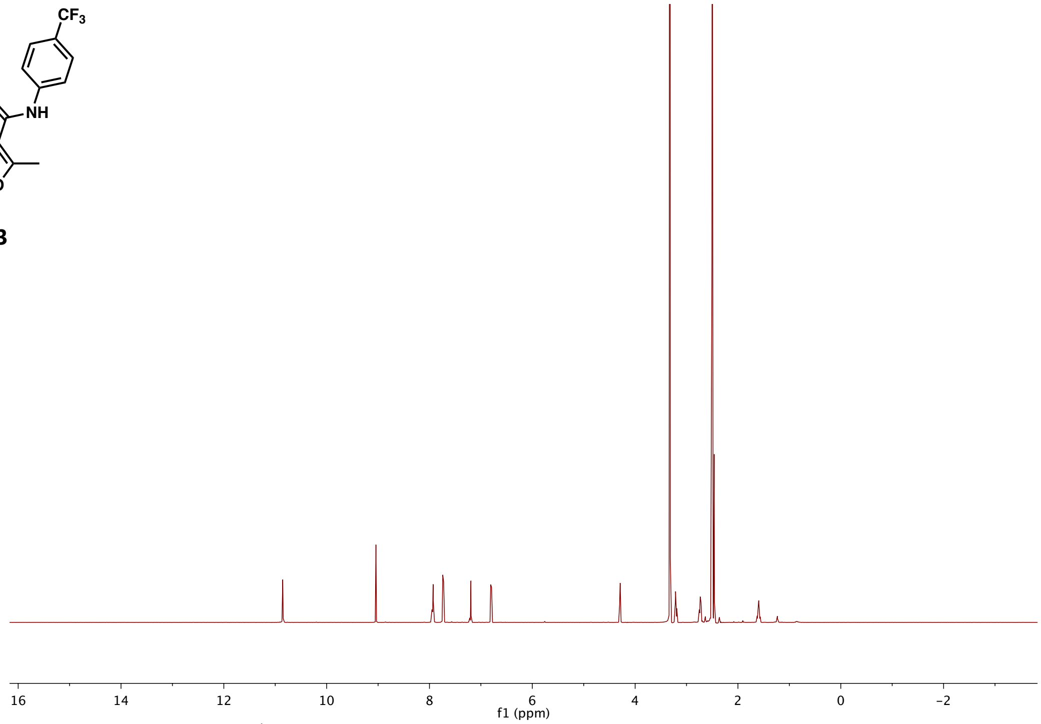

Supplementary Figure 56: ^{1}H NMR of **23**

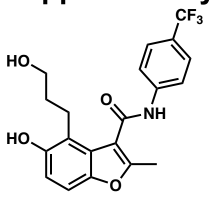

23

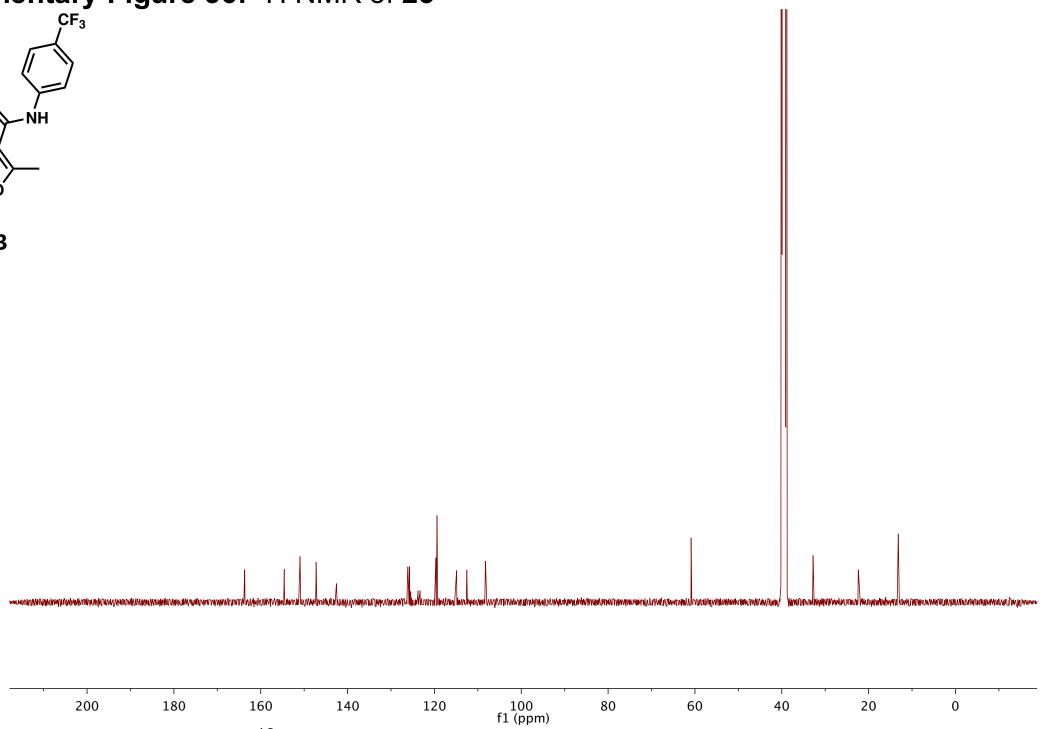

Supplementary Figure 57: ^{13}C NMR of **23**

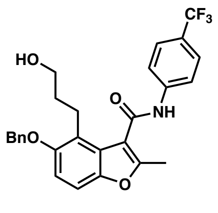

24

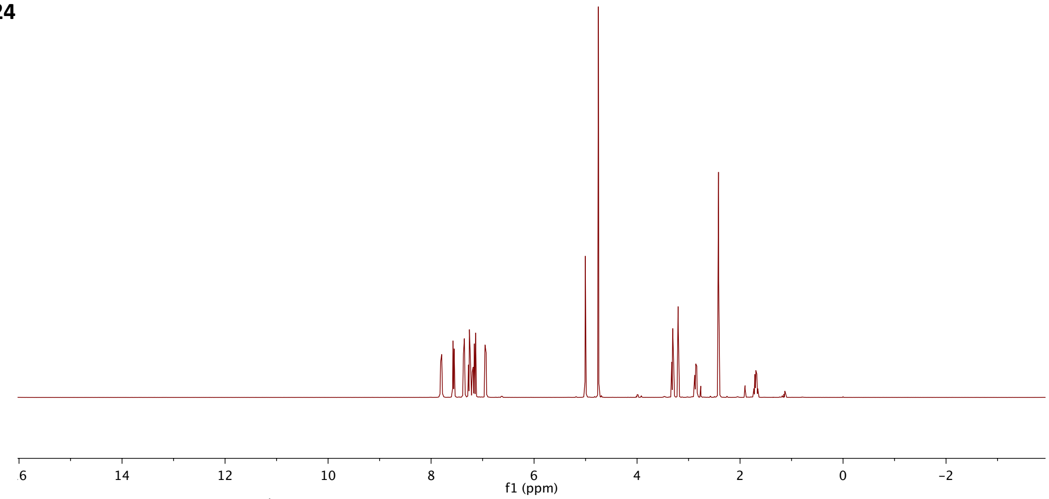

Supplementary Figure 58: ^{1}H NMR of **24**

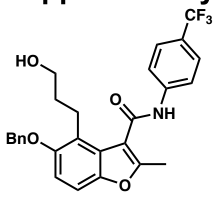

24

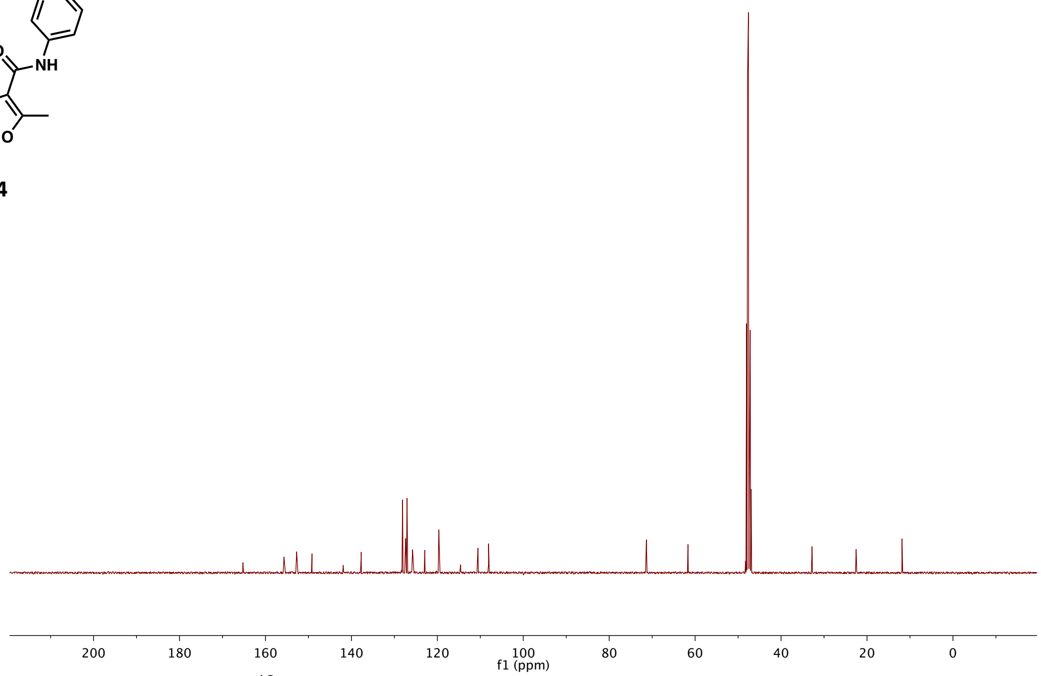

Supplementary Figure 59: ^{13}C NMR of **24**

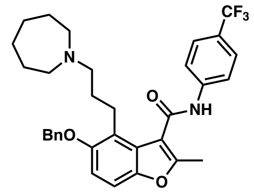

25

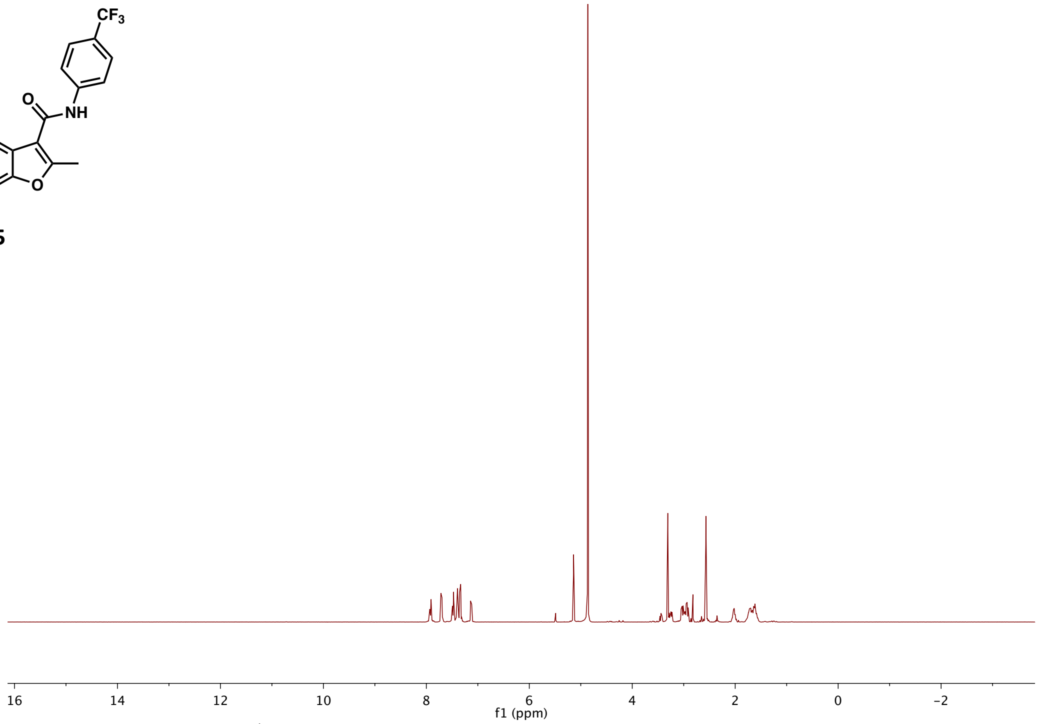

Supplementary Figure 60: ^{1}H NMR of **25**

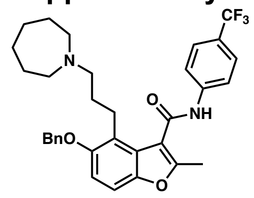

25

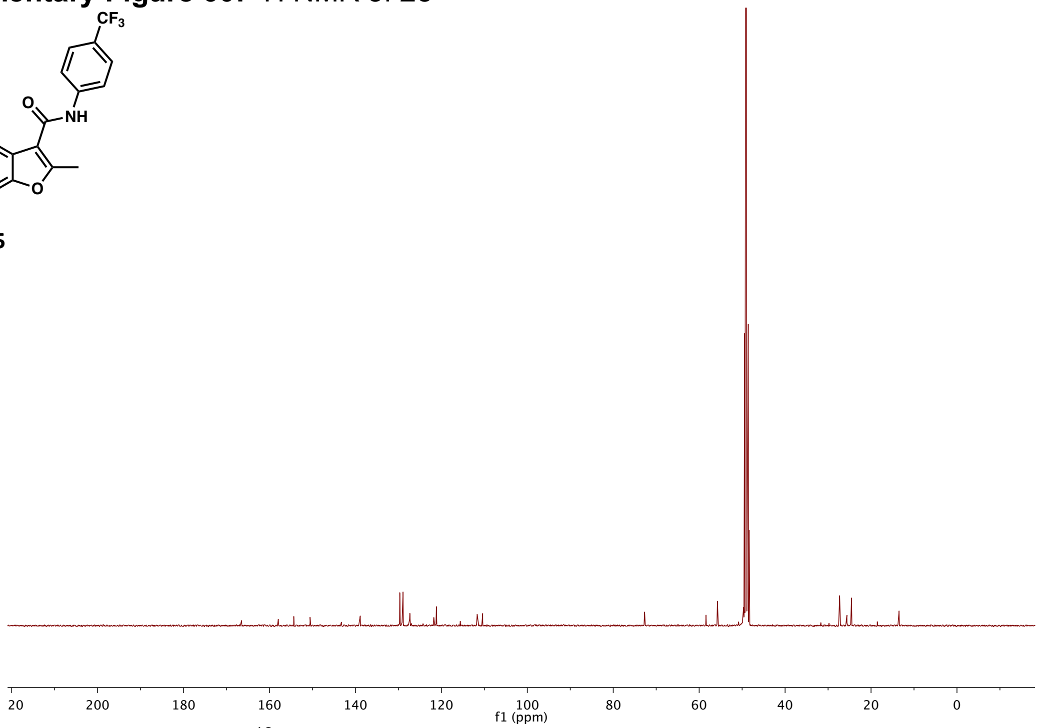

Supplementary Figure 61: ^{13}C NMR of **25**

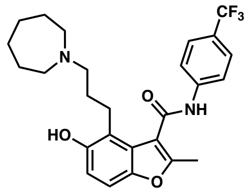

26

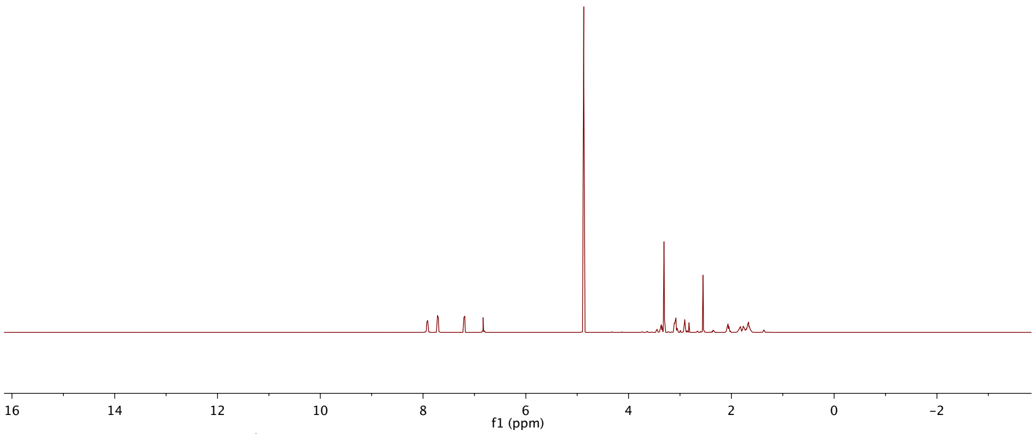

Supplementary Figure 62: ^{1}H NMR of **26**

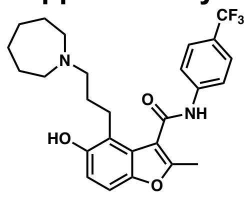

26

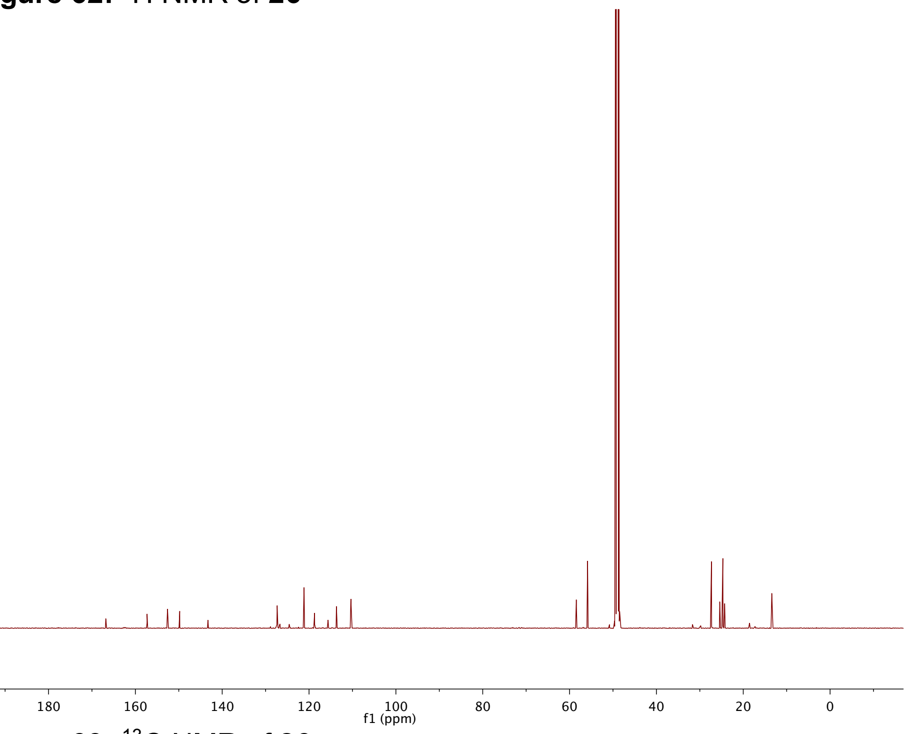

Supplementary Figure 63: ^{13}C NMR of **26**

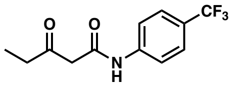

27

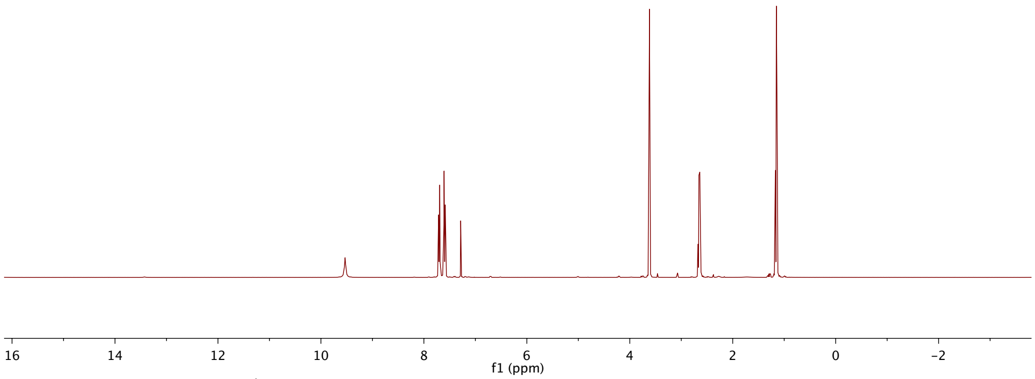

Supplementary Figure 64: ^{1}H NMR of **27**

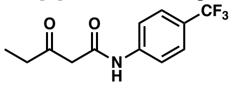

27

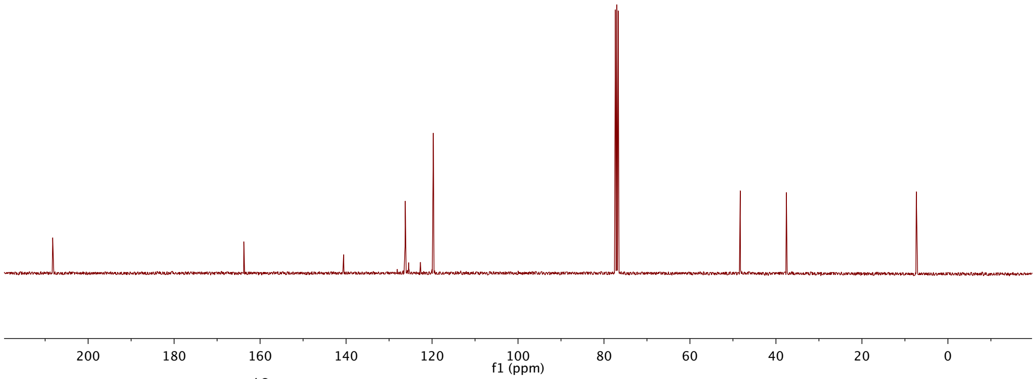

Supplementary Figure 65: ^{13}C NMR of **27**

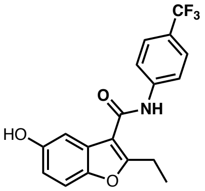

28

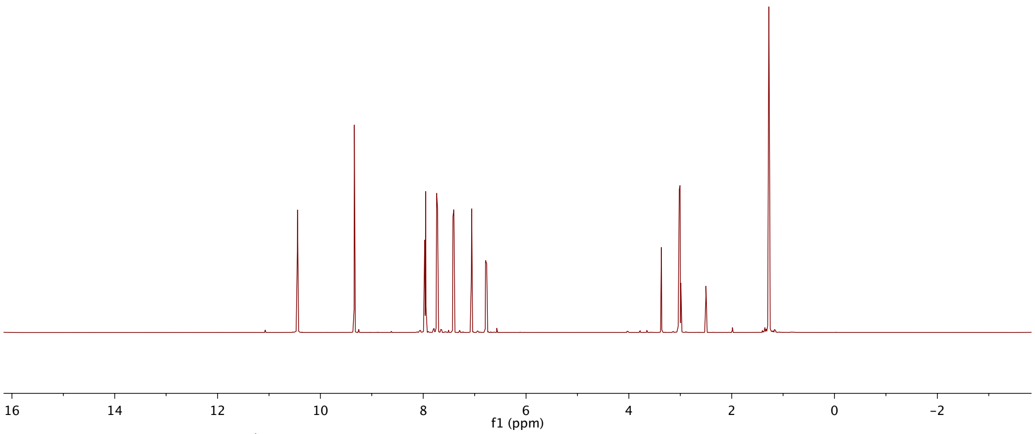

Supplementary Figure 66: ^{1}H NMR of **28**

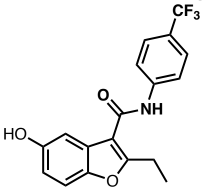

28

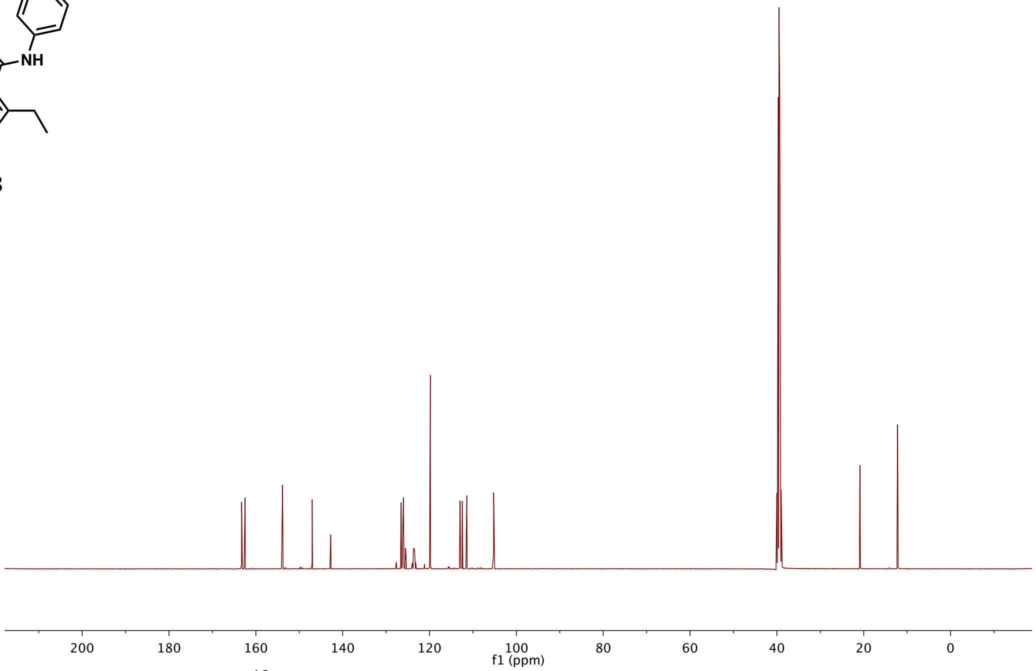

Supplementary Figure 67: ^{13}C NMR of **28**

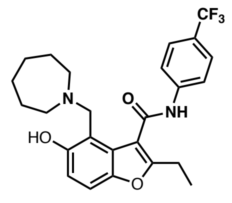

29

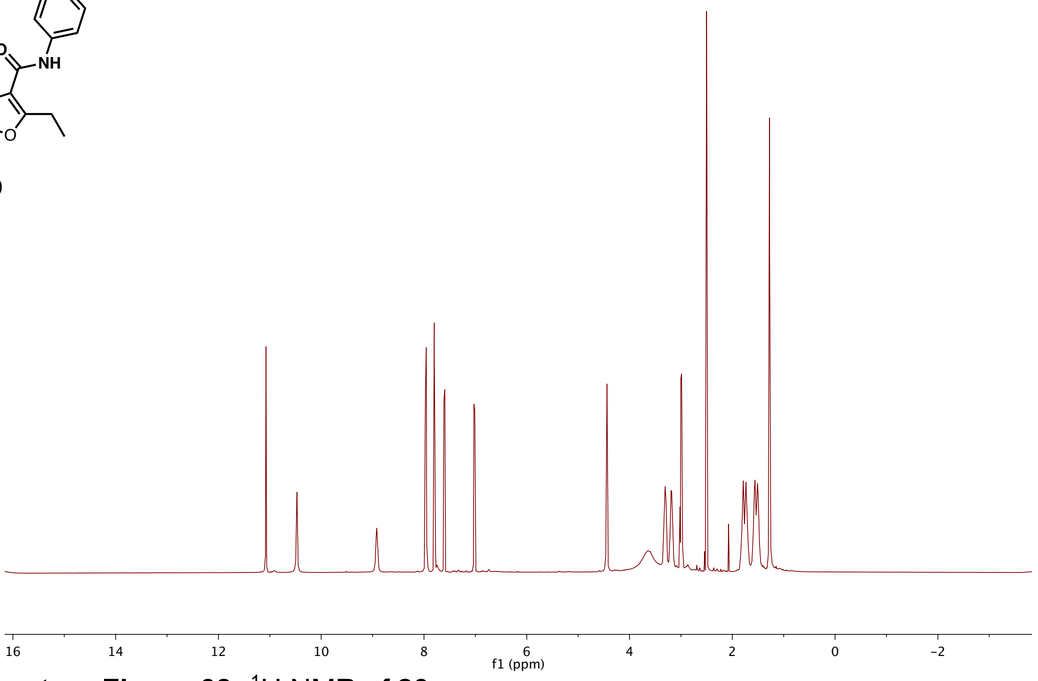

Supplementary Figure 68: ^{1}H NMR of **29**

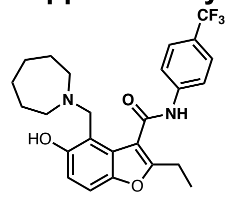

29

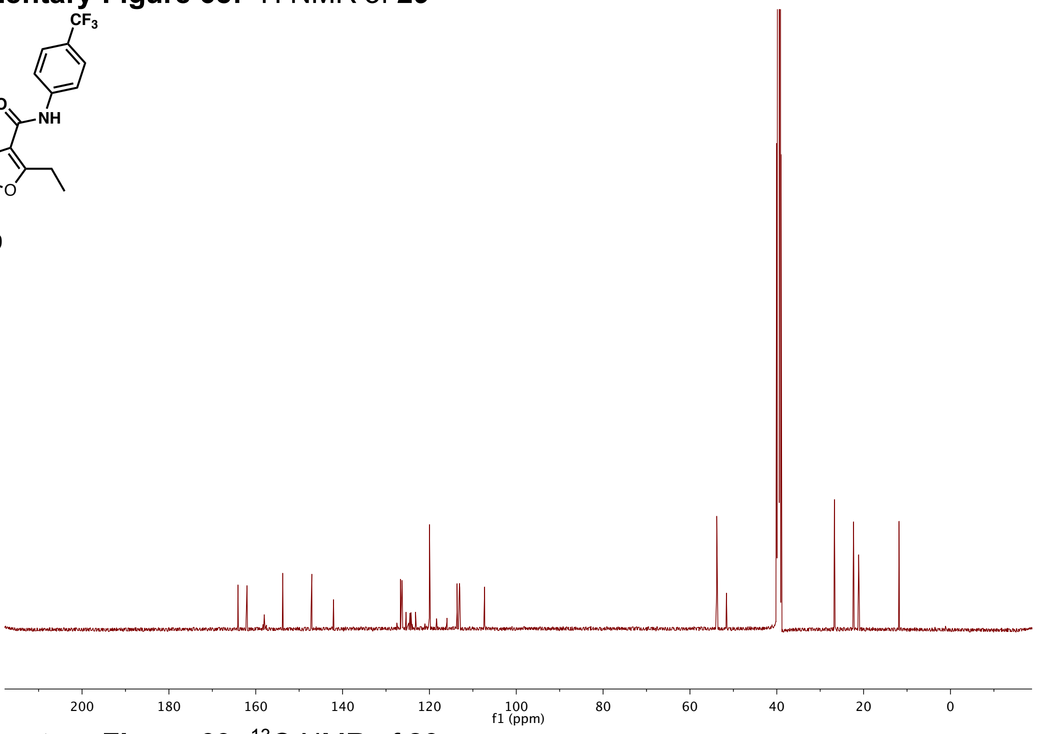

Supplementary Figure 69: ^{13}C NMR of **29**

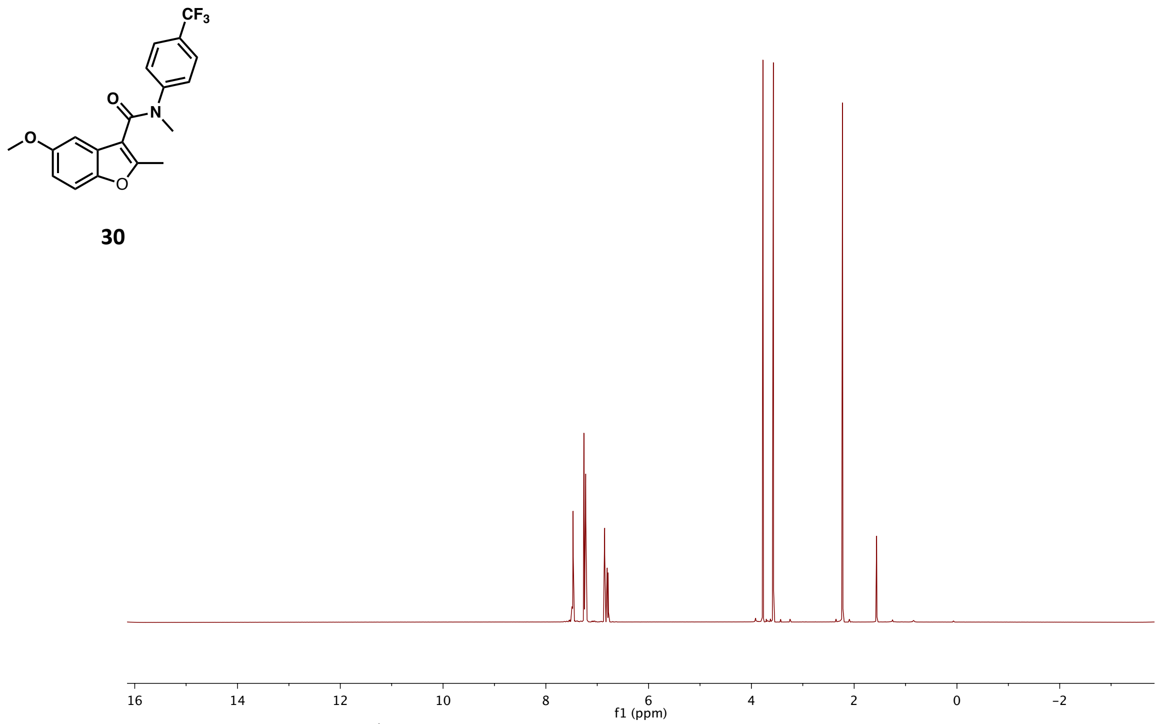

Supplementary Figure 70: ^{1}H NMR of **30**

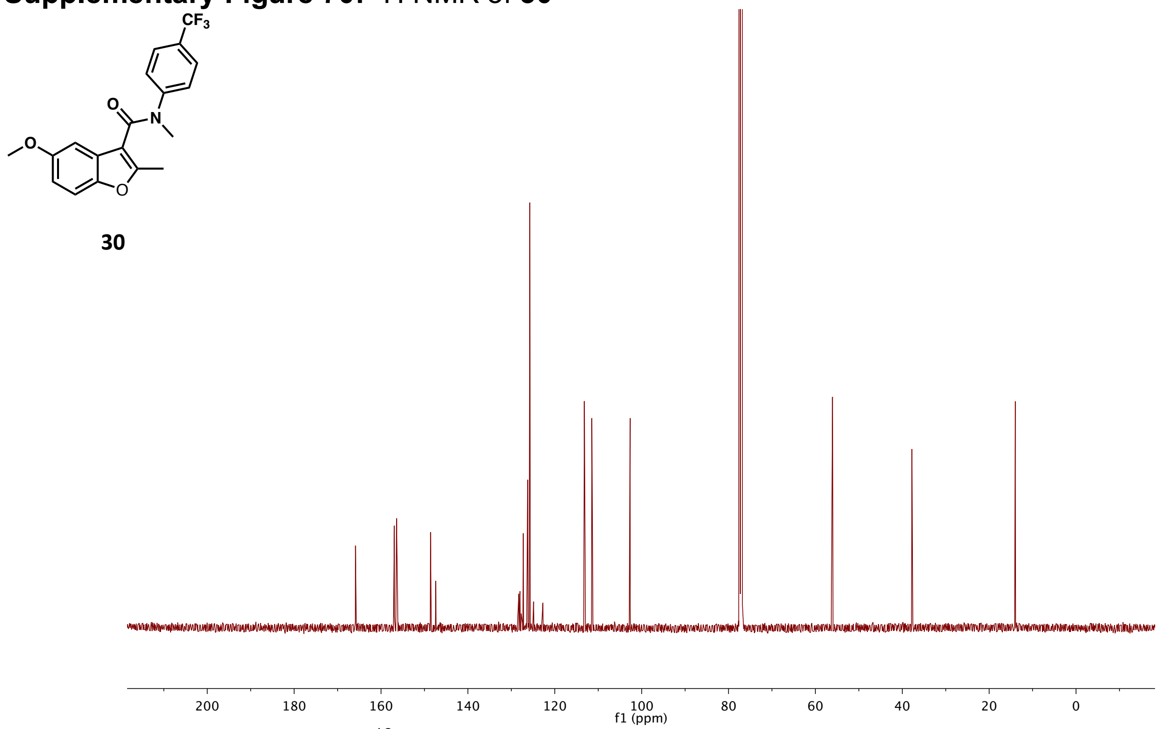

Supplementary Figure 71: ^{13}C NMR of **30**

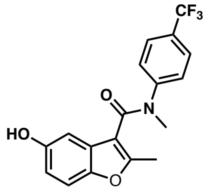

31

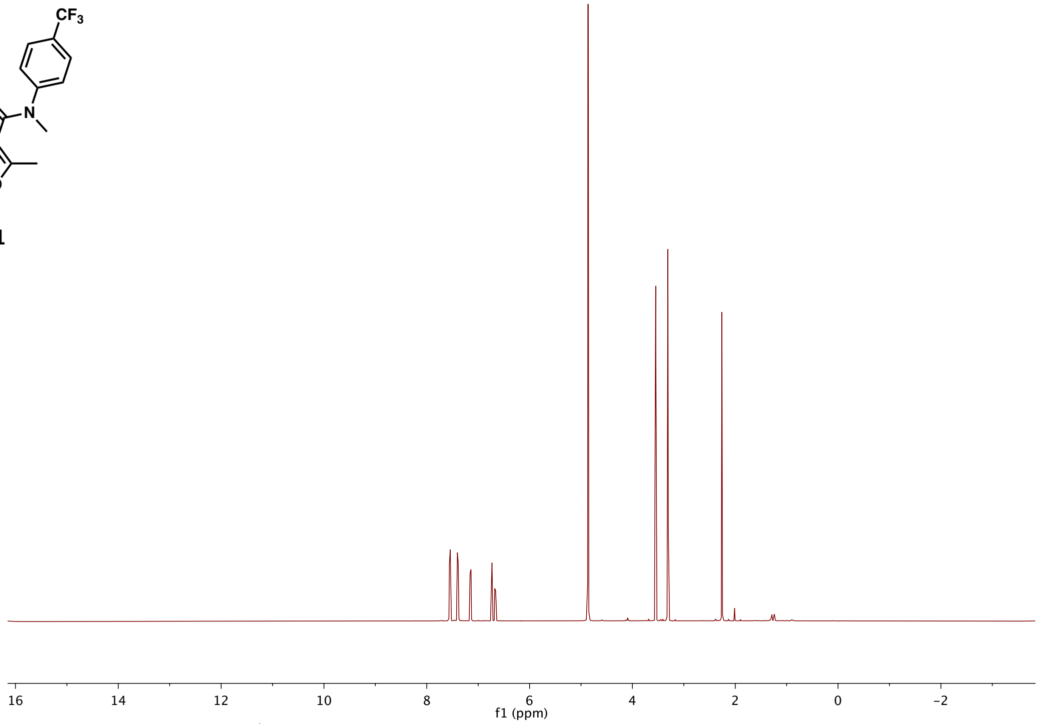

Supplementary Figure 72: ^{1}H NMR of **31**

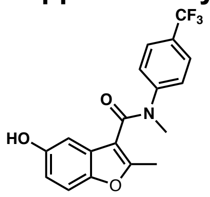

31

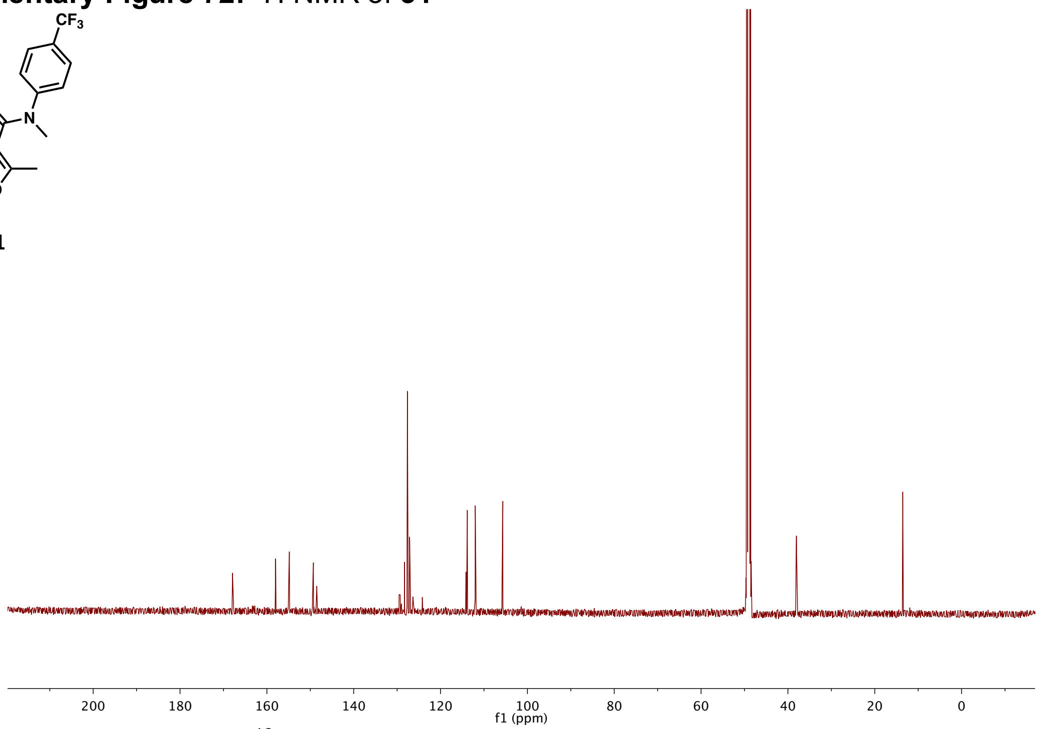

Supplementary Figure 73: ^{13}C NMR of **31**

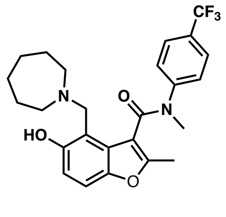

32

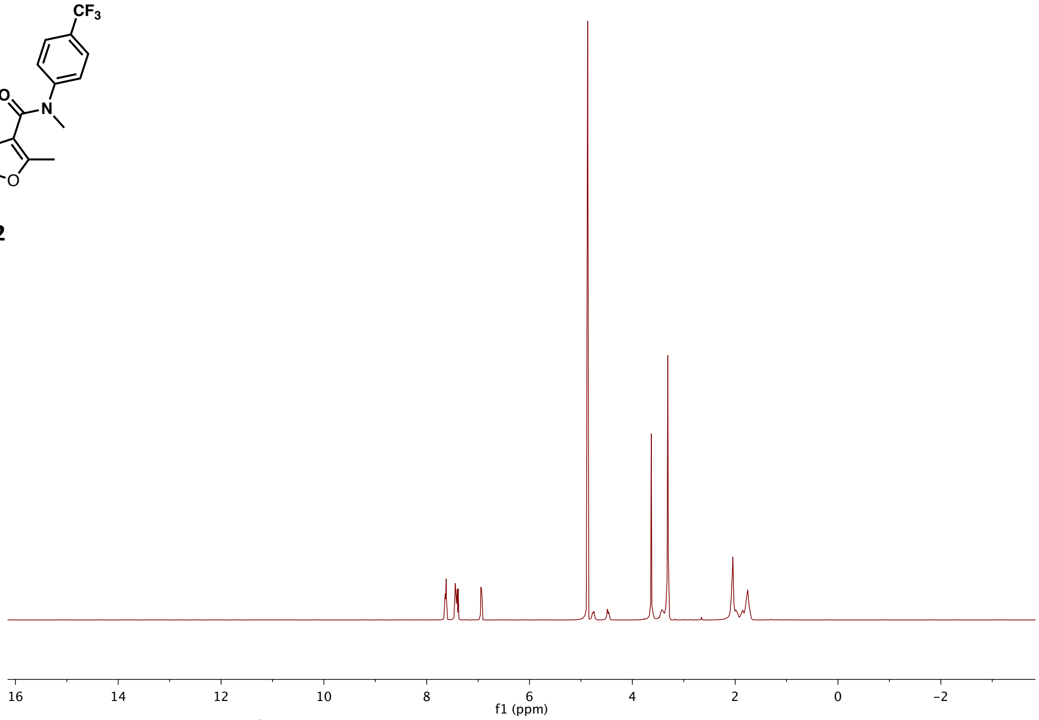

Supplementary Figure 74: ^{1}H NMR of **32**

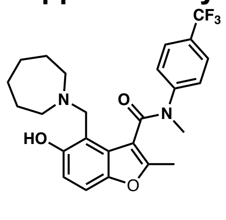

32

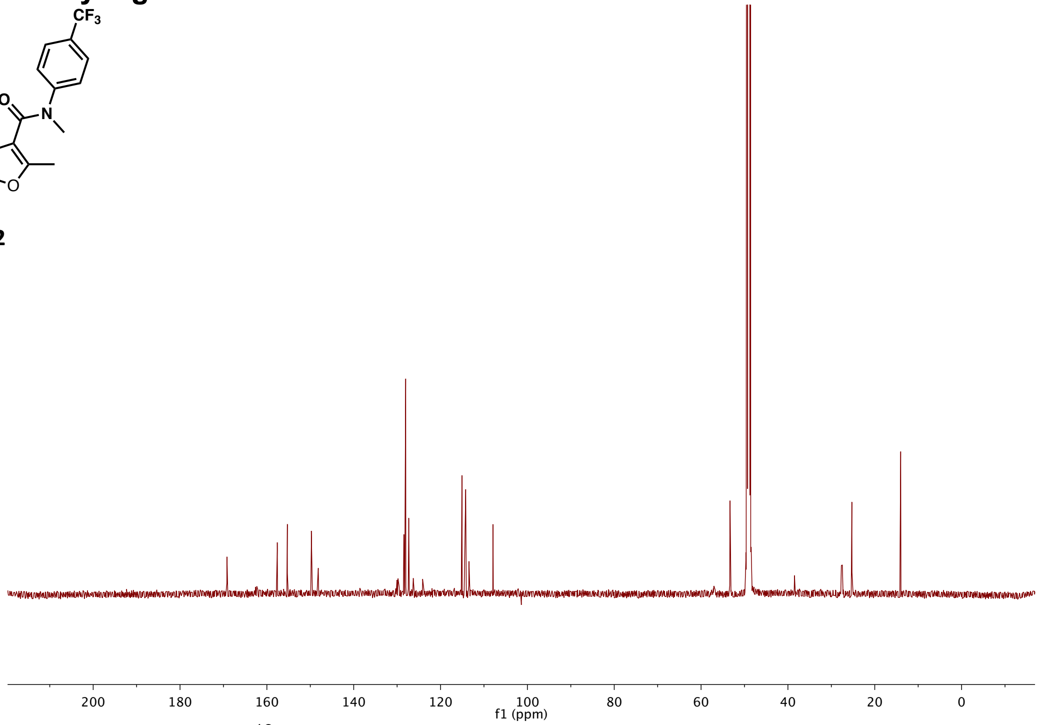

Supplementary Figure 75: ^{13}C NMR of **32**

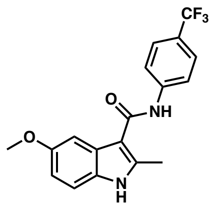

33

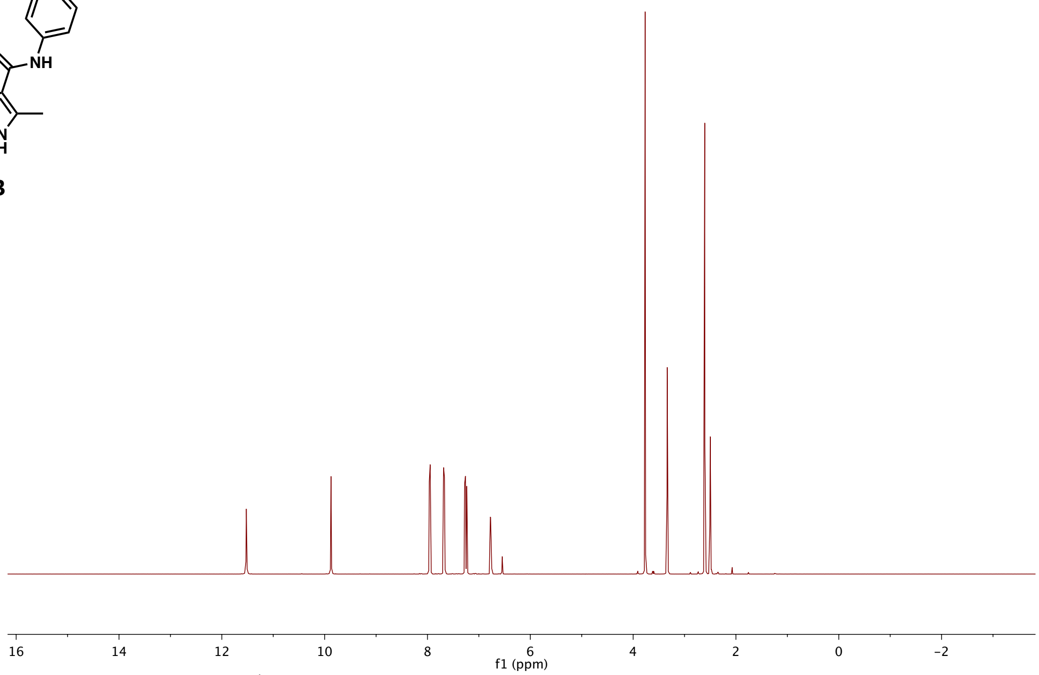

Supplementary Figure 76: ^{1}H NMR of **33**

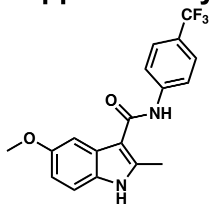

33

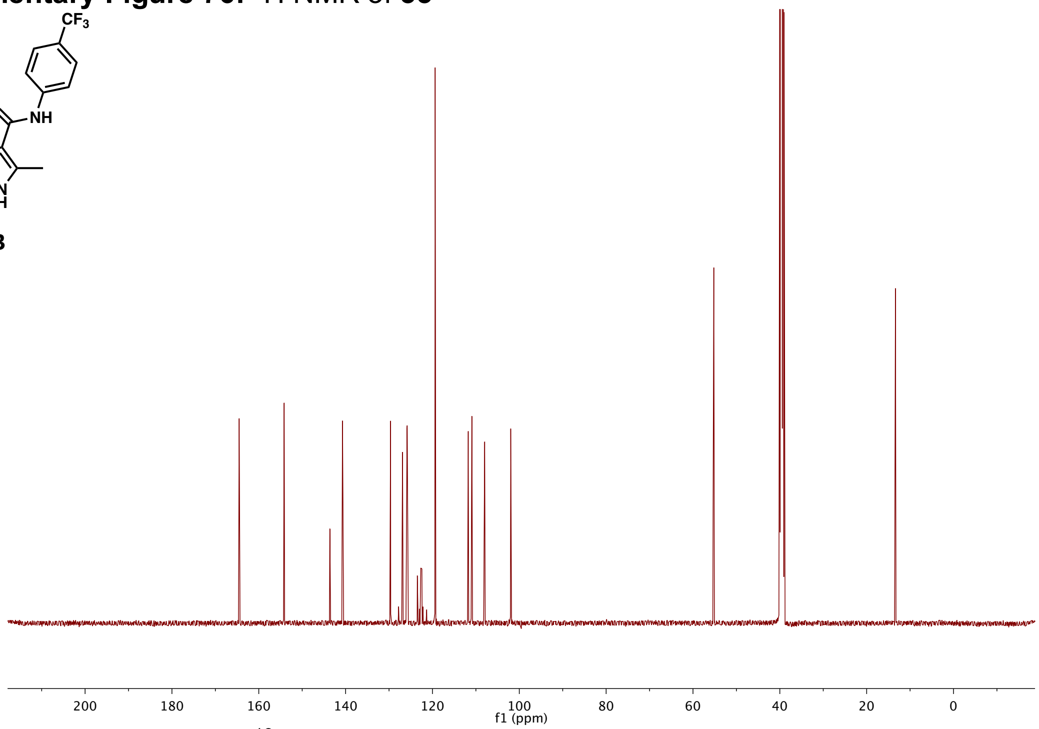

Supplementary Figure 77: ^{13}C NMR of **33**

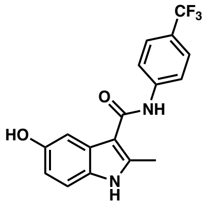

34

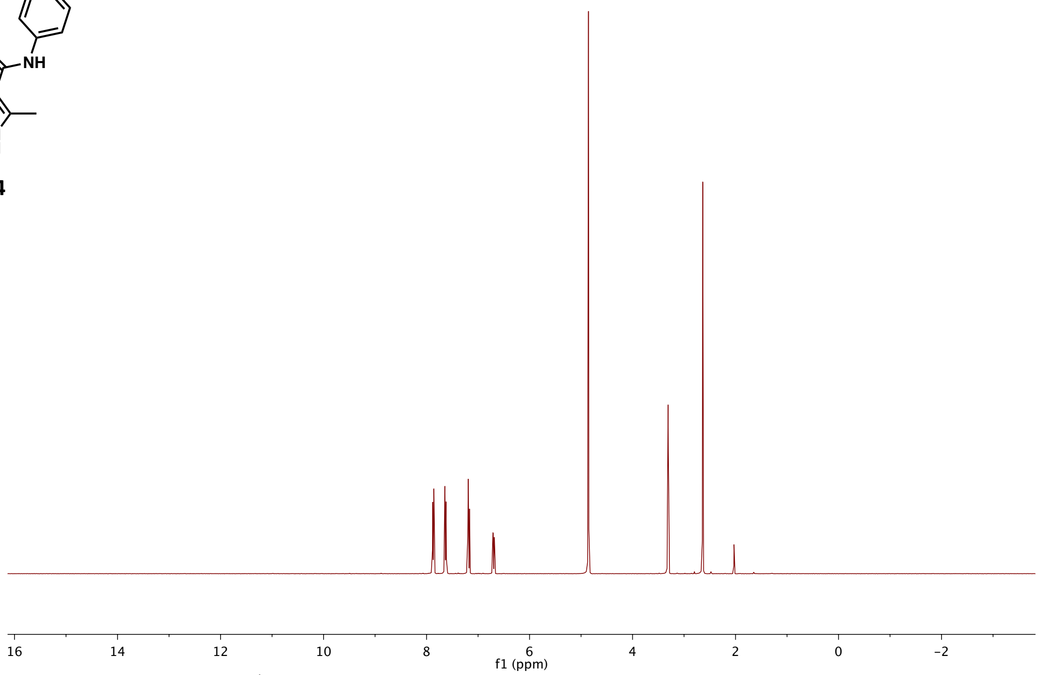

Supplementary Figure 78: ^{1}H NMR of **34**

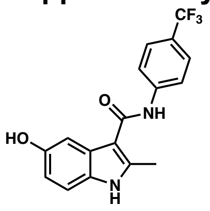

34

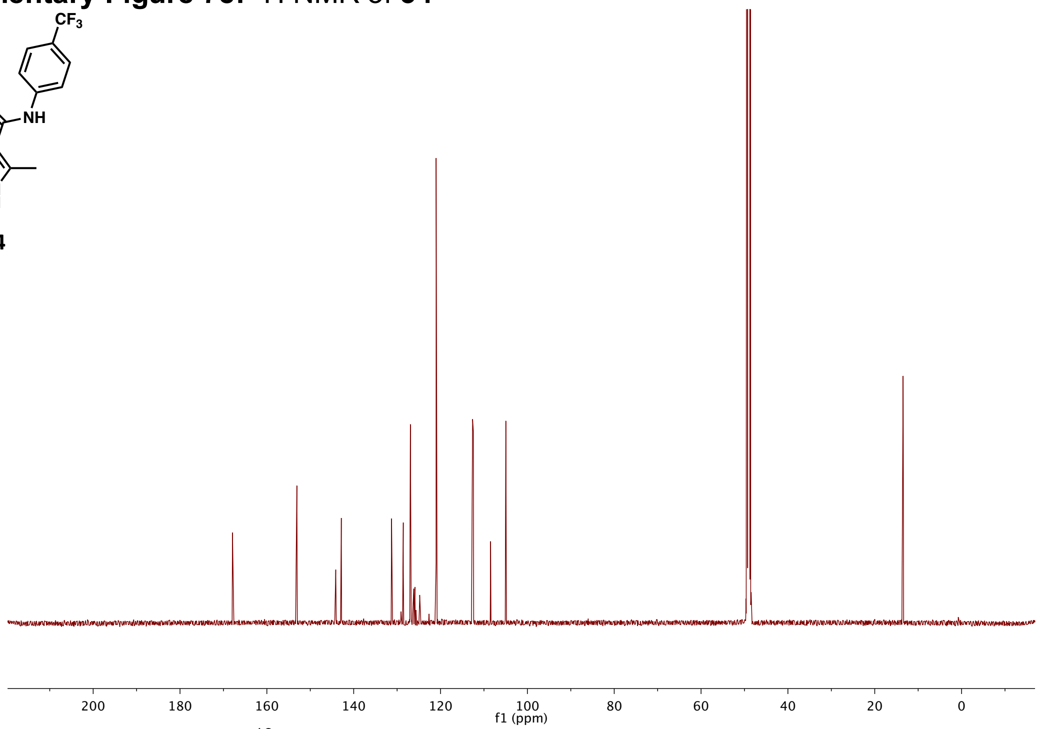

Supplementary Figure 79: ^{13}C NMR of **34**

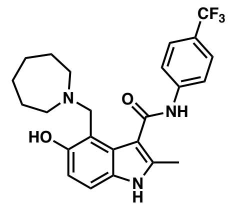

35

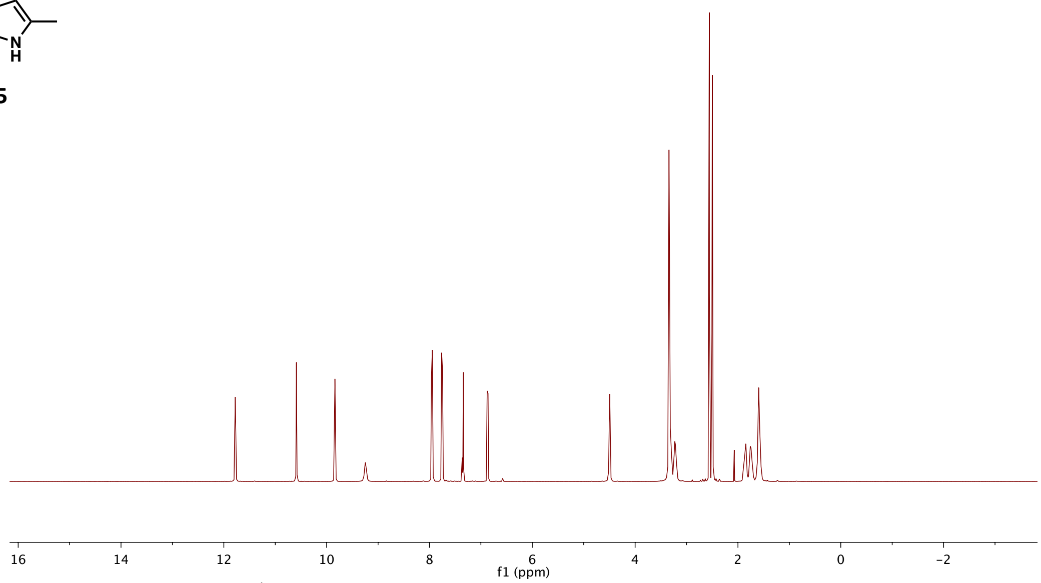

Supplementary Figure 80: ^{1}H NMR of **35**

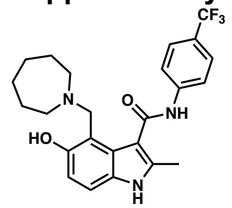

35

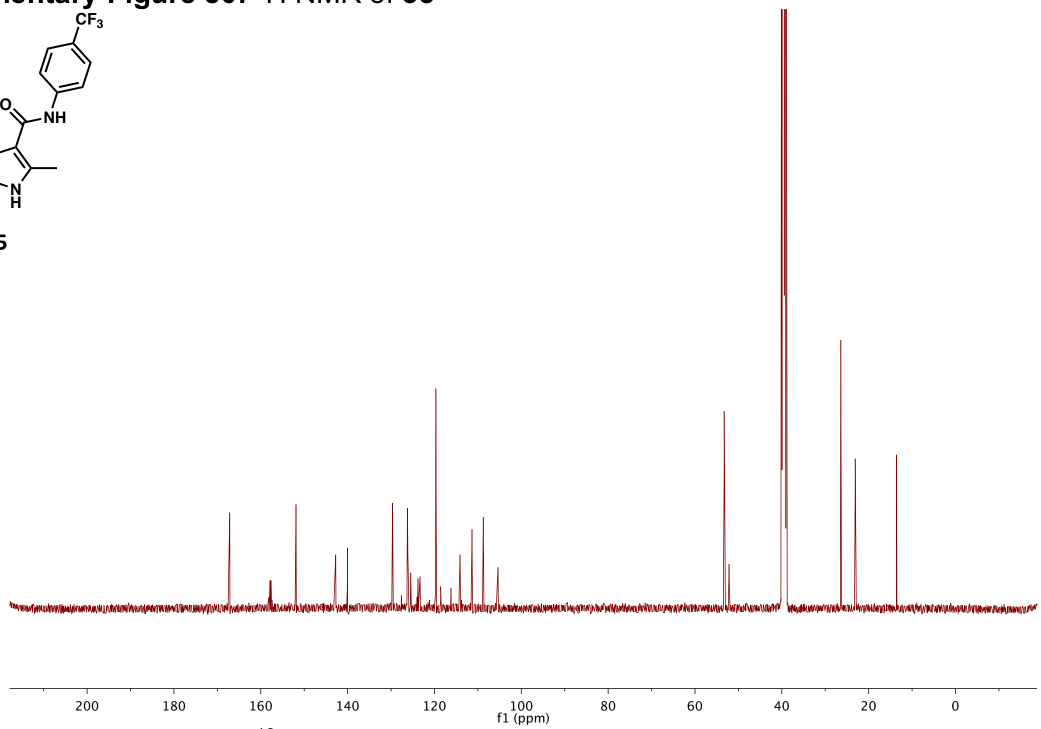

Supplementary Figure 81: ^{13}C NMR of **35**

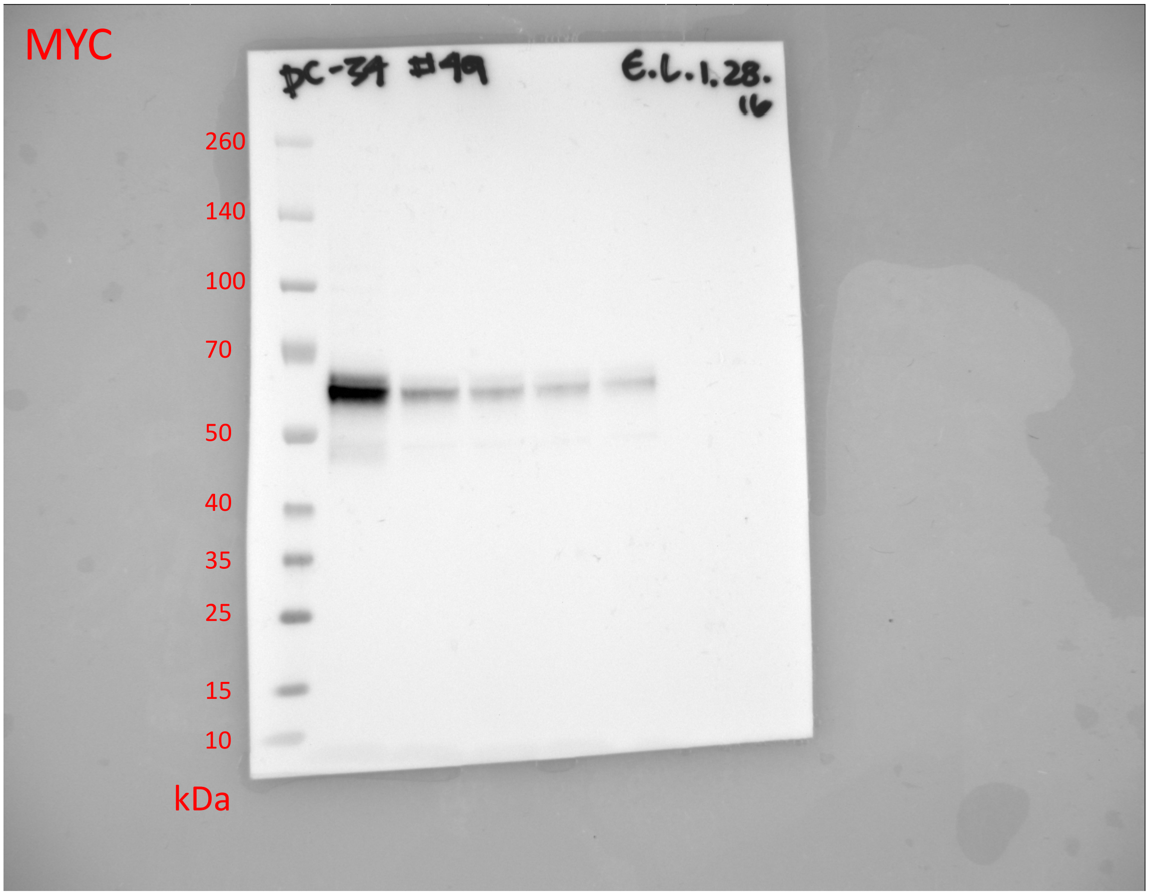

Supplementary Figure 82: Raw Western blot of MYC in Figure 3B.

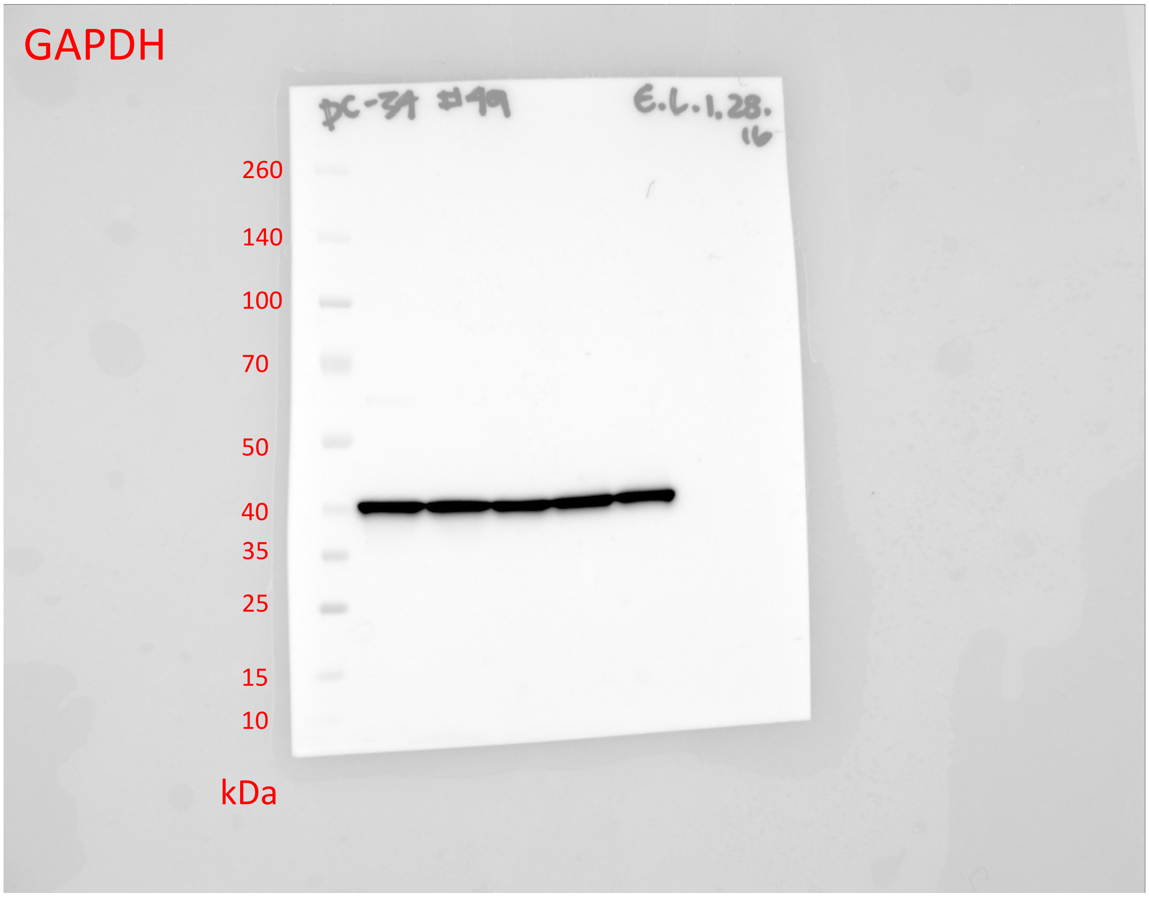

Supplementary Figure 83: Raw Western blot of GAPDH in Figure 3B.

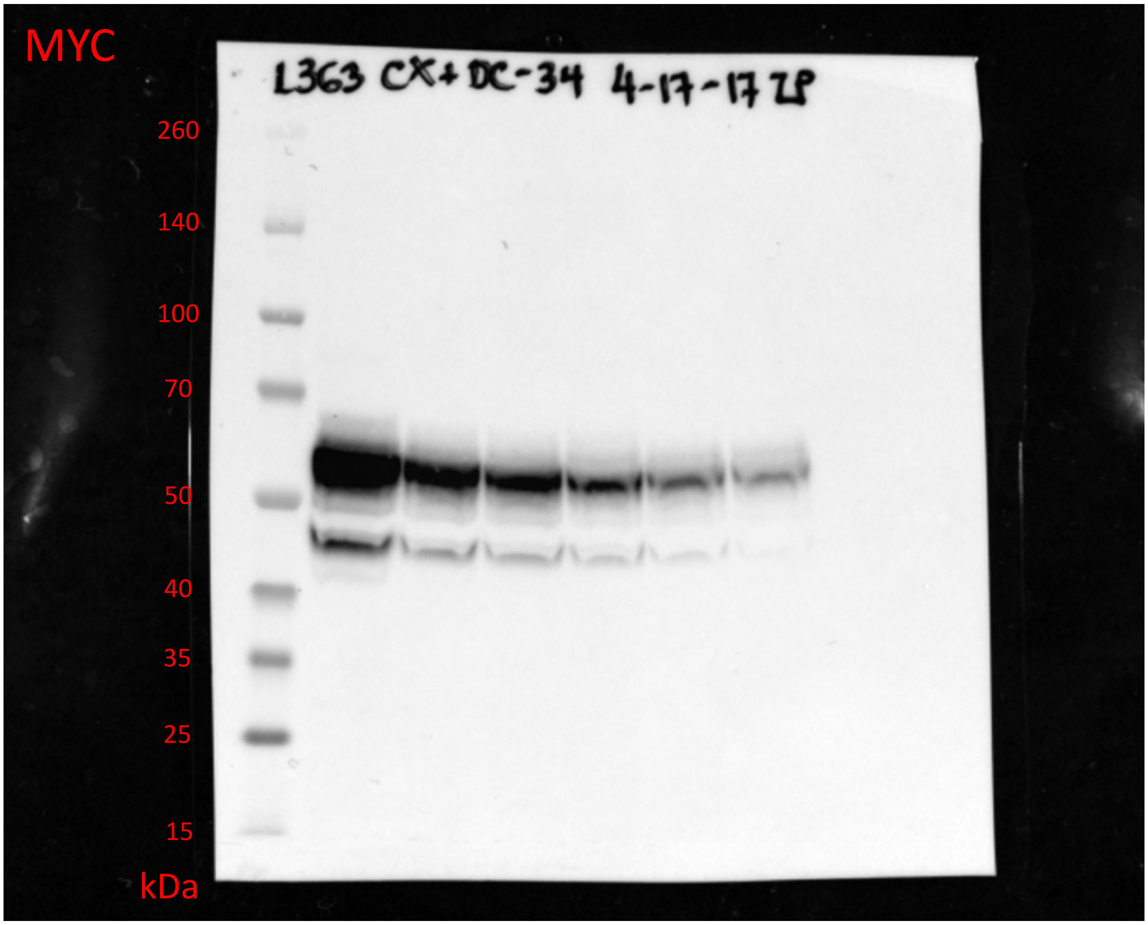

Supplementary Figure 84: Raw Western blot of MYC for CX in Figure 3D.

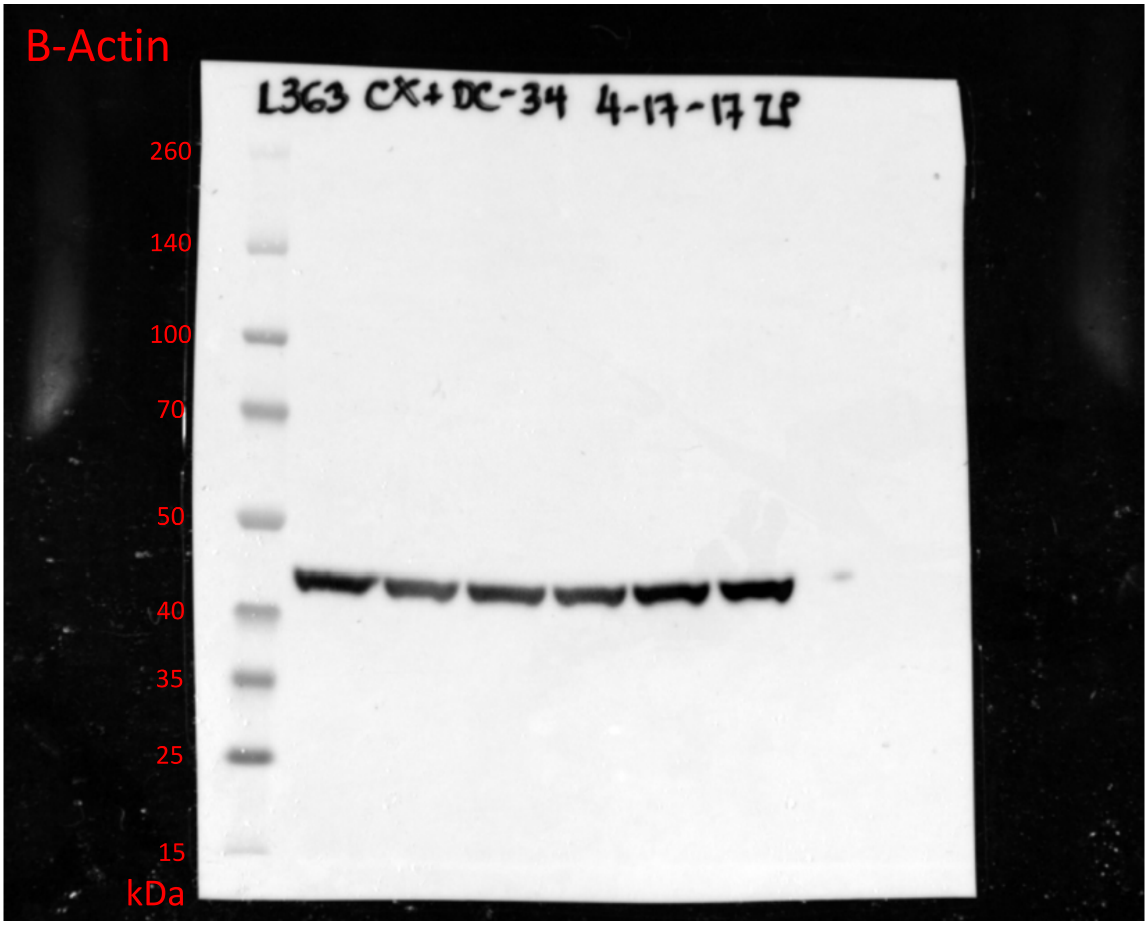

Supplementary Figure 85: Raw Western blot of Actin for CX in Figure 3D.

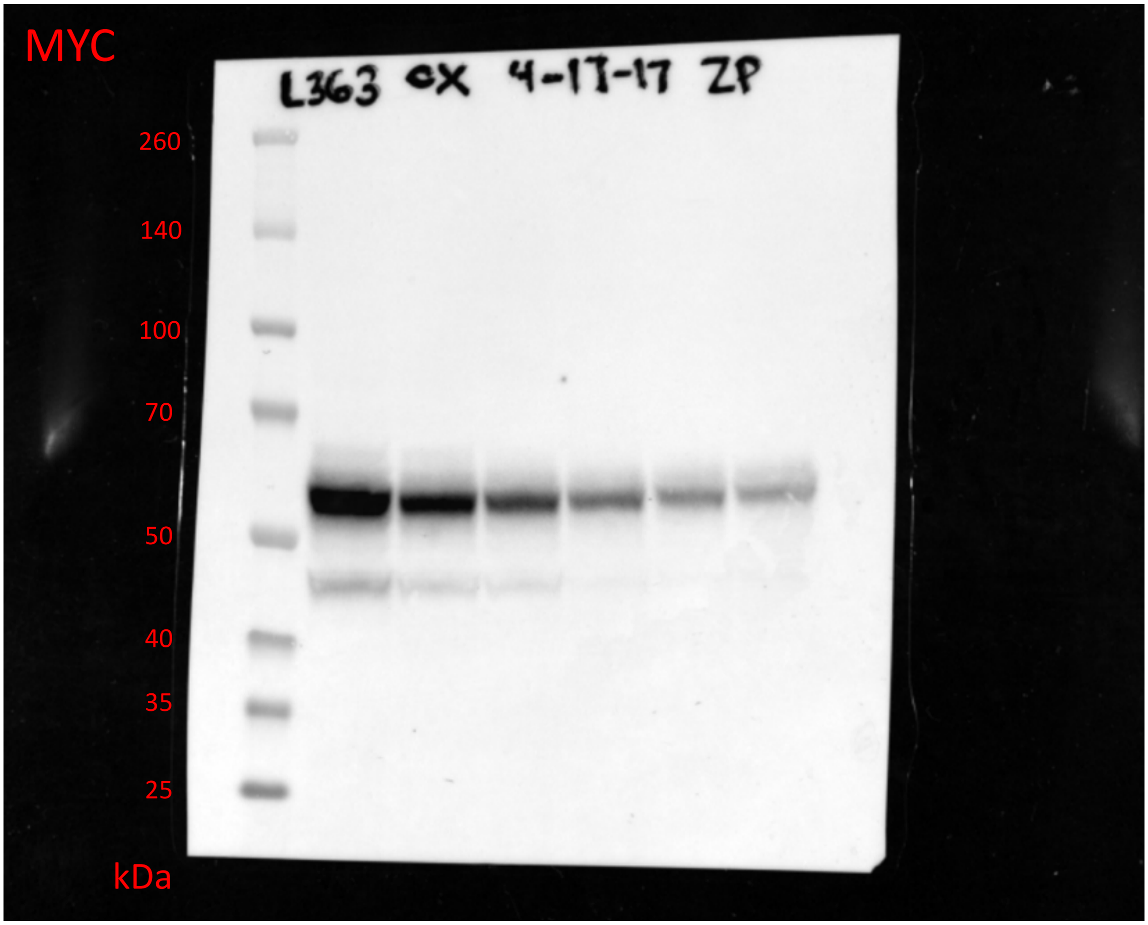

Supplementary Figure 86: Raw Western blot of MYC for CX + DC-34 in Figure 3D.

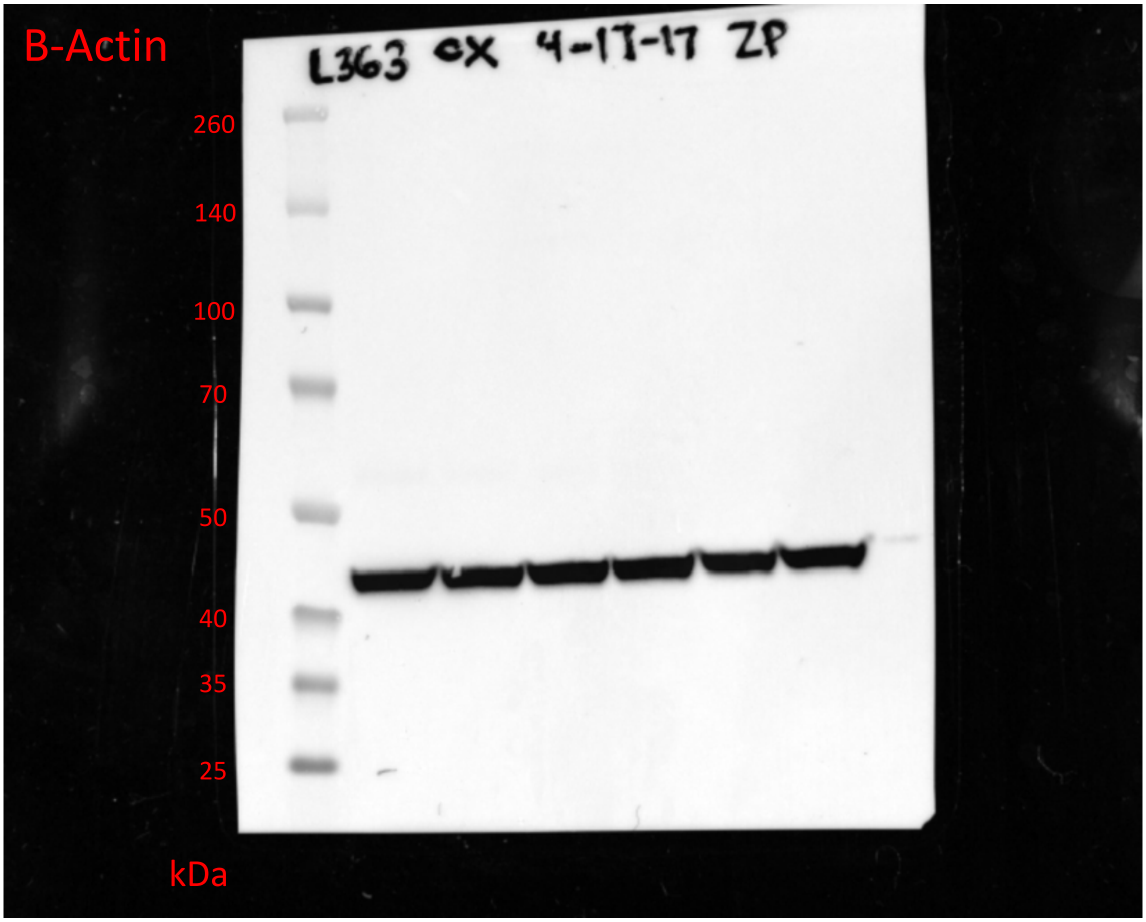

Supplementary Figure 87: Raw Western blot of Actin for CX + DC-34 in Figure 3D.

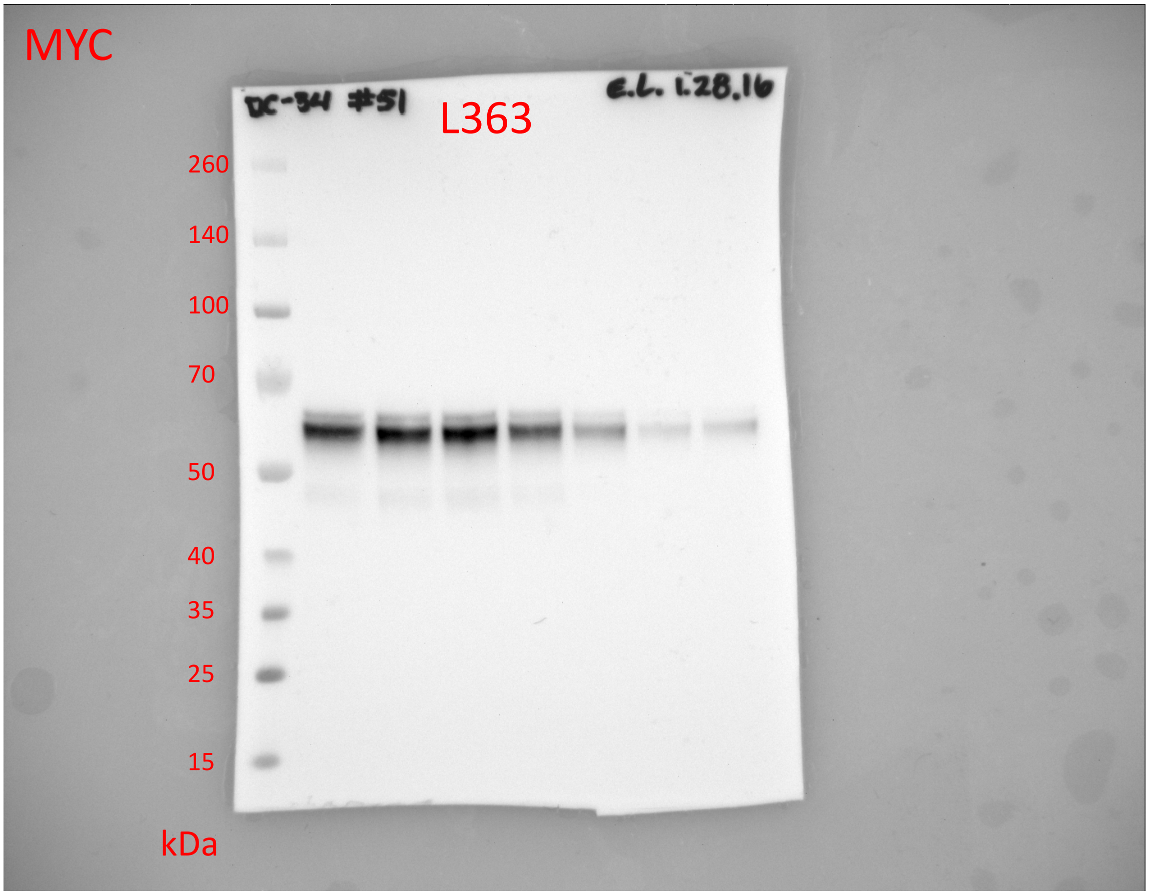

Supplementary Figure 88: Raw Western blot of MYC in L363 cells from Figure 3E.

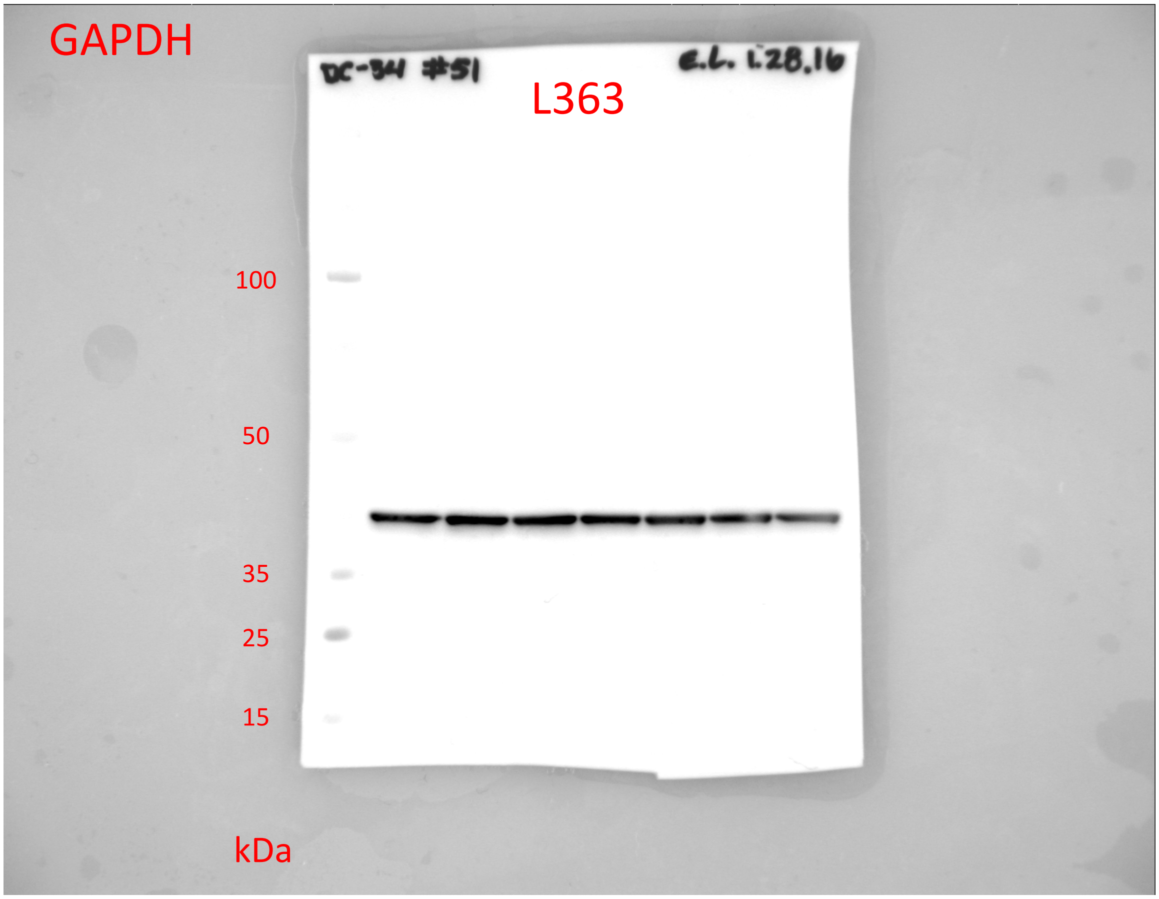

Supplementary Figure 89: Raw Western blot of GAPDH for L363 cells from Figure 3E.

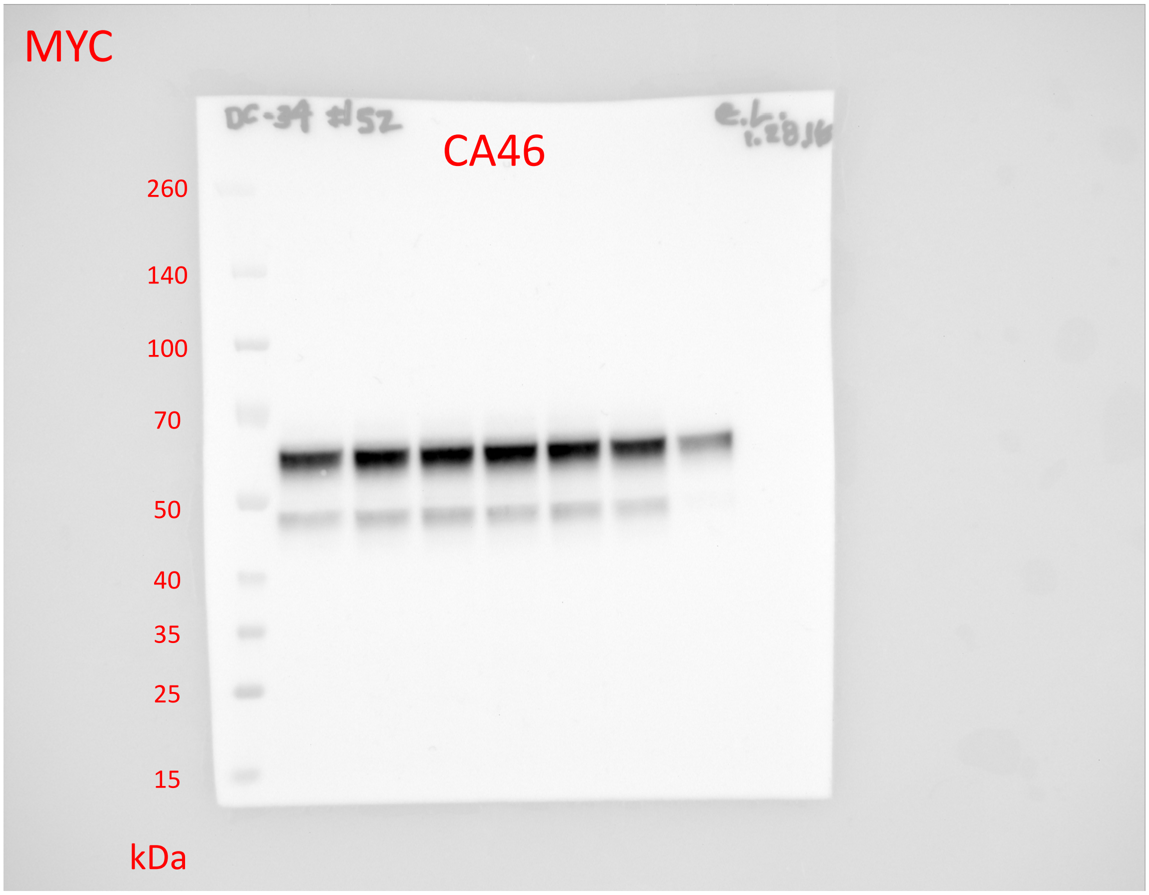

Supplementary Figure 90: Raw Western blot of MYC for CA46 cells from Figure 3E.

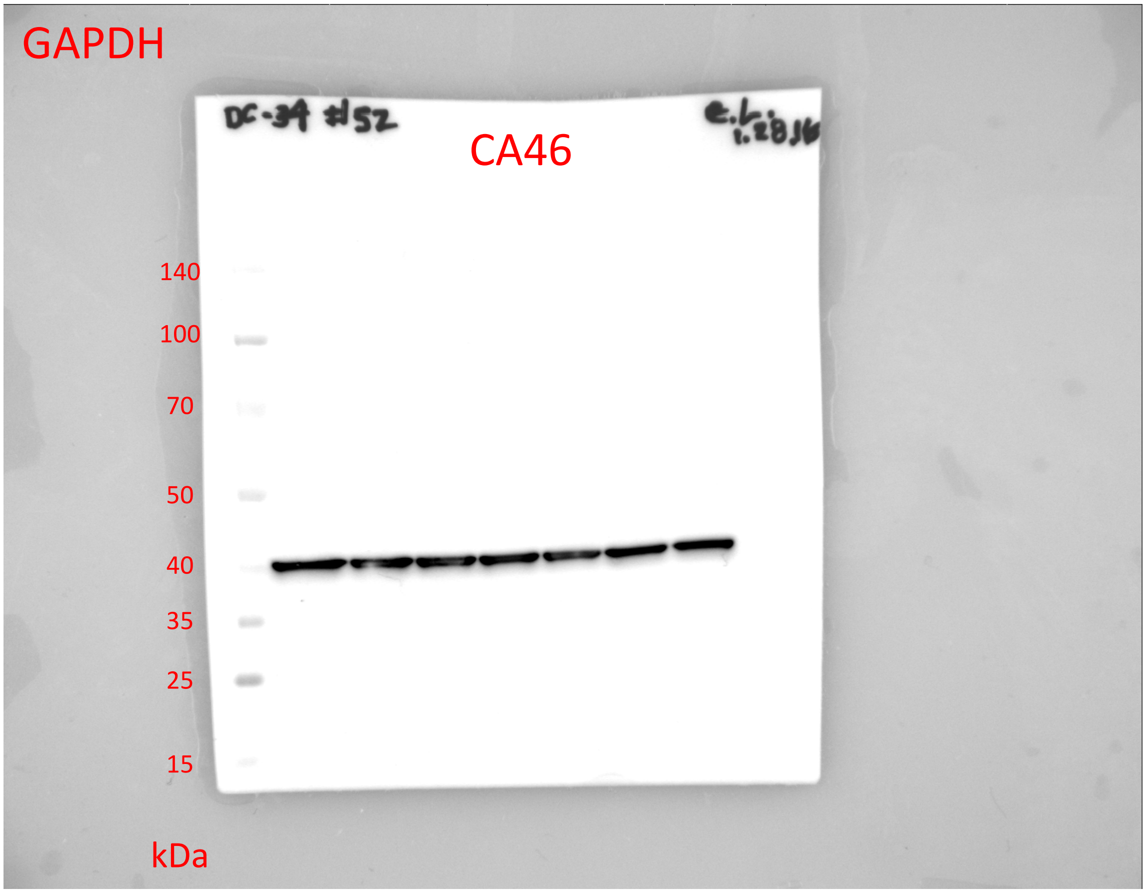

Supplementary Figure 91: Raw Western blot of GAPDH for CA46 cells from Figure 3E.

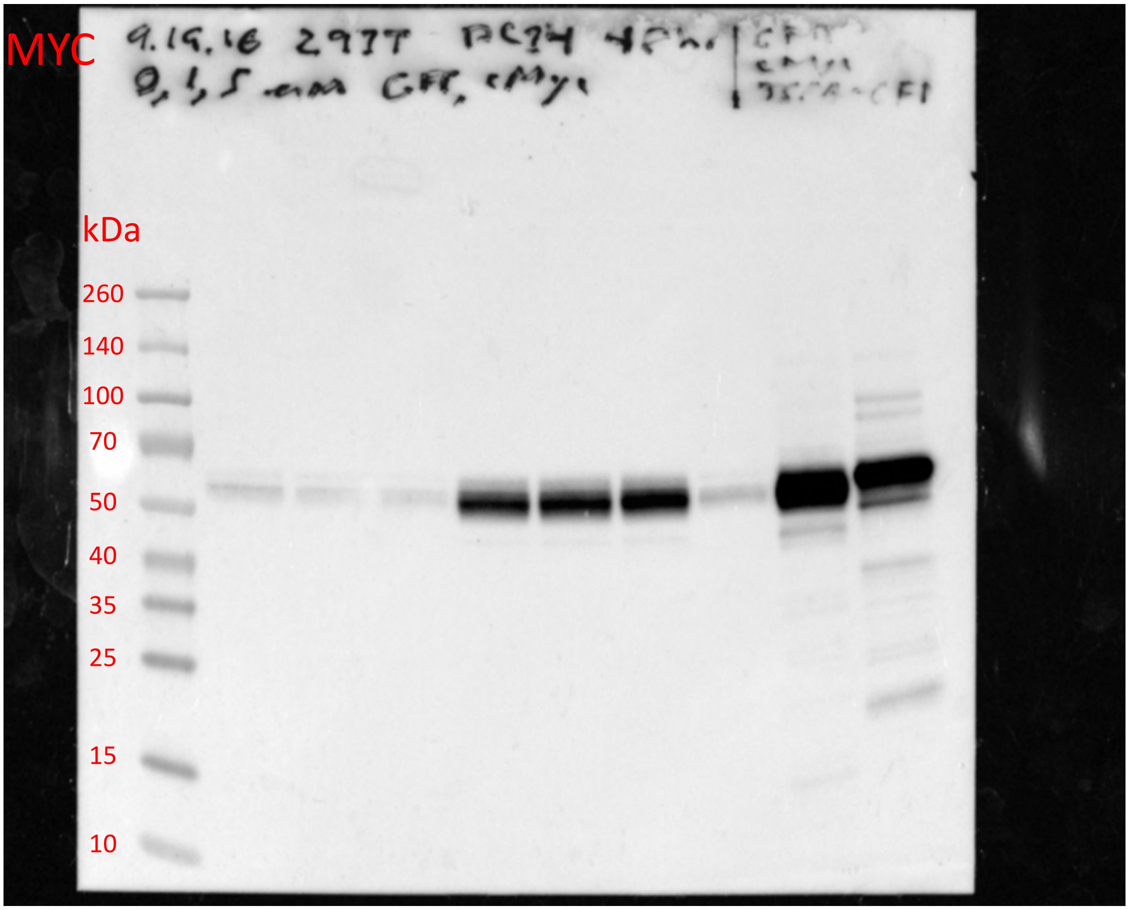

Supplementary Figure 92: Raw Western blot of 293T cells transiently transfected with either GFP or CMV-MYC plasmid (the CMV promoter lacks a MYC G4) (right) and dosed with different concentrations of DC-34 from Figure 3F. All western blots were exposed for less than 1 minute.

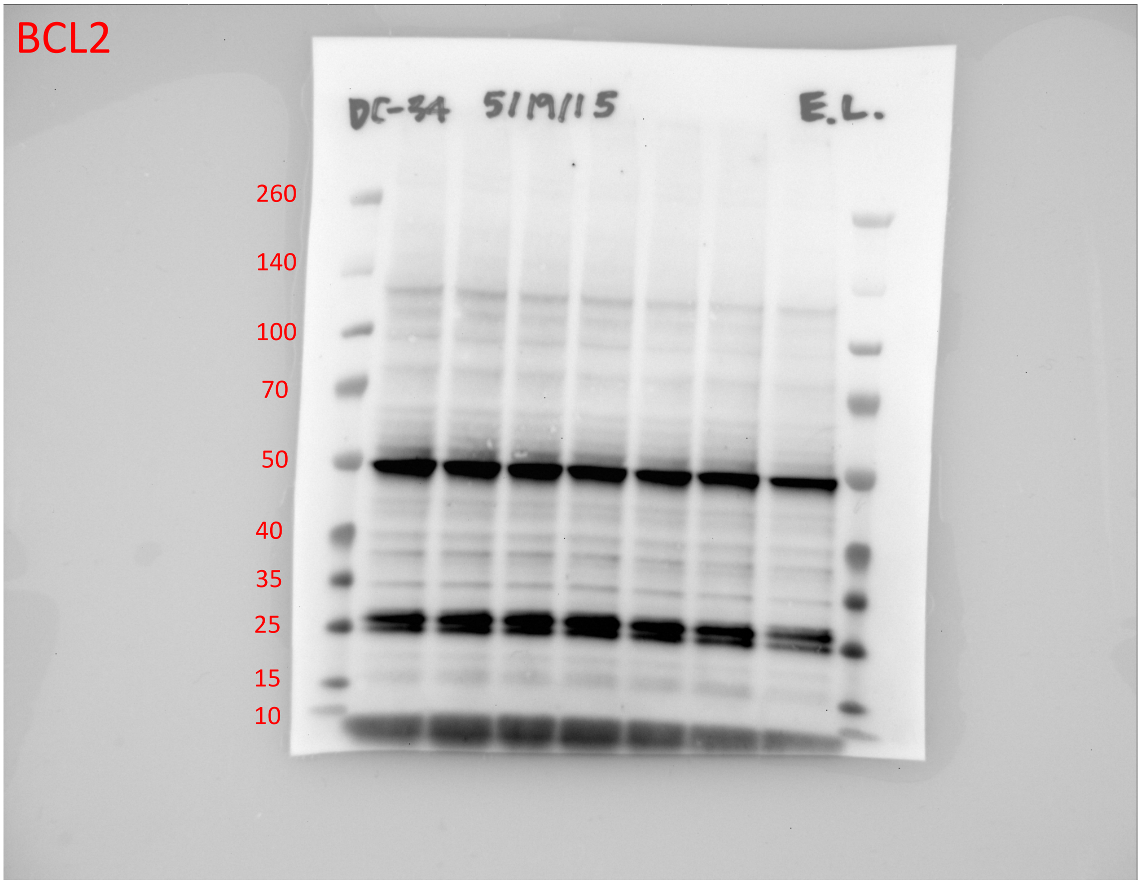

Supplementary Figure 93: Raw Western blot of BCL2 from Supplementary Figure 3.

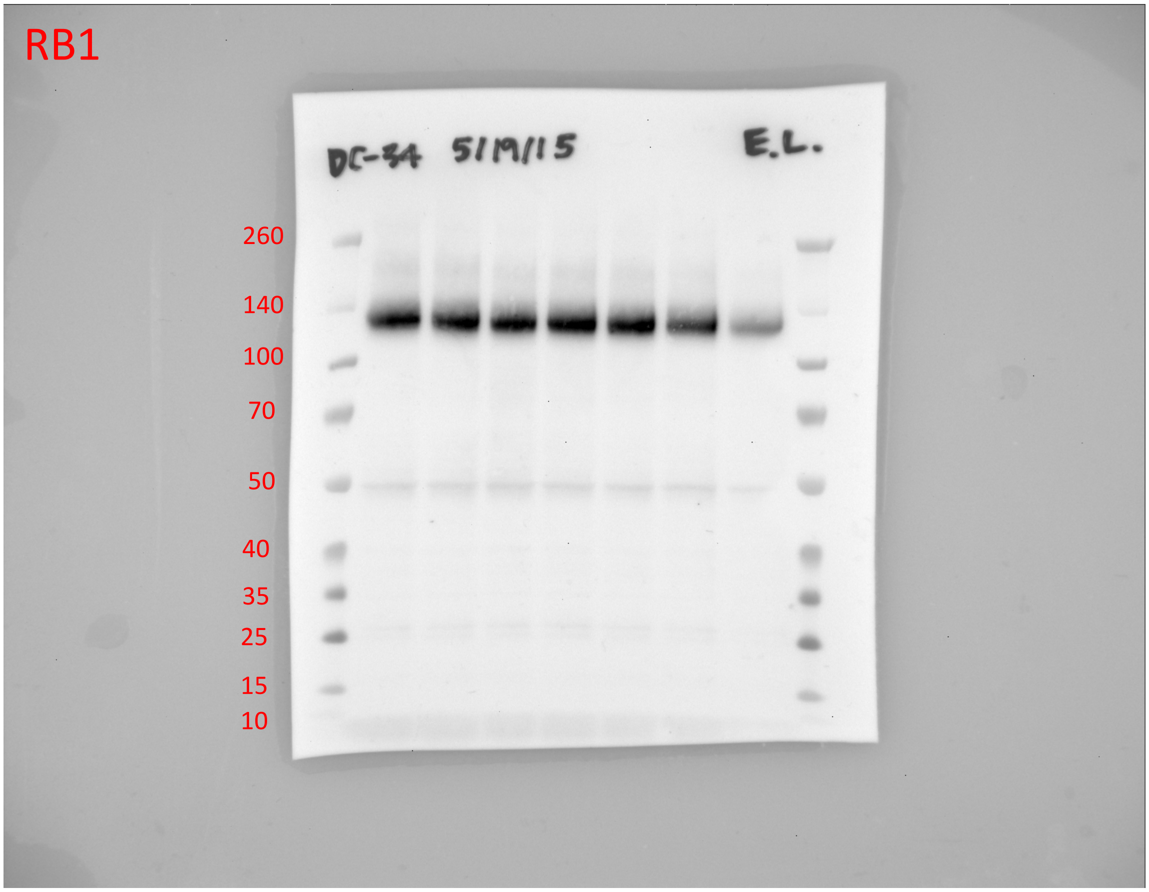

Supplementary Figure 94: Raw Western blot of RB1 from Supplementary Figure 3.

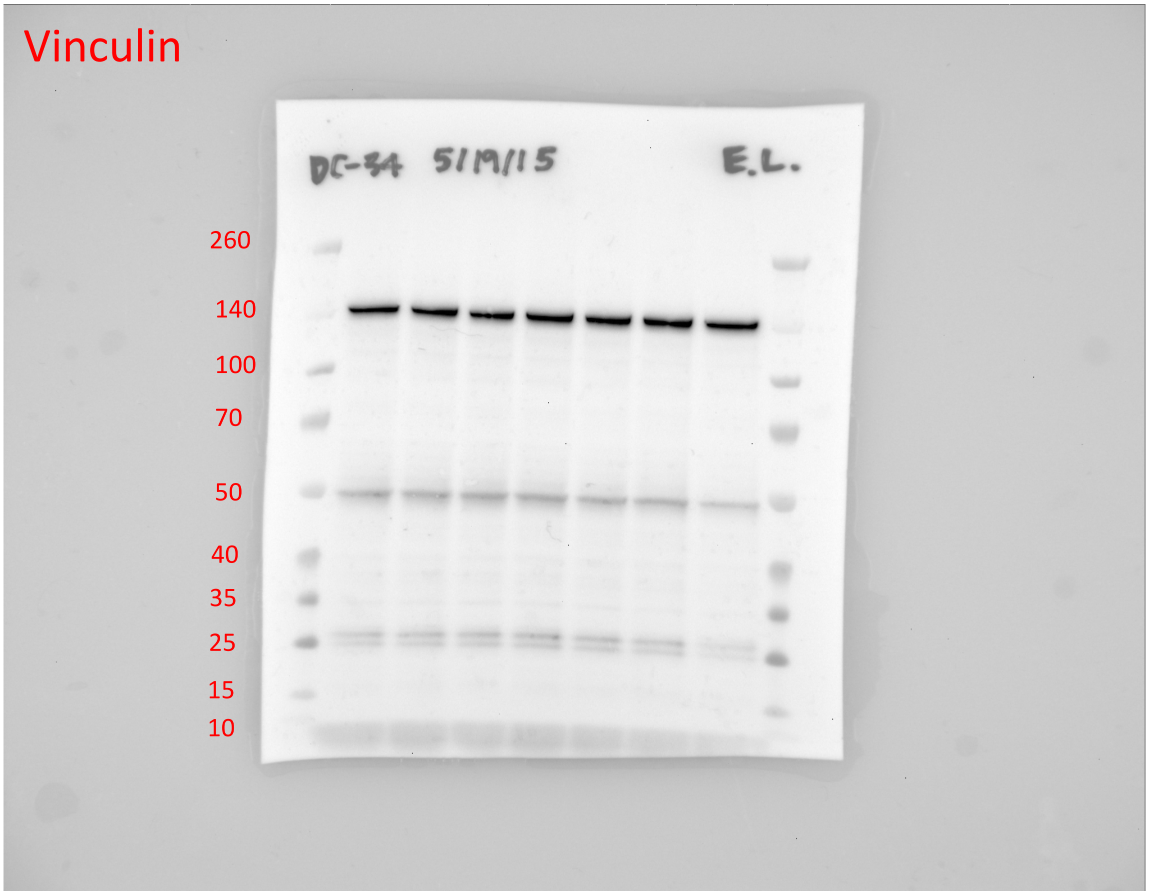

Supplementary Figure 95: Raw Western blot of Vinculin from Supplementary Figure 3.

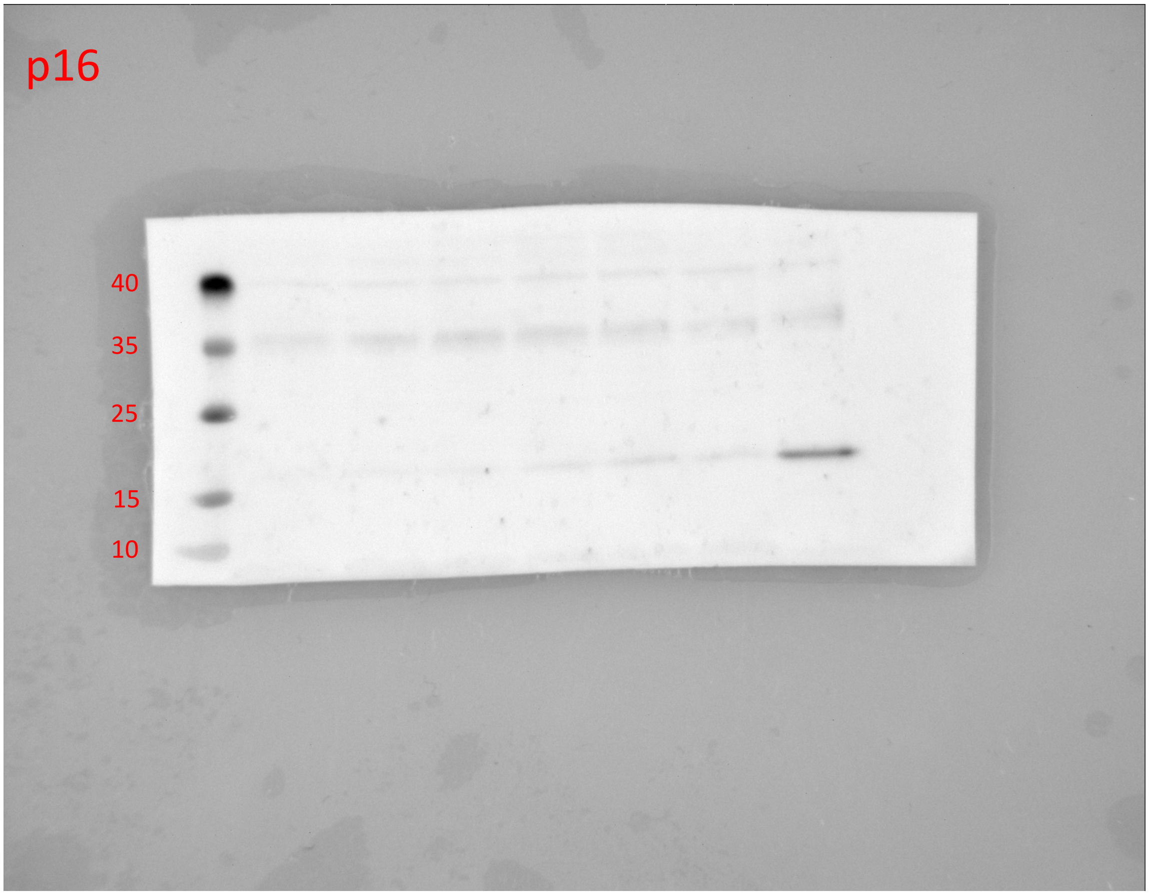

Supplementary Figure 96: Raw Western blot of p16 from Supplementary Figure 4.

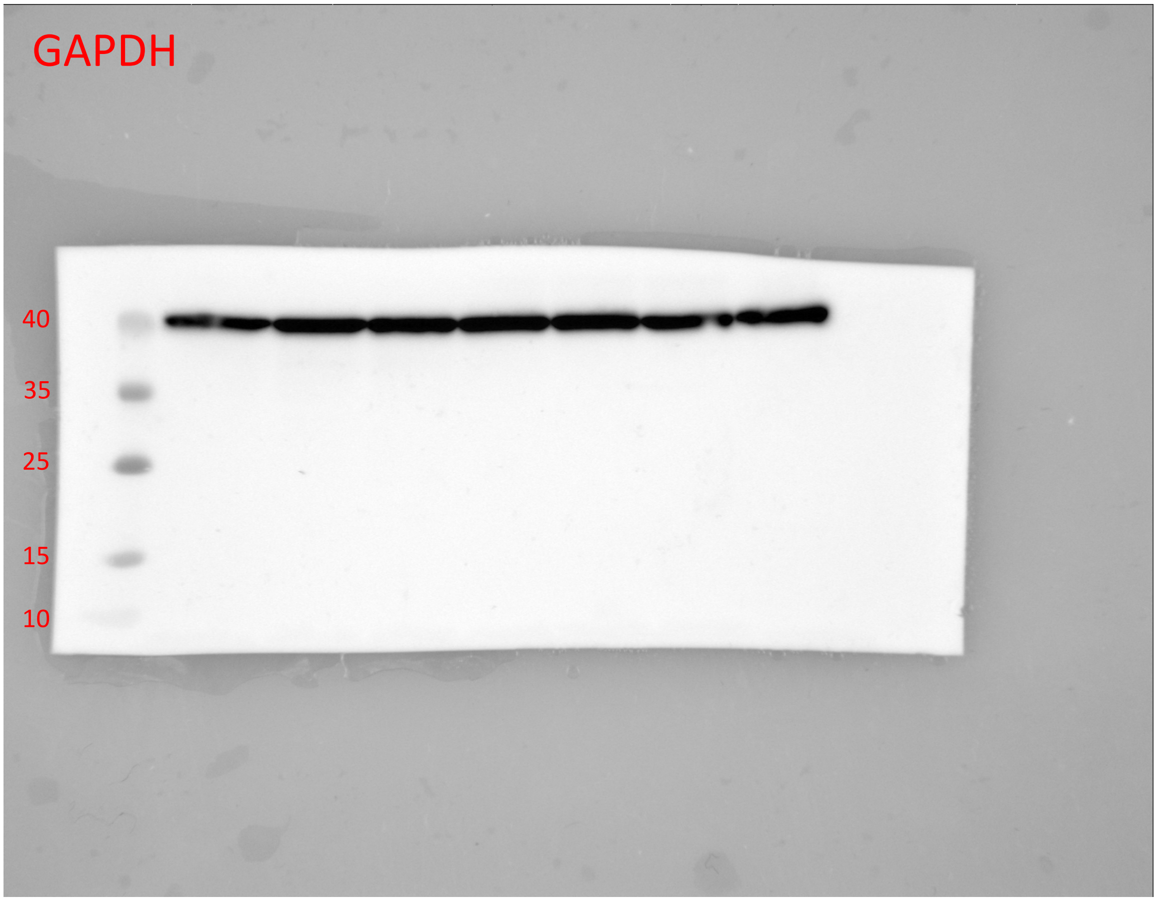

Supplementary Figure 97: Raw Western blot of GAPDH from Supplementary Figure 4.

References:

1. Chen, J.Q. *et al.* Absolute quantitation of endogenous proteins with precision and accuracy using a capillary Western system. *Anal Biochem* **442**, 97-103 (2013).
2. Delaglio, F. *et al.* Nmrpipe - a Multidimensional Spectral Processing System Based on Unix Pipes. *J Biomol Nmr* **6**, 277-293 (1995).
3. Bartels, C., Xia, T.H., Billeter, M., Guntert, P. & Wuthrich, K. The Program Xeasy for Computer-Supported Nmr Spectral-Analysis of Biological Macromolecules. *J Biomol Nmr* **6**, 1-10 (1995).
4. Bermejo, G.A., Clore, G.M. & Schwieters, C.D. Improving NMR Structures of RNA. *Structure* **24**, 806-815 (2016).
5. Schuttelkopf, A.W. & van Aalten, D.M.F. PRODRG: a tool for high-throughput crystallography of protein-ligand complexes. *Acta Crystallogr D* **60**, 1355-1363 (2004).
6. Pettersen, E.F. *et al.* UCSF chimera - A visualization system for exploratory research and analysis. *J Comput Chem* **25**, 1605-1612 (2004).
